# Regio- and stereoselective thiocyanatothiolation of alkynes and alkenes by using NH_4_SCN and *N*-thiosuccinimides†

**DOI:** 10.1039/d0ra06913b

**Published:** 2020-09-10

**Authors:** Liang Qi, Shiwen Liu, Linxia Xiao

**Affiliations:** Jiangsu Vocational College of Medicine Jie Fang South Road 283 th Yancheng 224000 China xiaolinxiaxlx@126.com; College of Textiles and Clothing, Yancheng Institute of Technology Yancheng 224051 China

## Abstract

A highly regioselective thiocyanatothiolation of alkynes and alkenes assisted by hydrogen bonding under simple and mild conditions is developed. Our thiocyanatothiolation reagents are readily available ammonium thiocyanate and *N*-thiosuccinimides. This metal-free system offers good chemical yields for a wide range of alkyne and alkene substrates with good functional group tolerance.

## Abstract 2

A highly regioselective thiocyanatothiolation of alkynes assisted by hydrogen bonding under simple and mild conditions is developed.

## Graphical abstract

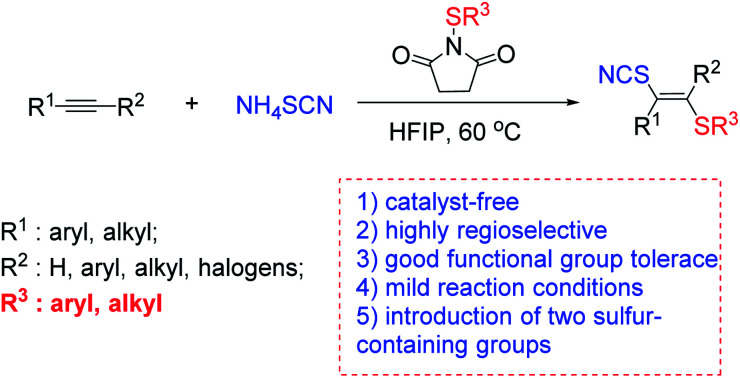

Sulfur-containing molecules are ubiquitous structural motifs and widely exist in natural products,^1,2^ pharmaceuticals^3,4^ and agrochemicals.^5–7^ Examples include the nonsteroidal anti-inflammatory drug Sulindac,^8^ the basal-cell carcinoma treatment drug Vismodegib,^9^ and drugs for the treatment of Parkinson's disease.^10^ Therefore, efficient introduction of sulfur into organic molecules has drawn much attention.^11–15^ And numerous approaches for the formation of C–S bonds have been developed.^16–20^ The most used organosulfur sources for the formation of C–S bonds are thiols and thiophenols, which have an unpleasant smell. Recently, inorganic metal sulfides have been extensively used to construct C–S bonds, such as sodium metabisulfite,^21^ K_2_S,^22^ Na_2_S^23^ and Na_2_S_2_O_3_.^24^ Compared to thiols and thiophenols, inorganic metal sulfides are cheaper and generally stable. Thus, introduction of sulfur-containing groups into molecules by using inorganic metal sulfides is one of the desired approaches. Among them, thiocyanates commonly serve as important precursors for the preparation of thioethers,^25^ trifluoromethyl sulfides,^26^ heteroaromatic compounds.^27^ In general, the sources of SCN used to introduce a sulfur-containing group into molecules are thiocyanate salts^28–35^ such as KSCN, NaSCN, AgSCN and NH_4_SCN. For example, thiocyanate salts were employed in thiocyanation of bromoalkenes *via* photocatalysis (Scheme 1a).^36^ Besides, the vinyl thiocyanates could be also obtained by thiocyanation of haloalkynes (Scheme 1b),^37^ iodothiocyanation of alkynes (Scheme 1c).^38^ Obviously, difunctionalization of alkynes is the most straightforward protocol to prepare vinyl thiocyanates.

**Scheme 1:**
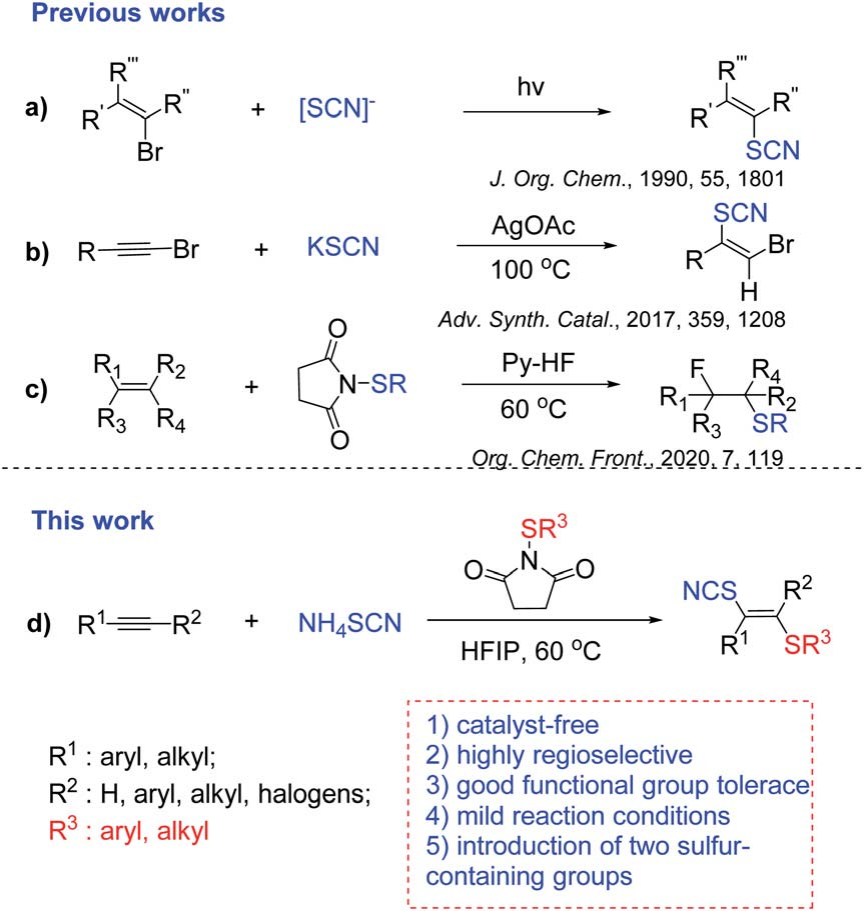
Methods for thiocyanatothiolation of alkynes and alkenes.

Recently, our group has focused on hydrogen-bonding network or cluster^39^ assisted transformations such as hydrofluorination of ynamides^40^ and alkenes,^41^ the addition of sulfonic acids to haloalkynes,^42^ fluorothiolation of alkenes,^20^ dihalogenation of alkynes^43^ and hydrochlorination of alkynes,^44–46^halothiolation of alkynes.^47^ Along this line, herein, we are glad to report a hydrogen bond network-enabled regio- and stereoselective thiocyanatothiolation of alkynes using NH_4_SCN and *N*-thiosuccinimides.

Initially, according to the previous report,^20^ we started the investigation of thiocyanatothiolation protocol using NH_4_SCN and *N*-(phenylthio)succinimide as thiolation reagents in DCM under air and carried out the reaction at 60 °C (Table 1). To our delight, the desired product 3a was obtained in 42% yield without any isomers found in the reaction mixture detected by GC-MS (Table 1, entry 1). Screening of solvents indicated that this transformation could not proceed in the polar solvents, such as acetone, THF, dioxane, i-PrOH, DMF (Table 1, entries 3–6) probably due to the solvation of electrophiles while moderate yield could be obtained in non-polar solvent (Table 1, entry 2). Strong hydrogen-bond donor solvents such as hexafluoro-2-propanol (HFIP), could form an H-bond network activating the electrophiles through a strong hydrogen bonding interaction.^48^ In order to enhance the H-bond interaction between the hydroxyl and 2, so AcOH was chosen to compare with HFIP (Table 1, entry 7). Along this line, hydrogen-bond donor solvents were used and further optimization of hydrogen-bond donor solvents indicated that HFIP was superior to AcOH and trifluoroethanol (Table 1, entries 7–9). Moreover, a screening of thiocyanate salts showed that NH_4_SCN was the best SCN source for this transformation compared with lithium thiocyanate, sodium thiocyanate and potassium thiocyanate (Table 1, entries 10–12). Additionally, decreasing the temperature from 60 °C to room temperature resulted in a lower yield (Table 1, entry 13) and the reaction yield was not improved significantly by raising the temperature from 60 °C to 80 °C (Table 1, entry 14).

**Table 1:** Optimization for the reaction conditions

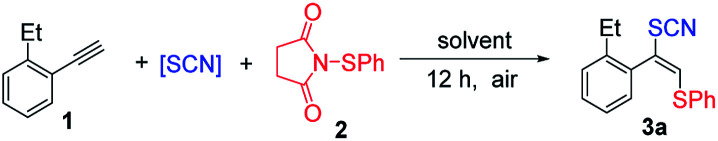				
Entry^a^	[SCN]	Solvent	Temp. (°C)	Yield^b^ (%)
1	NH_4_SCN	DCM	60	42
2	NH_4_SCN	DCE	60	47
3	NH_4_SCN	THF	60	0
4	NH_4_SCN	Acetone	60	0
5	NH_4_SCN	DMF	60	0
6	NH_4_SCN	iPrOH	60	0
7	NH_4_SCN	AcOH	60	24
8	NH_4_SCN	TFE	60	18
**9**	**NH**_**4**_ **SCN**	**HFIP**	**60**	**87**
10	LiSCN	HFIP	60	36
11	NaSCN	HFIP	60	42
12	KSCN	HFIP	60	49
13	NH_4_SCN	HFIP	25	63
14	NH_4_SCN	HFIP	80	83

a Reaction conditions: 1 (0.1 mmol), 2 (0.12 mmol), NH_4_SCN (0.2 mmol), solvent (0.5 mL), under air for 12 h at 60 °C.

b Determined by GC.

With the optimized conditions in hand, we next turned our attention to explore the substrate scope (Table 2). Firstly, *N*-(*p*-methoxyphenylthio)succinimide was used as electrophile to explore the scope of alkynes. In general, the reaction proceeded well to provide the desired products 3 in moderate to excellent yields with satisfactory regio- and stereoselectivity. Diverse aryl alkynes containing electron-donating groups such as isopropyl, hydroxy, methoxy, hydroxyethyl, *tert*-butyl and trifluoromethoxy groups (Table 2, 3e–3g and 3m–3p) at the *ortho*, *meta*, or *para* positions of aryl rings all reacted with *N*-thiosuccinimides to give the corresponding adducts in moderate to excellent yields. Besides, halide substitutes (F, Cl, Br) (Table 2, 3b, 3j–3l and 3w) and electron-withdrawing groups such as cyano and ester (Table 2, 3h and 3i) on phenyl ring were well tolerated. Furthermore, asymmetric or symmetrical internal alkynes also could be transformed into vinyl thiocyanates (Table 2, 3r, 3s and 3w) without any isomers. Remarkably, vinyl thiocyanates containing halogens could be obtained by using haloalkynes (Table 2, 3t–3v). Additionally, slightly low yields were observed for fused aromatic such as naphthalene and heterocyclic aromatic (Table 2, 3x and 3y). Due to good functional-group tolerance, derivatives of diacetone-d-glucose (Table 2, 3z), natural products l-menthol (Table 2, 3aa) and pharmaceuticals such as zaltoprofen (Table 2, 3ab) also worked well.

**Table 2:** Scope for thiocyanatothiolation of alkynes and *N*-arylsulfenylsuccinimides^a^^,^^b^

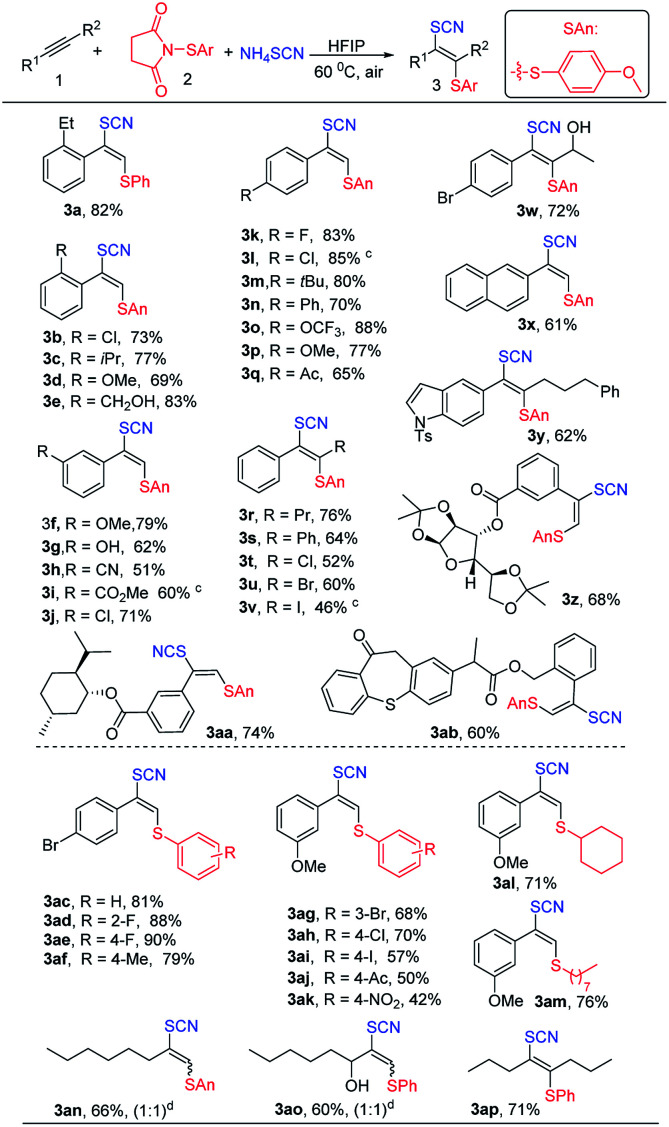

a Reaction conditions: 1 (0.1 mmol), 2 (0.12 mmol), NH_4_SCN (0.2 mmol), HFIP (0.5 mL), under air for 12 h at 60 °C.

b Isolated yield.

c Ar = Ph.

d Determined by NMR.

Next, we started to explore the scope of *N*-arylsulfenylsuccinimides. Various *N*-arylsulfenylsuccinimides can be obtained easily by the method in ESI.† To our delight, the introduction of electron-donating groups or halide substitutes to the phenyl ring of *N*-arylsulfenylsuccinimides had little influence on this reaction, providing the corresponding products in 57–90% yields (Table 2, 3ac–3ak) while electron-withdrawing groups on the phenyl ring such as acetyl or nitro resulted in lower yields (Table 2, 3aj and 3ak) probably due to the decrease of electrophilicity of *N*-arylsulfenylsuccinimides. Notably, the scope of *N*-sulfenylsuccinimides could be extended to *N*-alkylsulfenylsuccinimides (Table 2, 3al and 3am), affording the desired products with good yields and high selectivity. Unfortunately, the thiocyanatothiolated products (Table 2, 3an and 3ao) with poor stereoselectivity (Z/*E* = 1 : 1) were obtained when the unsymmetrically aliphatic alkynes were employed. We speculated that the steric hindrance of the aliphatic side chain maybe is small, resulting in a low Z/*E* ratios.

Encouraged by the success of thiocyanatothiolation of alkynes, we next turned our focus to the thiocyanatothiolation of alkenes. Under the optimized conditions, no product was observed. And the HFIP as nucleophile replaced the NH_4_SCN, giving hexafluoroisopropanol thiolated product. As result, non-polar solvent DCE was used to avoid the hexafluoroisopropanol thiolat of alkenes. To our delight, the thiocyanatothiolation of alkenes could proceed smoothly though moderate or lower chemical yields were obtained. Among them, aromatic alkenes gave moderate yields without any isomers (Table 3, 5a–5e) and aliphatic alkenes gave lower yields (Table 3, 5f–5h).

**Table 3:** Scope for thiocyanatothiolation of alkenes^a^^,^^b^

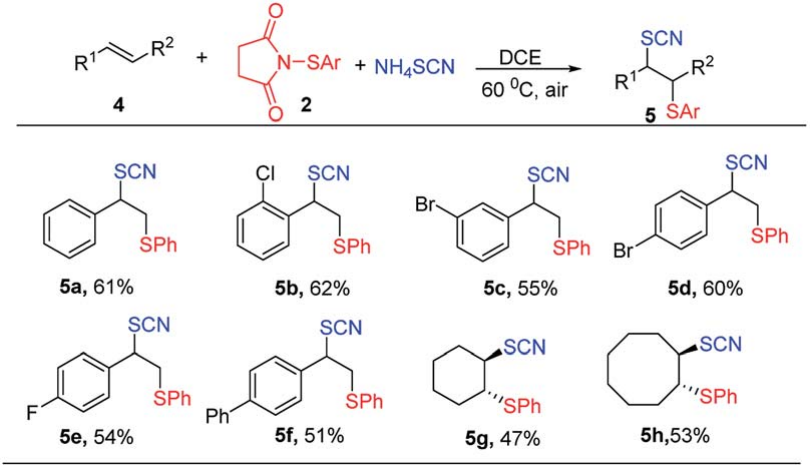

a Reaction conditions: 4 (0.1 mmol), 2 (0.12 mmol), NH_4_SCN (0.2 mmol), DCE (1.0 mL), under air for 12 h at 60 °C.

b Isolated yield.

To demonstrate the scalability of this protocol, a gram-scale reaction of 1,1′-biphenyl-4-ethynyl (6 mmol) with *N*-(4-bromo thio)succinimide was carried out, and the corresponding product 3aq was obtained in 62% yield (Scheme 2).

**Scheme 2:**
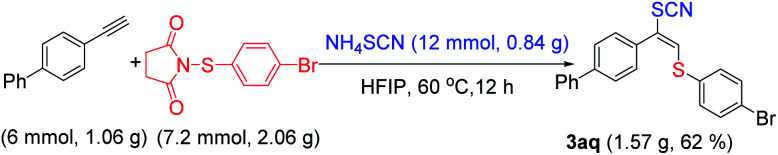
Gram-scale preparation of 3aq.

To identify the configuration, the single crystal of product 3aq was cultivated by solvent evaporation. And the regio- and stereoselectivity of products were further confirmed the X-ray crystallographic analysis of the obtained product 3aq (Fig. 1).

**Fig. 1:**
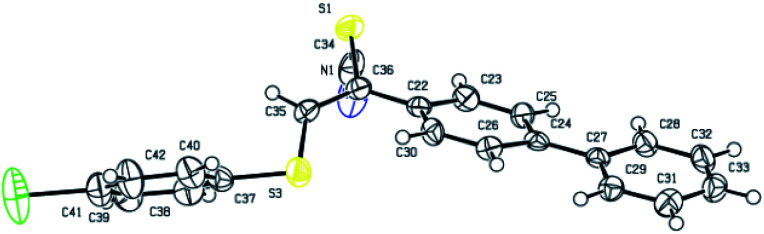
Single crystal structure of 3aq.

Based on our previous work,^47^ a plausible reaction pathway was proposed in Scheme 3. The interaction of HFIP hydrogen bonding linear aggregates^48^ with sulfenylation reagent 2a may strongly activate the sulfenylation reagent, which generates the active intermediate B (Scheme 3). Sequentially, a sulfonium C is produced from intermediate B with an alkyne, followed by a nucleophilic attack of SCN anion to obtain the products 3.

**Scheme 3:**
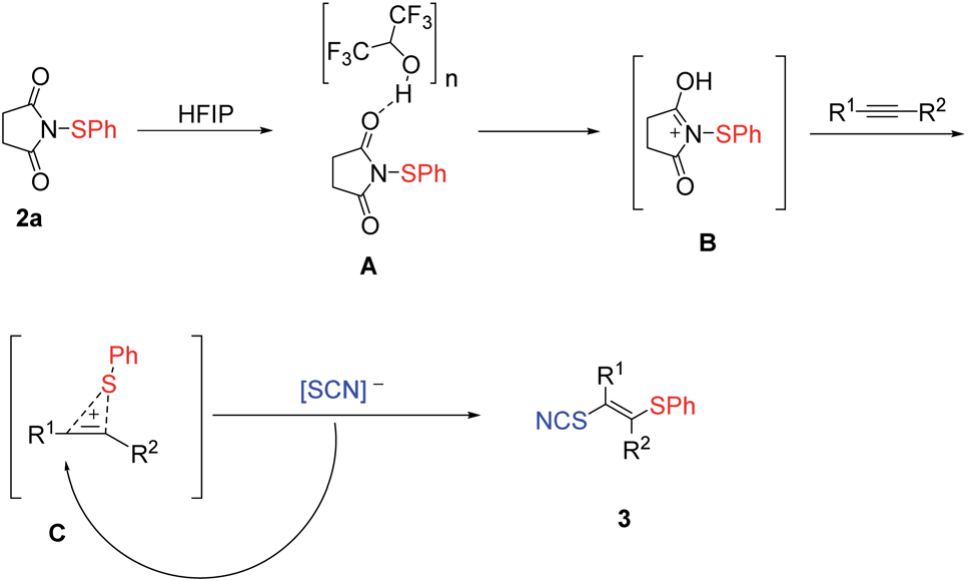
Plausible mechanism.

## Conclusions

In summary, we have developed a widely applicable regio- and stereoselective thiocyanatothiolation of alkynes and alkenes under simple and mild conditions. This metal-free system offers good chemical yields and functional group tolerance. At present, the fluorinated reagent HFIP, which is not a green solvent, is indeed a limitation of this method, but as scientific research continues, we believe that green fluorinated reagents can be discovered. Other similar thiolation systems are currently investigated in our laboratory.

## Conflicts of interest

There are no conflicts to declare.

## Supplementary Material

RA-010-D0RA06913B-s001

RA-010-D0RA06913B-s002
