# Supplementary material for: Regio- and stereoselective thiocyanatothiolation of alkynes and alkenes by using NH4SCN and N-thiosuccinimides
Source: RSC Adv. 2020 Sep 10;10(55):33450–4. doi: 10.1039/d0ra06913b (PMC9056709; doi:10.1039/d0ra06913b)
Supplement: RA-010-D0RA06913B-s001 [file RA-010-D0RA06913B-s001.pdf]

Electronic Supplementary Material (ESI) for RSC Advances.
This journal is © The Royal Society of Chemistry 2020

Supporting information

Regio- and Stereoselective Thiocyanatothiolation of Alkynes and Alkenes by Using NH_4SCN and *N*-thiosuccinimides

Liang Qi,[a] Shiwen Liu, [b] Linxia Xiao * [a]

[a] Jiangsu Vocational College of Medicine ,Jie Fang South Road 283 th, Yancheng, 224000, China.

[b] College of Textiles and Clothing, Yancheng Institute of Technology, Yancheng, 224051, China. Email:xiaolinxiaxlx@126.com

Table of contents

1. General.....3
2. General procedure for the preparation of *N*-thiosuccinimides.....3
3. General procedure for regioselective thiocyanatothiolation of alkynes.....3
4. Characterization data for the products 3.....4
5. General procedure for regioselective thiocyanatothiolation of alkenes.....17
6. Characterization data for the products 5.....17
7. X-ray Crystallographic Analysis of product 3aq.....20
8. Copies of NMR Spectra.....22
9. References.....147

1. General

Commercial reagents and solvents were obtained from the commercial providers and used without further purification. The products were purified using a commercial flash chromatography system or a regular glass column. TLC was developed on silica gel 60 F254 glass plates. ^{1}H NMR (400 MHz) and ^{13}C NMR (100 MHz) spectra were recorded on a Bruker NMR apparatus. The chemical shifts are reported in δ (ppm) values (^{1}H and ^{13}C NMR relative to $CHCl_3$, δ 7.26 ppm for ^{1}H NMR and δ 77.0 ppm for ^{13}C NMR). Or alternatively, ^{1}H NMR chemical shifts were referenced to tetramethylsilane signal (0 ppm). Multiplicities are recorded by s (singlet), d (doublet), t (triplet), m (multiplet) and br (broad). Coupling constants (*J*), are reported in Hertz (Hz). GC analyses were performed using a Shimadzu GC-2010-ultra gas chromatography–mass spectrometry instrument equipped with a Shimadzu AOC-20s autosampler.

2. General procedure for the preparation of *N*-thiosuccinimides

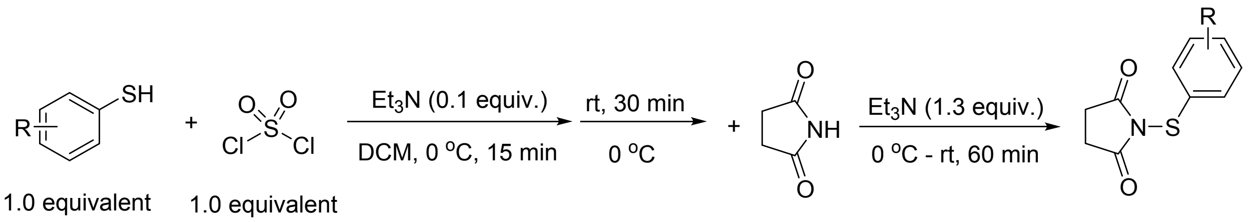

Sulfuryl chloride (1.0 equivalent) was added dropwise to a solution of thiol (1.0 equivalent) and triethylamine (0.1 equivalents) in dichloromethane (1M) at 0 °C. After stirring for 15 minutes, the mixture was warmed to room temperature and stirred for 30 minutes and then cooled to 0 °C. The resulting solution was transferred dropwise via cannula to a solution of succinimide (1.0 equivalent) in dichloromethane (1M) and triethylamine (1.3 equivalents) at 0 °C, and the mixture was then warmed to room temperature over 1 hour. The solution was diluted with water and extracted with an equal volume of dichloromethane before being dried over sodium sulfate. Evaporation of the solvent gave crude product that was purified by using flash column chromatography.[1]

3. General procedure for regioselective thiocyanatothiolation of alkynes

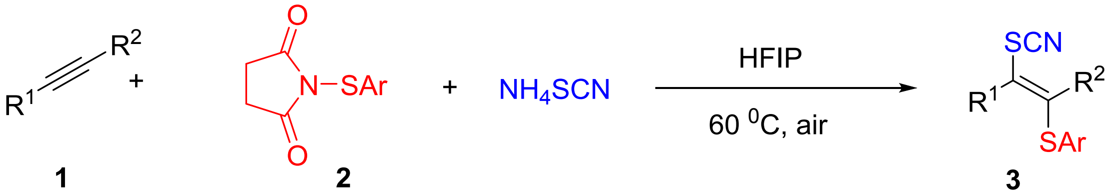

An oven-dried glass vial was charged with *N*-thiosuccinimides (0.12 mmol), NH_4SCN (0.20 mmol)

and alkynes (0.10 mmol). Then, HFIP (0.5 mL) was added and the mixture was heated to 60℃ and stirred for 12 h under air. After that, water was added to quench the reaction, and the resulting aqueous mixture was extracted with ethyl acetate (3 x 5 mL). The combined organic layers were dried over Na_2SO_4 and concentrated. The crude product was further purified by silica gel column chromatography.

4. Characterization data for the products 3

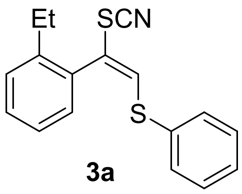

3a

(*E*)-(2-(2-ethylphenyl)-2-thiocyanatovinyl)(phenyl)sulfane (3a). Yellow oil (24 mg, 82%). ^{1}H NMR (600 MHz, $CDCl_3$) δ 7.44 – 7.37 (m, 6H), 7.35–7.30 (m, 3H), 7.15 (s, 1H), 2.77 (q, J = 7.6 Hz, 2H), 1.34 (t, J = 7.6 Hz, 3H). ^{13}C NMR (151 MHz, $CDCl_3$) δ 142.65, 135.98, 133.62, 133.36, 130.47, 130.24, 129.90, 129.51, 129.06, 128.08, 126.59, 118.38, 109.91, 25.84, 15.08. HRMS (EI) Calculated for $C_{17}H_{15}NS_2$ (M^+) 297.0646, found 297.0641.

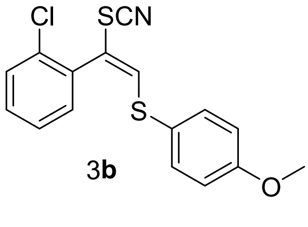

3**b**

(*E*)-(2-(2-chlorophenyl)-2-thiocyanatovinyl)(4-methoxyphenyl)sulfane (3b). Yellow oil (24 mg, 73%). ^{1}H NMR (600 MHz, $CDCl_3$) δ 7.50 – 7.49 (m, 2H), 7.41 – 7.36 (m, 3H), 7.21 (s, 1H), 6.92 (d, J = 8.7 Hz, 2H), 3.83 (s, 3H). ^{13}C NMR (151 MHz, $CDCl_3$) δ 160.62, 143.65, 137.87, 135.59, 134.02, 129.41, 127.25 , 123.11, 115.29, 113.17, 109.94, 55.51.HRMS (EI) Calculated for $C_{16}H_{12}ClNOS_2$ (M^+) 333.0049, found 333.0041.

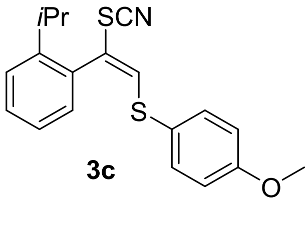

3c

(*E*)-(2-(2-isopropylphenyl)-2-thiocyanatovinyl)(4-methoxyphenyl)sulfane (3c). Yellow oil (26 mg, 77%). ^{1}H NMR (400 MHz, $CDCl_3$) δ 7.43 – 7.41 (m, 2H), 7.33 (d, *J* = 8.8 Hz, 2H), 7.29 – 7.23 (m, 2H), 7.03 (s, 1H), 6.88 (d, *J* = 8.8 Hz, 2H), 3.81 (s, 3H), 3.10 (dt, *J* = 13.7, 6.8 Hz, 1H), 1.30 (d, *J* = 16.4 Hz, 6H). ^{13}C NMR (151 MHz, $CDCl_3$) δ 160.10, 147.62, 138.56, 133.48, 132.83, 130.35, 129.90, 126.53 126.46, 123.91, 116.50, 115.09, 110.10, 55.47, 30.59, 24.44. HRMS (EI) Calculated for $C_{19}H_{19}NOS_2$ (M^+) 341.0908, found 341.0903.

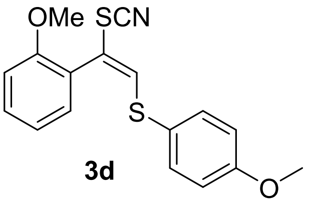

3d

(*E*)-(4-methoxyphenyl)(2-(2-methoxyphenyl)-2-thiocyanatovinyl)sulfane (3d). Yellow oil (22 mg, 69%). ^{1}H NMR (400 MHz, $CDCl_3$) δ 7.44 – 7.35 (m, 4H), 7.08 (s, 1H), 7.06 – 7.02 (m, 1H), 6.99 (d, *J* = 8.3 Hz, 1H), 6.90 – 6.86 (m, 2H), 3.91 (s, 3H), 3.81 (s, 3H). ^{13}C NMR (151 MHz, $CDCl_3$) δ 160.05, 156.66, 140.94, 133.55, 131.24, 130.70, 124.11, 123.86, 120.79, 115.00, 114.40 , 111.48, 114.43 55.73, 55.45. HRMS (EI) Calculated for $C_{17}H_{15}NO_2S_2$ (M^+) 329.0544, found 329.0551.

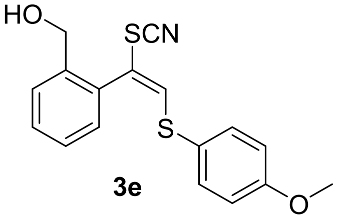

3e

(*E*)-(2-(2-((4-methoxyphenyl)thio)-1-thiocyanatovinyl)phenyl)methanol (3e). Yellow oil (27 mg, 83%). ^{1}H NMR (600 MHz, $CDCl_3$) δ 7.62 (d, *J* = 7.6 Hz, 1H), 7.48 (d, *J* = 7.5, 1H), 7.45 – 7.40 (m, 1H), 7.38 – 7.31 (m, 3H), 7.07 (s, 1H), 6.89 (d, *J* = 8.8 Hz, 2H), 4.78 (d, *J* = 6.2 Hz, 2H), 3.82 (s, 3H), 1.95 (t, *J* = 6.4 Hz, 1H). ^{13}C NMR (150 MHz, $CDCl_3$) δ 160.34, 140.39, 139.18, 133.76, 133.25, 130.29, 129.82, 128.92, 128.70, 123.07, 115.18, 115.11, 110.34, 62.88, 55.48. HRMS (EI) Calculated for $C_{17}H_{15}NO_2S_2$ (M^+) 329.0544, found 329.0547.

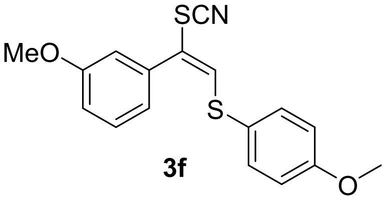

3f

(*E*)-(4-methoxyphenyl)(2-(3-methoxyphenyl)-2-thiocyanatovinyl)sulfane (3f). Yellow oil (26 mg, 79%). ^{1}H NMR (600 MHz, $CDCl_3$) δ 7.41 – 7.36 (m, 3H), 7.21 – 7.18 (m, 1H), 7.15 – 7.12 (m, 1H), 7.08 (s, 1H), 6.96 – 6.93 (m, 1H), 6.92 – 6.89 (m, 2H), 3.86 (s, 3H), 3.82 (s, 3H). ^{13}C NMR (150 MHz, $CDCl_3$) δ 160.30, 159.73, 139.54, 136.15, 133.74, 129.93, 124.19, 121.21, 116.33, 115.25, 115.14, 114.21, 110.63, 55.49, 55.43. HRMS (EI) Calculated for $C_{17}H_{15}NO_2S_2$ (M^+) 329.0544, found 329.0547.

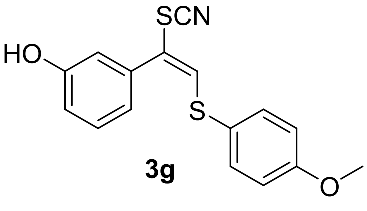

3g

(*E*)-3-(2-((4-methoxyphenyl)thio)-1-thiocyanatovinyl)phenol (3g). Yellow oil (19 mg, 62%). ^{1}H NMR (600 MHz, $CDCl_3$) δ 7.38 (d, *J* = 8.7 Hz, 2H), 7.33 (t, *J* = 7.9 Hz, 1H), 7.17 (d, *J* = 7.7 Hz, 1H), 7.08 (s, 2H), 6.90 (d, *J* = 8.7 Hz, 2H), 6.87 - 6.86 (m, 1H), 5.28 (s, 1H), 3.82 (s, 3H). ^{13}C NMR (150MHz, $CDCl_3$) δ 160.31, 155.86, 139.96, 136.37, 133.76, 130.18, 124.12, 121.35, 116.64, 115.88, 115.62, 115.15, 110.74, 55.49. HRMS (EI) Calculated for $C_{16}H_{13}NO_2S_2$ (M^+) 315.0388, found 315.0379.

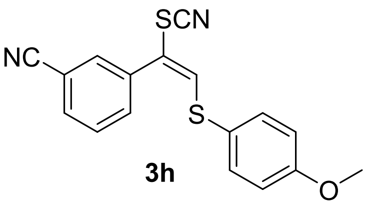

3h

(*E*)-(2-(3-isocyanophenyl)-2-thiocyanatovinyl)(4-methoxyphenyl)sulfane (3h). Yellow oil (16 mg, 51%). ^{1}H NMR (600 MHz, $CDCl_3$) δ 7.93 (s, 1H), 7.87 (dd, *J* = 7.9, 1.0 Hz, 1H), 7.71 (dd, *J* = 7.7, 1.1 Hz, 1H), 7.62 (t, *J* = 7.8 Hz, 1H), 7.41 (d, *J* = 8.3 Hz, 2H), 7.28 (s, 1H), 6.94 (d, *J* = 8.3 Hz, 2H), 3.85 (s, 3H). ^{13}C NMR (150 MHz, $CDCl_3$) δ 160.66, 143.47, 136.44, 134.02, 133.15, 132.63, 132.40, 129.89, 122.96, 118.12, 115.32, 113.62, 113.34, 109.91, 55.52. HRMS (EI) Calculated for $C_{17}H_{12}N_2OS_2$ (M^+) 324.0391, found 324.0393.

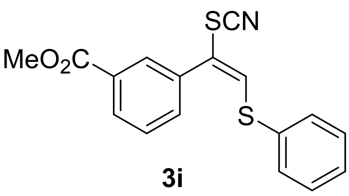

3i

(*E*)-3-(2-(phenylthio)-1-thiocyanatovinyl)phenyl acetate (3i). Yellow oil (19 mg, 60%). ^{1}H NMR (600 MHz, CDCl$_3$) δ 8.27 (t, *J* = 1.5 Hz, 1H), 8.09 (d, *J* = 7.8 Hz, 1H), 7.79 (d, *J* = 7.8 Hz, 1H), 7.57 (t, *J* = 7.8 Hz, 1H), 7.43 (dd, *J* = 8.1, 1.3 Hz, 2H), 7.40 -7.35 (m, 3H), 7.24 (s, 1H), 3.96 (s, 3H). ^{13}C NMR (150 MHz, CDCl$_3$) δ 166.33, 138.53, 135.23, 133.34, 133.22, 131.01, 130.97, 130.58, 130.06, 129.64, 129.13, 128.60, 117.18, 110.10, 52.46. HRMS (EI) Calculated for $C_{17}H_{13}NO_2S_2$ (M^+) 327.0388, found 327.0380.

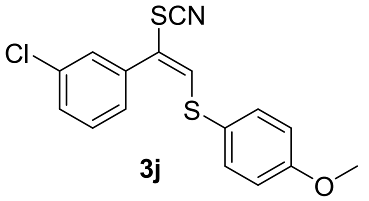

3j

(*E*)-(2-(3-chlorophenyl)-2-thiocyanatovinyl)(4-methoxyphenyl)sulfane (3j). Yellow oil (23 mg, 71%). ^{1}H NMR (400 MHz, CDCl$_3$) δ 7.59 (d, *J* = 1.7 Hz, 1H), 7.42 – 7.36 (m, 1H), 7.43 – 7.34 (m, 4H), 7.14 (s, 1H), 6.90 (d, *J* = 8.8 Hz, 2H), 3.82 (s, 3H). ^{13}C NMR (151 MHz, CDCl$_3$) δ 160.46, 141.61, 136.71, 134.85, 133.89, 130.14, 129.53, 128.84, 127.10, 123.61, 115.22, 114.77, 110.27, 55.50. HRMS (EI) Calculated for $C_{16}H_{12}ClNOS_2$ (M^+) 333.0049, found 333.0041.

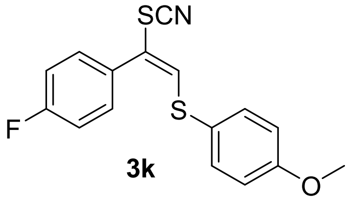

3k

(*E*)-(2-(3-chlorophenyl)-2-thiocyanatovinyl)(4-methoxyphenyl)sulfane (3k). Yellow oil (26 mg, 83%). ^{1}H NMR (400 MHz, CDCl$_3$) δ 7.62 – 7.57 (m, 2H), 7.38 (d, *J* = 8.8 Hz, 2H), 7.19 – 7.13 (m, 2H), 7.07 (s, 1H), 6.91 (dd, *J* = 6.8, 5.0 Hz, 2H), 3.82 (s, 3H). ^{19}F NMR (377 MHz, CDCl$_3$) δ -110.32 – -110.42 (m). ^{13}C NMR (150 MHz, CDCl$_3$) δ 162.89 (d, J = 250.6Hz), 160.38, 139.75, 133.78, 130.96 (d, J = 9.06 Hz), 123.78 , 116.04 (d, J = 22.6Hz), 115.96, 115.58, 115.19, 110.43, 55.49. HRMS (EI) Calculated for $C_{16}H_{12}FNOS_2$ (M^+) 317.0344, found 317.0351.

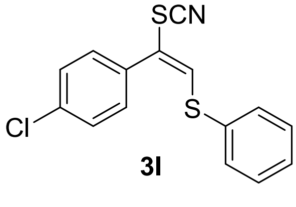

3l

(*E*)-(2-(4-chlorophenyl)-2-thiocyanatovinyl)(phenyl)sulfane (3l). Yellow oil (25 mg, 85%). ^{1}H NMR (600 MHz, CDCl$_3$) δ 7.57 – 7.54 (m, 2H), 7.47 – 7.42 (m, 4H), 7.41 – 7.35 (m, 3H), 7.19 (s,

1H). ^{13}C NMR (151 MHz, $CDCl_3$) δ 137.97, 135.50, 133.36, 133.24, 130.98, 130.27, 129.66, 129.22, 128.61, 117.13, 110.16. HRMS (EI) Calculated for $C_{15}H_{10}ClNS_2$ (M^+) 302.9943, found 302.9941.

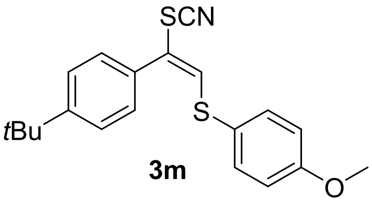

3m

(*E*)-(2-(4-(tert-butyl)phenyl)-2-thiocyanatovinyl)(4-methoxyphenyl)sulfane (3m). Yellow oil (23 mg, 80%). ^{1}H NMR (600 MHz, $CDCl_3$) δ 7.57 (d, *J* = 8.3 Hz, 2H), 7.50 (d, *J* = 8.3 Hz, 2H), 7.41 (d, *J* = 8.5 Hz, 2H), 7.07 (s, 1H), 6.93 (d, *J* = 8.6 Hz, 2H), 3.85 (s, 3H), 1.38 (s, 9H). ^{13}C NMR (150 MHz, $CDCl_3$) δ 160.22, 152.61, 138.27, 133.64, 131.85, 128.56, 125.76 , 124.41, 116.85, 115.11, 110.88, 55.47, 34.88, 31.22. HRMS (EI) Calculated for $C_{20}H_{21}NOS_2$ (M^+) 355.1065, found 355.1060.

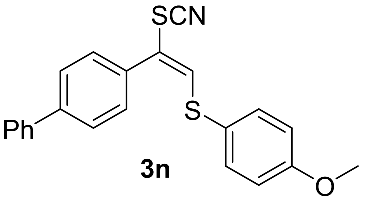

3n

(*E*)-(2-([1,1'-biphenyl]-4-yl)-2-thiocyanatovinyl)(4-methoxyphenyl)sulfane (3n). Yellow oil (26 mg, 70%). ^{1}H NMR (400 MHz, $CDCl_3$) δ 7.69 (s, 4H), 7.63 (d, *J* = 7.4 Hz, 2H), 7.47 (t, *J* = 7.5 Hz, 3H), 7.41 (d, *J* = 8.7 Hz, 2H), 7.12 (s, 1H), 6.91 (d, *J* = 8.7 Hz, 2H), 3.82 (s, 3H). ^{13}C NMR (150MHz, $CDCl_3$) δ 160.33, 142.16, 140.17, 139.43, 133.94, 133.76, 129.31, 128.91, 127.83, 127.48, 127.19, 124.15, 116.41, 115.16, 110.72, 55.49. HRMS (EI) Calculated for $C_{22}H_{17}NOS_2$ (M^+) 375.0752, found 375.0755.

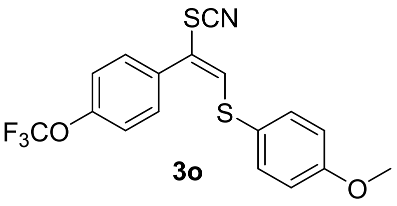

3o

(*E*)-(4-methoxyphenyl)(2-thiocyanato-2-(4-(trifluoromethoxy)phenyl)vinyl)sulfane (3o). Yellow oil (33 mg, 88%). ^{1}H NMR (600 MHz, $CDCl_3$) δ 7.68 (d, *J* = 8.6 Hz, 2H), 7.41 (d, *J* = 8.6 Hz, 2H), 7.33 (d, *J* = 8.5 Hz, 2H), 7.17 (s, 1H), 6.94 (d, *J* = 8.7 Hz, 2H), 3.85 (s, 3H). ^{13}C NMR (150 MHz, $CDCl_3$) δ 160.47, 149.47, 141.15, 133.86, 133.47, 130.57, 123.57, 121.06, 120.40 (q, d = 258 Hz), 117.84, 115.22, 114.93, 110.32, 55.49. ^{19}F NMR (565 MHz, $CDCl_3$) δ -57.65 (s). HRMS (EI)

Calculated for $C_{17}H_{12}F_3NO_2S_2$ (M^+) 383.0262, found 383.0260.

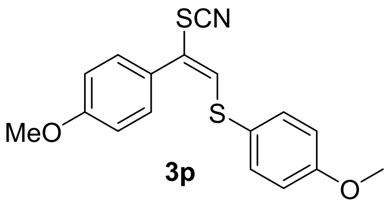

3p

(*E*)-(4-methoxyphenyl)(2-(4-methoxyphenyl)-2-thiocyanatovinyl)sulfane (3p). Yellow oil (23 mg, 77%). ^1H NMR (400 MHz, $CDCl_3$) δ 7.54 (d, *J* = 8.8 Hz, 2H), 7.37 (d, *J* = 8.7 Hz, 2H), 6.99 - 6.97 (m, 3H), 6.90 (d, *J* = 8.7 Hz, 2H), 3.85 (s, 3H), 3.81 (s, 3H). ^{13}C NMR (150 MHz, $CDCl_3$) δ 160.25, 160.19, 137.13, 133.61, 130.42, 127.06, 124.35, 116.95, 115.10, 114.19, 110.80, 55.48, 55.40. HRMS (EI) Calculated for $C_{17}H_{15}NO_2S_2$ (M^+) 329.0544, found 329.0548.

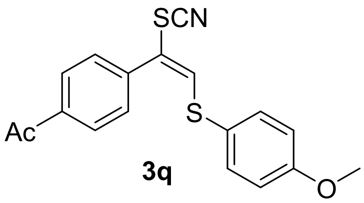

3q

(*E*)-1-(4-(2-((4-methoxyphenyl)thio)-1-thiocyanatovinyl)phenyl)ethan-1-one (3q). Yellow oil (22 mg, 65%). ^1H NMR (400 MHz, $CDCl_3$) δ 8.06 (d, *J* = 8.3 Hz, 2H), 7.73 (d, *J* = 8.3 Hz, 2H), 7.40 (d, *J* = 8.7 Hz, 2H), 7.22 (s, 1H), 6.92 (d, *J* = 8.7 Hz, 2H), 3.83 (s, 3H), 2.65 (s, 3H). ^{13}C NMR (150 MHz, $CDCl_3$) δ 197.23, 160.54, 142.39, 139.49, 137.24, 133.92, 129.11, 128.83, 126.65, 123.53, 115.25, 110.25, 55.51, 26.74. HRMS (EI) Calculated for $C_{18}H_{15}NO_2S_2$ (M^+) 341.0544, found 341.0551.

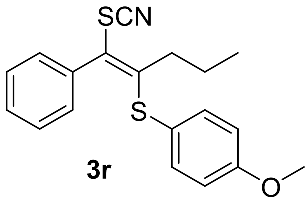

3r

(*E*)-(4-methoxyphenyl)(1-phenyl-1-thiocyanatopent-1-en-2-yl)sulfane (3r). Yellow oil (26 mg, 76%). ^1H NMR (600 MHz, $CDCl_3$) δ 7.47 – 7.37 (m, 5H), 7.29 (d, *J* = 8.7 Hz, 2H), 6.84 (d, *J* = 8.7 Hz, 2H), 3.81 (s, 3H), 2.45 – 2.39 (m, 2H), 1.55 (dd, *J* = 15.1, 7.5 Hz, 2H), 0.87 (t, *J* = 7.4 Hz, 3H). ^{13}C NMR (150 MHz, $CDCl_3$) δ 160.32, 149.06, 137.77, 136.02, 129.68, 128.94, 128.71, 122.29, 117.58, 114.75, 110.30, 55.40, 35.69, 22.13, 13.55. HRMS (EI) Calculated for $C_{19}H_{19}NOS_2$ (M^+) 341.0908, found 341.0901.

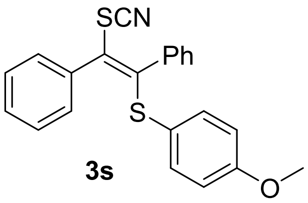

3s

(*E*)-(1,2-diphenyl-2-thiocyanatovinyl)(4-methoxyphenyl)sulfane (3s). Yellow oil (24 mg, 64%). ^{1}H NMR (400 MHz, $CDCl_3$) δ 7.60 – 7.58 (m, 2H), 7.54 – 7.49 (m, 2H), 7.47 – 7.42 (m, 1H), 7.25 – 7.19 (m, 3H), 7.17 – 7.13 (m, 2H), 7.00 (d, *J* = 8.8 Hz, 2H), 6.56 (d, *J* = 8.8 Hz, 2H), 3.67 (s, 3H). ^{13}C NMR (150 MHz, $CDCl_3$) δ 159.84, 145.75, 136.76, 136.75, 136.06, 129.77, 129.37, 129.32, 128.81, 128.70, 128.29, 122.25, 120.41, 114.14, 110.32, 55.24. HRMS (EI) Calculated for $C_{22}H_{17}NOS_2$ (M^+) 375.0752, found 375.0759.

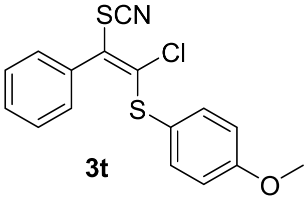

3t

(*Z*)-(1-chloro-2-phenyl-2-thiocyanatovinyl)(4-methoxyphenyl)sulfane (3t). Yellow oil (23 mg, 52%). ^{1}H NMR (600 MHz, $CDCl_3$) δ 7.52 – 7.46 (m, 2H), 7.43 – 7.40 (m, 1H), 7.30 (d, *J* = 8.7 Hz, 1H), 6.88 (d, *J* = 8.7 Hz, 1H), 3.82 (s, 2H). ^{13}C NMR (150 MHz, $CDCl_3$) δ 160.76, 135.49, 134.98, 131.38, 130.07, 129.62, 128.93, 127.64, 121.54, 114.88, 108.75, 55.43. HRMS (EI) Calculated for $C_{16}H_{12}ClNOS_2$ (M^+) 333.0049, found 333.0040.

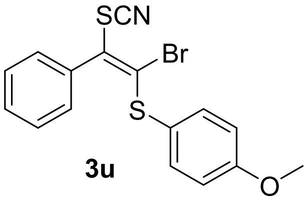

3u

(*Z*)-(1-bromo-2-phenyl-2-thiocyanatovinyl)(4-methoxyphenyl)sulfane (3u). Yellow oil (22 mg, 60%). ^{1}H NMR (600 MHz, $CDCl_3$) δ 7.52 – 7.45 (m, 3H), 7.43 – 7.39 (m, 2H), 7.27 (d, *J* = 8.8 Hz, 2H), 6.88 (d, *J* = 8.6 Hz, 2H), 3.82 (s, 3H). ^{13}C NMR (150 MHz, $CDCl_3$) δ 160.65, 135.61, 134.99, 132.13, 130.04, 129.27, 128.94, 123.20, 121.38, 114.87, 109.02, 55.43. HRMS (EI) Calculated for $C_{16}H_{12}BrNOS_2$ (M^+) 376.9544, found 376.9545.

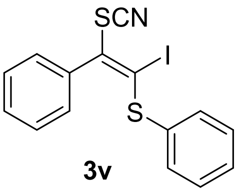

3v

(*Z*)-(1-iodo-2-phenyl-2-thiocyanatovinyl)(phenyl)sulfane (3v). Yellow oil (18 mg, 46%). ^{1}H NMR (600 MHz, $CDCl_3$) δ 7.59 – 7.58 (m, 1H), 7.50 – 7.45 (m, 3H), 7.45 – 7.35 (m, 3H), 7.35 – 7.30 (m, 3H). ^{13}C NMR (150 MHz, $CDCl_3$) δ 142.85 139.63, 132.41, 130.21, 129.65, 129.14, 128.81, 128.52, 128.02, 112.28, 109.34. HRMS (EI) Calculated for $C_{15}H_{10}INS_2$ (M^+) 394.9299, found 394.9233.

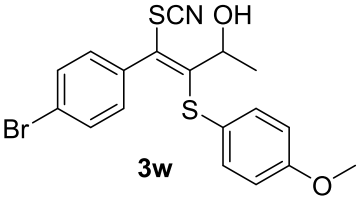

3w

(*E*)-4-(4-bromophenyl)-3-((4-methoxyphenyl)thio)-4-thiocyanatobut-3-en-2-ol (3w). Yellow oil (23 mg, 72%). ^{1}H NMR (600 MHz, $CDCl_3$) δ 7.43 (d, J = 8.4 Hz, 2H), 7.07 (d, J = 8.4 Hz, 2H), 7.00 (d, J = 8.7 Hz, 2H), 6.70 (d, J = 8.7 Hz, 2H), 4.91 (p, J = 6.3 Hz, 1H), 3.77 (s, 3H), 2.22 (d, J = 6.2 Hz, 1H), 1.54 (d, J = 6.5 Hz, 3H). ^{13}C NMR (150 MHz, $CDCl_3$) δ 159.73, 147.73, 136.23, 133.99, 131.67, 131.15, 123.94, 123.53, 123.29, 114.78 109.70, 69.85, 55.44, 21.70. HRMS (EI) Calculated for $C_{18}H_{16}BrNO_2S_2$ (M^+) 420.9806, found 420.9801.

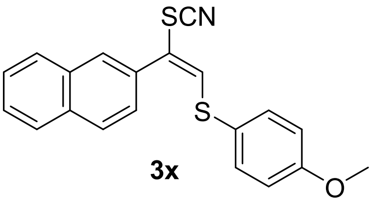

3x

(E)-(4-methoxyphenyl)(2-(naphthalen-2-yl)-2-thiocyanatovinyl)sulfane (3x). Yellow oil (22 mg, 61%). ^{1}H NMR (600 MHz, $CDCl_3$) δ 8.07 (s, 1H), 7.95 – 7.90 (m, 2H), 7.89 – 7.85 (m, 1H), 7.72 – 7.70 (m, 1H), 7.57 – 7.53 (m, 2H), 7.41 (d, J = 8.7 Hz, 2H), 7.17 (s, 1H), 6.91 (d, J = 8.7 Hz, 2H), 3.82 (s, 3H). ^{13}C NMR (150 MHz, $CDCl_3$) δ 160.30, 139.67, 133.73, 133.43, 132.95, 132.26, 129.05, 128.78, 128.47, 127.83, 127.26, 126.81, 125.56, 124.14, 116.76, 115.15, 110.66, 55.48.HRMS (EI) Calculated for $C_{20}H_{15}NOS_2$ (M^+) 349.0595, found 349.0590.

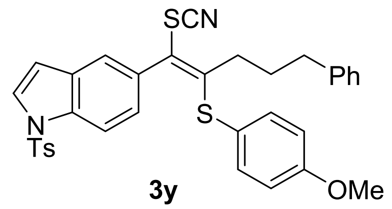

3y

(*E*)-5-(2-((4-methoxyphenyl)thio)-5-phenyl-1-thiocyanatopent-1-en-1-yl)-1-tosyl-1H-indole (3y). Yellow oil (37 mg, 62%). ^{1}H NMR (600 MHz, $CDCl_3$) δ 8.05 (d, *J* = 8.6 Hz, 1H), 7.83 (d, *J* = 8.4 Hz, 2H), 7.62 - 7.51 (m, 1H), 7.55 (d, *J* = 1.4 Hz, 1H), 7.35 - 7.33 (m, 1H), 7.30 - 7.28 (m, 5H), 7.22 (d, *J* = 8.7 Hz, 3H), 7.12 (d, *J* = 7.2 Hz, 2H), 6.77 (d, *J* = 8.7 Hz, 2H), 6.69 (d, *J* = 3.6 Hz, 1H), 3.82 (s, 3H), 2.59 – 2.50 (m, 4H), 2.38 (s, 3H), 1.97 - 1.85 (m, 2H). ^{13}C NMR (150 MHz, $CDCl_3$) δ 160.35, 149.93, 145.25, 141.21, 136.13, 135.30, 134.60, 132.82, 130.72, 130.10, 128.41, 128.37, 127.06, 126.93, 126.01, 122.94, 121.96, 117.10 , 114.72, 113.76, 110.41, 109.08, 100.00, 55.37, 35.34, 33.89 , 30.39, 21.64. HRMS (EI) Calculated for $C_{34}H_{30}N_2O_3S_3$ (M^+) 610.1419, found 610.1411.

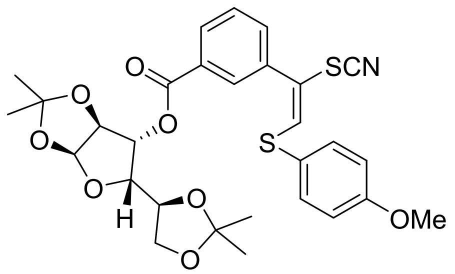

3z

(3a*S*, 5*S*, 6*R*, 6a*S*)-5-((S)-2, 2-dimethyl-1,3-dioxolan-4-yl)-2, 2-dimethyltetrahydrofuro[2, 3-d][1, 3]dioxol-6-yl 3-((E)-2-((4-methoxyphenyl)thio)-1-thiocyanatovinyl)benzoate (3z). Yellow oil (39 mg, 68%). ^{1}H NMR (600 MHz, $CDCl_3$) δ 8.27 (s, 1H), 8.05 (d, *J* = 7.8 Hz, 1H), 7.84 (d, *J* = 7.8 Hz, 1H), 7.58 (t, *J* = 7.8 Hz, 1H), 7.39 (d, *J* = 8.6 Hz, 2H), 7.18 (s, 1H), 6.91 (d, *J* = 8.6 Hz, 2H), 5.97 (d, *J* = 3.5 Hz, 1H), 5.51 (d, *J* = 2.7 Hz, 1H), 4.67 (d, *J* = 3.6 Hz, 1H), 4.43 (dd, *J* = 9.4, 4.1 Hz, 1H), 4.33 (dd, *J* = 8.1, 2.8 Hz, 1H), 4.15 – 4.09 (m, 2H), 3.82 (s, 3H), 1.62 (s, 3H), 1.42 (s, 3H), 1.33 (s, 3H), 1.26 (s, 3H). ^{13}C NMR (150 MHz, $CDCl_3$) δ 164.54, 160.51, 141.68, 135.60, 133.92, 133.67, 130.44, 130.18, 129.30, 123.43, 115.25, 112.44, 110.20, 109.51, 105.18, 83.33, 80.00, 72.56 , 67.38, 55.50, 26.87, 26.74, 26.22, 25.20. HRMS (EI) Calculated for $C_{29}H_{31}NO_8S_2$ (M^+) 585.1491, found 585.1498.

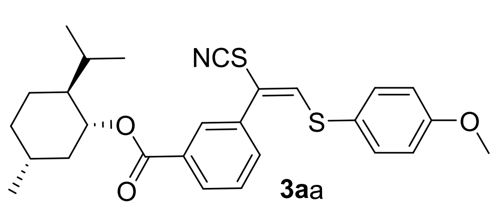

3aa

(*1R*, *2S*, *5R*)-2-isopropyl-5-methylcyclohexyl 3-((*E*)-2-((4-methoxyphenyl)thio)-1-thiocyanatovinyl) benzoate (3aa). Yellow oil (35 mg, 74%). ^{1}H NMR (600 MHz, $CDCl_3$) δ 8.31 (t, J = 1.6 Hz, 1H), 8.09 (d, J = 7.8 Hz, 1H), 7.84 – 7.80 (m, 1H), 7.58 (t, J = 7.8 Hz, 1H), 7.41 (d, J = 8.8 Hz, 2H), 7.19 (s, 1H), 6.93 (d, J = 8.8 Hz, 2H), 4.98 (td, J = 10.9, 4.4 Hz, 1H), 3.85 (s, 3H), 2.21 – 2.16 (m, 1H), 2.04 – 2.02 (m, 1H), 1.78 – 1.75 (m, 2H), 1.62 – 1.59 (m, 2H), 1.21 – 1.12 (m, 2H), 0.98 – 0.94 (m, 7H), 0.84 (d, J = 6.9 Hz, 3H). ^{13}C NMR (150 MHz, $CDCl_3$) δ 165.34, 160.41, 141.04, 135.25, 133.85, 132.93, 131.57, 130.36, 130.04, 128.98, 123.77, 115.50, 115.19, 110.36, 75.36, 55.49, 47.26, 40.92, 34.31, 31.48, 26.52, 23.63, 22.08, 20.84, 16.57. HRMS (EI) Calculated for $C_{27}H_{31}NO_3S_2$ (M^+) 481.1745, found 481.1740.

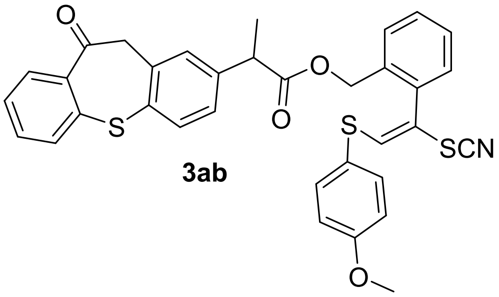

3ab

(*E*)-2-(2-((4-methoxyphenyl)thio)-1-thiocyanatovinyl)benzyl 2-(10-oxo-10, 11-dihydrodibenzo[b, f]thiepin-2-yl)propanoate (3ab). Yellow oil (36 mg, 60%). ^{1}H NMR (400 MHz, $CDCl_3$) δ 8.19 (dd, J = 8.0, 1.5 Hz, 1H), 7.62 – 7.54 (m, 2H), 7.46 – 7.27 (m, 9H), 7.15 (dd, J = 8.0, 1.9 Hz, 1H), 6.99 (s, 1H), 6.87 (d, J = 8.8 Hz, 2H), 5.30 – 5.12 (m, 2H), 4.32 (s, 2H), 3.81 (s, 3H), 1.50 (d, J = 7.2 Hz, 3H). ^{13}C NMR (150 MHz, $CDCl_3$) δ 191.32, 173.68, 160.26, 142.47, 140.18, 137.95, 136.13, 133.95, 133.65, 133.36, 132.55, 131.55, 130.89, 130.10, 129.75, 129.28, 128.71, 126.88, 126.52, 123.15, 115.15, 109.89, 64.30, 55.48, 51.03, 45.17, 18.49. HRMS (EI) Calculated for $C_{34}H_{27}NO_4S_3$ (M^+) 609.1102, found 609.1106.

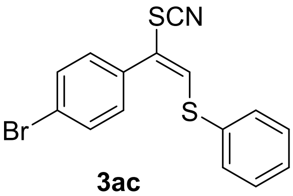

3ac

(*E*)-(2-(4-bromophenyl)-2-thiocyanatovinyl)(phenyl)sulfane (3ac). Yellow oil (28 mg, 81%). ^{1}H NMR (400 MHz, $CDCl_3$) δ 7.61 (d, J = 8.4 Hz, 2H), 7.48 (d, J = 8.4 Hz, 2H), 7.43 (d, J = 7.3 Hz, 2H), 7.38 (d, J = 7.3 Hz, 2H), 7.19 (s, 1H). ^{13}C NMR (150 MHz, $CDCl_3$) δ 138.08, 133.72, 133.34, 132.17, 130.99, 130.48, 129.66, 128.63, 123.80, 117.13, 110.13. HRMS (EI) Calculated for $C_{15}H_{10}BrNS_2$ (M^+) 346.9438, found 346.9434.

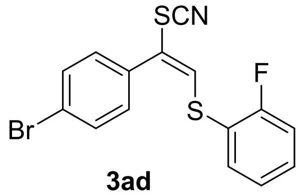

3ad

(*E*)-2-(2-((4-methoxyphenyl)thio)-1-thiocyanatovinyl)benzyl 2-(10-oxo-10, 11-dihydrodibenzo[b, f]thiepin-2-yl)propanoate (3ad). Yellow oil (32 mg, 88%). ^{1}H NMR (600 MHz, CDCl$_3$) δ 7.64 (d, *J* = 8.6 Hz, 2H), 7.51 (d, *J* = 8.6 Hz, 2H), 7.48–7.45 (m, 1H), 7.43 – 7.38 (m, 1H), 7.22 – 7.16 (m, 2H), 7.09 (s, 1H). ^{13}C NMR (150 MHz, CDCl$_3$) δ 161.21 (d, *J* =249.1 Hz), 138.18 135.88, 133.55, 133.38, 132.26, 131.16 (d, *J* = 8.0 Hz), 130.54, 125.16 (d, *J* = 3.9 Hz), 124.01, 118.44, 116.52 (d, *J* = 21.1 Hz), 109.89.HRMS (EI) Calculated for $C_{15}H_9BrFNS_2$ (M^+) 364.9344, found 364.9346.

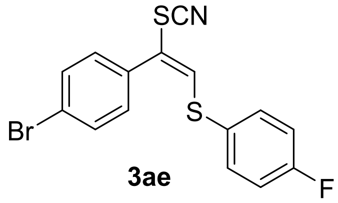

3ae

(*E*)-(2-(4-bromophenyl)-2-thiocyanatovinyl)(4-fluorophenyl)sulfane (3ae). Yellow oil (33 mg, 90%). ^{1}H NMR (600 MHz, CDCl$_3$) δ 7.65 – 7.62 (m, 1H), 7.51 – 7.48 (m, 1H), 7.47 – 7.43 (m, 1H), 7.14 – 7.09 (m, 2H). ^{13}C NMR (150 MHz, CDCl$_3$) δ 163.09 (d, *J* = 250.6 Hz), 138.19, 133.65 (d, *J* = 9.06 Hz), 133.57, 132.21, 130.44 , 128.42 (d, *J* = 3.5 Hz), 123.90 , 117.15, 116.97 ((d, *J* = 22.6 Hz),), 110.04. HRMS (EI) Calculated for $C_{15}H_9BrFNS_2$ (M^+) 364.9344, found 364.9343.

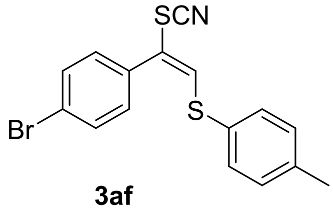

3af

(*E*)-(2-(4-bromophenyl)-2-thiocyanatovinyl)(p-tolyl)sulfane (3af). Yellow oil (28 mg, 79%). ^{1}H NMR (600 MHz, CDCl$_3$) δ 7.63 (d, *J* = 8.6 Hz, 2H), 7.51 (d, *J* = 8.6 Hz, 2H), 7.35 (d, *J* = 8.1 Hz, 2H), 7.22 (d, *J* = 7.9 Hz, 2H), 7.19 (s, 1H), 2.39 (s, 3H). ^{13}C NMR (150 MHz, CDCl$_3$) δ 139.39, 139.07, 133.83, 132.13, 131.41, 130.46, 130.40, 129.74, 123.66, 116.17, 110.26, 21.20. HRMS (EI) Calculated for $C_{16}H_{12}BrNS_2$ 360.9595 (M^+), found 360.9599.

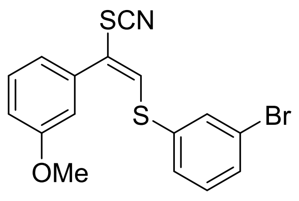

3ag

(E)-(3-bromophenyl)(2-(3-methoxyphenyl)-2-thiocyanatovinyl)sulfane (3ag). Yellow oil (25 mg, 68%). ^{1}H NMR (400 MHz, CDCl$_3$) δ 7.57– 7.56 (m, 1H), 7.48–7.45 (m, 1H), 7.39 (t, *J* = 8.0 Hz, 1H), 7.37–7.33 (m, 1H), 7.27 – 7.22 (m, 1H), 7.16–7.13 (m, 1H), 7.09 –7.08 (m, 2H), 6.98 – 6.95 (m, 1H), 3.86 (s, 3H). ^{13}C NMR (150 MHz, CDCl$_3$) δ 159.78 , 135.92, 135.65, 134.28, 133.08, 131.38, 130.84 , 130.06, 129.05 , 123.23, 121.21, 120.08 , 115.60, 114.26, 110.08, 55.45. HRMS (EI) Calculated for $C_{16}H_{12}BrNOS_2$ (M^+) 376.9544, found 376.9550.

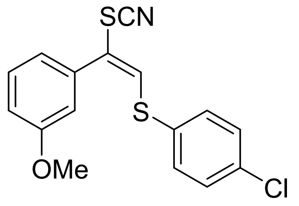

3ah

(*E*)-(4-chlorophenyl)(2-(3-methoxyphenyl)-2-thiocyanatovinyl)sulfane (3ah). Yellow oil (23 mg, 70%). ^{1}H NMR (400 MHz, CDCl$_3$) δ 7.41 – 7.33 (m, 5H), 7.17 – 7.14 (m, 1H), 7.10 – 7.08 (m, 1H), 7.06 (s, 1H), 6.97 – 6.94 (m, 1H), 3.86 (s, 3H). ^{13}C NMR (150 MHz, CDCl$_3$) δ 159.78, 135.72, 135.27, 134.68, 132.29, 132.09, 130.04, 129.75, 121.20, 119.28, 115.50, 114.29, 110.20, 55.44. HRMS (EI) Calculated for $C_{16}H_{12}ClNOS_2$ (M^+) 333.0049, found 333.0052.

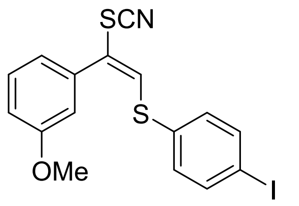

3ai

(*E*)-(4-iodophenyl)(2-(3-methoxyphenyl)-2-thiocyanatovinyl)sulfane (3ai). Yellow oil (24 mg, 57%). ^{1}H NMR (600 MHz, CDCl$_3$) δ 7.74 – 7.70 (m, 2H), 7.42 – 7.39 (m, 1H), 7.20 – 7.14 (m, 3H), 7.12 – 7.10 (m, 1H), 7.08 (s, 1H), 6.99 – 6.97 (m, 1H), 3.88 (s, 3H). ^{13}C NMR (150 MHz, CDCl$_3$) δ 159.77, 138.59, 135.69, 134.67, 133.84, 132.22, 130.05, 121.20, 119.63, 115.53, 114.29, 110.15, 93.92, 55.45. HRMS (EI) Calculated for $C_{16}H_{12}INOS_2$ (M^+) 424.9405, found 424.9401.

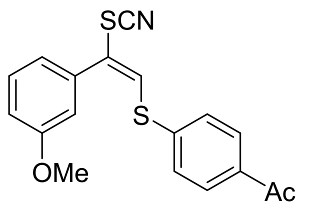

3aj

(*E*)-1-(4-((2-(3-methoxyphenyl)-2-thiocyanatovinyl)thio)phenyl)ethan-1-one (3aj). Yellow oil (17 mg, 50%). ^{1}H NMR (400 MHz, $CDCl_3$) δ 7.94 (d, *J* = 8.5 Hz, 2H), 7.48 – 7.44 (m, 2H), 7.40 (t, *J* = 8.0 Hz, 1H), 7.17 – 7.13 (m, 2H), 7.10 – 7.07 (m, 1H), 7.00 – 6.95 (m, 1H), 3.86 (s, 3H), 2.60 (s, 3H). ^{13}C NMR (150 MHz, $CDCl_3$) δ 196.99, 159.81, 140.33, 135.45, 131.71, 130.11, 129.30, 128.96, 126.09, 121.76, 121.23, 115.69, 114.36, 109.92, 55.46, 26.63. HRMS (EI) Calculated for $C_{18}H_{15}NO_2S_2$ (M^+) 341.0544, found 341.0549.

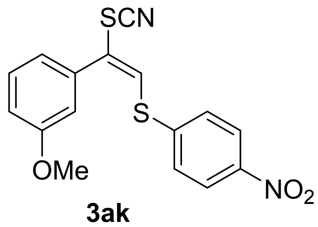

3ak

(*E*)-(2-(3-methoxyphenyl)-2-thiocyanatovinyl)(4-nitrophenyl)sulfane (3ak). Yellow oil (36 mg, 42%). ^{1}H NMR (600 MHz, $CDCl_3$) δ 8.24 (d, *J* = 8.8 Hz, 2H), 7.51 (d, *J* = 8.8 Hz, 2H), 7.42 (t, *J* = 8.0 Hz, 1H), 7.16 – 7.11 (m, 2H), 7.09 – 7.05 (m, 1H), 7.02 – 7.01 (m, 1H), 3.88 (s, 3H). ^{13}C NMR (150 MHz, $CDCl_3$) δ 159.87, 146.79, 143.24, 134.97, 130.23, 129.21, 128.66, 128.34, 124.51, 121.20, 115.93, 114.44, 109.51, 55.48. HRMS (EI) Calculated for $C_{34}H_{27}NO_4S_3$ (M^+) 344.0289, found 344.0296.

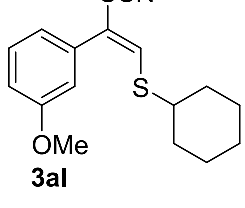

3al

(E)-cyclohexyl(2-(3-methoxyphenyl)-2-thiocyanatovinyl)sulfane (3al). Yellow oil (21 mg, 71%). ^{1}H NMR (400 MHz, $CDCl_3$) δ 7.34 (t, *J* = 8.0 Hz, 1H), 7.16 – 7.14 (m, 1H), 7.10 –7.09 (m, 2H), 6.89 (d, *J* = 8.3 Hz, 1H), 3.84 (s, 3H), 3.01 – 2.92 (m, 1H), 2.08 – 1.98 (m, 2H), 1.80–1.77 (m, 2H), 1.44 – 1.24 (m, 7H). ^{13}C NMR (150 MHz, $CDCl_3$) δ 159.59, 137.49, 136.61, 129.74, 121.20, 115.39, 114.87, 114.20, 110.97, 55.37, 47.80, 33.66, 25.85, 25.35. HRMS (EI) Calculated for $C_{16}H_{19}NOS_2$ (M^+) 305.0908, found 305.0911.

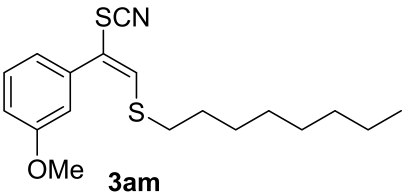

3am

(*E*)-(2-(3-methoxyphenyl)-2-thiocyanatovinyl)(octyl)sulfane (3am). Yellow oil (25 mg, 76%). ^{1}H NMR (400 MHz, $CDCl_3$) δ 7.34 (t, *J* = 8.0 Hz, 1H), 7.15 (d, *J* = 7.7 Hz, 1H), 7.10 (s, 1H), 7.01 (s, 1H), 6.90 (d, *J* = 8.3 Hz, 1H), 3.83 (s, 3H), 2.78 (t, *J* = 7.4 Hz, 2H), 1.71 – 1.61 (m, 2H), 1.41 – 1.34 (m, 2H), 1.27 (s, 8H), 0.88 (t, *J* = 6.5 Hz, 3H). ^{13}C NMR (150 MHz, $CDCl_3$) δ 159.63, 138.99, 136.50, 129.78, 121.17, 115.70, 114.96, 114.15, 110.85, 55.36, 35.55, 31.78, 30.23, 29.13, 29.06, 28.44, 22.64, 14.11. HRMS (EI) Calculated for $C_{18}H_{25}NOS_2$ (M^+) 335.1378, found 335.1374.

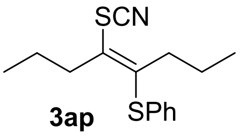

3ap

(*E*)-phenyl(5-thiocyanatooct-4-en-4-yl)sulfane (3ap). Yellow oil (19 mg, 71%). ^{1}H NMR (400 MHz, $CDCl_3$) δ 7.53 – 7.11 (m, 5H), 2.93 – 2.83 (m, 2H), 2.37 – 2.28 (m, 2H), 1.76 – 1.64 (m, 2H), 1.54 – 1.43 (m, 2H), 1.00 (t, *J* = 7.3 Hz, 3H), 0.81 (t, *J* = 7.3 Hz, 3H). ^{13}C NMR (150 MHz, $CDCl_3$) δ 142.10, 133.06, 131.63, 129.27, 127.77, 126.25, 110.28, 37.24, 36.14, 21.74, 21.75, 13.43, 13.39. HRMS (EI) Calculated for $C_{15}H_{19}NS_2$ (M^+) 277.0959, found 277.0964.

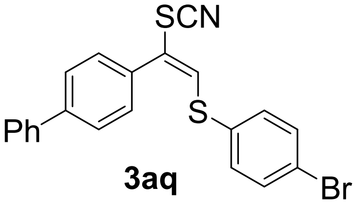

3aq

(*E*)-(2-([1,1'-biphenyl]-4-yl)-2-thiocyanatovinyl)(4-bromophenyl)sulfane (3aq). Yellow oil (1.57 g, 62%). ^{1}H NMR (400 MHz, $CDCl_3$) δ 7.71 – 7.61 (m, 6H), 7.55 – 7.47 (m, 4H), 7.39 (t, *J* = 7.4 Hz, 1H), 7.31 (d, *J* = 8.5 Hz, 2H), 7.10 (s, 1H). ^{13}C NMR (150 MHz, $CDCl_3$) δ 142.58, 140.02, 134.80, 133.28, 132.94, 132.71, 132.22, 129.36, 128.95, 127.94, 127.56, 127.19, 122.69, 119.65, 110.24. HRMS (EI) Calculated for $C_{21}H_{14}BrNS_2$ (M^+) 422.9751, found 422.9757.

5. General procedure for regioselective thiocyanatothiolation of alkenes

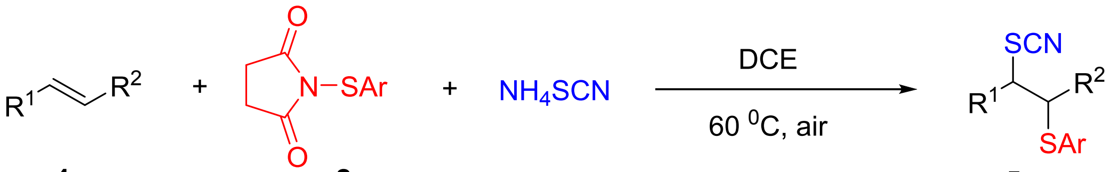

An oven-dried glass vial was charged with *N*-thiosuccinimides (0.12 mmol), NH_4SCN (0.20 mmol) and alkenes (0.10 mmol). Then, DCE (0.5 mL) was added and the mixture was heated to 60℃ and stirred for 12 h under air. After that, water was added to quench the reaction, and the resulting aqueous mixture was extracted with ethyl acetate (3 x 5 mL). The combined organic layers were dried over Na_2SO_4 and concentrated. The crude product was further purified by silica gel column chromatography.

6. Characterization data for the products 5

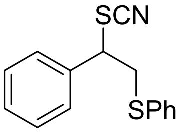

5a

Phenyl(2-phenyl-2-thiocyanatoethyl)sulfane (5a). Yellow oil (16 mg, 61%). ^1H NMR (400 MHz, $CDCl_3$) δ 7.42 – 7.27 (m, 10H), 4.46 –4.43 (m, 1H), 3.69 –3.56 (m, 2H). ^{13}C NMR (100 MHz, $CDCl_3$) δ 136.31, 133.59, 131.18, 129.46, 129.40, 129.22, 127.83, 127.62, 111.25, 52.34, 39.96. HRMS (EI) Calculated for $C_{15}H_{13}NS_2$ (M^+) 271.0489, found 271.0493.

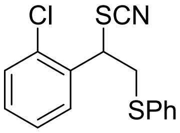

5b

(2-(2-chlorophenyl)-2-thiocyanatoethyl)(phenyl)sulfane (5b). Yellow oil (19 mg, 62%). ^1H NMR (600 MHz, $CDCl_3$) δ 7.45 – 7.40 (m, 4H), 7.36 – 7.30 (m, 5H), 4.96 – 4.90 (m, 1H), 3.73 – 3.64 (m, 2H). ^{13}C NMR (150 MHz, $CDCl_3$) δ 134.22, 133.97, 133.31, 131.71, 130.33, 130.26, 129.39, 128.23, 127.87, 127.62, 110.69, 47.94, 39.32. HRMS (EI) Calculated for $C_{15}H_{12}ClNS_2$ (M^+) 305.0100, found 305.0109.

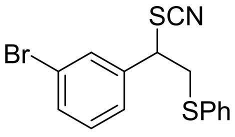

5c

(2-(3-bromophenyl)-2-thiocyanatoethyl)(phenyl)sulfane (5c). Yellow oil (19 mg, 55%). ^{1}H NMR (600 MHz, $CDCl_3$) δ 7.52 - 7.50 (m, 1H), 7.43 (d, *J* = 1.7 Hz, 1H), 7.40 – 7.29 (m, 5H), 7.28 – 7.22 (m, 2H), 4.41–4.38 (m, 1H), 3.65 –3.55 (m, 2H). ^{13}C NMR (150 MHz, $CDCl_3$) δ 138.58, 133.18, 132.58, 131.40, 130.91, 130.70, 129.47, 127.88, 126.50, 123.13, 110.75, 51.62, 39.84. HRMS (EI) Calculated for $C_{15}H_{12}BrNS_2$ (M^+) 348.9595, found 348.9590.

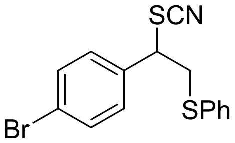

5d

(2-(4-bromophenyl)-2-thiocyanatoethyl)(phenyl)sulfane (5d). Yellow oil (19 mg, 60%). ^{1}H NMR (600 MHz, $CDCl_3$) δ 7.52 (d, *J* = 8.4 Hz, 2H), 7.38 - 7.31 (m, 5H), 7.17 (d, *J* = 8.4 Hz, 2H), 4.42 - 4.40 (m, 1H), 3.67 - 3.54 (m, 2H). ^{13}C NMR (150 MHz, $CDCl_3$) δ 135.36, 133.24, 132.39, 131.37, 129.46, 127.81, 123.59, 110.84, 51.64, 39.81. HRMS (EI) Calculated for $C_{15}H_{12}BrNS_2$ (M^+) 348.9595, found 348.9590.

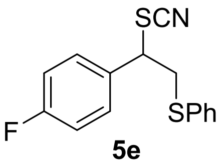

5e

(2-(4-fluorophenyl)-2-thiocyanatoethyl)(phenyl)sulfane (5e). Yellow oil (15 mg, 54%). ^{1}H NMR (400 MHz, $CDCl_3$) δ 7.37 – 7.24 (m, 7H), 7.06 (t, *J* = 8.6 Hz, 2H), 4.45 – 4.41 (m, 1H), 3.67 –3.52 (m, 2H). ^{19}F NMR (377 MHz, $CDCl_3$) δ -111.10 – -111.25 (m). ^{13}C NMR (100 MHz, $CDCl_3$) δ 163.05 (d, *J* = 250.4 Hz), 133.37, 132.16 (d, *J* = 3.4 Hz), 131.30, 129.73 (d, *J* = 8.5 Hz), 129.44, 127.76, 116.39 (d, *J* = 22.2 Hz), 111.01, 51.64, 40.05. HRMS (EI) Calculated for $C_{15}H_{12}FNS_2$ (M^+) 289.0395, found 289.0390.

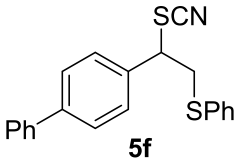

5f

(2-([1,1'-biphenyl]-4-yl)-2-thiocyanatoethyl)(phenyl)sulfane (5f). Yellow oil (17 mg, 51%). ^{1}H NMR (400 MHz, CDCl$_3$) δ 7.57 (d, *J* = 8.0 Hz, 4H), 7.45 (t, *J* = 7.6 Hz, 2H), 7.41 – 7.25 (m, 8H), 4.52 – 4.48 (m, 1H), 3.72 – 3.60 (m, 2H). ^{13}C NMR (150 MHz, CDCl$_3$) δ 142.35, 140.11, 135.14, 133.62, 131.26, 129.40, 128.91, 128.29, 127.87, 127.81, 127.63, 127.15, 111.27, 52.23, 39.99. HRMS (EI) Calculated for $C_{21}H_{17}NS_2$ (M^+) 347.0802, found 347.0809.

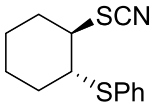

5g

(2-(2-chlorophenyl)-2-thiocyanatoethyl)(phenyl)sulfane (5g). Yellow oil (12 mg, 47%). ^{1}H NMR (400 MHz, CDCl$_3$) δ 7.55 – 7.46 (m, 2H), 7.40 – 7.28 (m, 3H), 3.25– 3.17 (m, 1H), 3.05– 2.99 (m, 1H), 2.52 – 2.39 (m, 1H), 2.28 – 2.15 (m, 1H), 1.84 – 1.70 (m, 3H), 1.58 – 1.24 (m, 3H). ^{13}C NMR (100 MHz, CDCl$_3$) δ 134.18, 131.80, 129.20, 128.45, 111.63, 52.18, 51.47, 33.56, 33.51, 25.08, 25.04. HRMS (EI) Calculated for $C_{13}H_{15}NS_2$ (M^+) 249.0646, found 249.0653.

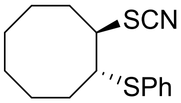

5h

phenyl((1R,2R)-2-thiocyanatocyclooctyl)sulfane (5h). Yellow oil (19 mg, 53%). ^{1}H NMR (400 MHz, CDCl$_3$) δ 7.52 – 7.44 (m, 2H), 7.38 – 7.28 (m, 3H), 3.60 – 3.55 (m, 1H), 3.39 – 3.32 (m, 1H), 2.40 – 2.30 (m, 1H), 2.25 – 2.10 (m, 2H), 1.91 – 1.78 (m, 3H), 1.72 – 1.58 (m, 3H), 1.55 – 1.41 (m, 3H). ^{13}C NMR (100 MHz, CDCl$_3$) δ 133.32, 133.11, 129.25, 128.07, 112.49, 55.13, 53.31, 31.52, 31.28, 25.96, 25.92, 25.64, 25.28. HRMS (EI) Calculated for $C_{15}H_{19}NS_2$ (M^+) 277.0959, found 277.0963.

7. X-ray Crystallographic Analysis of product 3aq

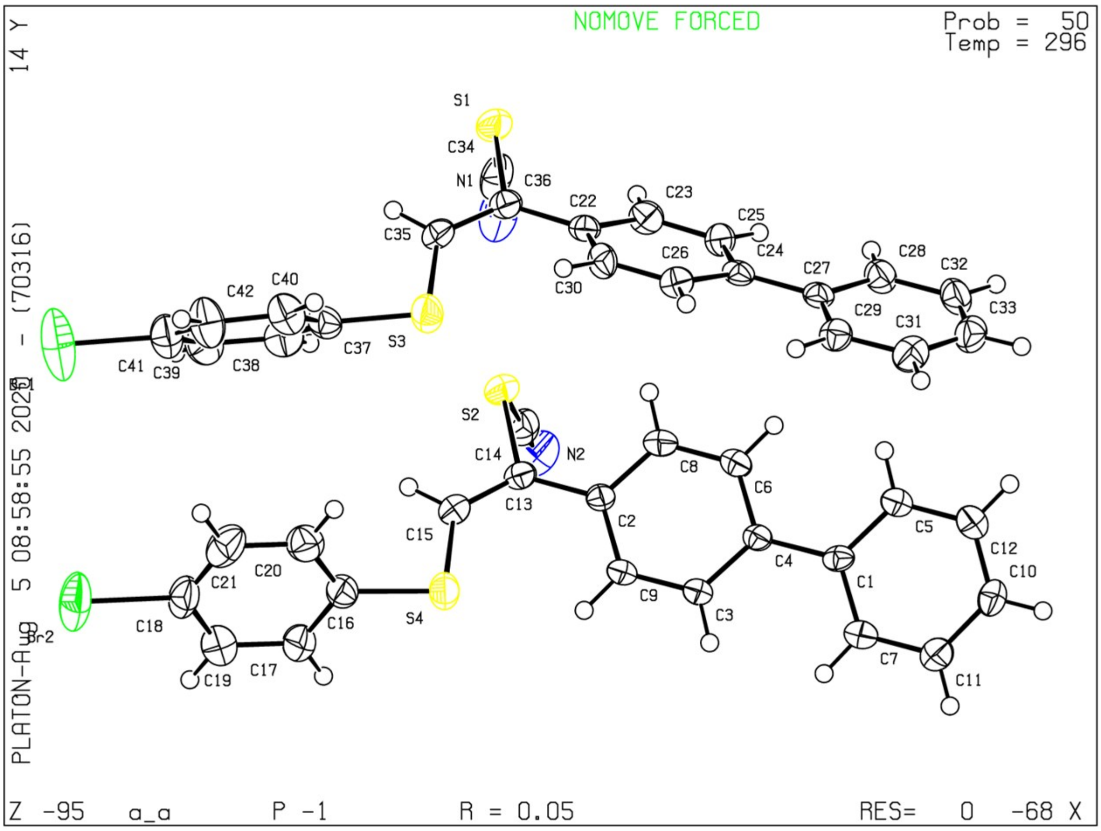

Table 1. Crystal data and structure refinement for A_a.

Identification code	a_a	
Empirical formula	C21 H14 Br N S2	
Formula weight	424.36	
Temperature	296(2) K	
Wavelength	0.71073 Å	
Crystal system	Triclinic	
Space group	P-1	
Unit cell dimensions	a = 9.4365(5) Å	α= 89.393(3)°.
	b = 9.6809(7) Å	β= 88.231(3)°.
	c = 20.5558(11) Å	γ = 74.156(3)°.
Volume	1805.64(19) Å^3	
Z	4	

Density (calculated)	1.561 Mg/m^3
Absorption coefficient	2.511 mm^{-1}
F(000)	856
Crystal size	0.180 x 0.160 x 0.150 mm^3
Theta range for data collection	1.982 to 26.772°.
Index ranges	-10<=h<=11, -9<=k<=12, -26<=l<=24
Reflections collected	15061
Independent reflections	7459 [R(int) = 0.0426]
Completeness to theta = 25.242°	97.8 %
Absorption correction	None
Refinement method	Full-matrix least-squares on F^2
Data / restraints / parameters	7459 / 0 / 451
Goodness-of-fit on F^2	0.984
Final R indices [I>2sigma(I)]	R1 = 0.0537, wR2 = 0.1525
R indices (all data)	R1 = 0.0927, wR2 = 0.1764
Extinction coefficient	n/a
Largest diff. peak and hole	1.253 and -1.001 e.$Å^{-3}$

.

8. Copies of NMR Spectra

1. ^{1}H NMR of **3a** (600 MHz, $CDCl_3$)

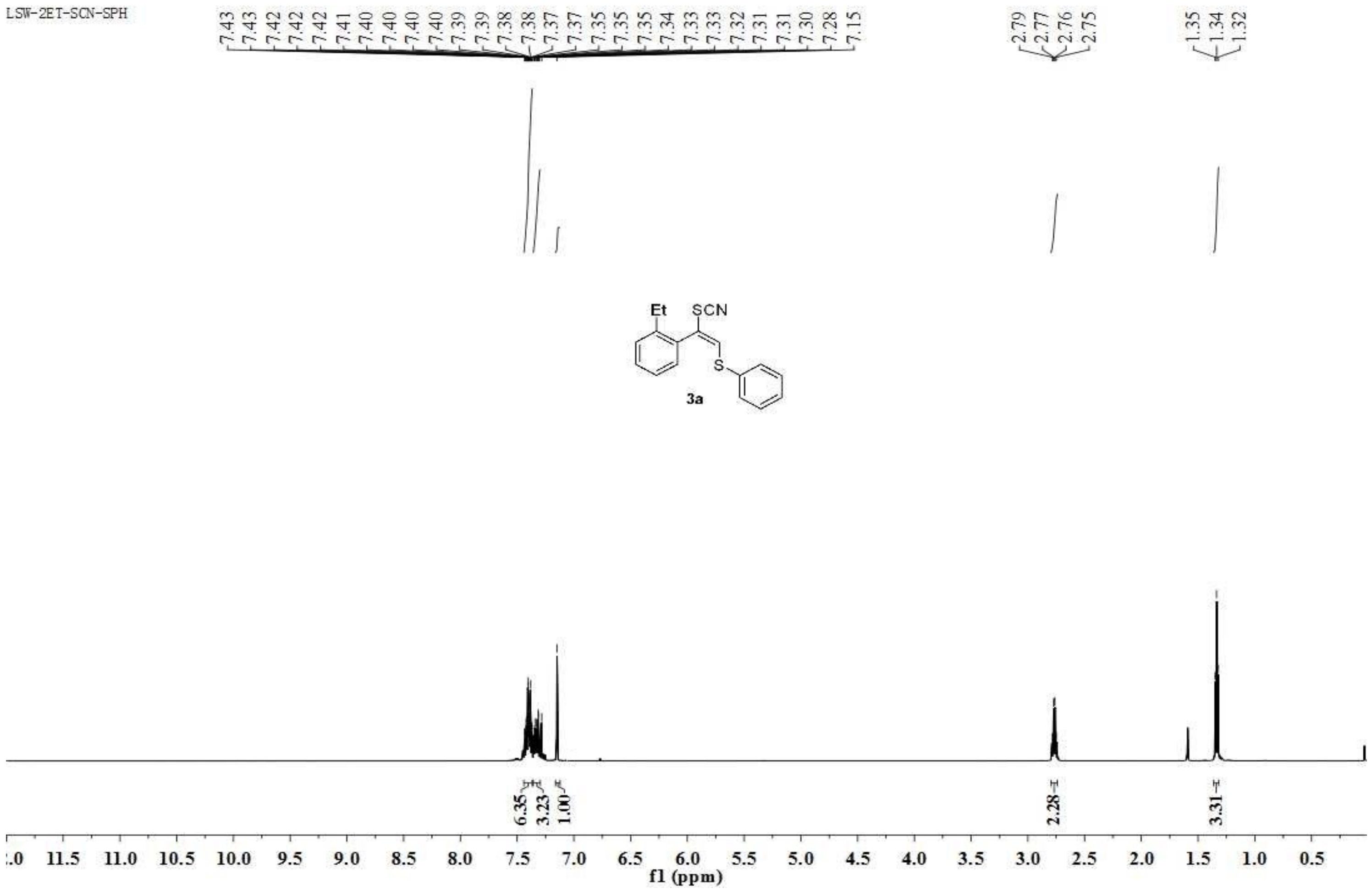

2. ^{13}C NMR of **3a** (125 MHz, $CDCl_3$)

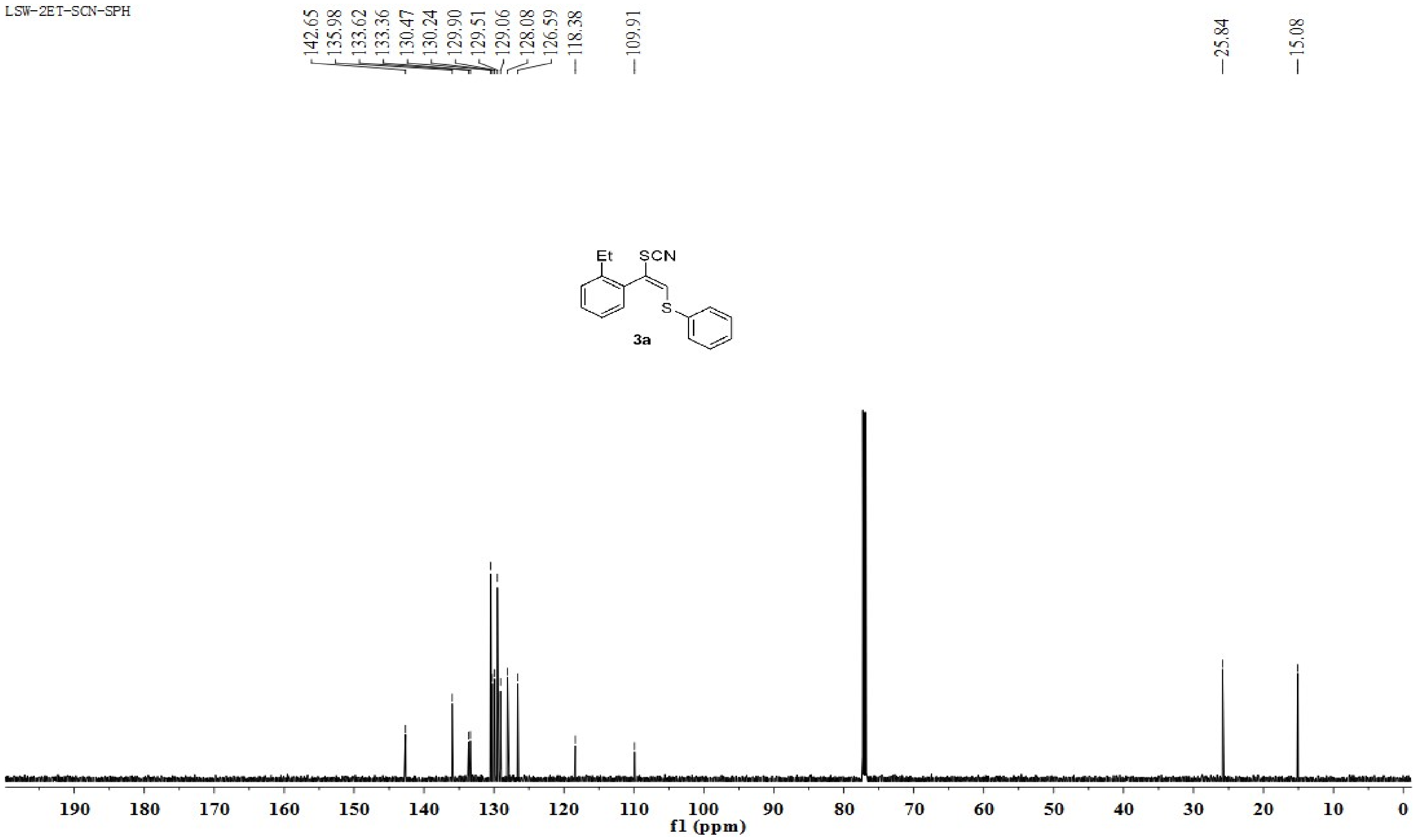

3. ^{1}H NMR of **3b** (600 MHz, $CDCl_3$)

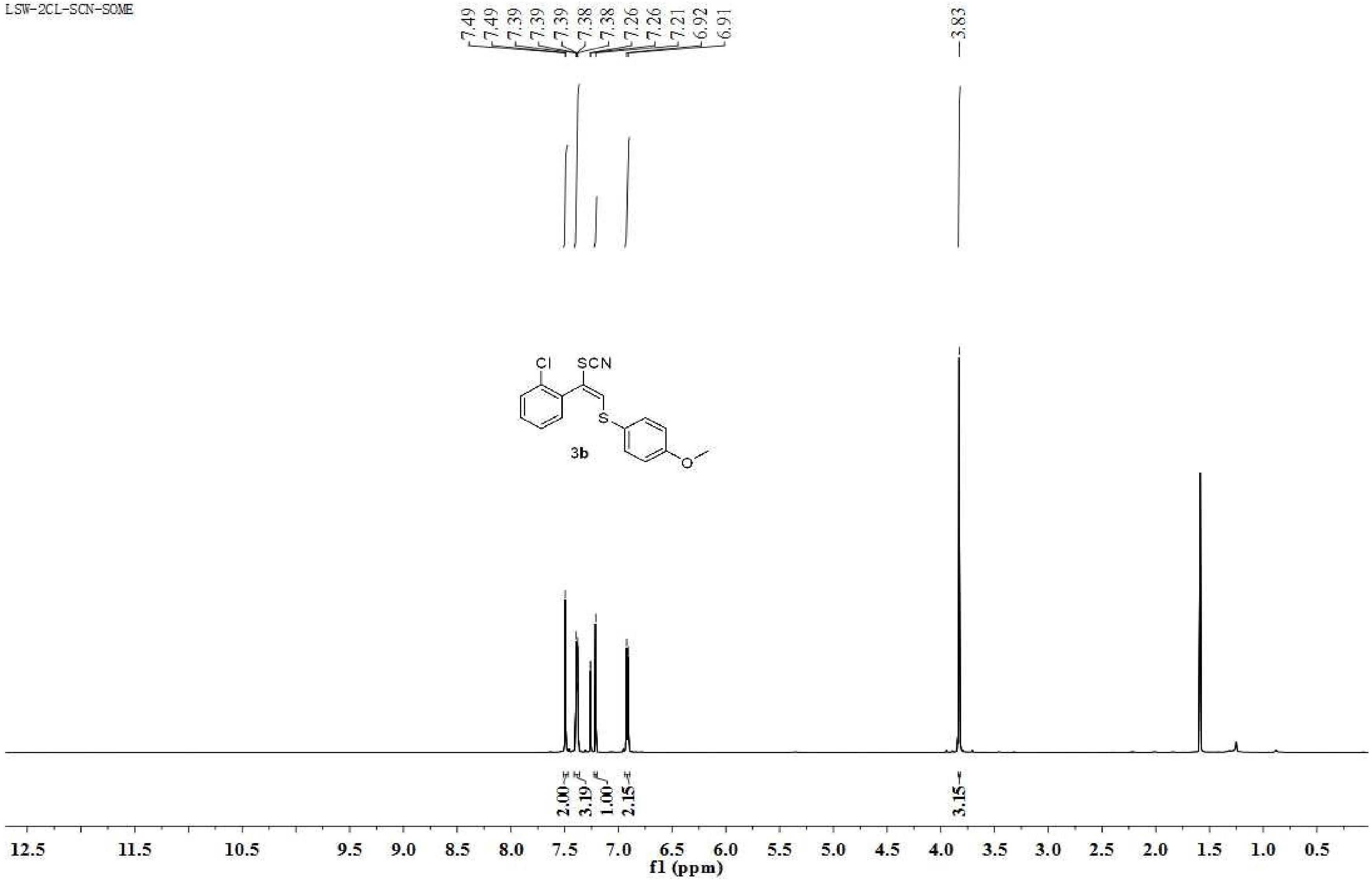

3b

4. ^{13}C NMR of **3b** (125 MHz, $CDCl_3$)

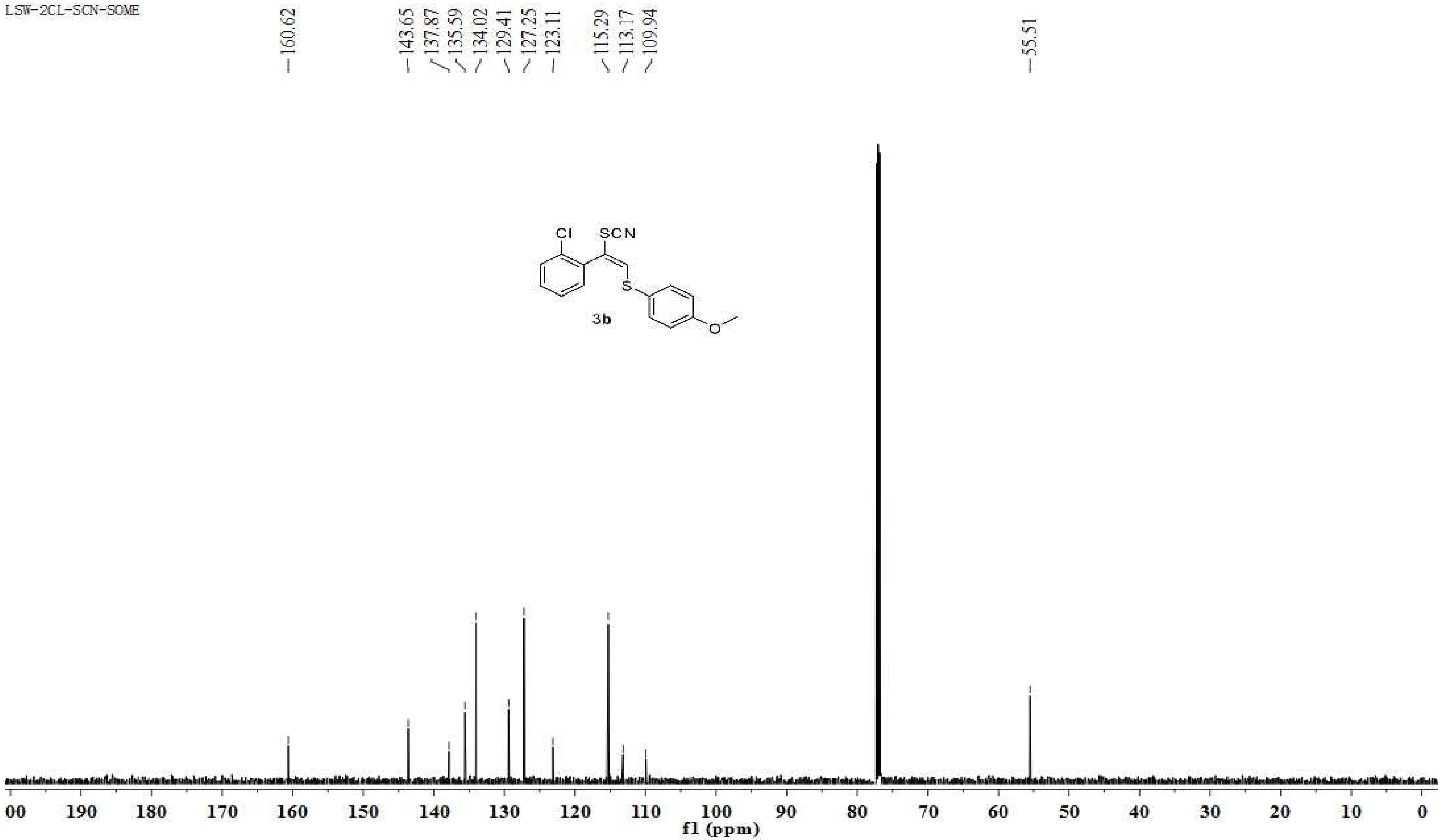

5. ^{1}H NMR of **3c** (600 MHz, $CDCl_3$)

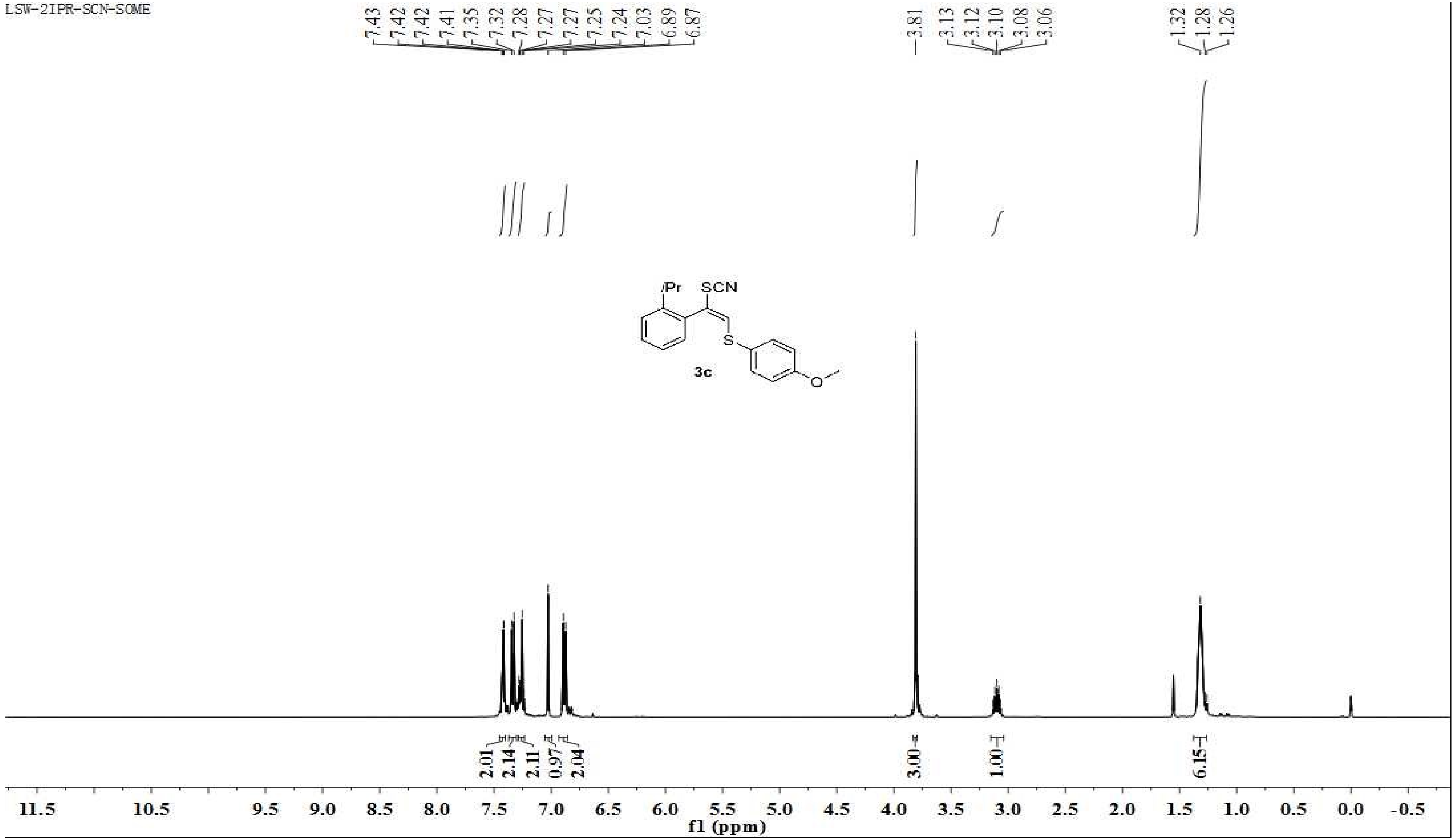

6. ^{13}C NMR of **3c** (125 MHz, $CDCl_3$)

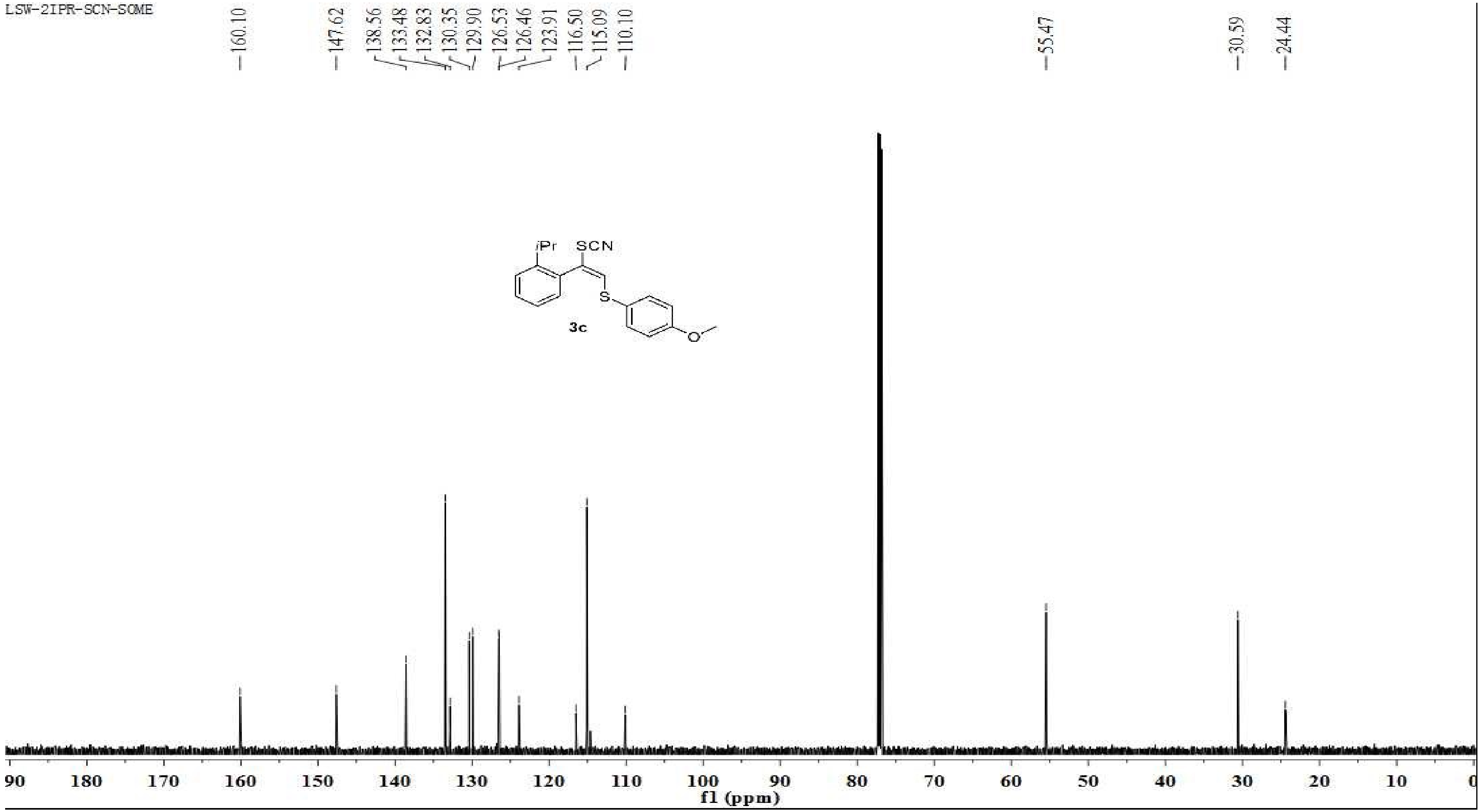

7. ^{1}H NMR of **3d** (600 MHz, $CDCl_3$)

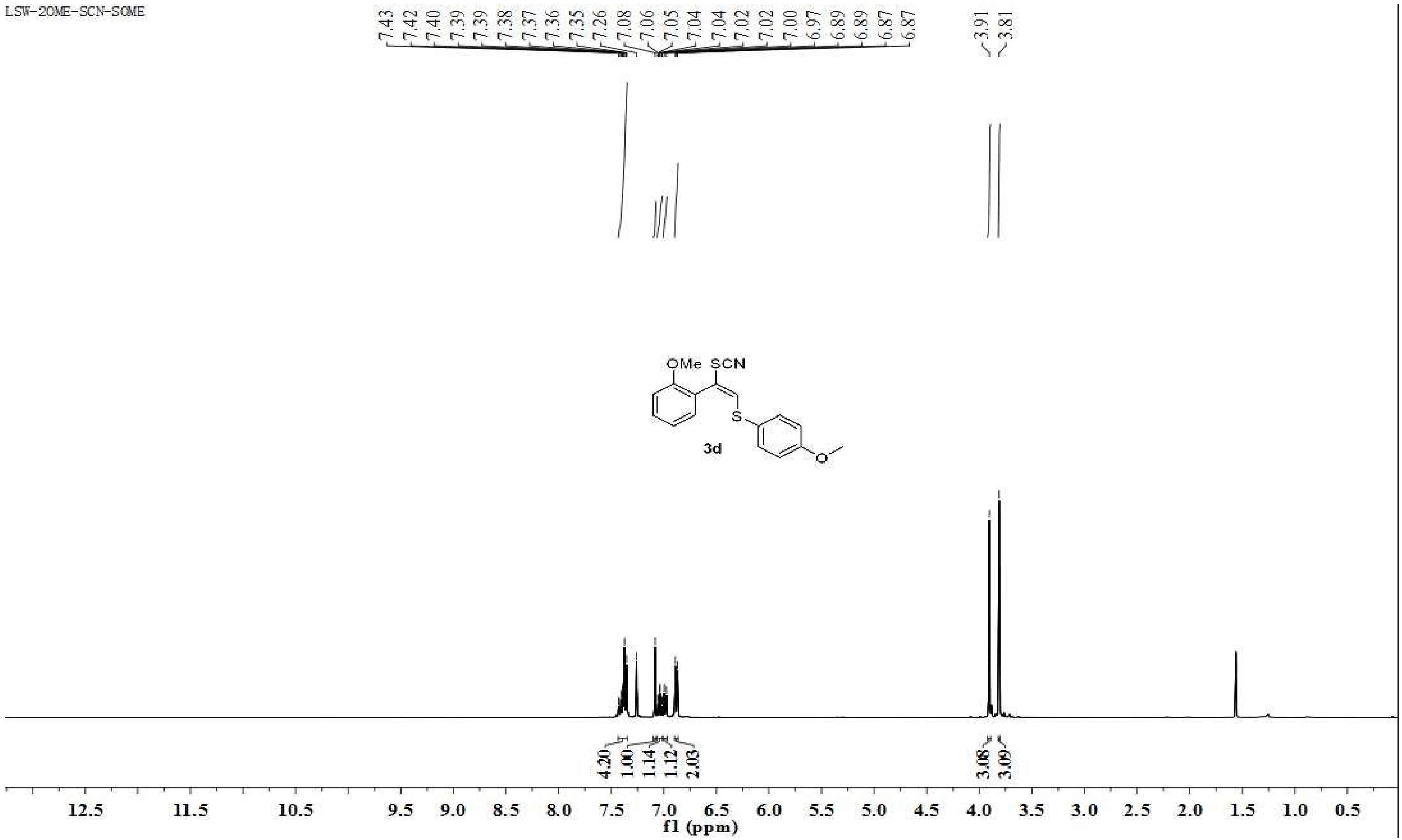

8. ^{13}C NMR of **3d** (125 MHz, $CDCl_3$)

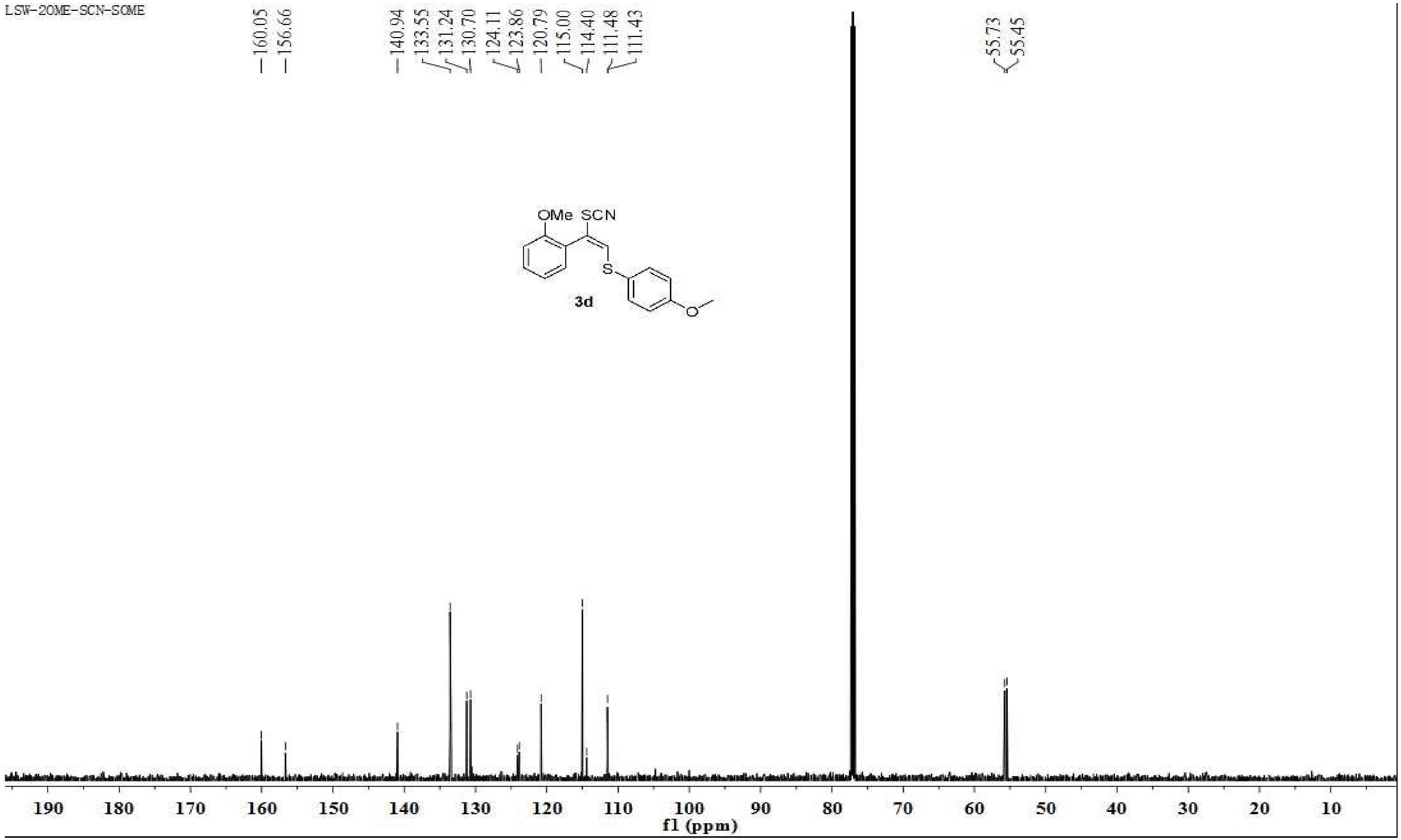

9. ^{1}H NMR of **3e** (600 MHz, $CDCl_3$)

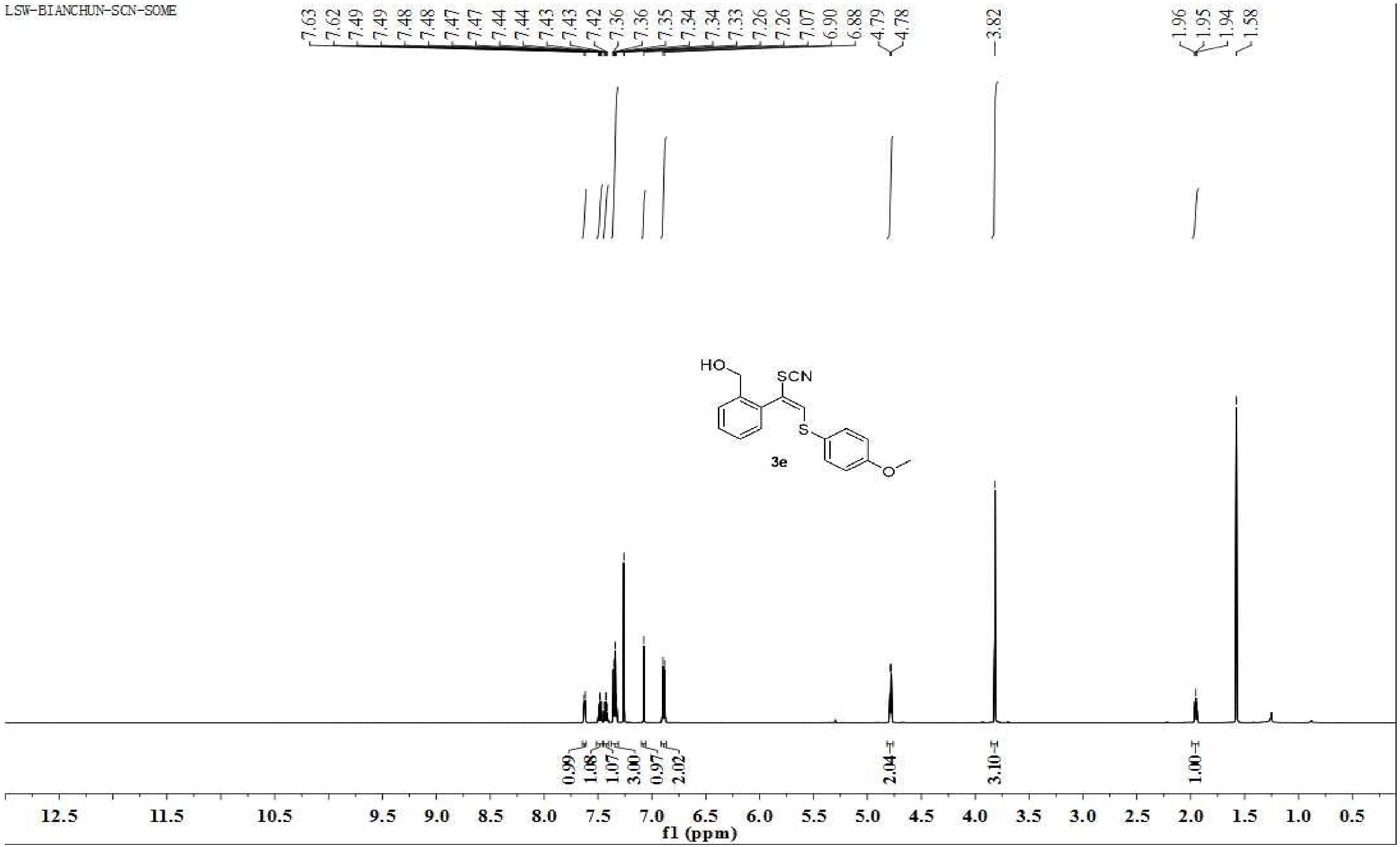

10. ^{13}C NMR of **3e** (125 MHz, $CDCl_3$)

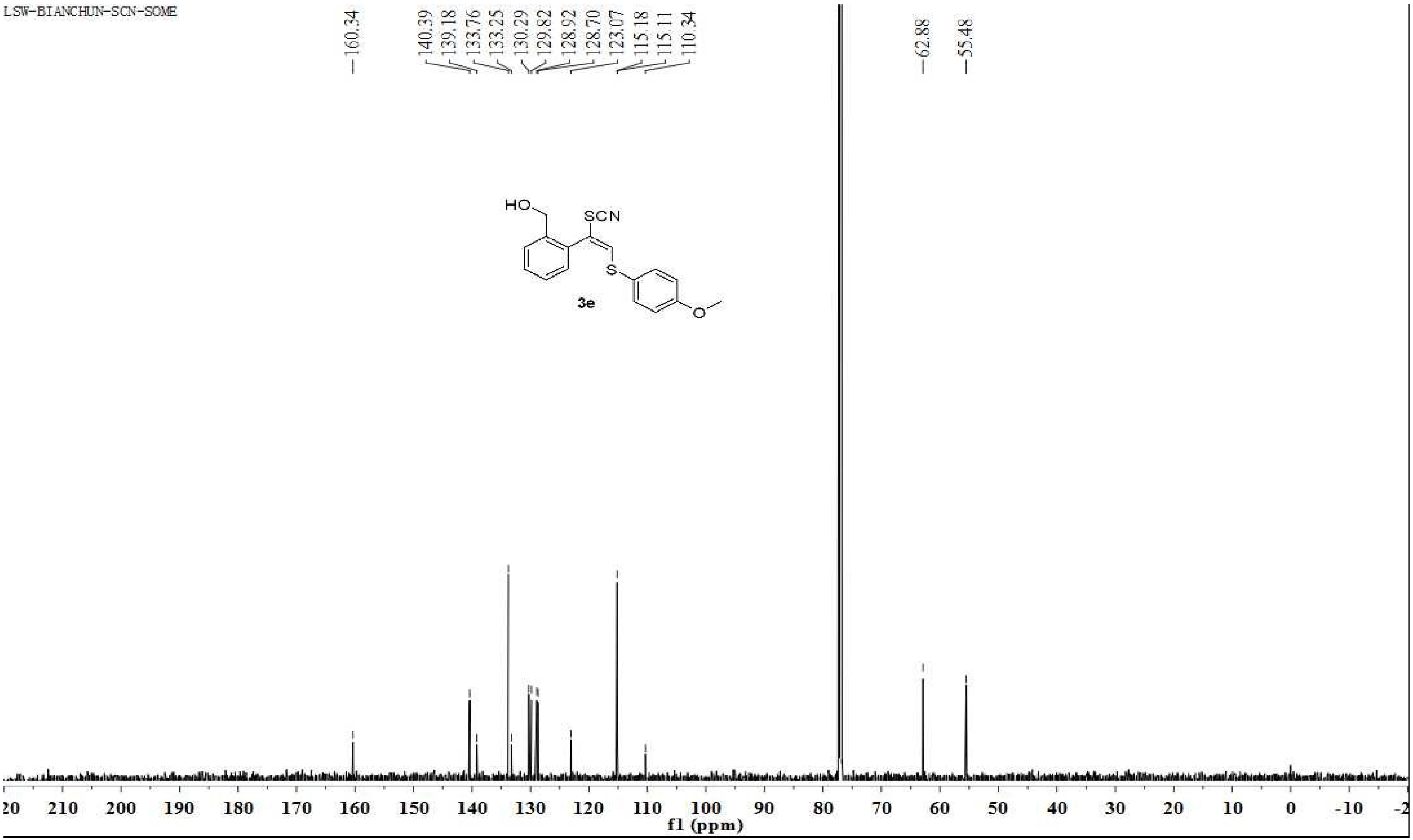

4.

11. ^{1}H NMR of **3f** (600 MHz, $CDCl_3$)

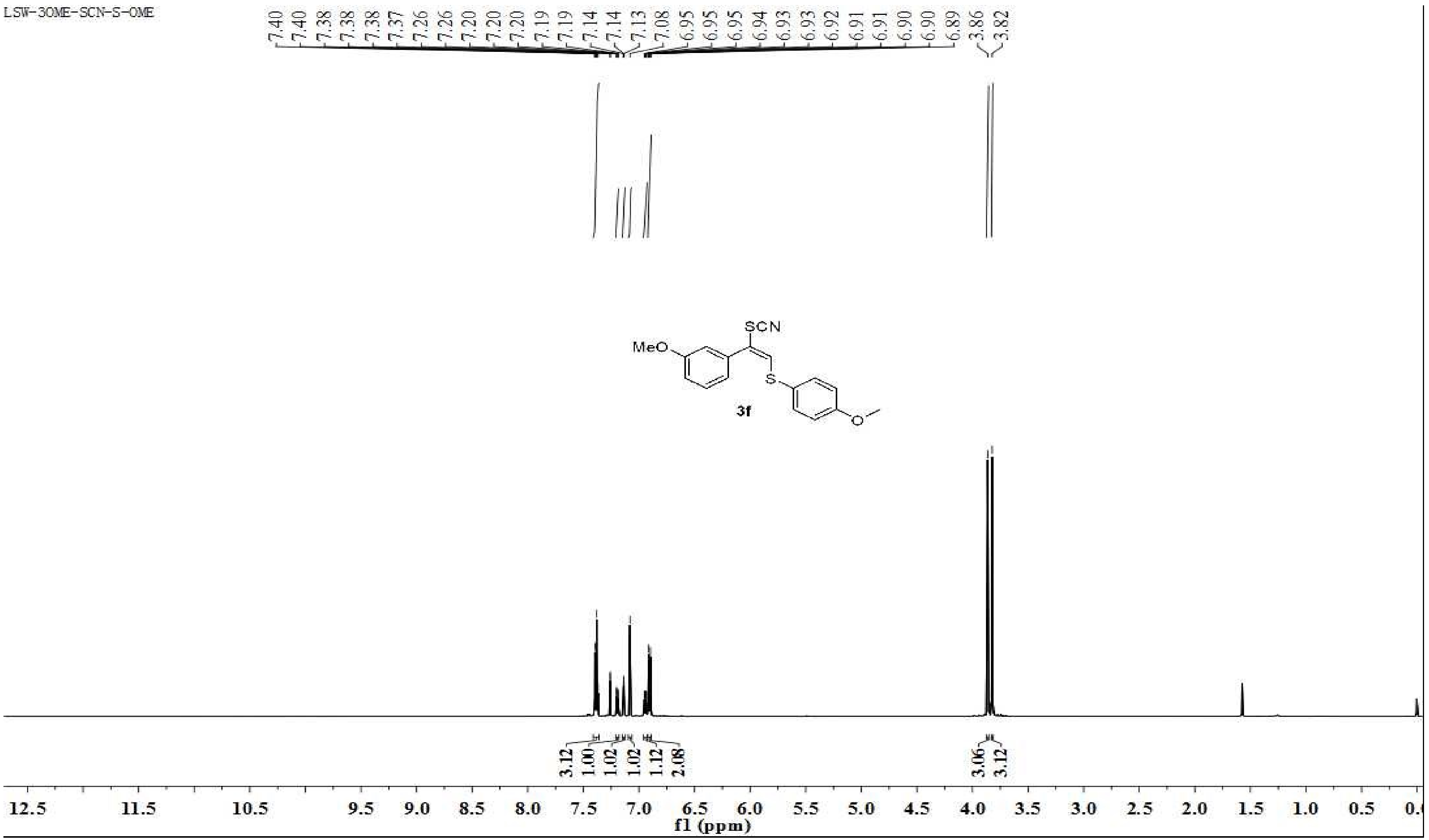

5.

12. ^{13}C NMR of **3a** (125 MHz, $CDCl_3$)

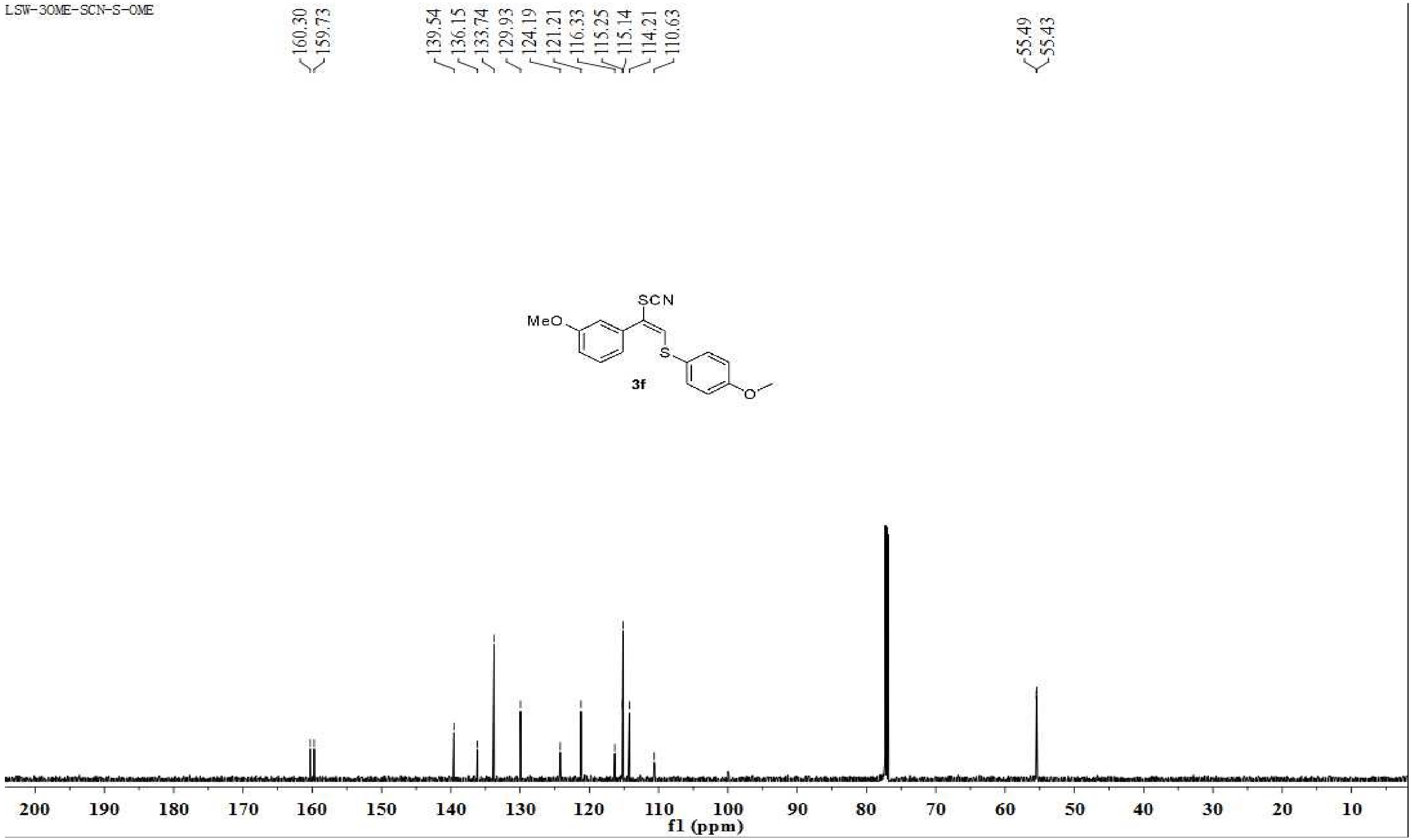

13. ^{1}H NMR of **3g** (600 MHz, $CDCl_3$)

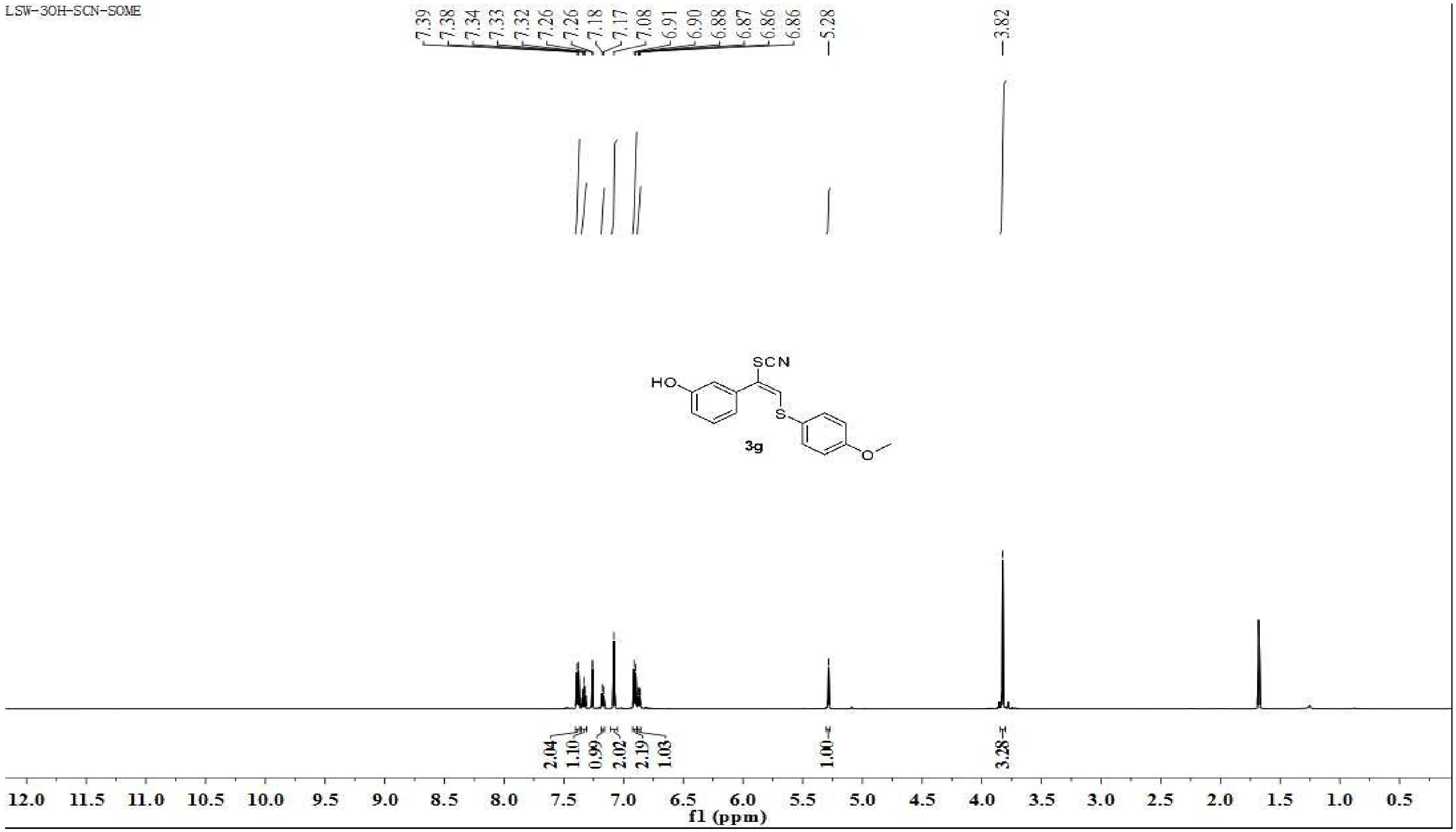

14. ^{13}C NMR of **3g** (125 MHz, $CDCl_3$)

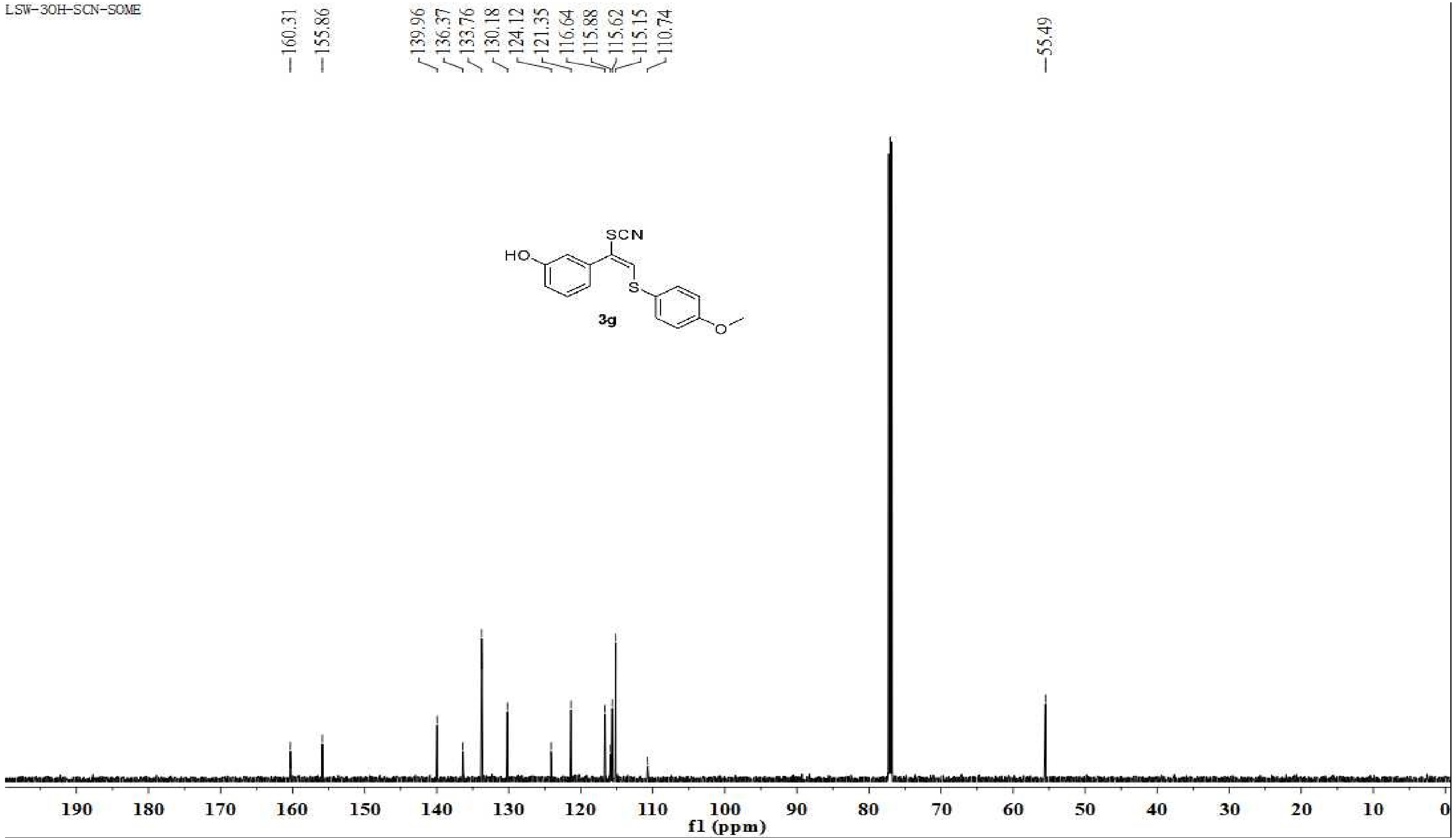

15. ^{1}H NMR of **3h** (600 MHz, $CDCl_3$)

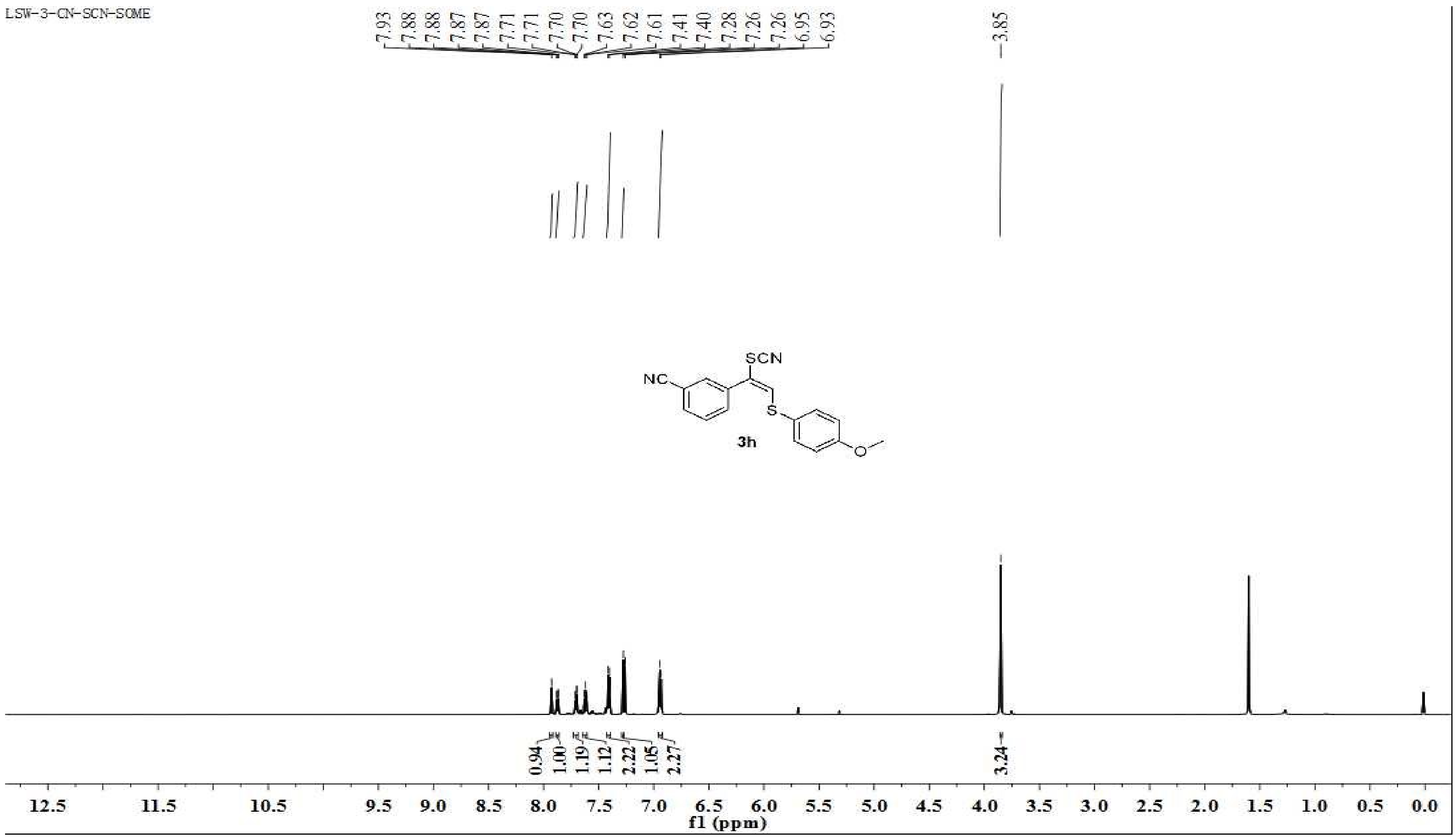

16. ^{13}C NMR of **3a** (125 MHz, $CDCl_3$)

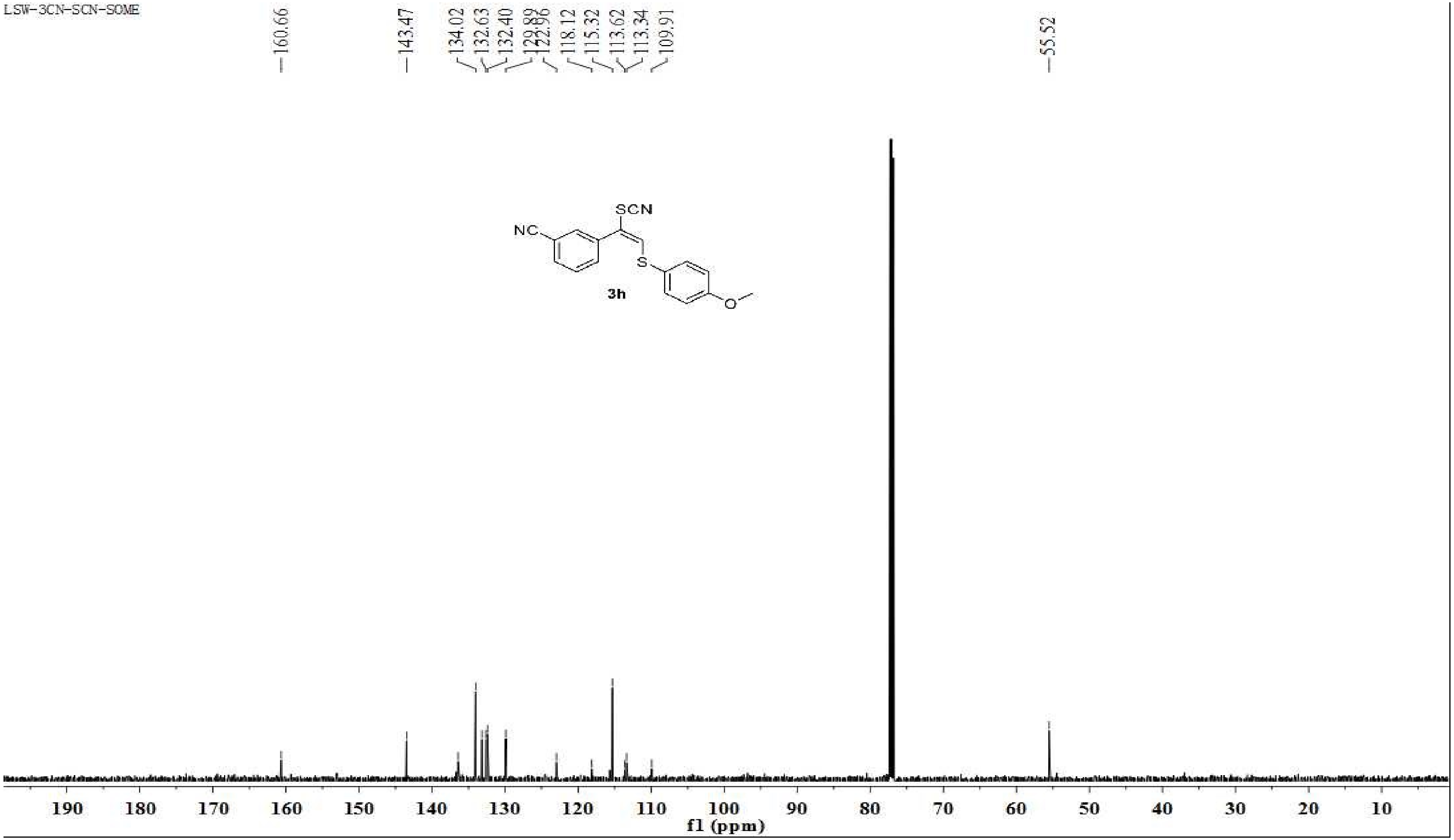

6.

17. ^{1}H NMR of **3i** (600 MHz, $CDCl_3$)

7. 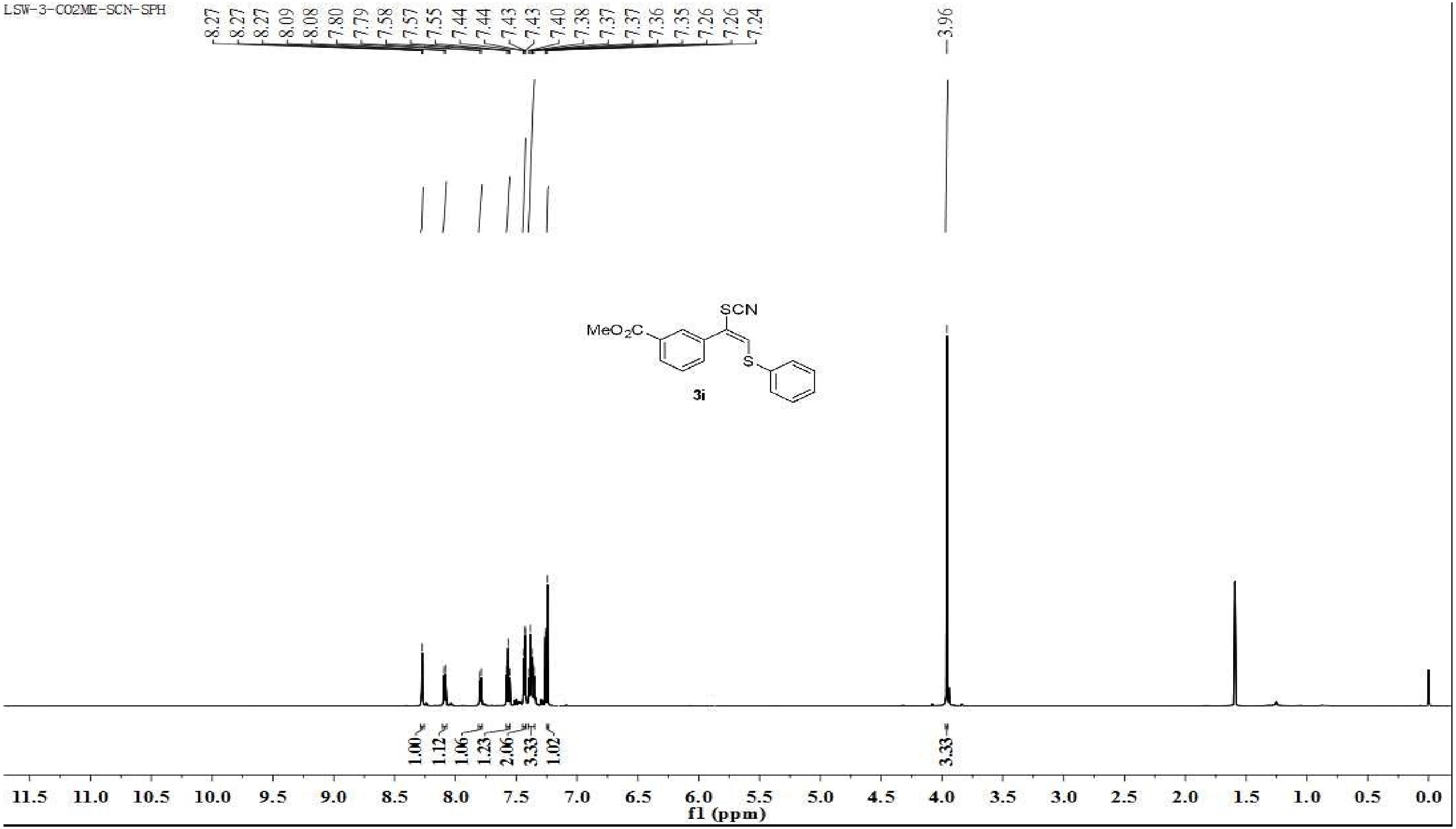

18. ^{13}C NMR of **3i** (125 MHz, $CDCl_3$)

8. 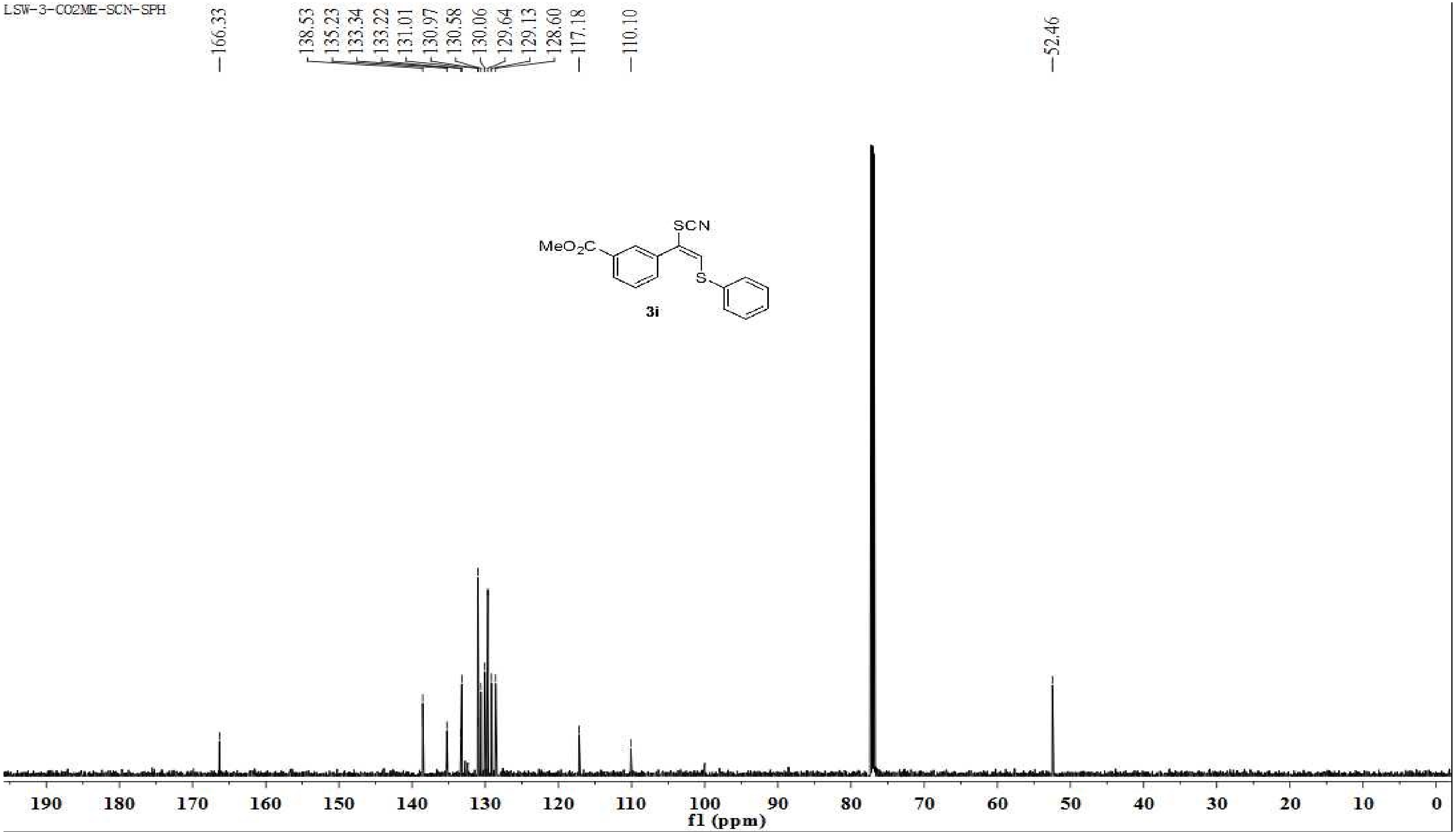

19. ^{1}H NMR of **3j** (600 MHz, $CDCl_3$)

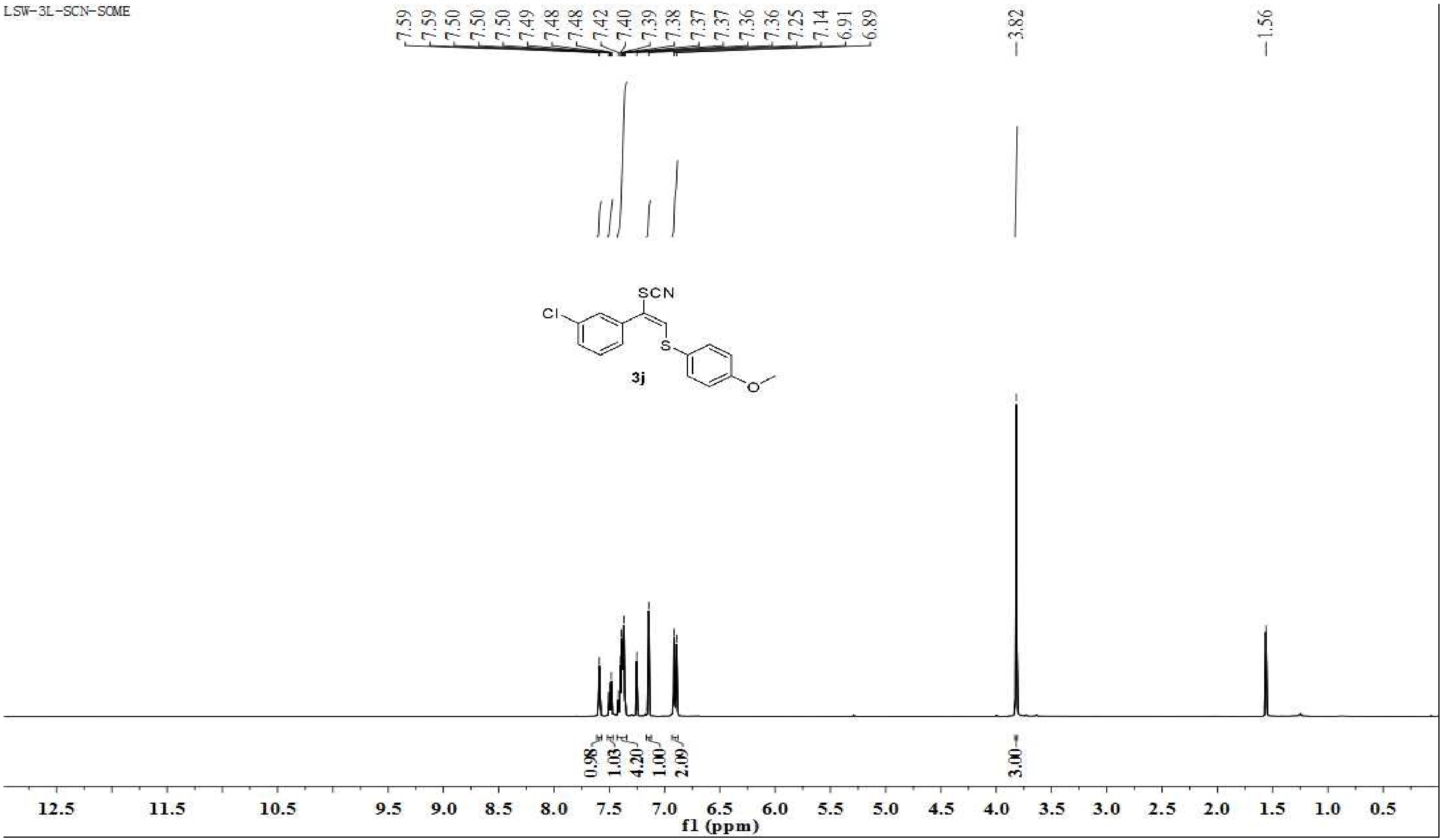

9.

20. ^{13}C NMR of **3j** (125 MHz, $CDCl_3$)

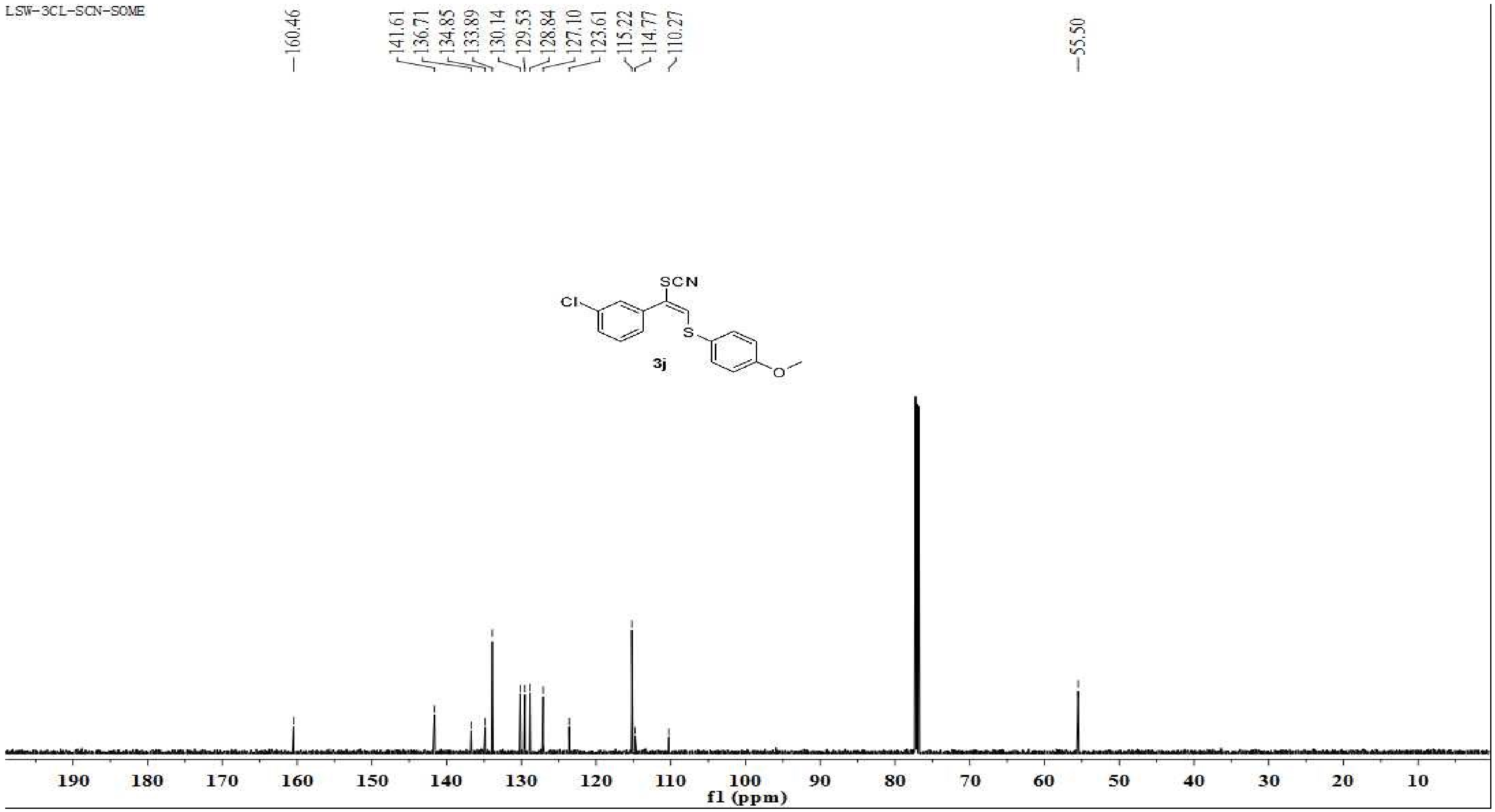

LSW-3CL-SCN-SOME
160.46
141.61
136.71
134.85
133.89
130.14
129.53
128.84
127.10
123.61
115.22
114.77
110.27
55.50
SCN
Cl
S
O
3j
190
180
170
160
150
140
130
120
110
100
90
80
70
60
50
40
30
20
10
f1 (ppm)

21. ^{1}H NMR of **3k** (600 MHz, $CDCl_3$)

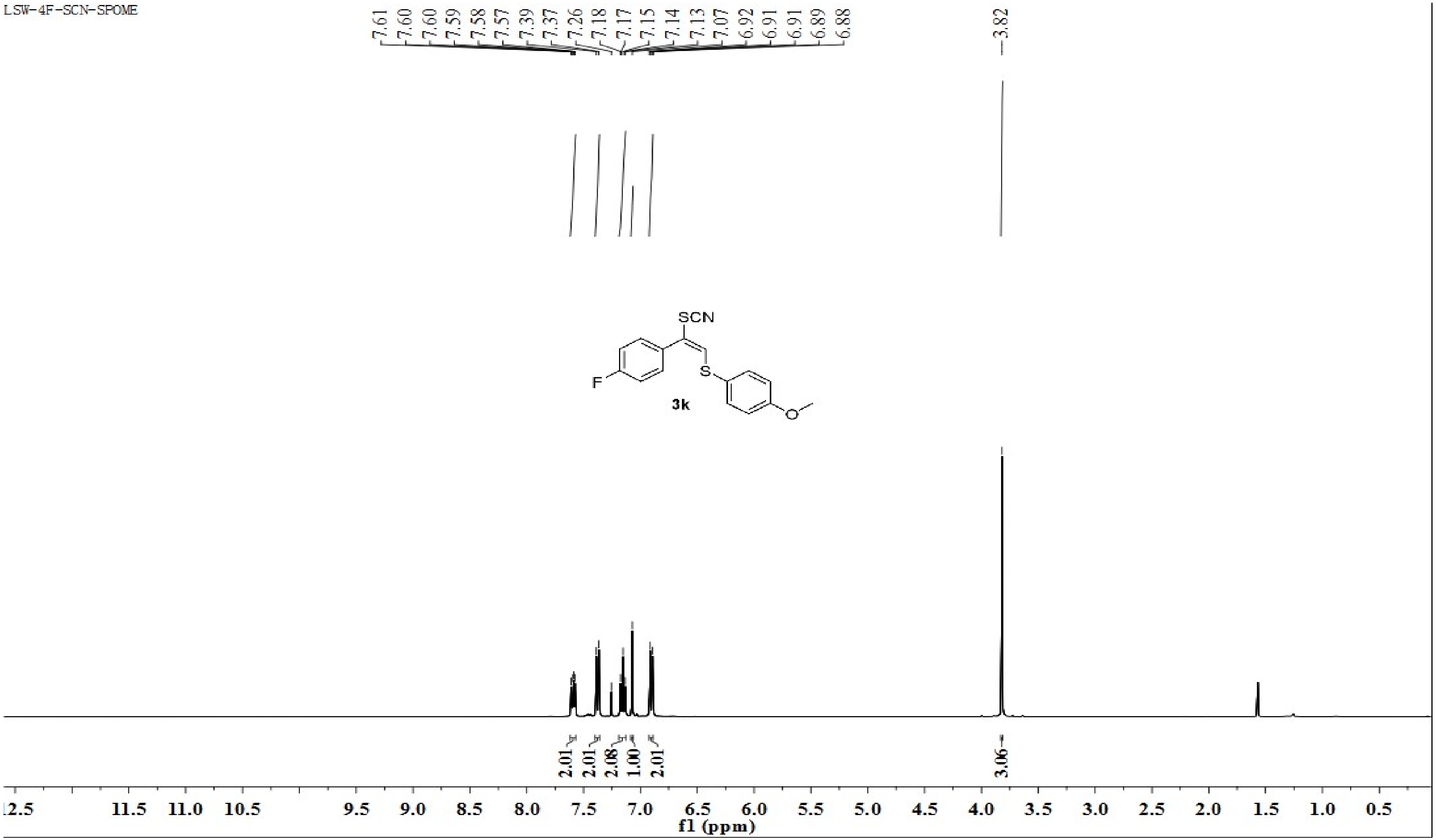

22. ^{13}C NMR of **3k** (125 MHz, $CDCl_3$)

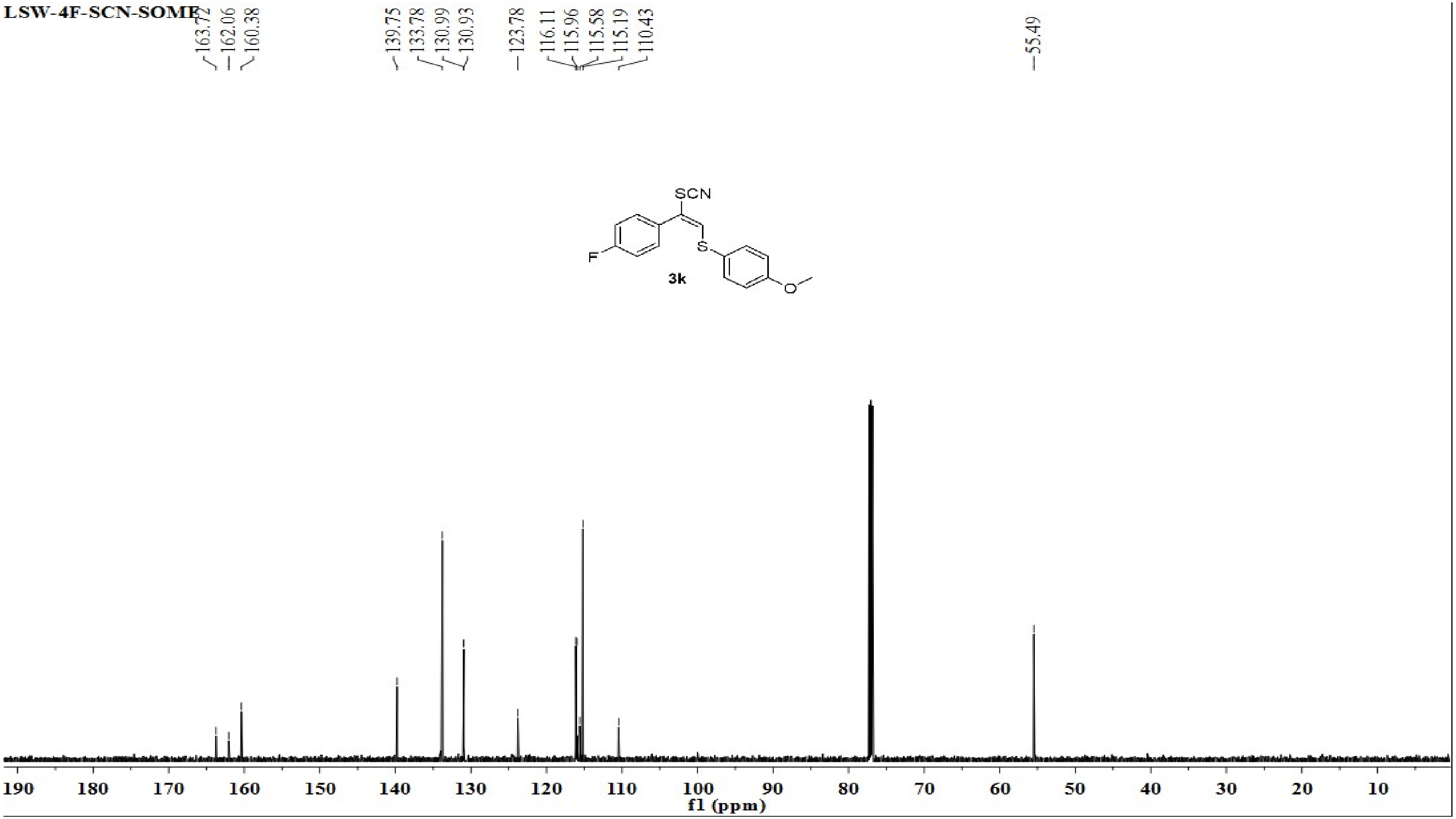

^{19}F NMR of **3k** (600 MHz, $CDCl_3$)

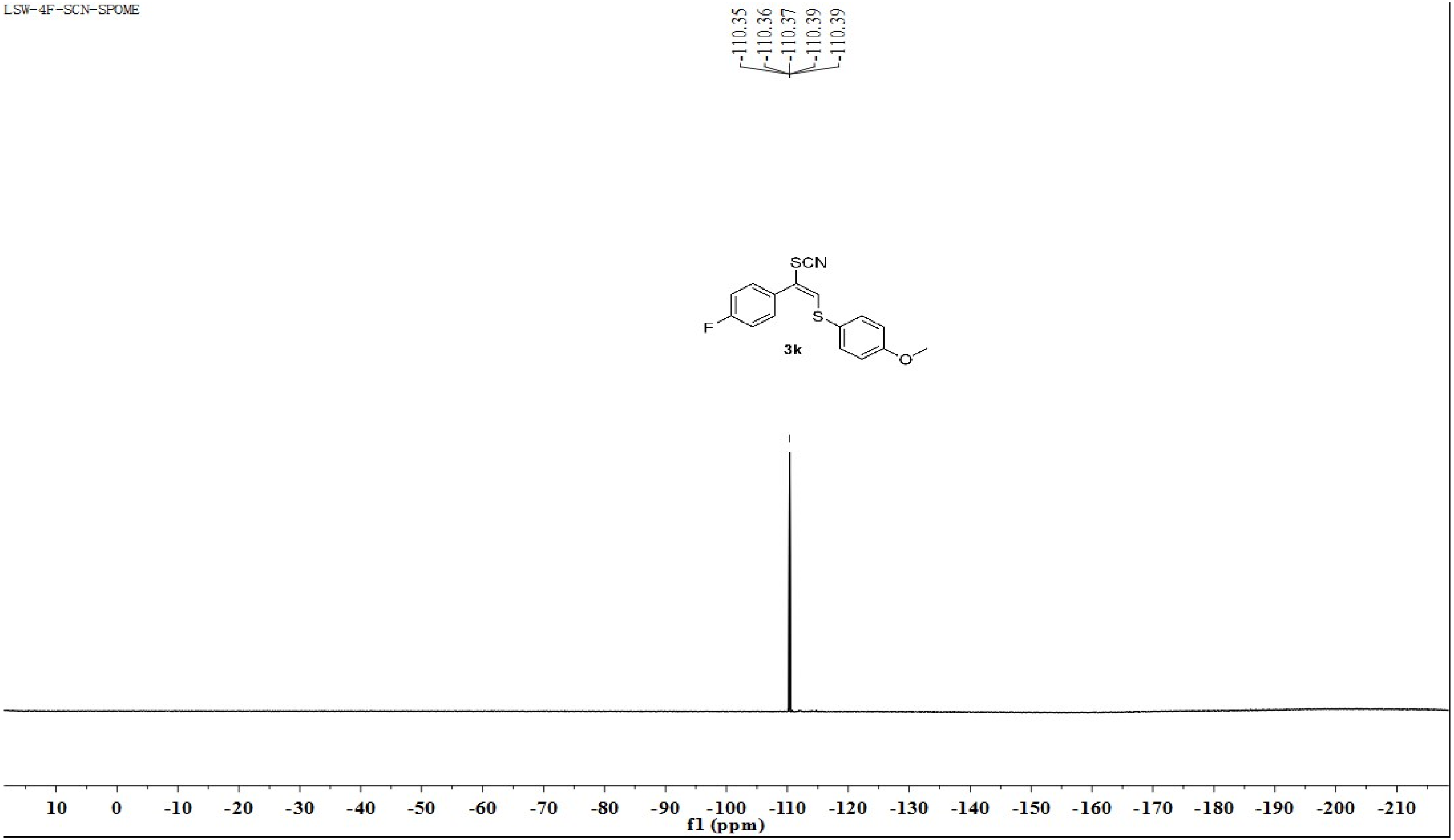

24. ^{1}H NMR of **3l** (600 MHz, $CDCl_3$)

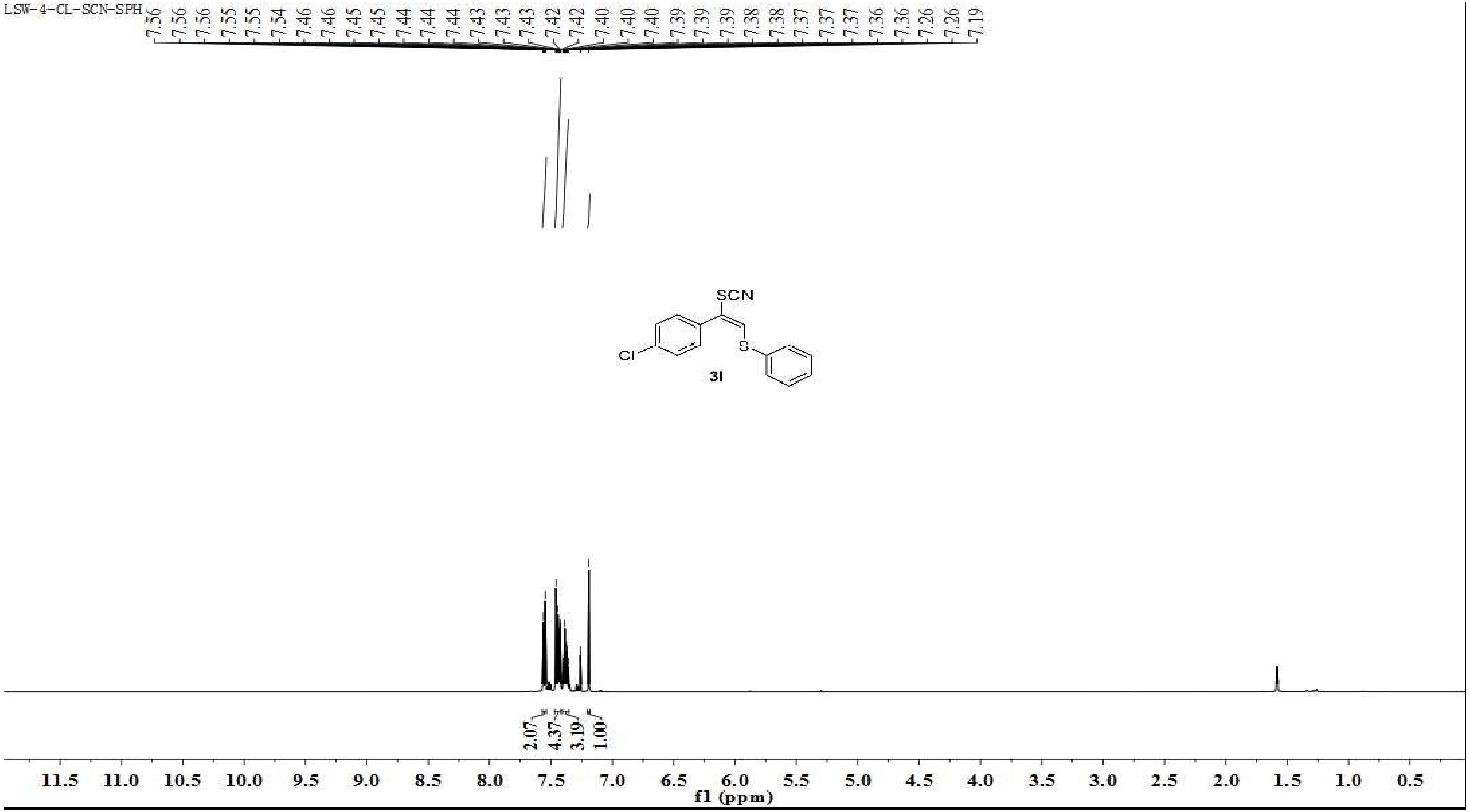

25. ^{13}C NMR of **3l** (125 MHz, $CDCl_3$)

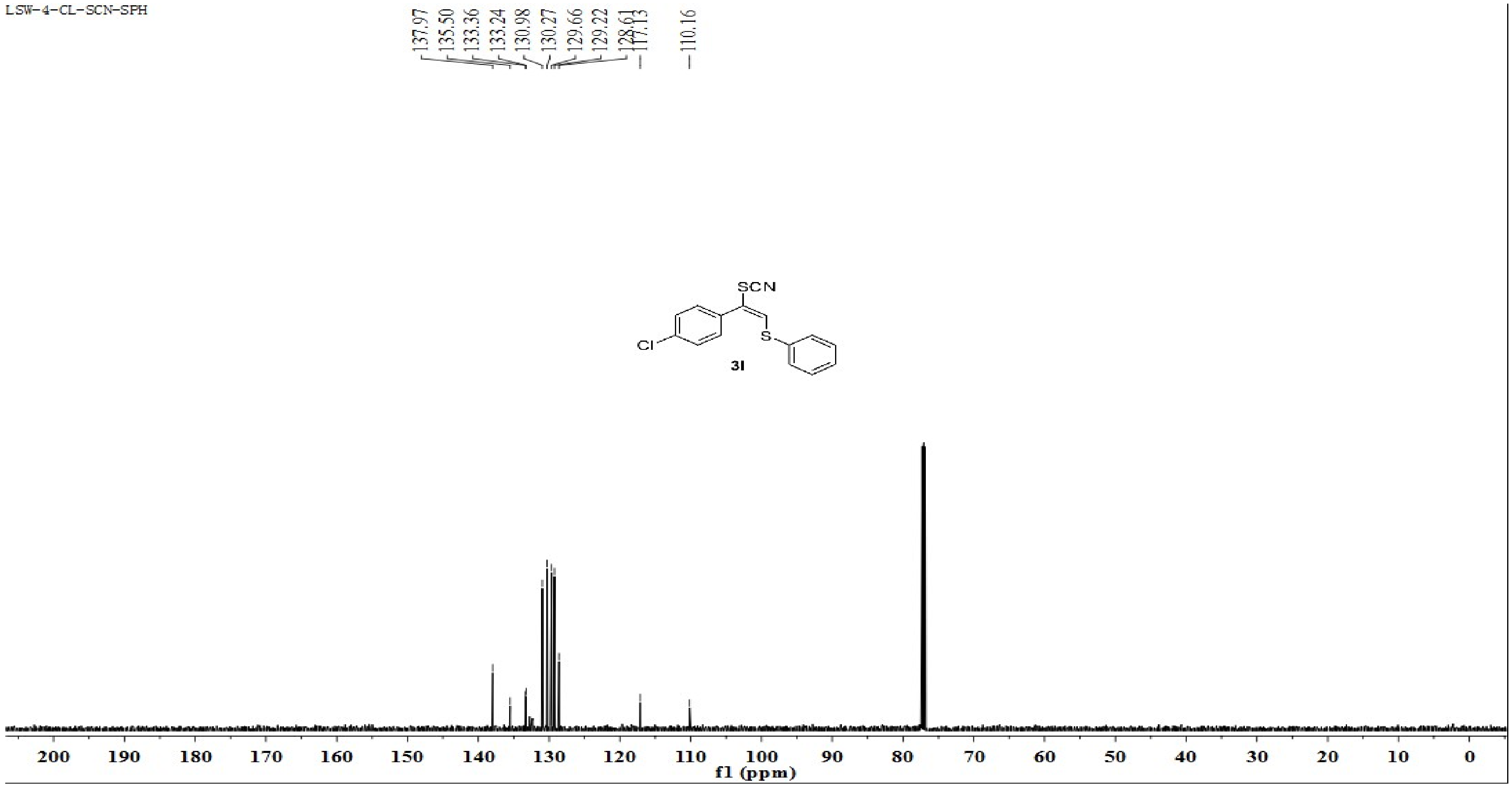

26. ^{1}H NMR of **3m** (600 MHz, $CDCl_3$)

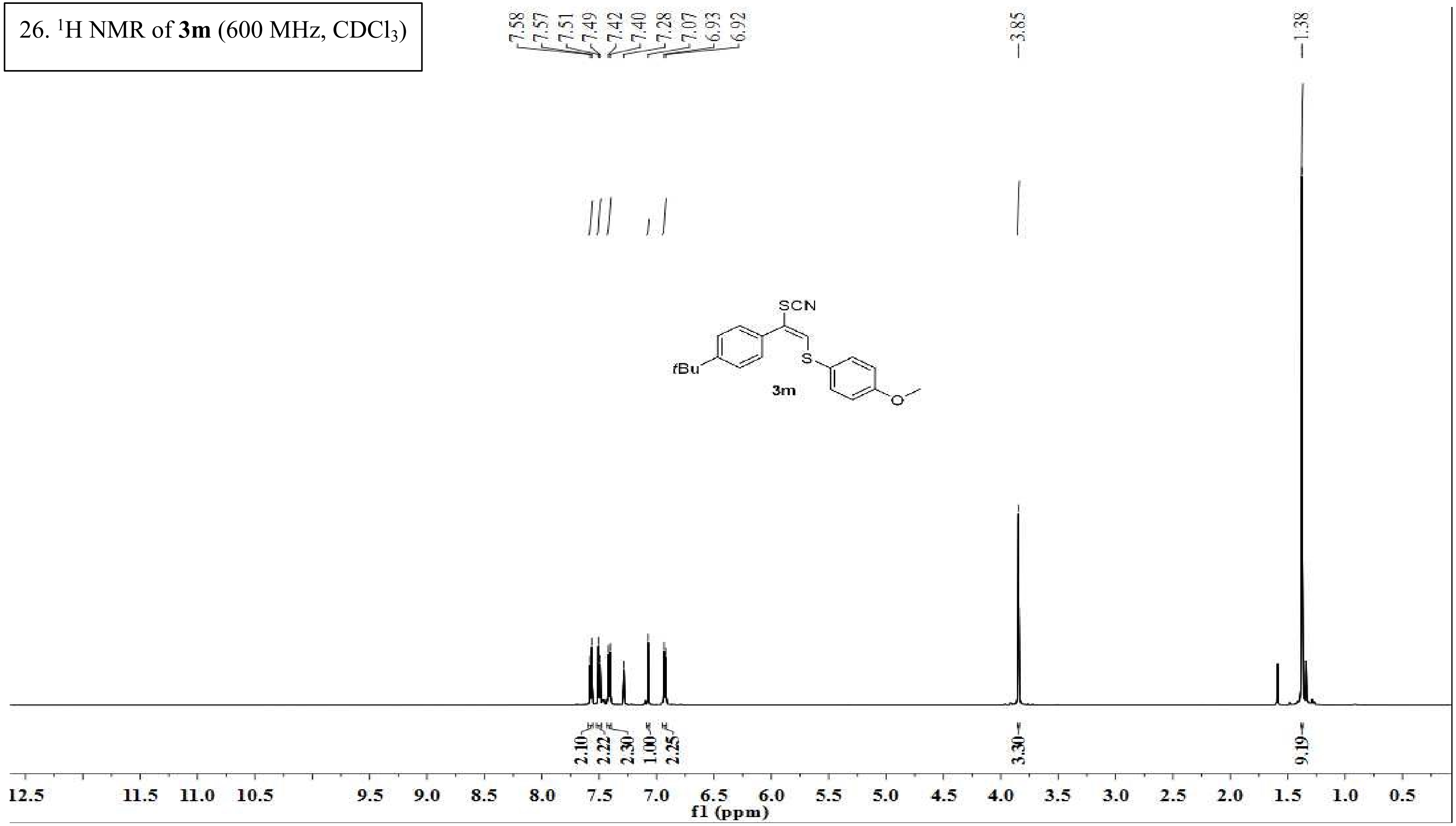

27. ^{13}C NMR of **3m** (125 MHz, $CDCl_3$)

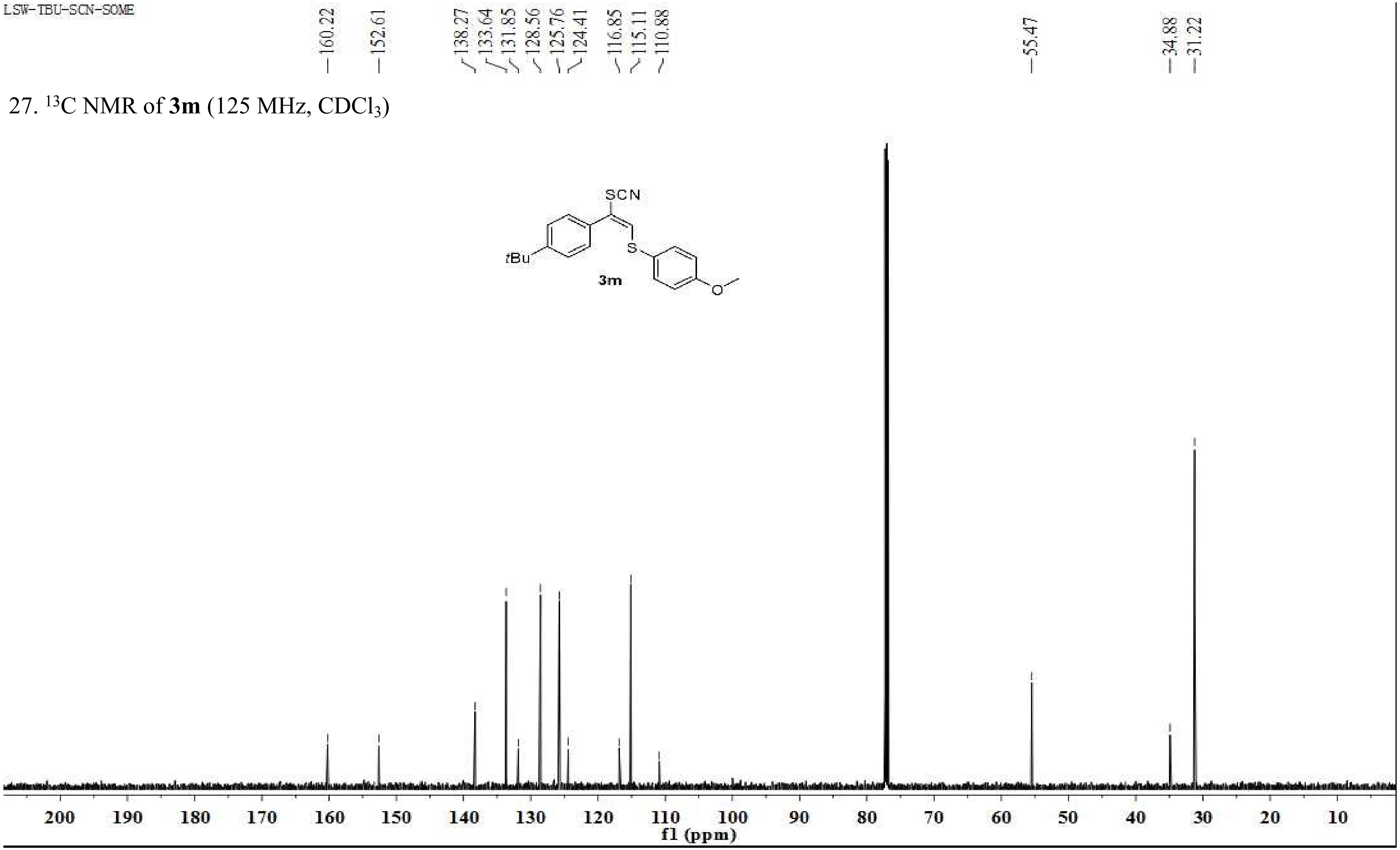

28. ^{1}H NMR of **3n** (600 MHz, $CDCl_3$)

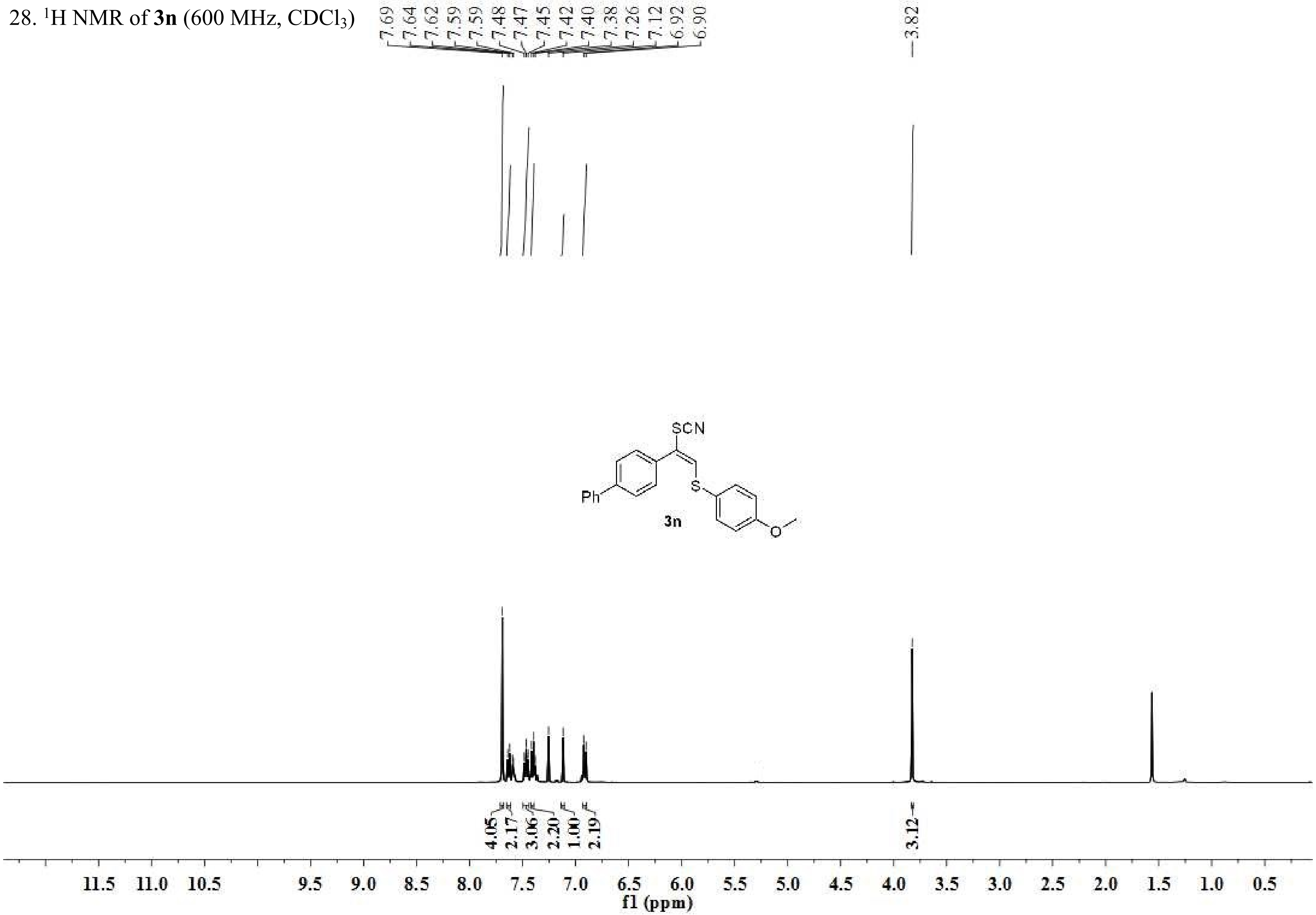

29. ^{13}C NMR of **3n** (125 MHz, $CDCl_3$)

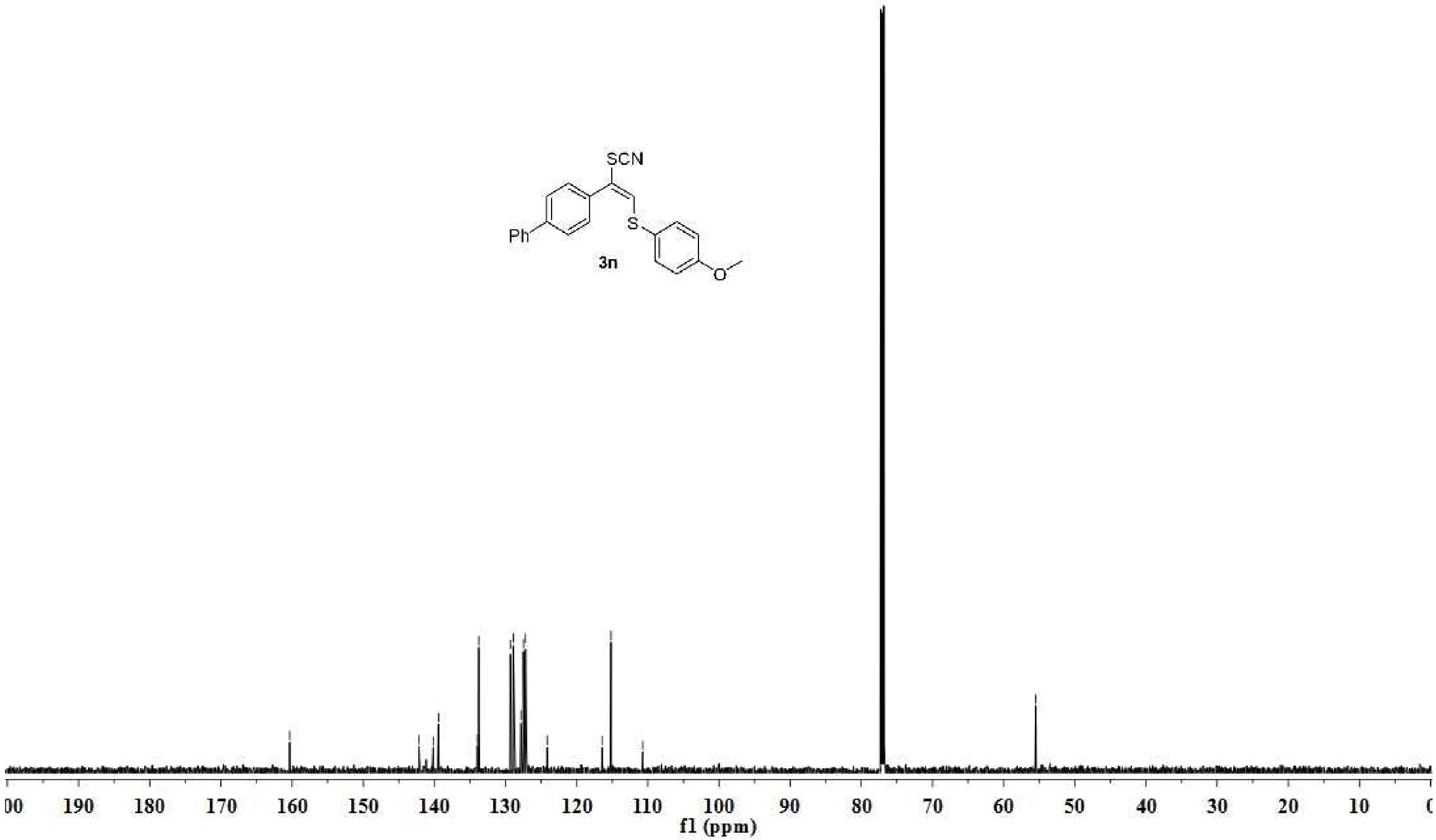

30. ^{1}H NMR of **3o** (600 MHz, $CDCl_3$)

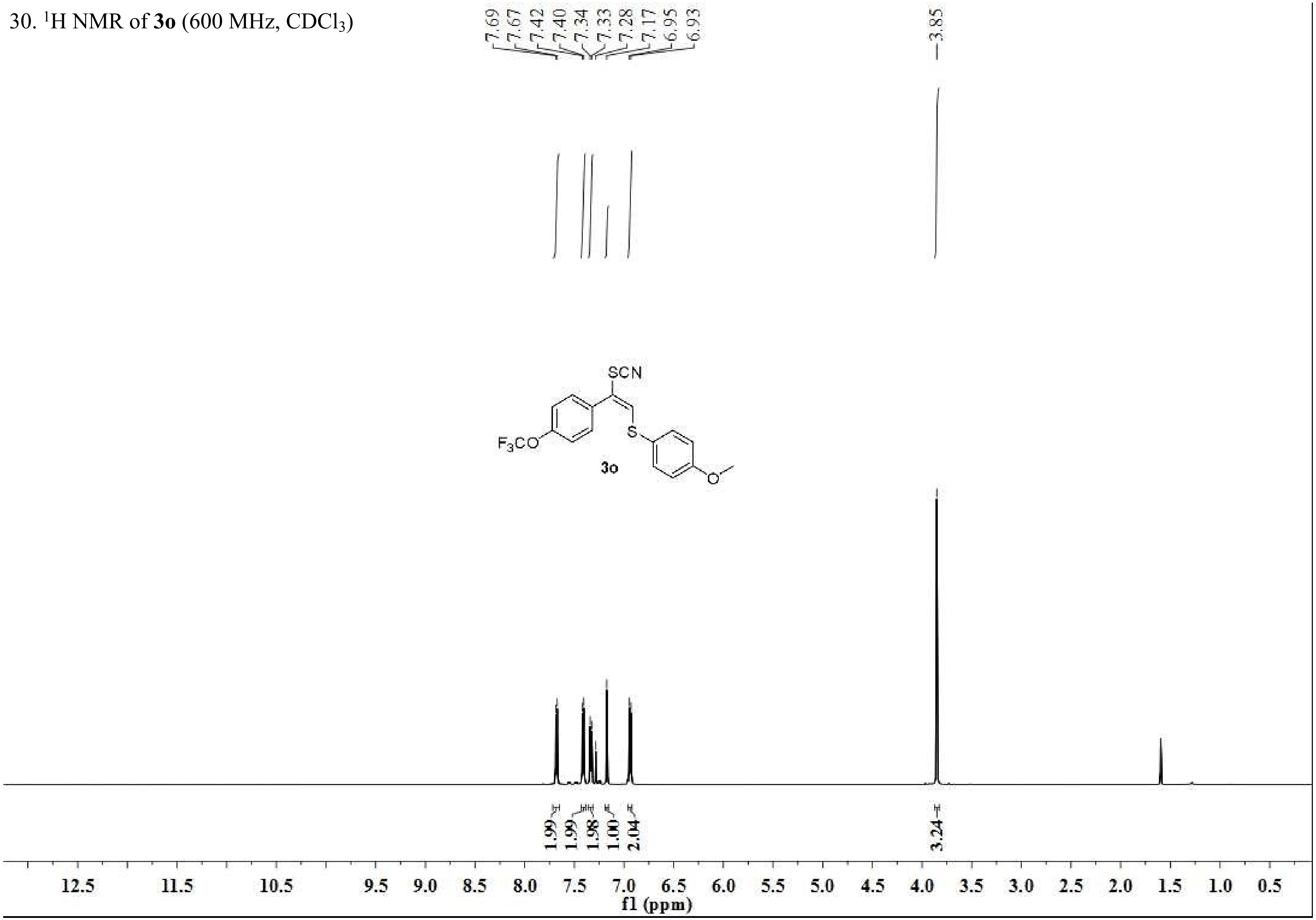

31. ^{13}C NMR of **3o** (125 MHz, $CDCl_3$)

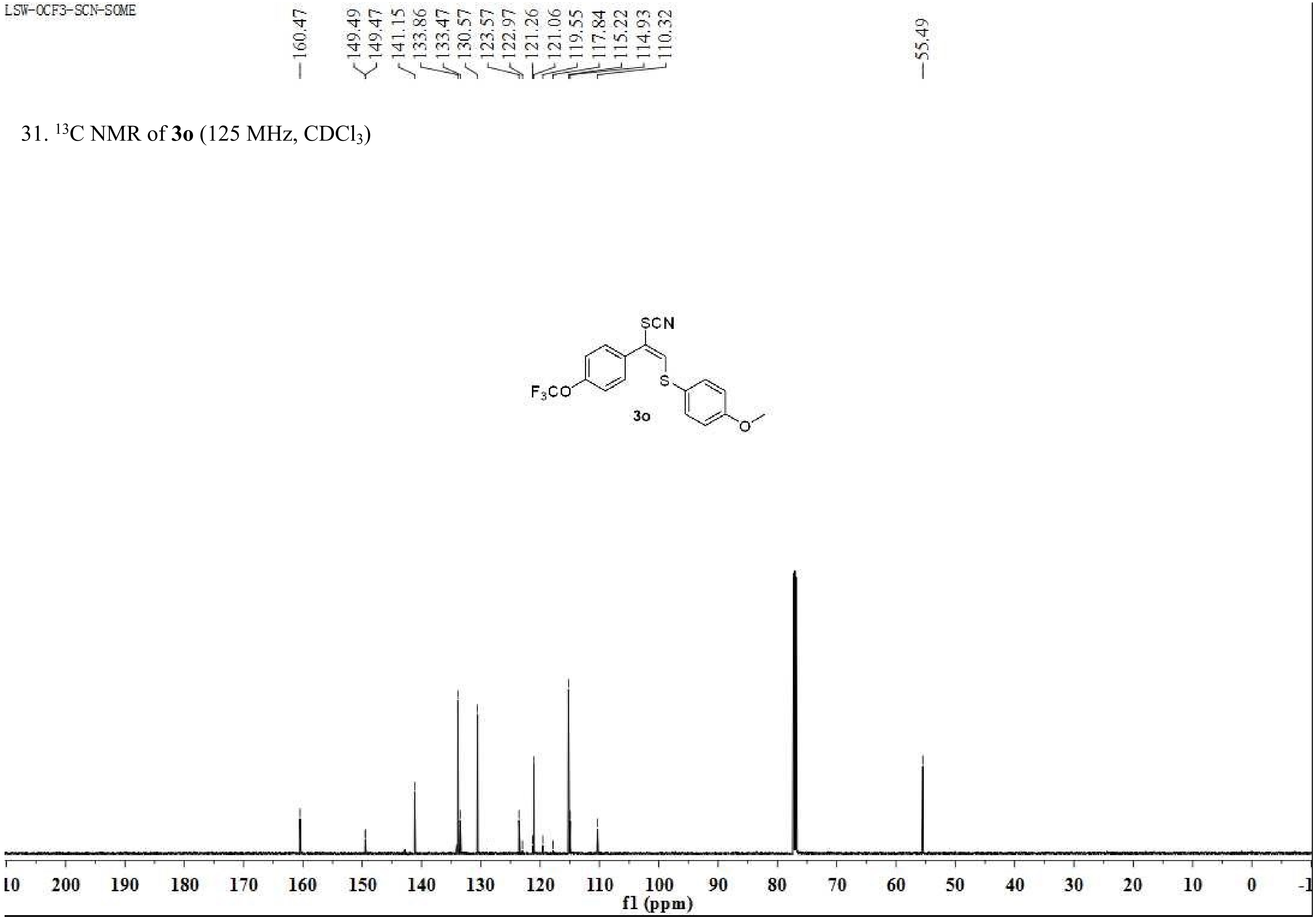

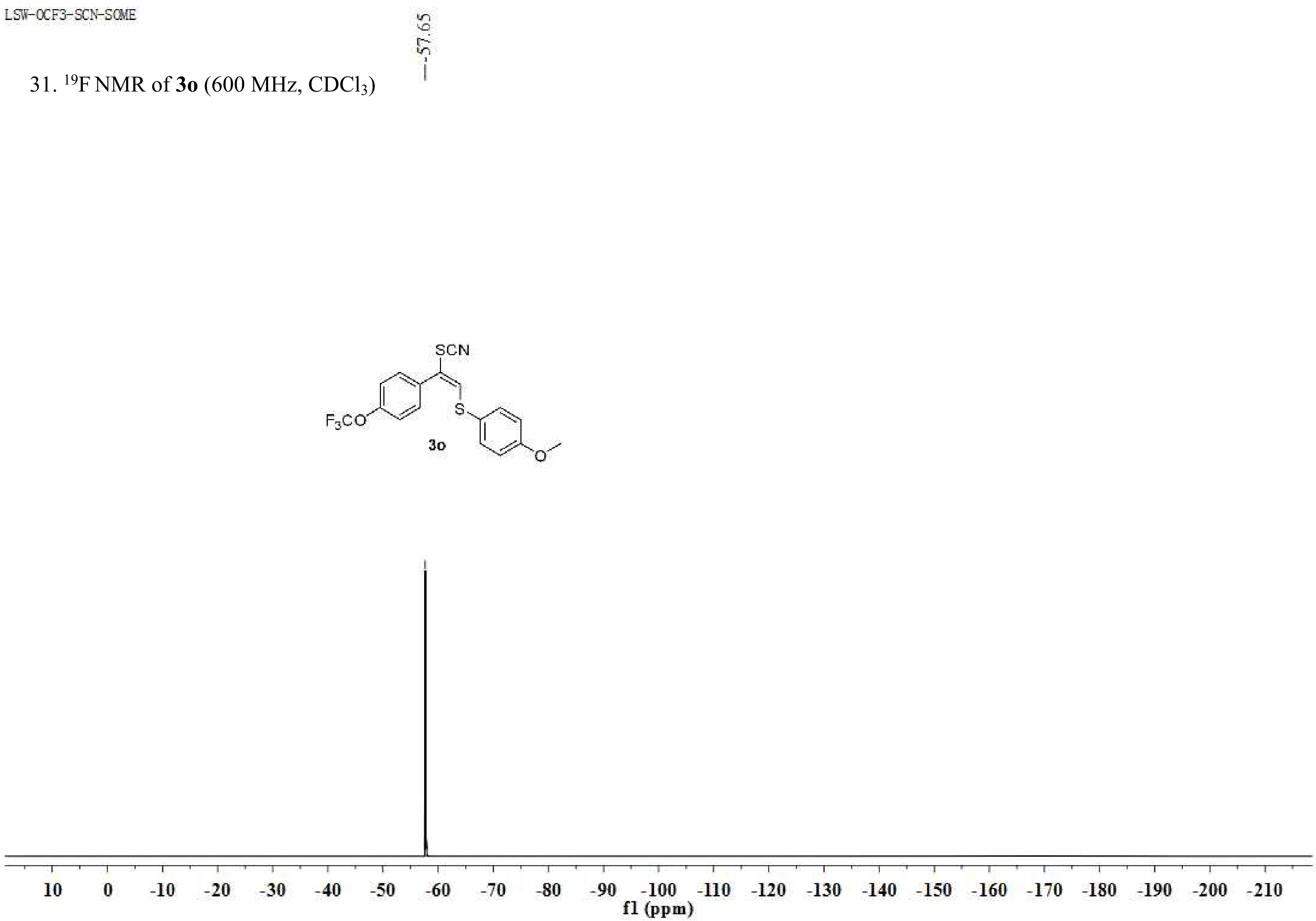

31. ^{19}F NMR of **3o** (600 MHz, $CDCl_3$)

32. ^{1}H NMR of **3p** (600 MHz, $CDCl_3$)

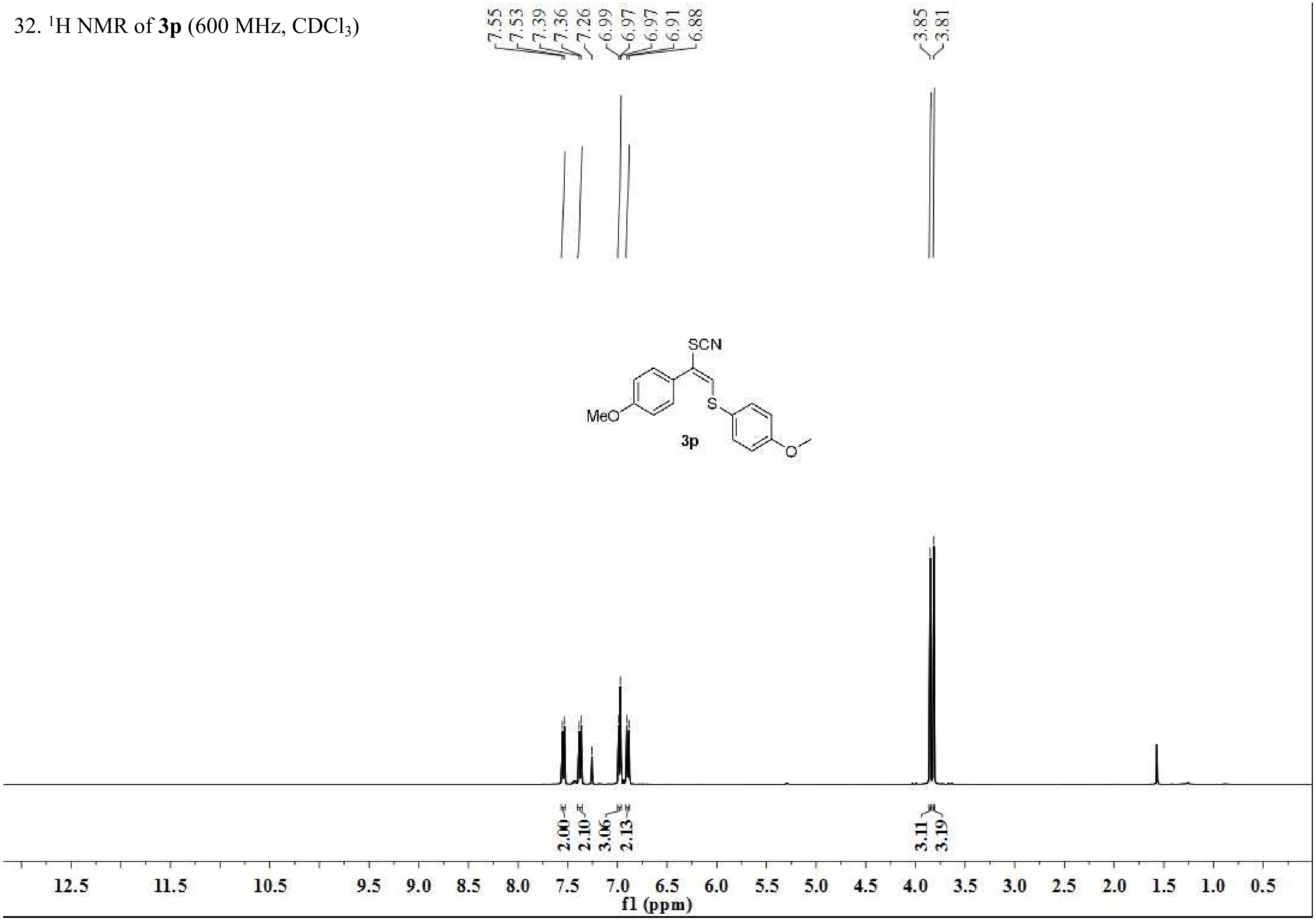

33. ^{13}C NMR of **3p** (125 MHz, $CDCl_3$)

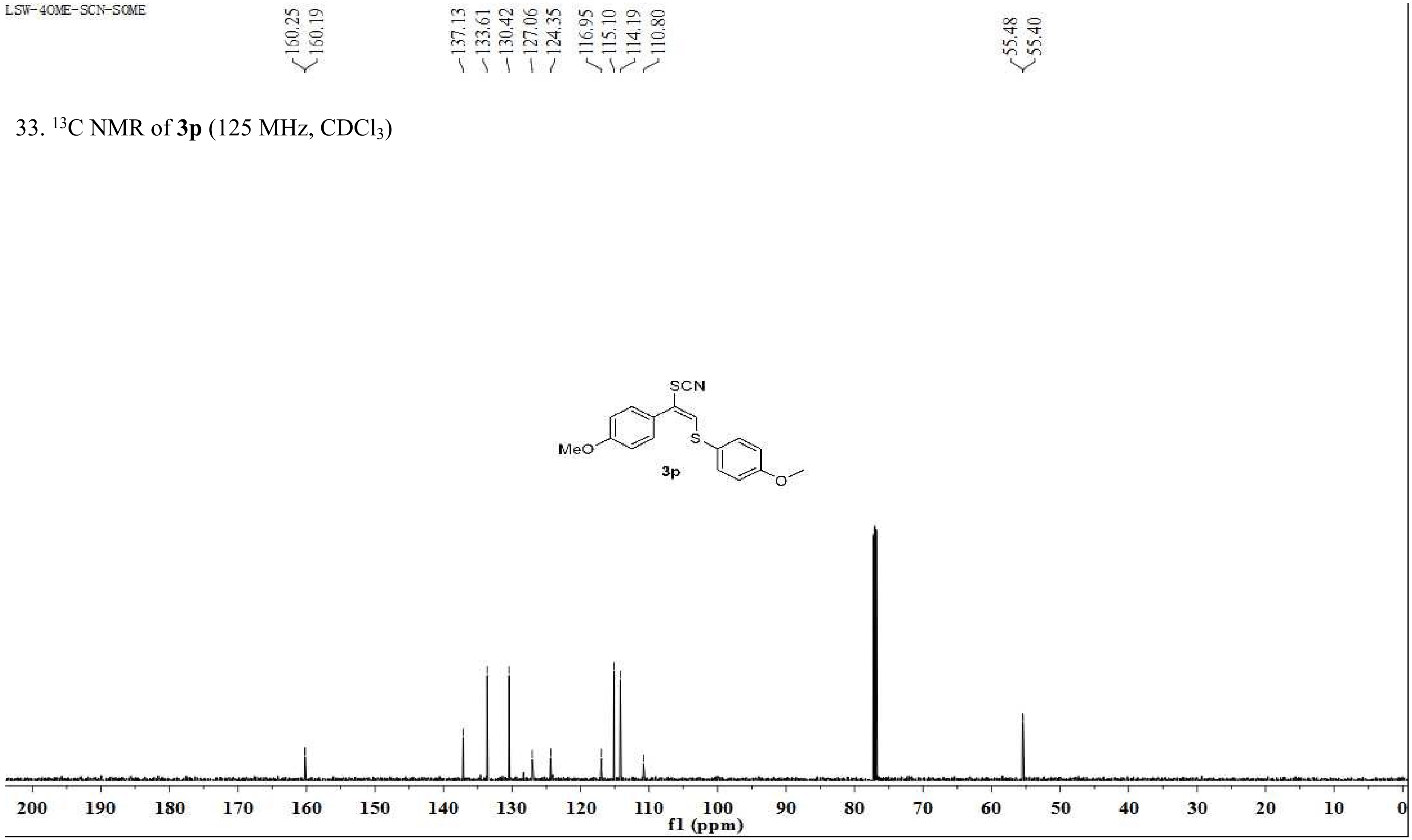

34. ^{1}H NMR of **3q** (600 MHz, $CDCl_3$)

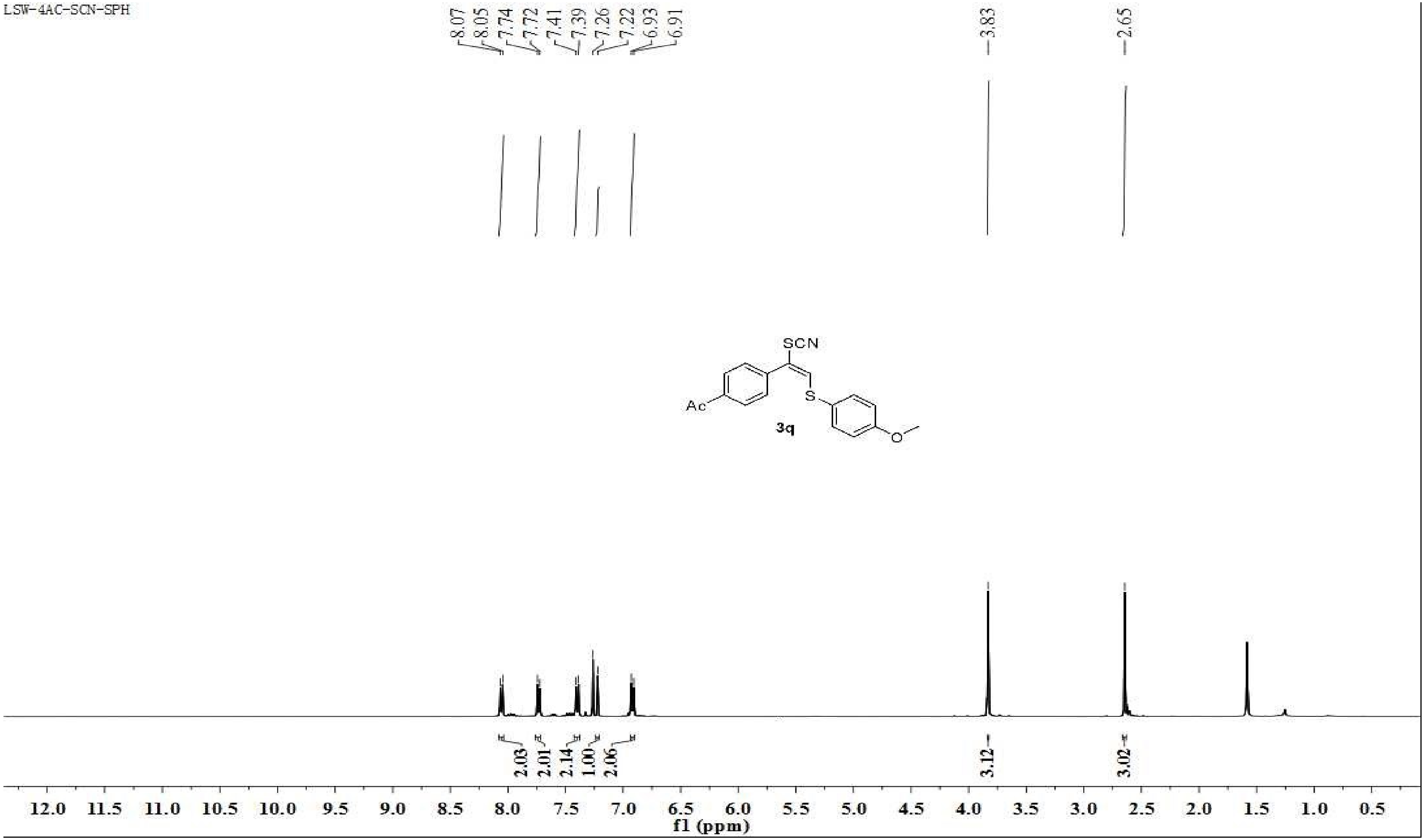

35. ^{13}C NMR of **3q** (125 MHz, $CDCl_3$)

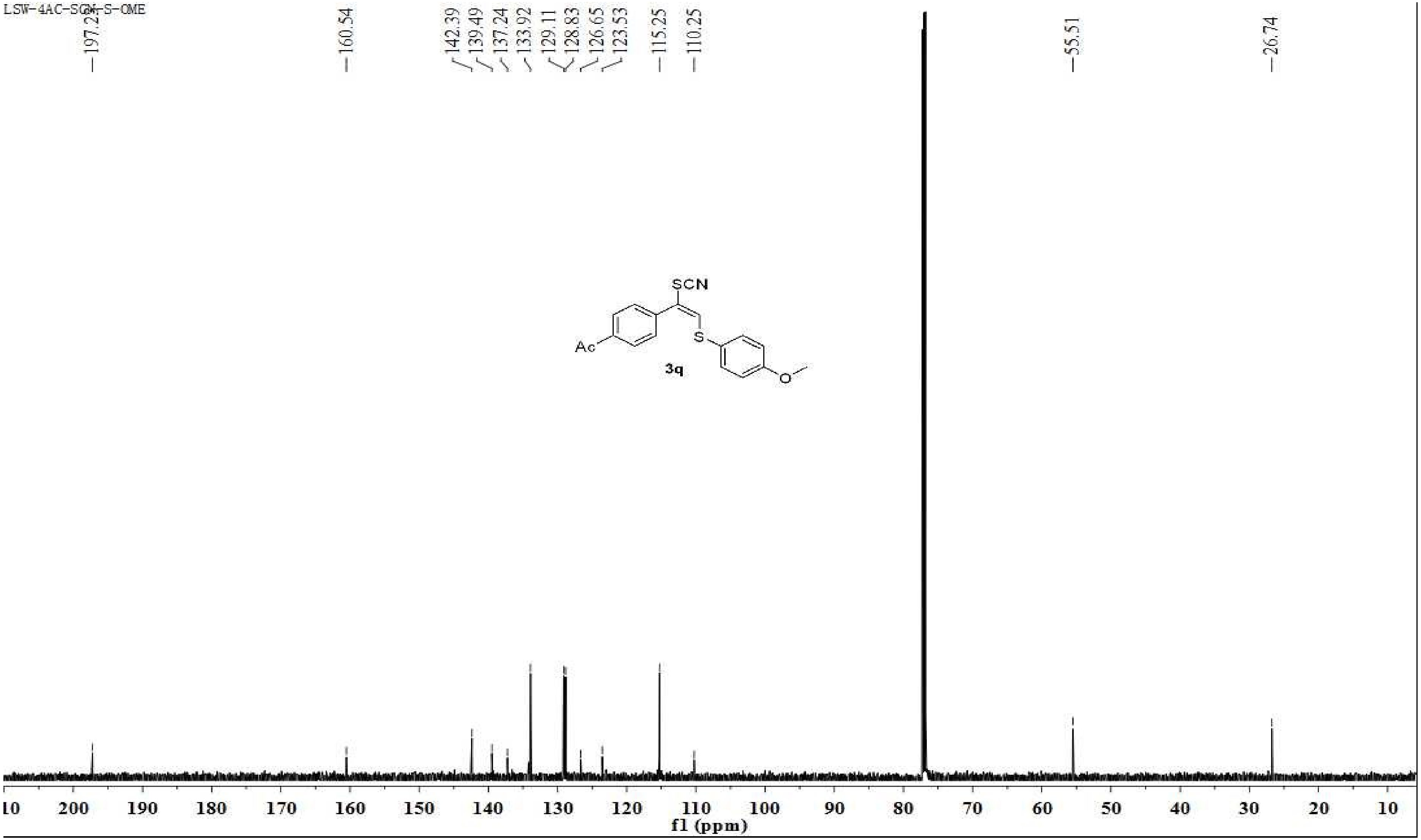

36. ^{1}H NMR of **3r** (600 MHz, $CDCl_3$)

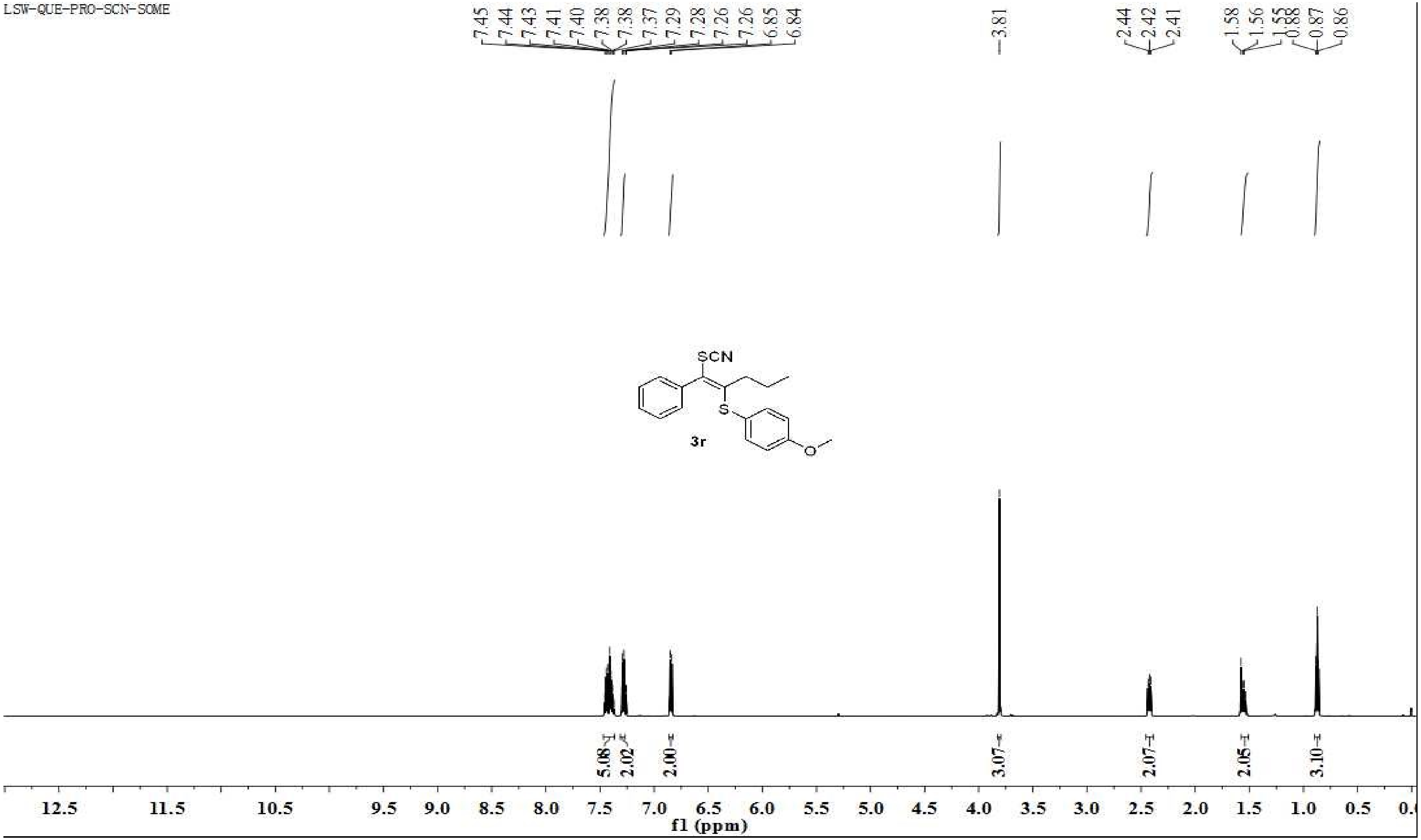

37. ^{13}C NMR of **3r** (125 MHz, $CDCl_3$)

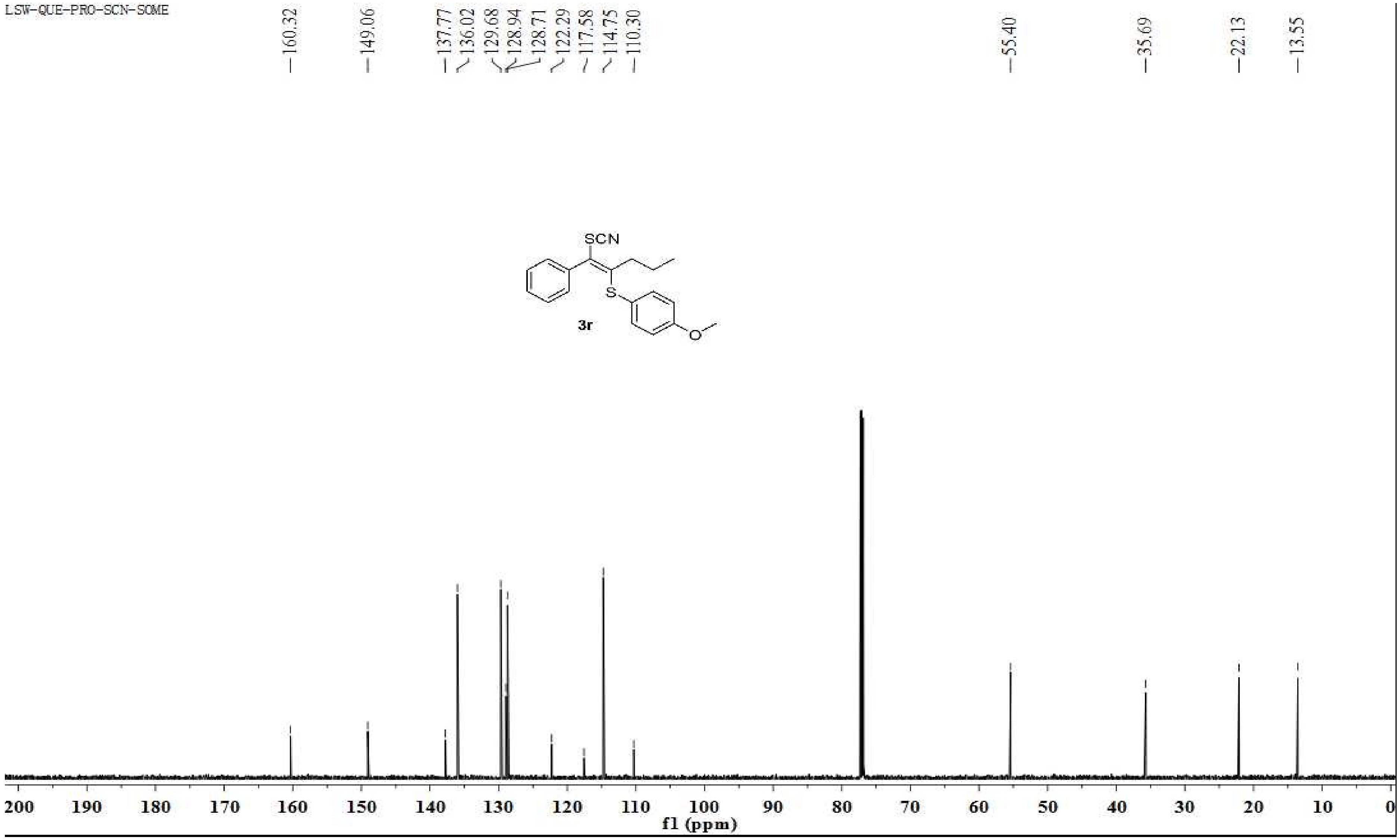

38. ^{1}H NMR of **3s** (125 MHz, CDCl$_3$)

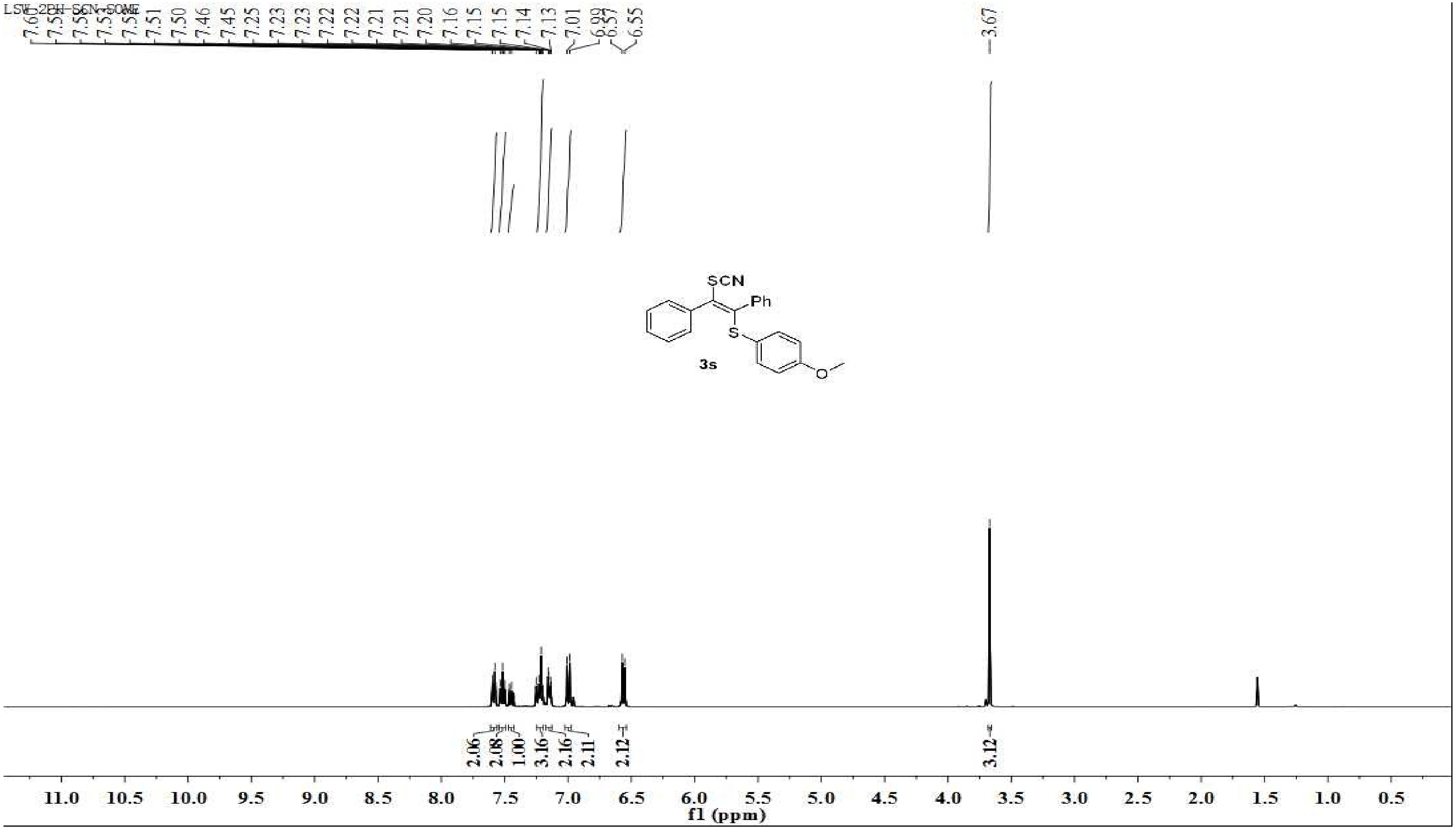

39. ^{13}C NMR of **3s** (125 MHz, $CDCl_3$)

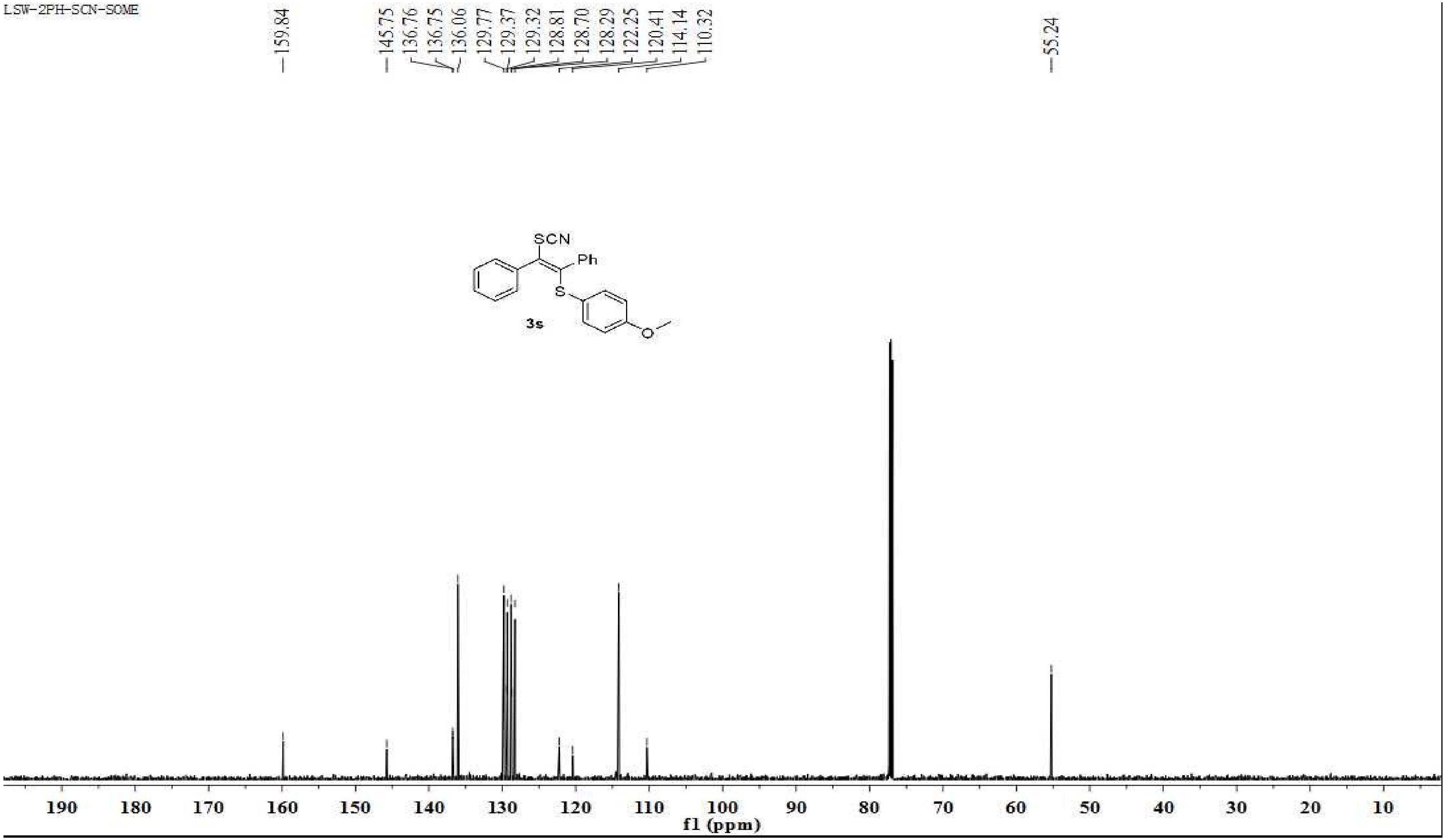

40. ^{1}H NMR of **3t** (600 MHz, $CDCl_3$)

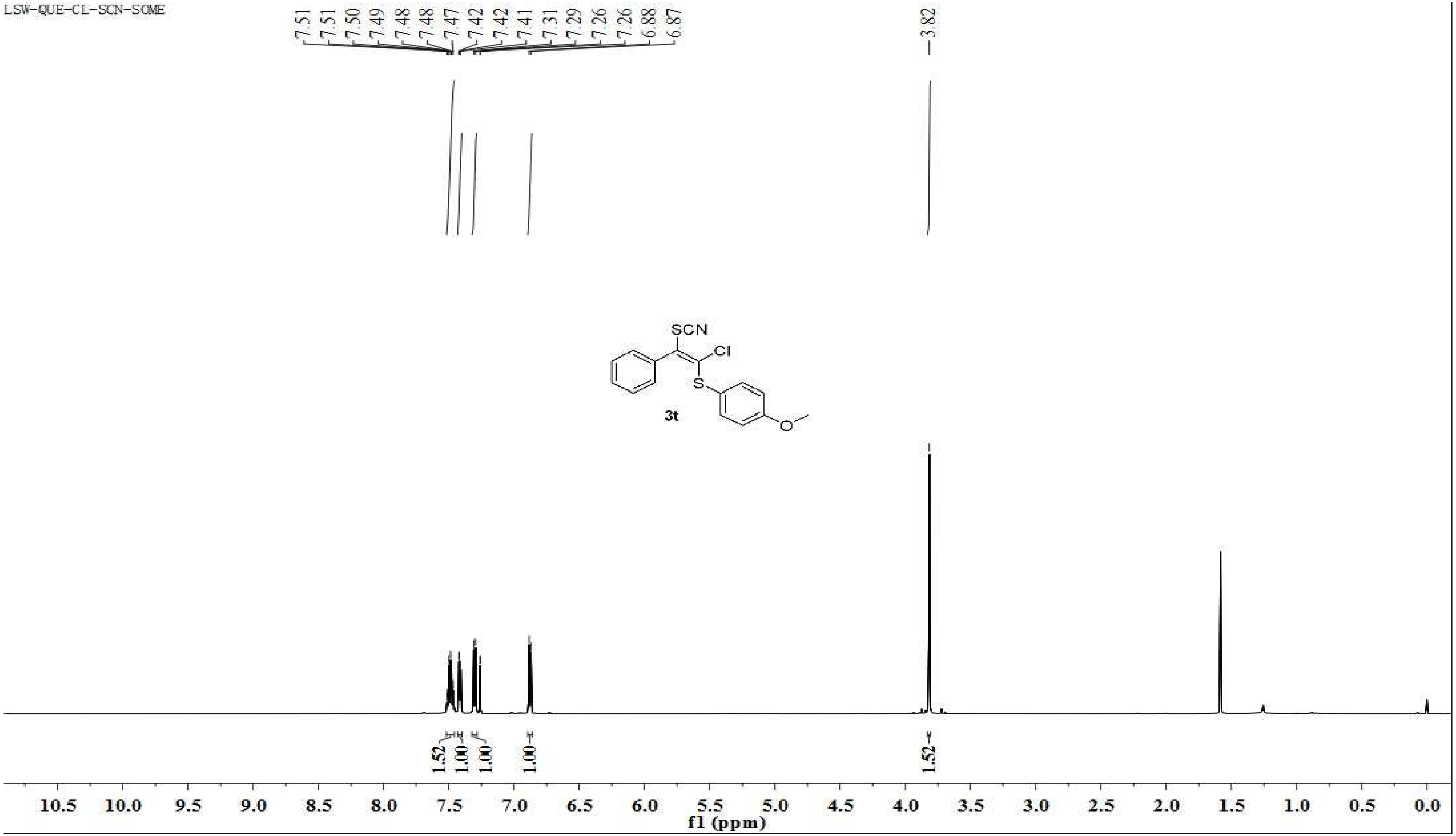

41. ^{13}C NMR of **3t** (125 MHz, $CDCl_3$)

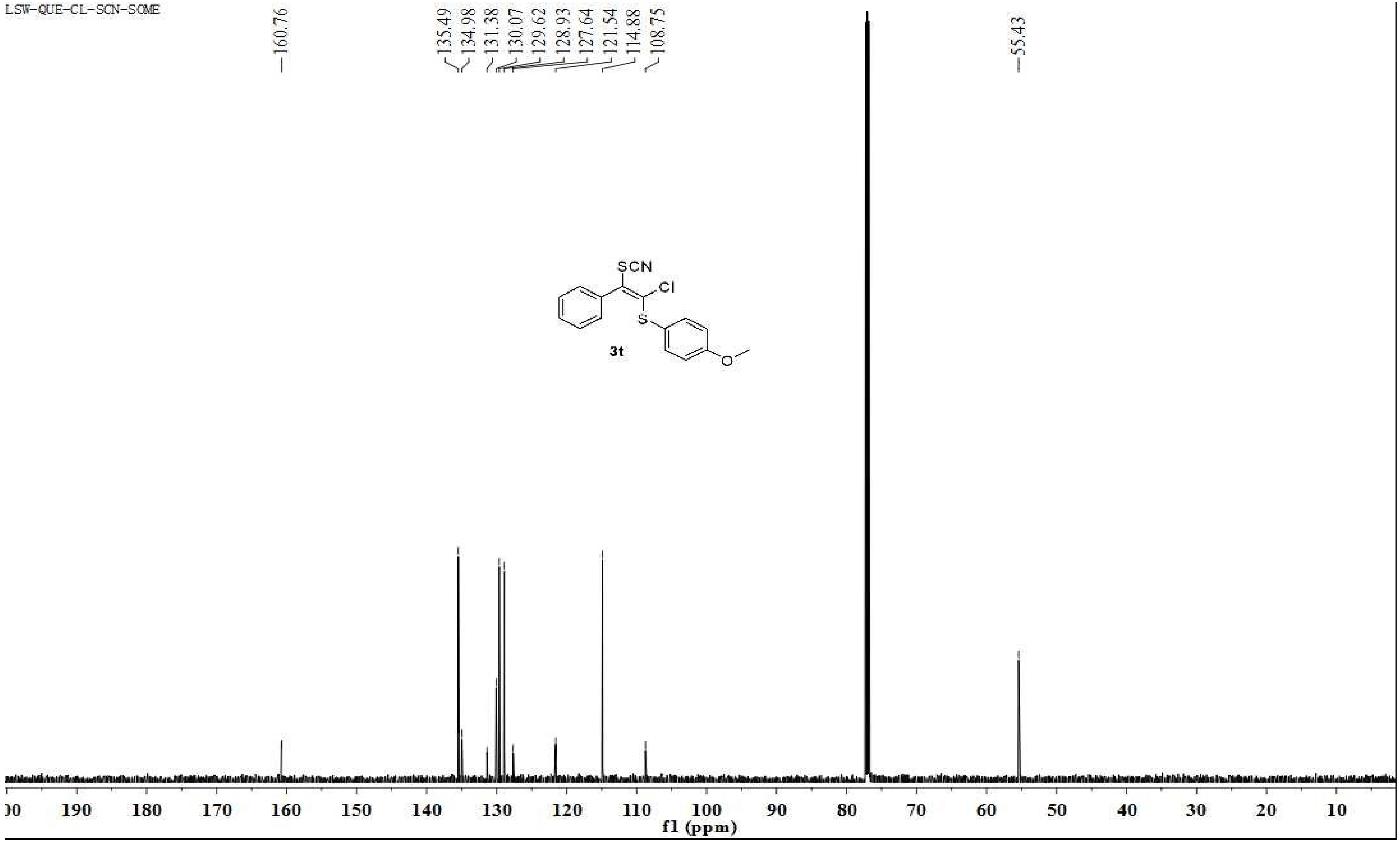

42. ¹H NMR of **3u** (600 MHz, $CDCl_3$)

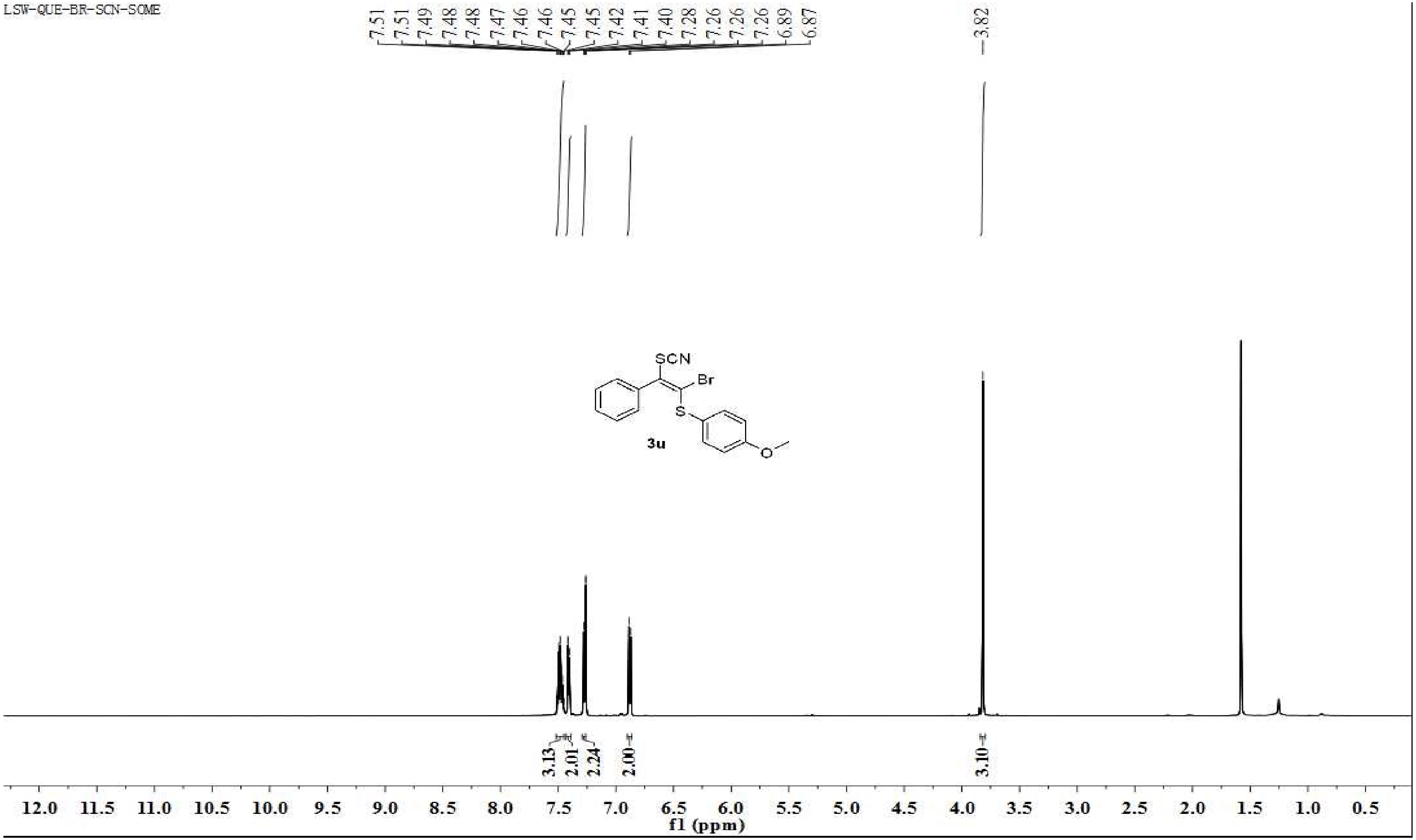

43. ^{13}C NMR of **3u** (125 MHz, $CDCl_3$)

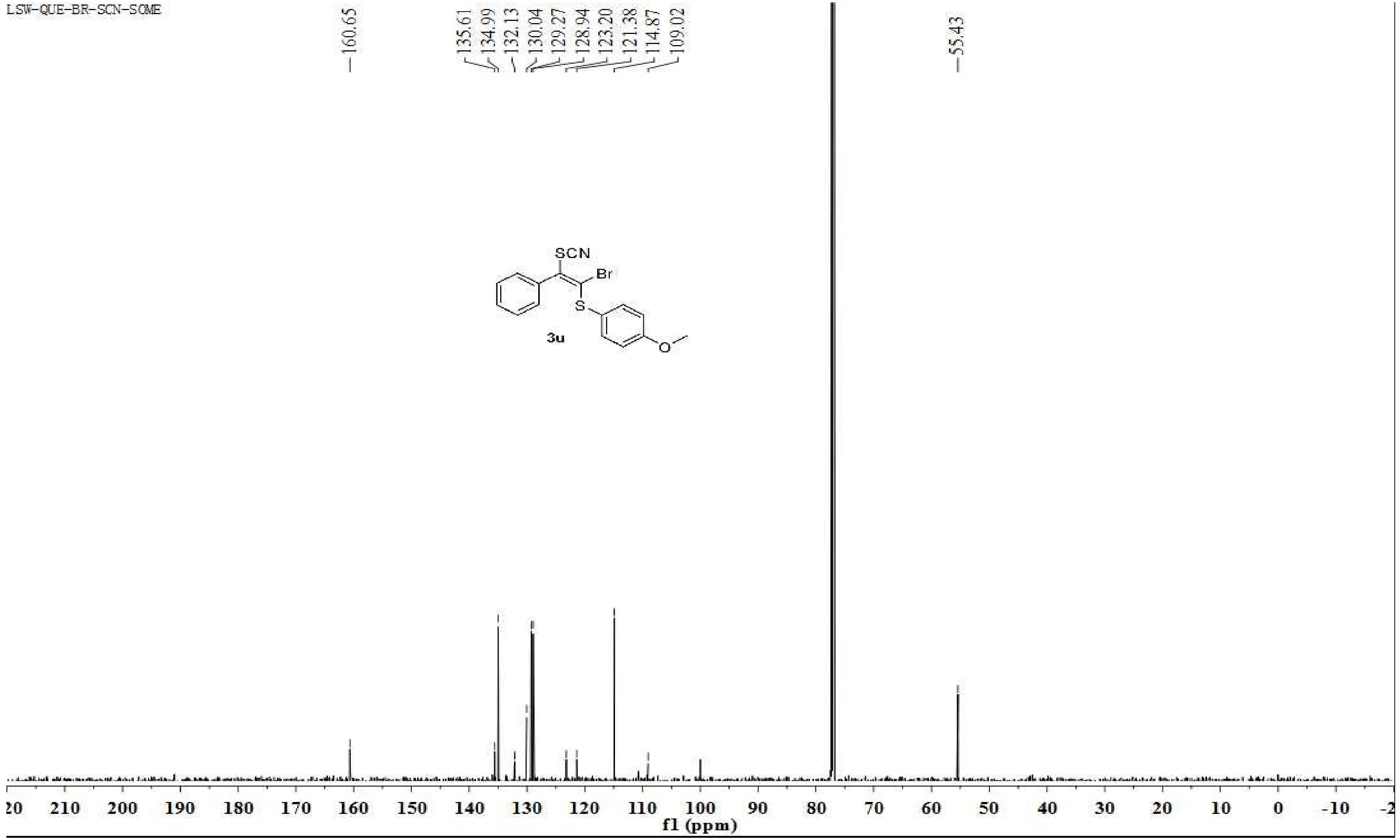

44. ^{1}H NMR of **3v** (600 MHz,CDCl$_3$)

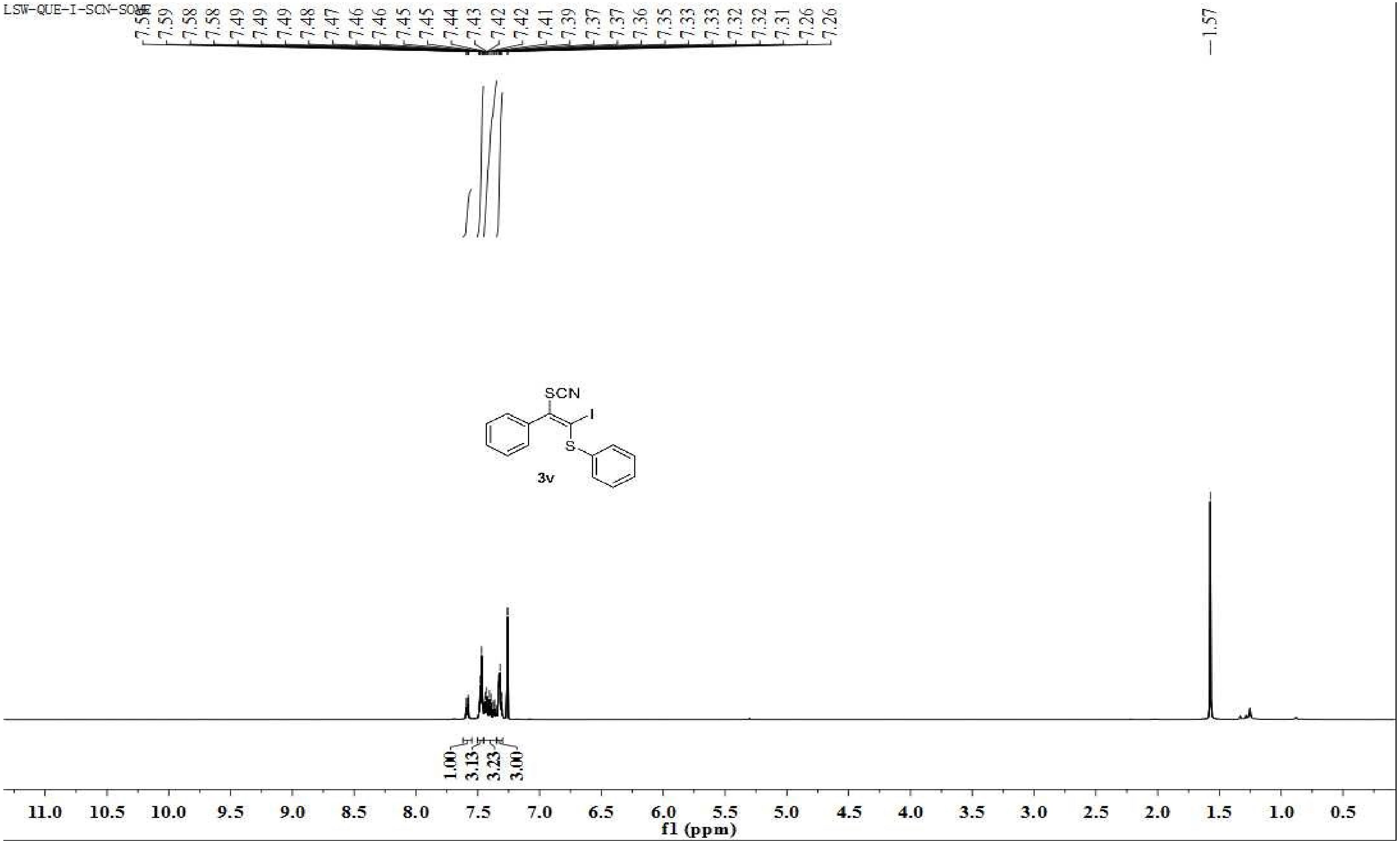

45. ^{13}C NMR of **3v** (125 MHz,CDCl$_3$)

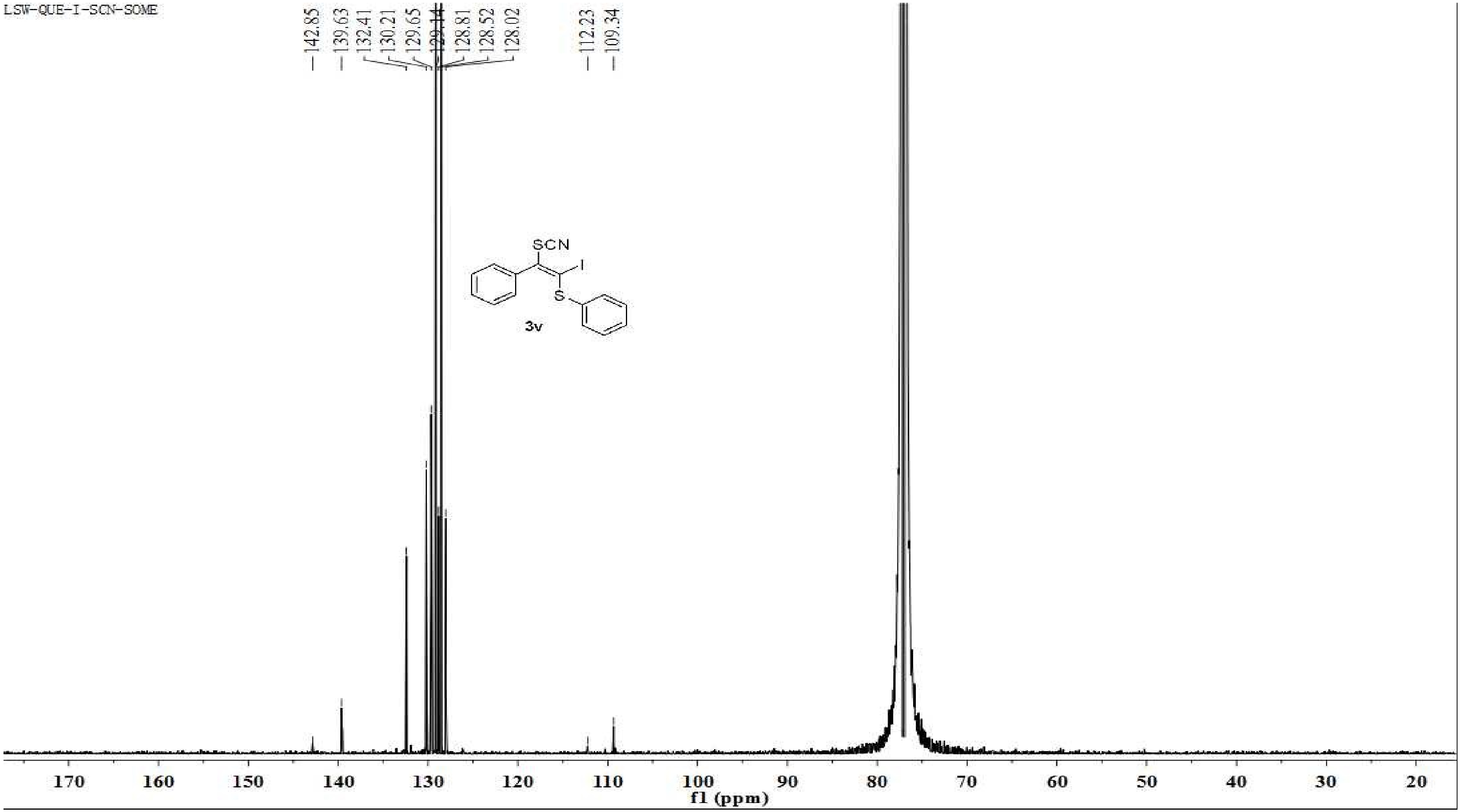

46. ^{1}H NMR of **3v** (600 MHz, $CDCl_3$)

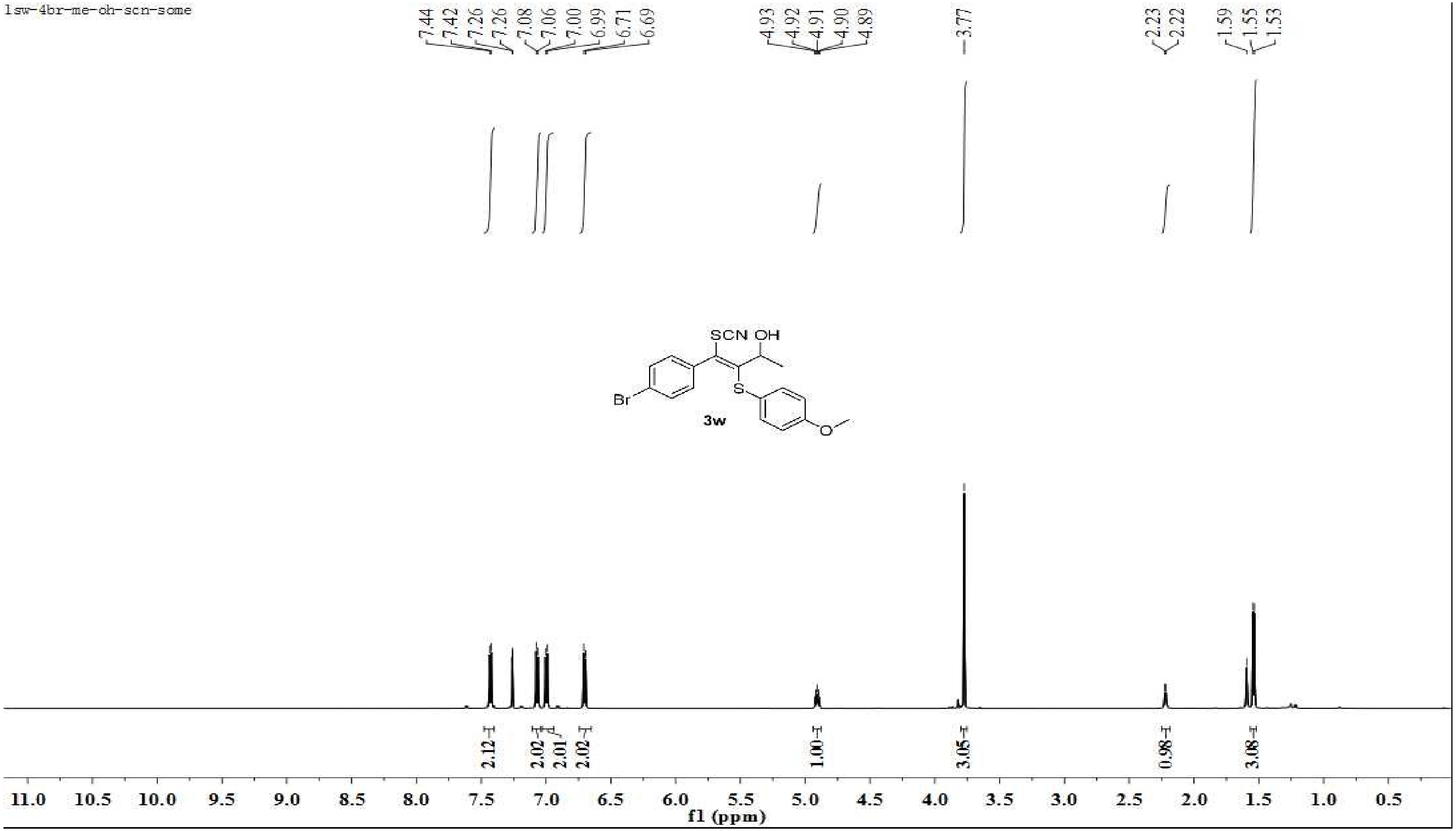

47. ^{13}C NMR of **3w** (125 MHz, $CDCl_3$)

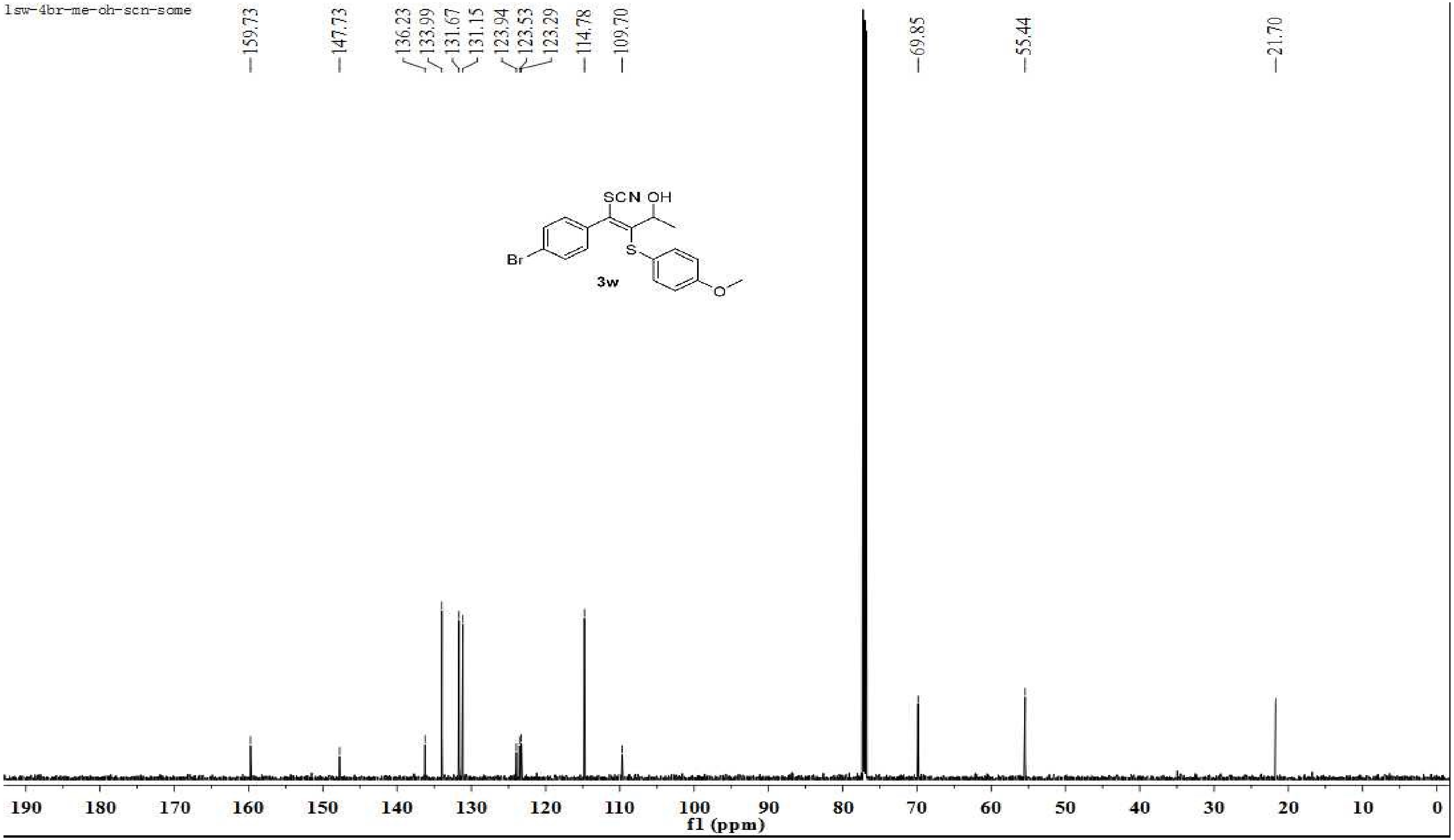

.48 . ^{1}H NMR of **3x** (600 MHz, $CDCl_3$)

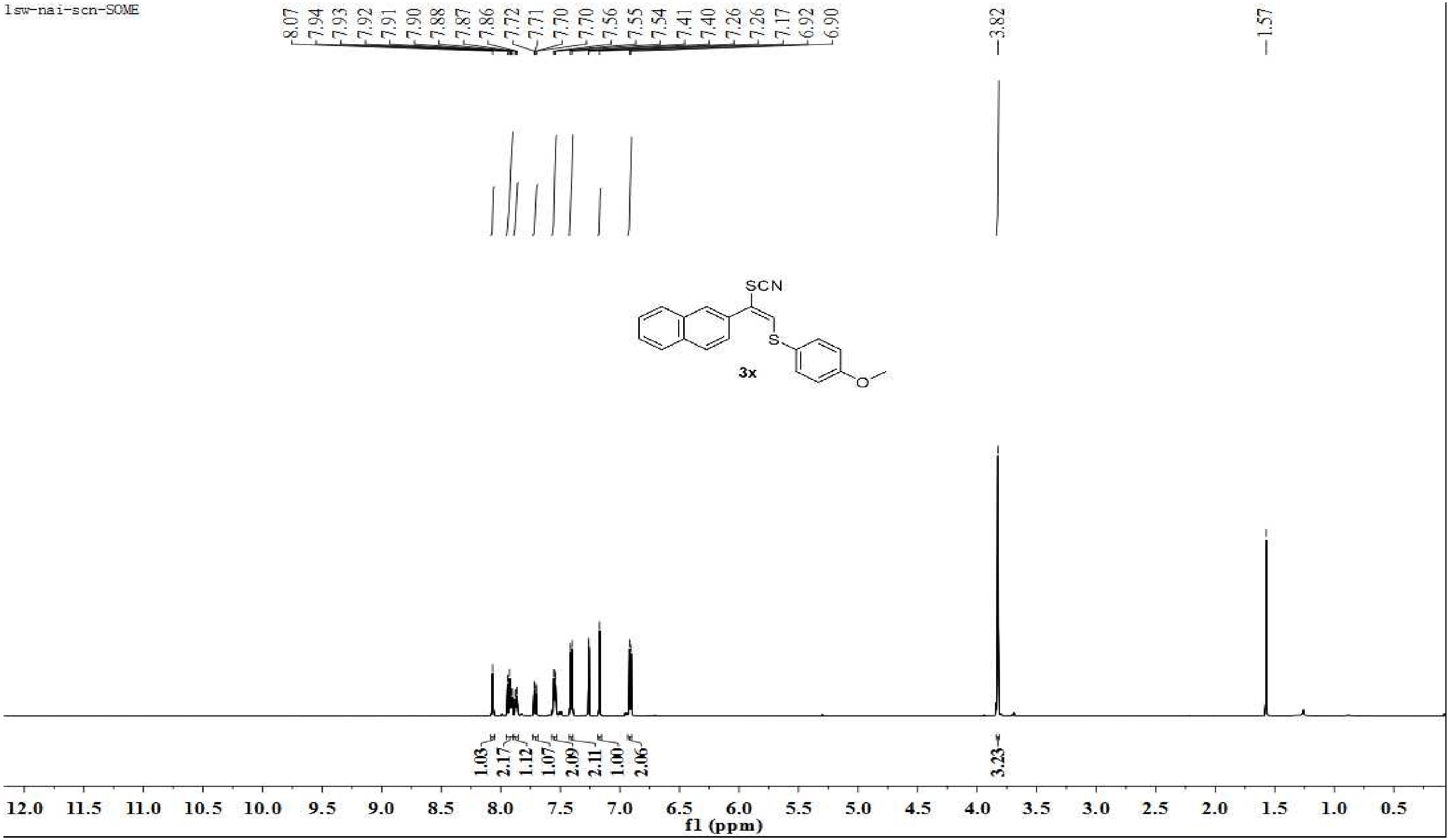

49. ^{13}C NMR of **3x** (125 MHz, $CDCl_3$)

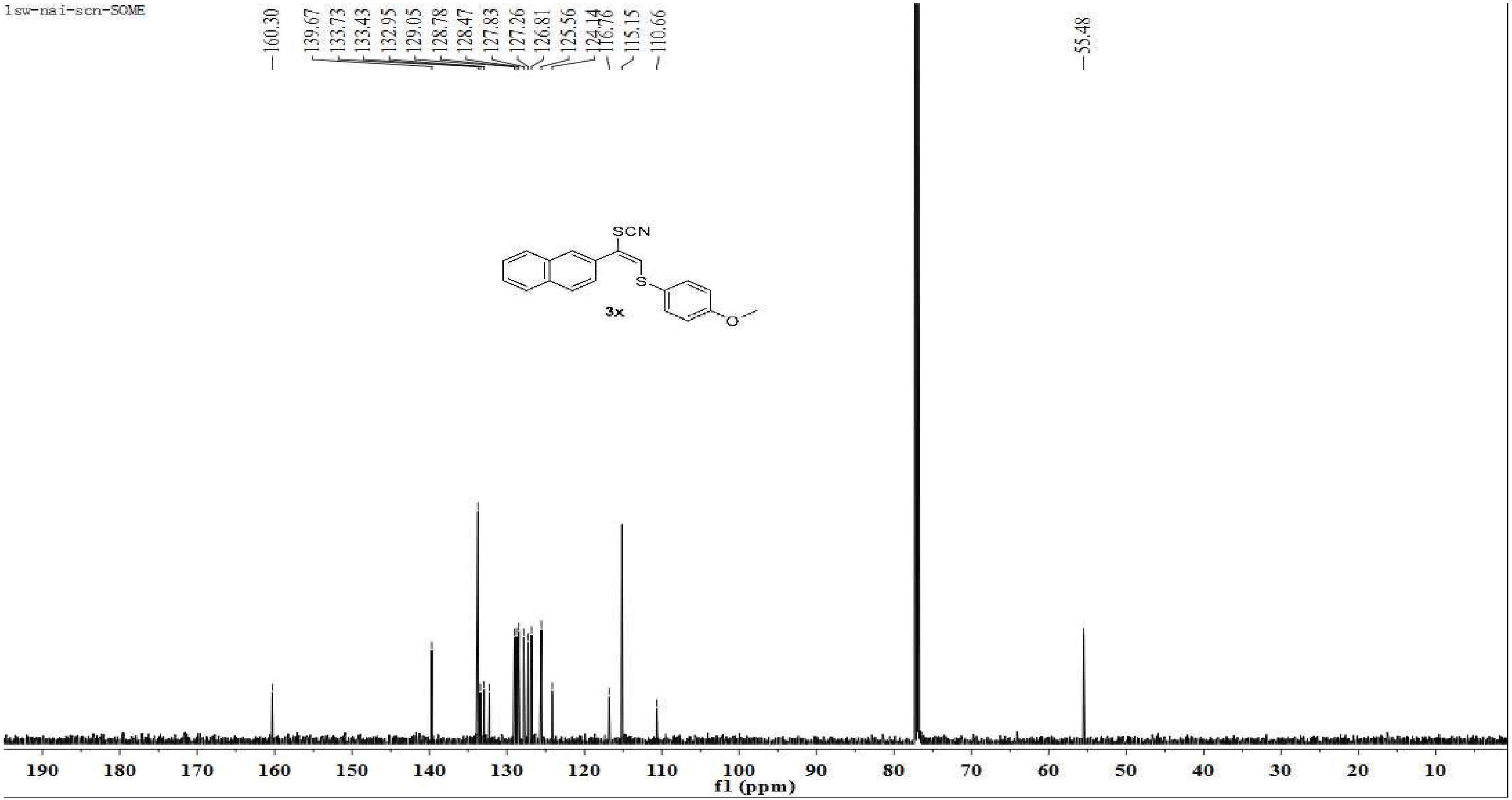

.50. ^{1}H NMR of **3y** (600 MHz, $CDCl_3$)

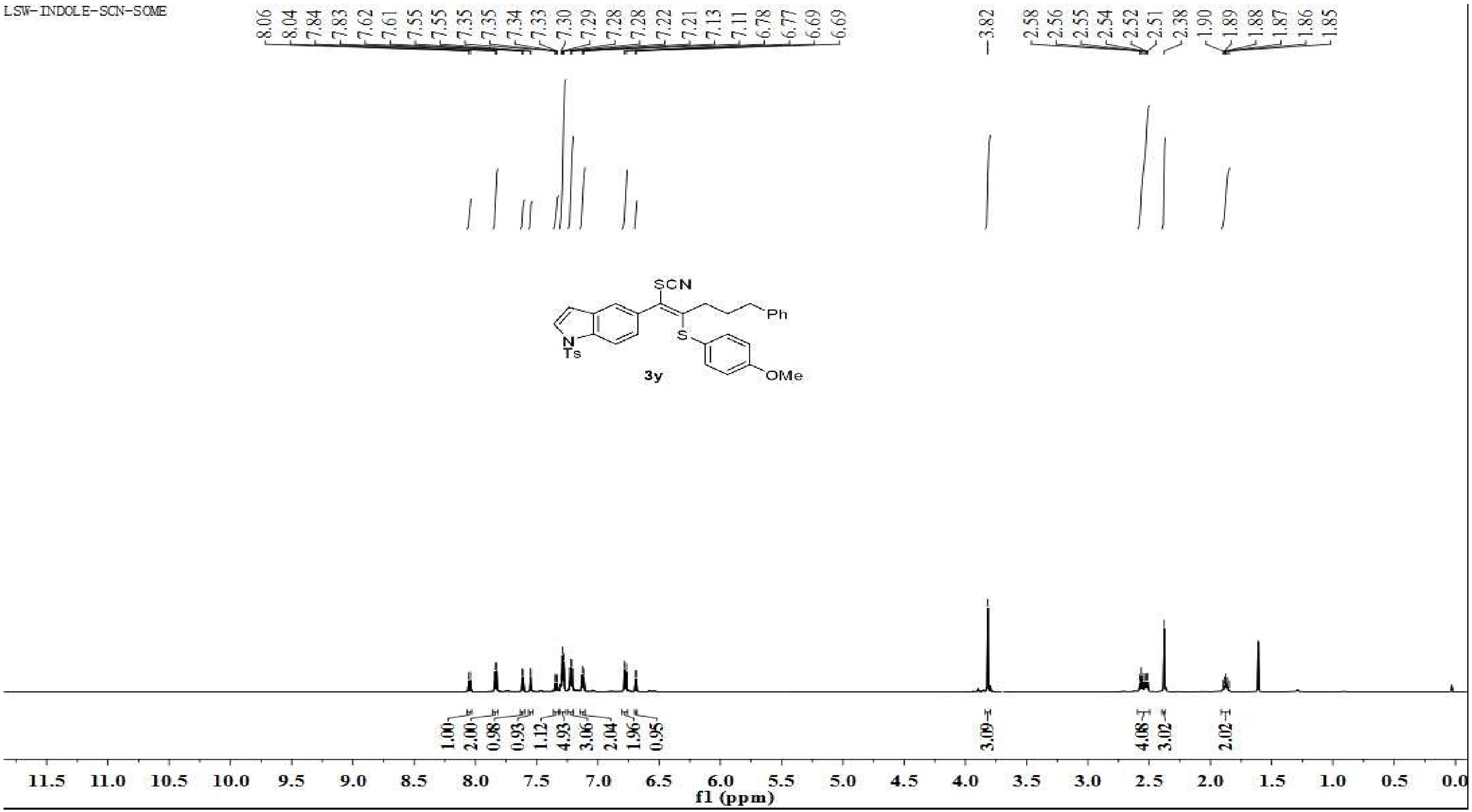

51.^{13}C NMR of **3y** (125 MHz, $CDCl_3$)

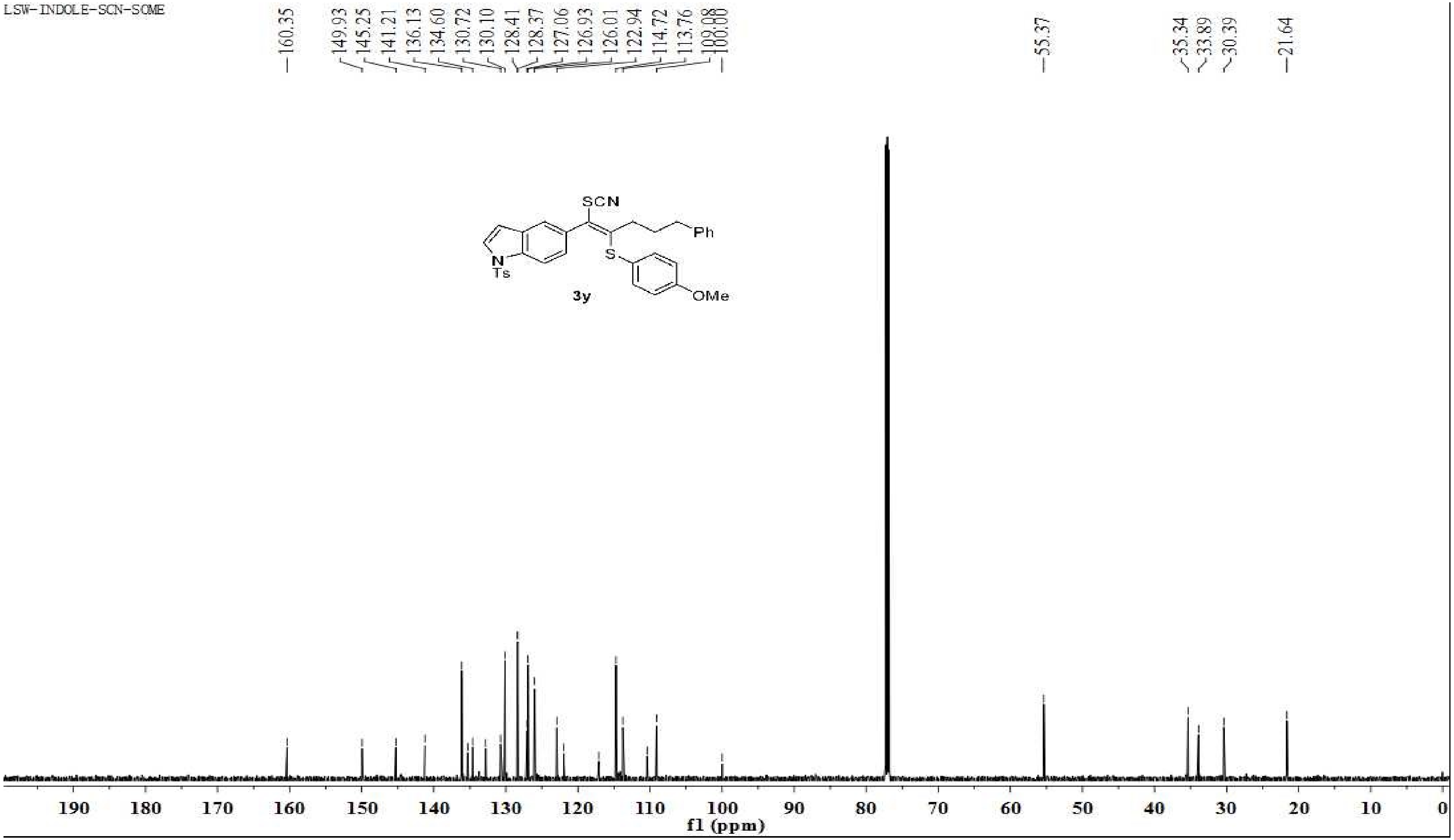

52. ^{1}H NMR of **3z** (600 MHz, $CDCl_3$)

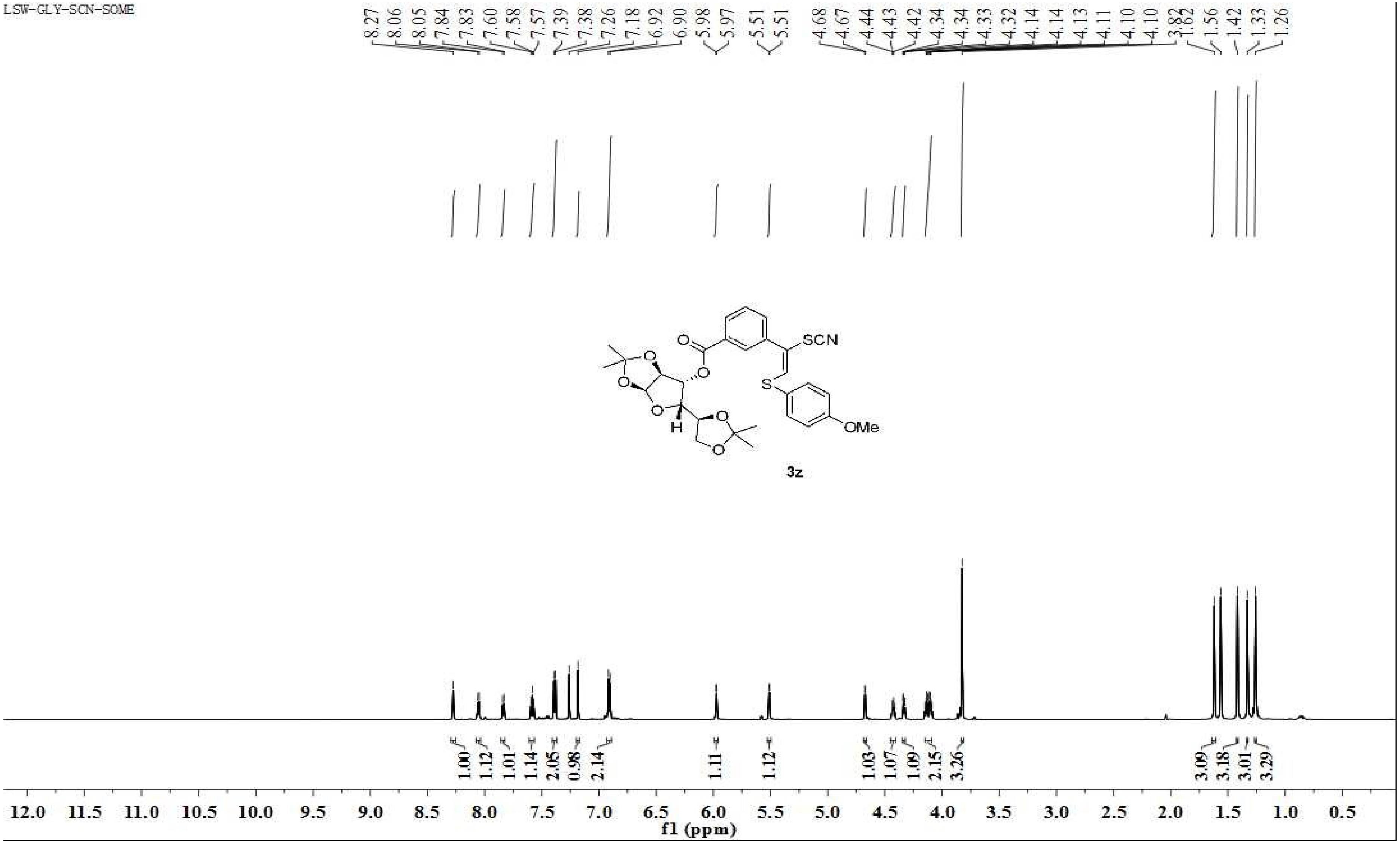

53. ^{13}C NMR of **3z** (125 MHz, $CDCl_3$)

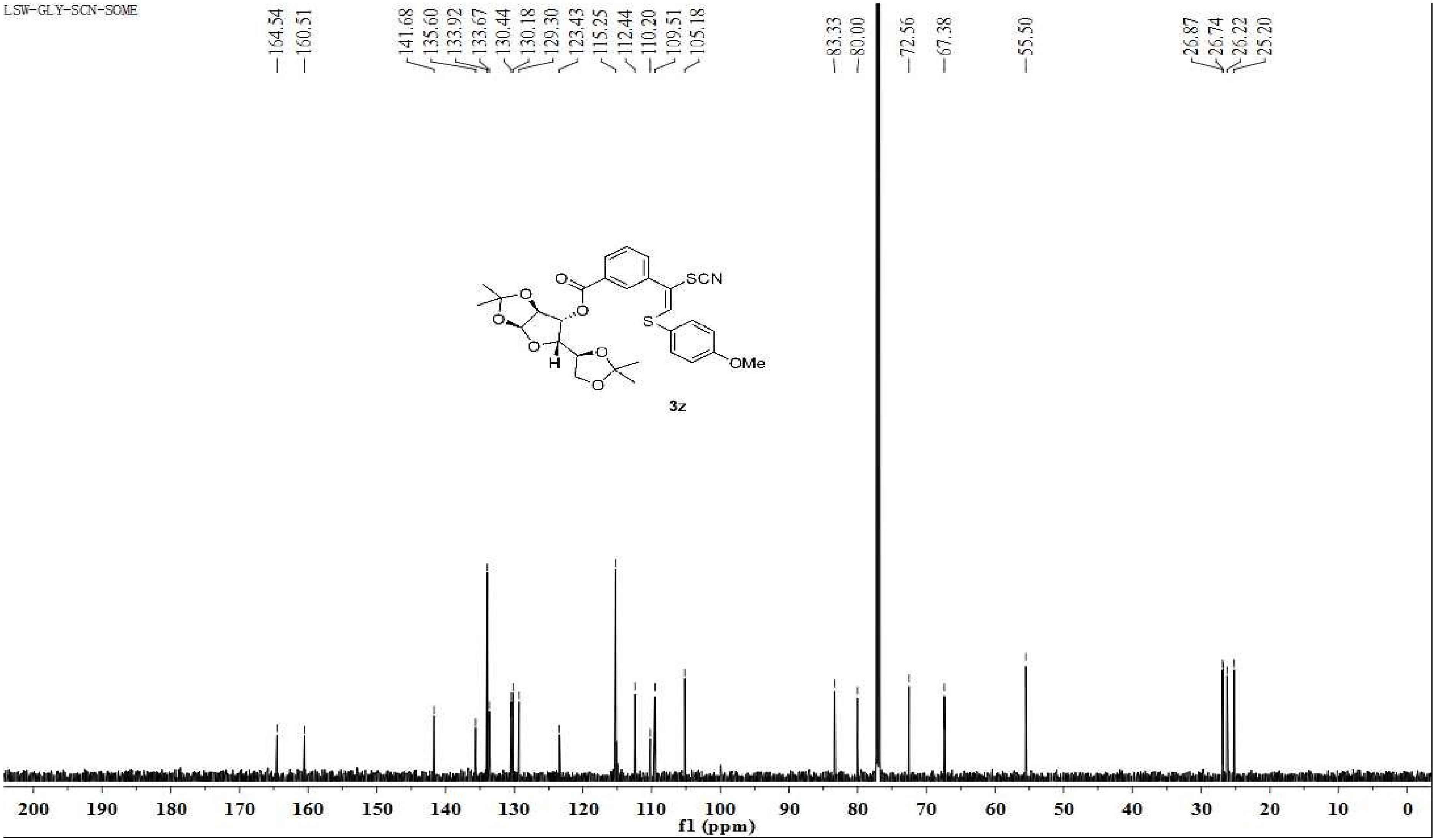

54. ^{1}H NMR of **3aa** (600 MHz, $CDCl_3$)

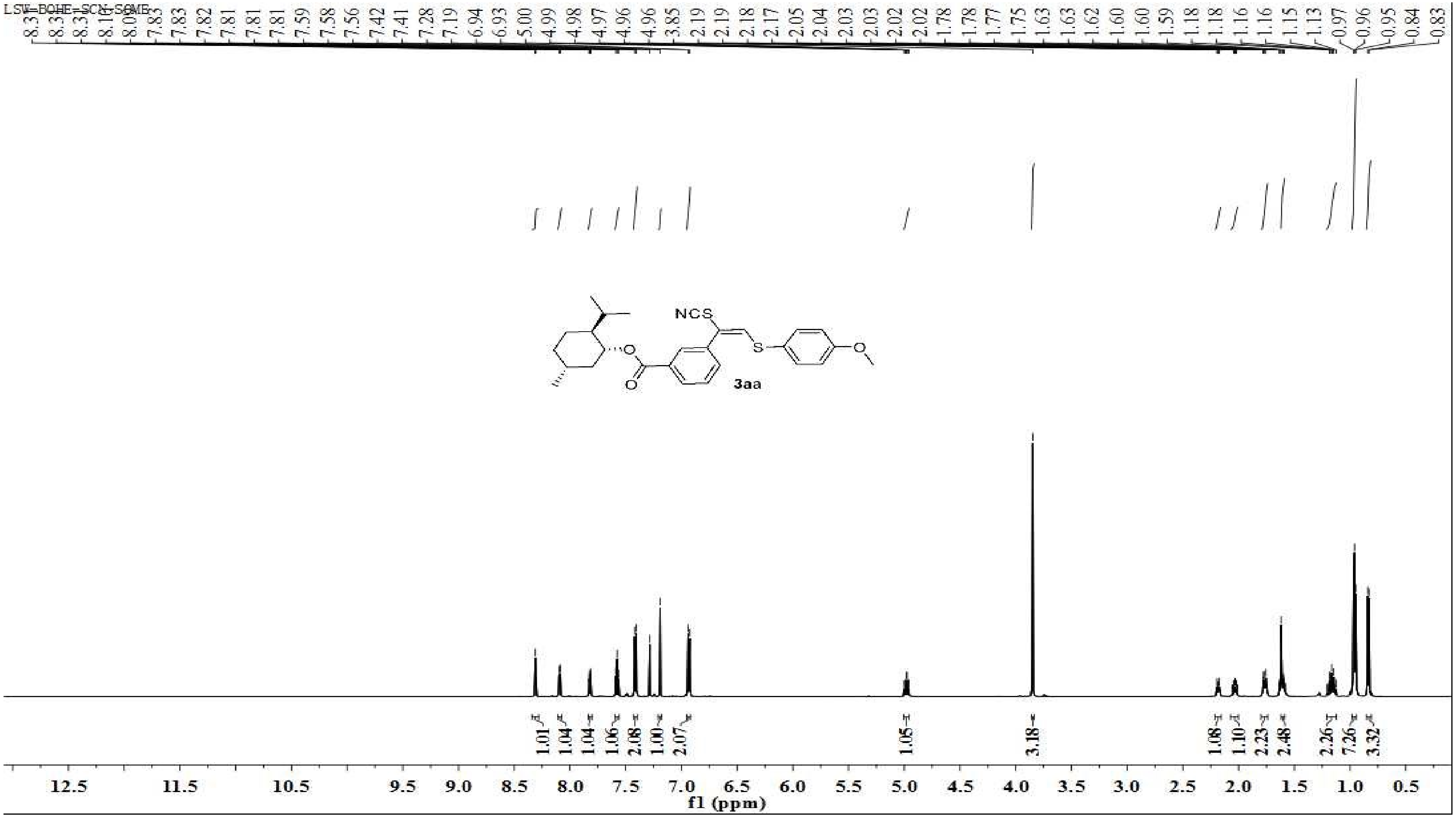

.55.^{13}C NMR of **3aa** (125 MHz, $CDCl_3$)

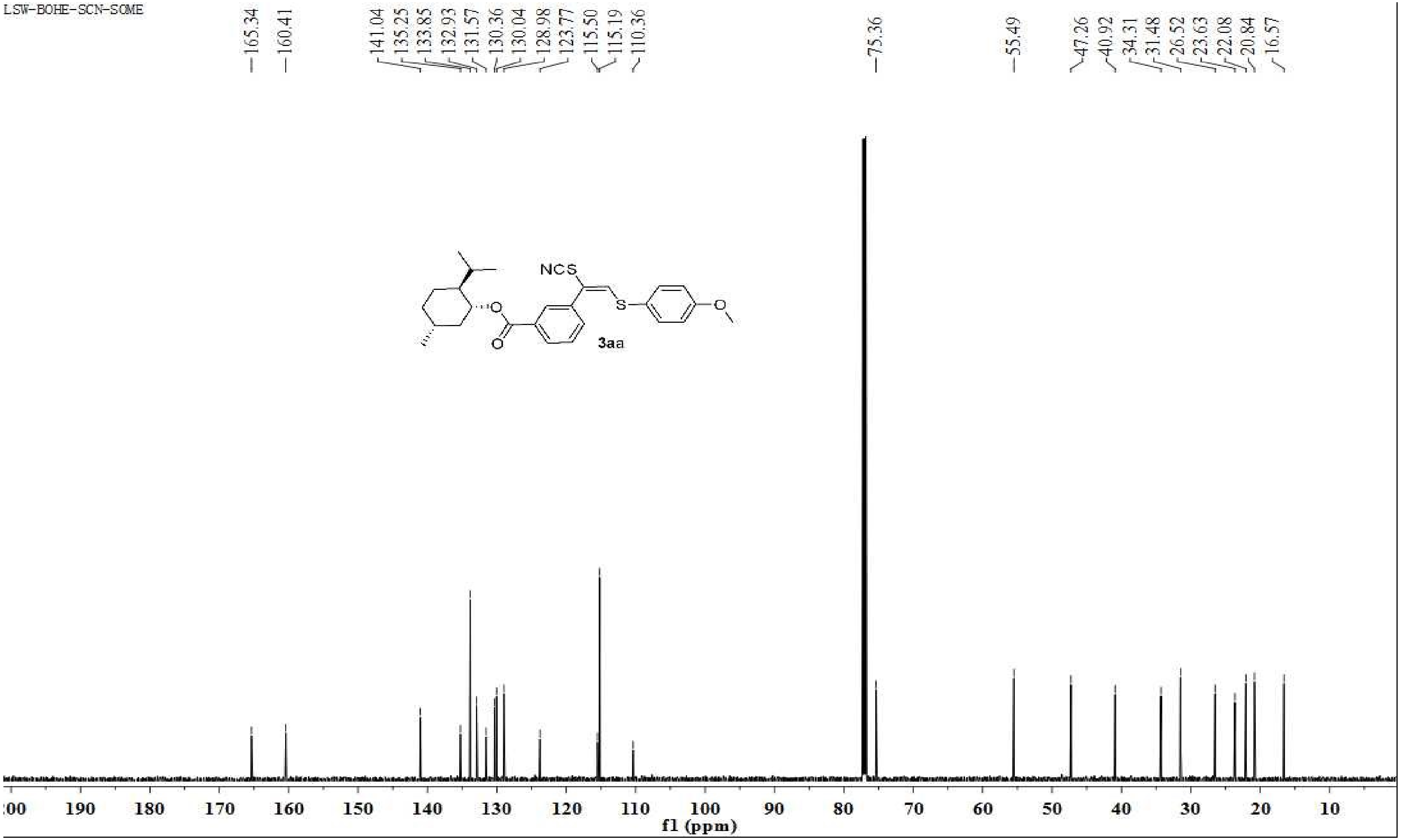

56. ^{1}H NMR of **3ab** (600 MHz, $CDCl_3$)

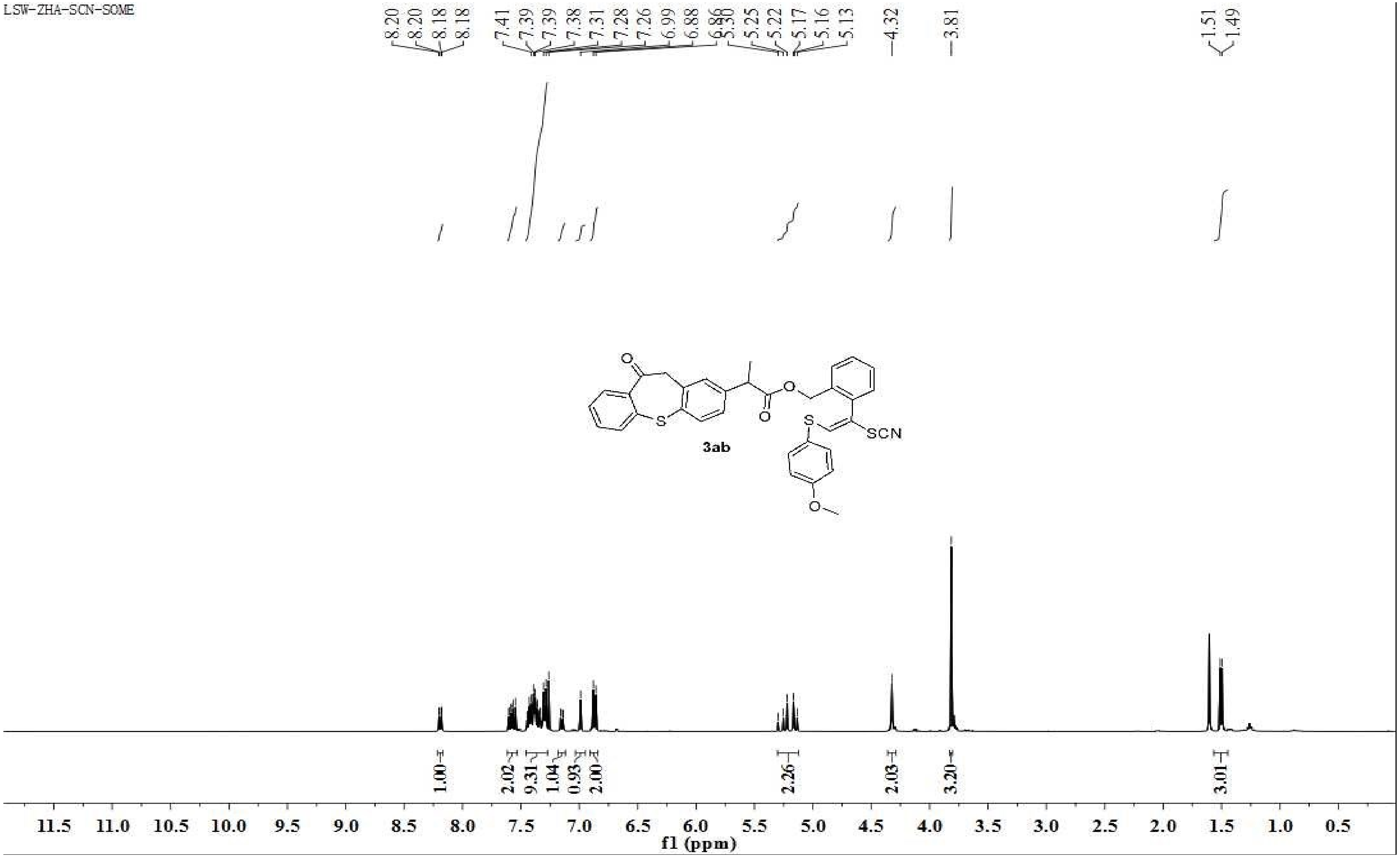

57. ^{13}C NMR of **3ab** (125 MHz, $CDCl_3$)

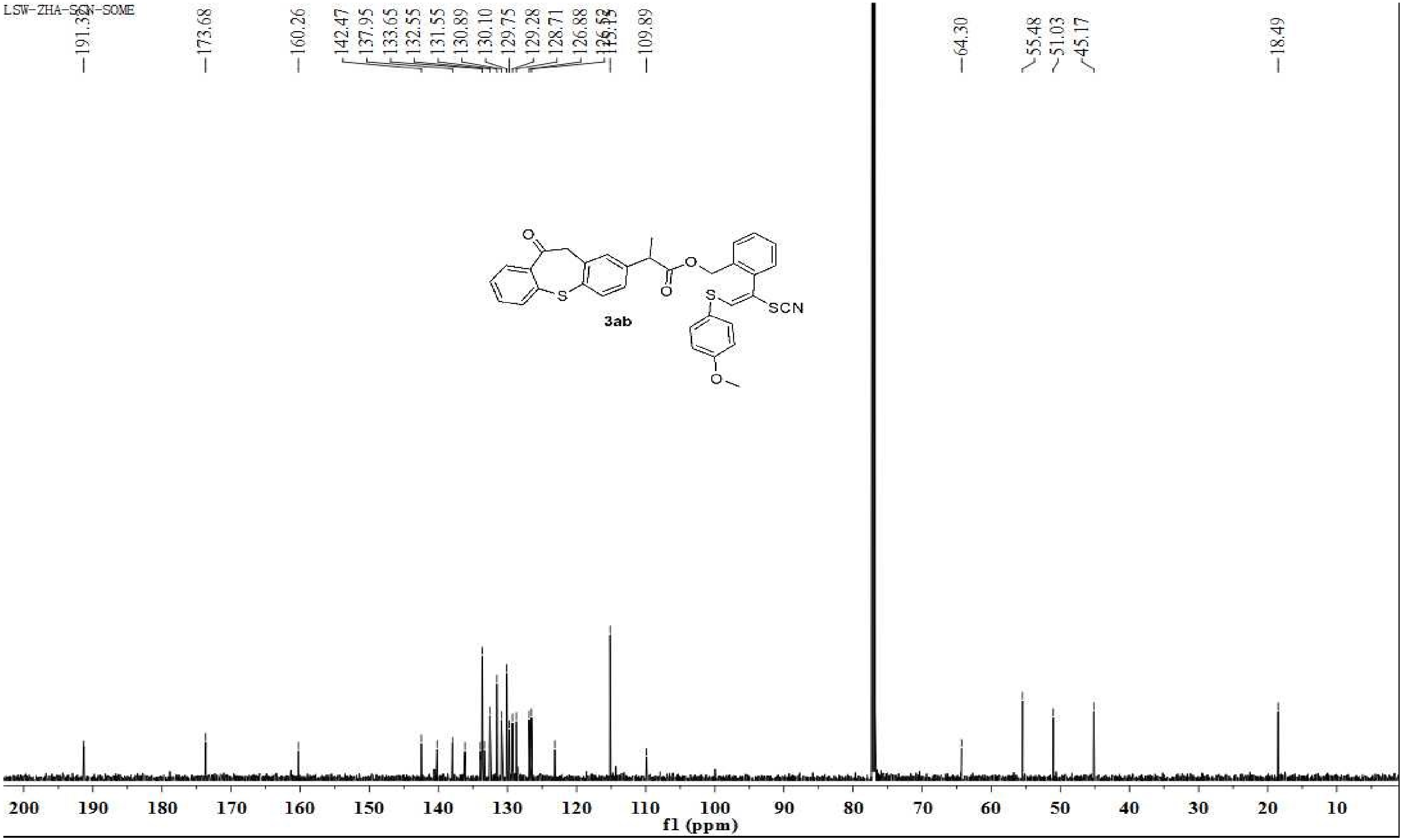

58. ^{1}H NMR of **3ac** (600 MHz, $CDCl_3$)

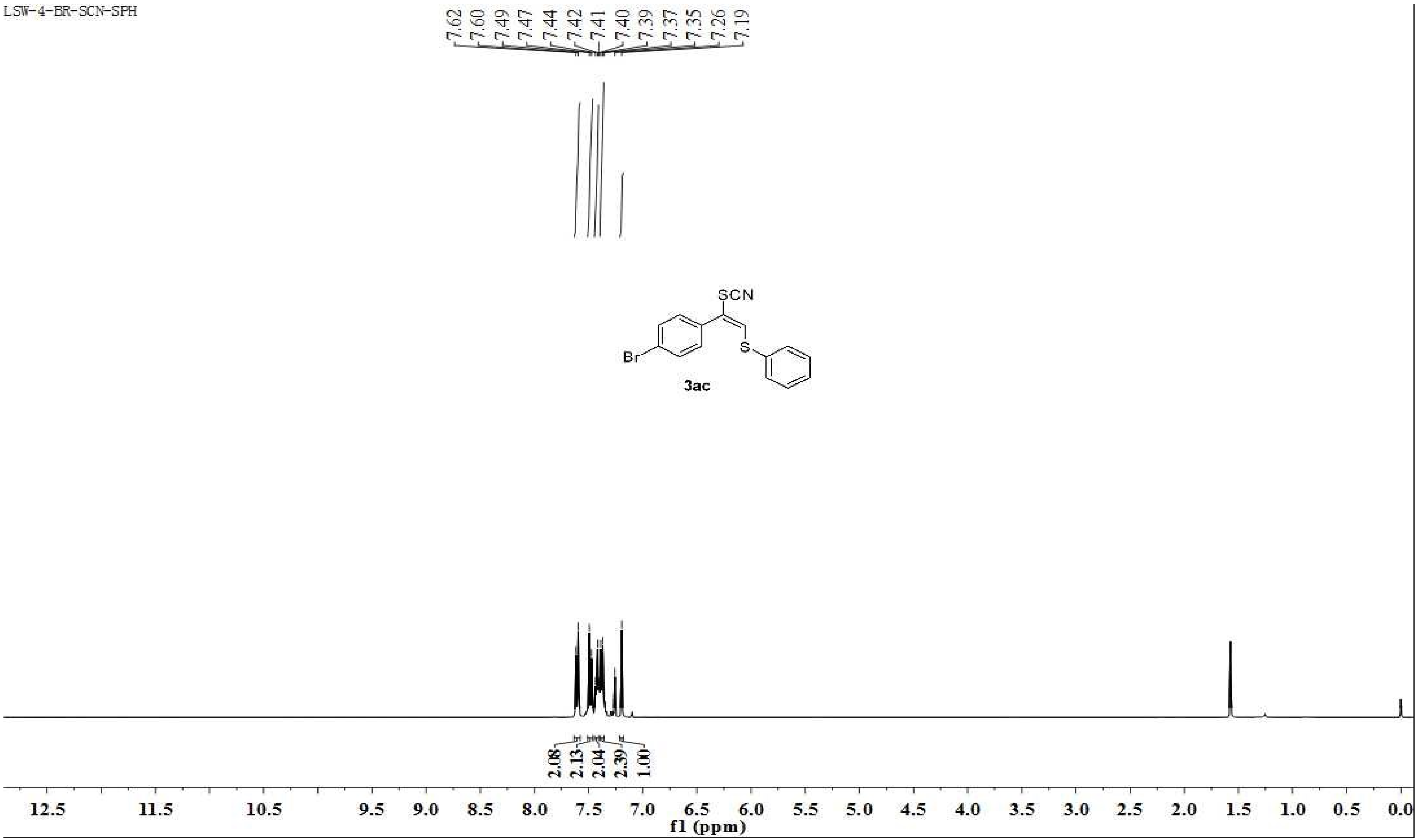

59. ^{1}H NMR of **3z** (600 MHz, $CDCl_3$)

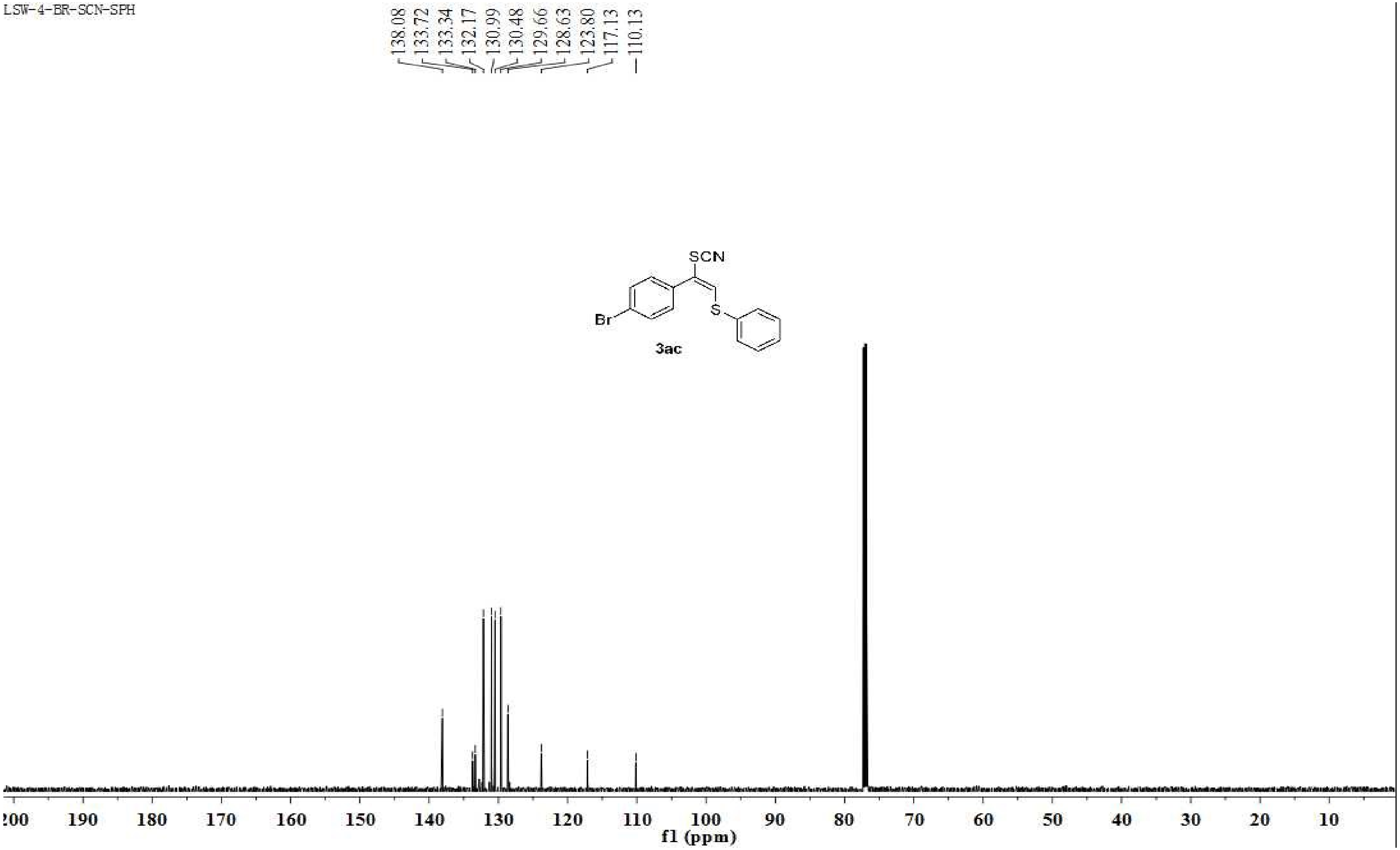

60. ^{1}H NMR of **3ad** (600 MHz, $CDCl_3$)

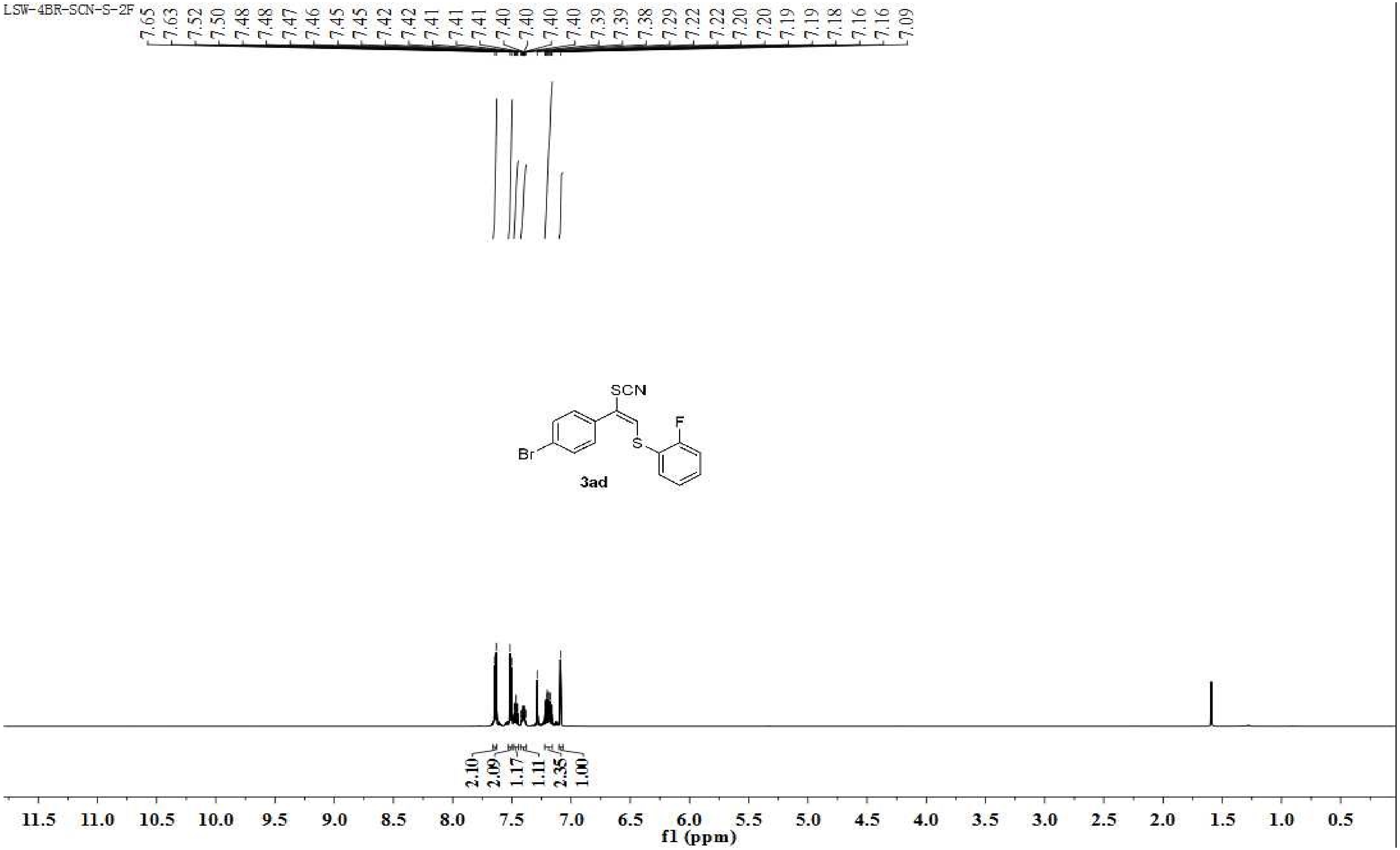

61. ^{13}C NMR of **3ad** (125 MHz, $CDCl_3$)

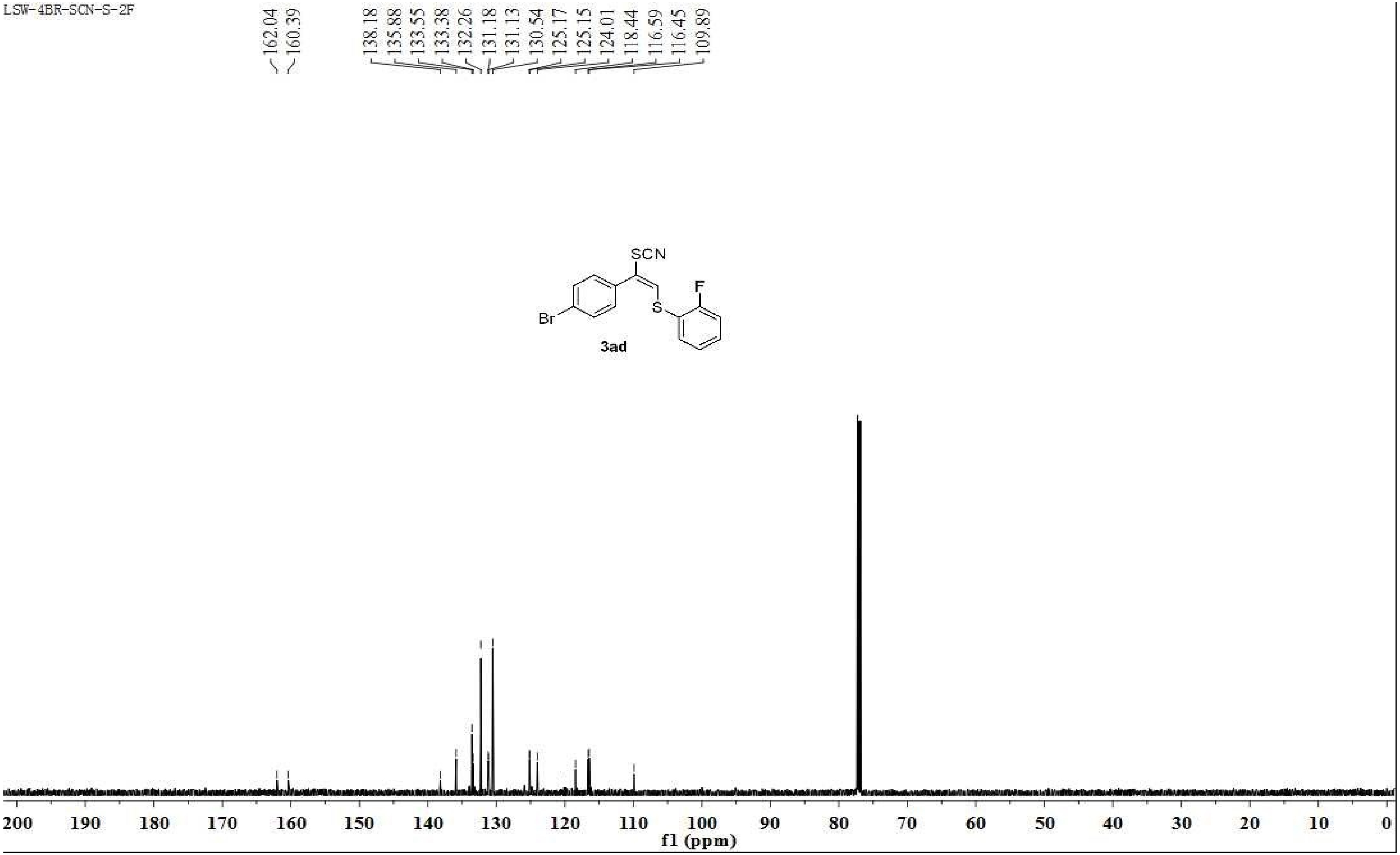

62. ¹H NMR of **3ae** (600 MHz, $CDCl_3$)

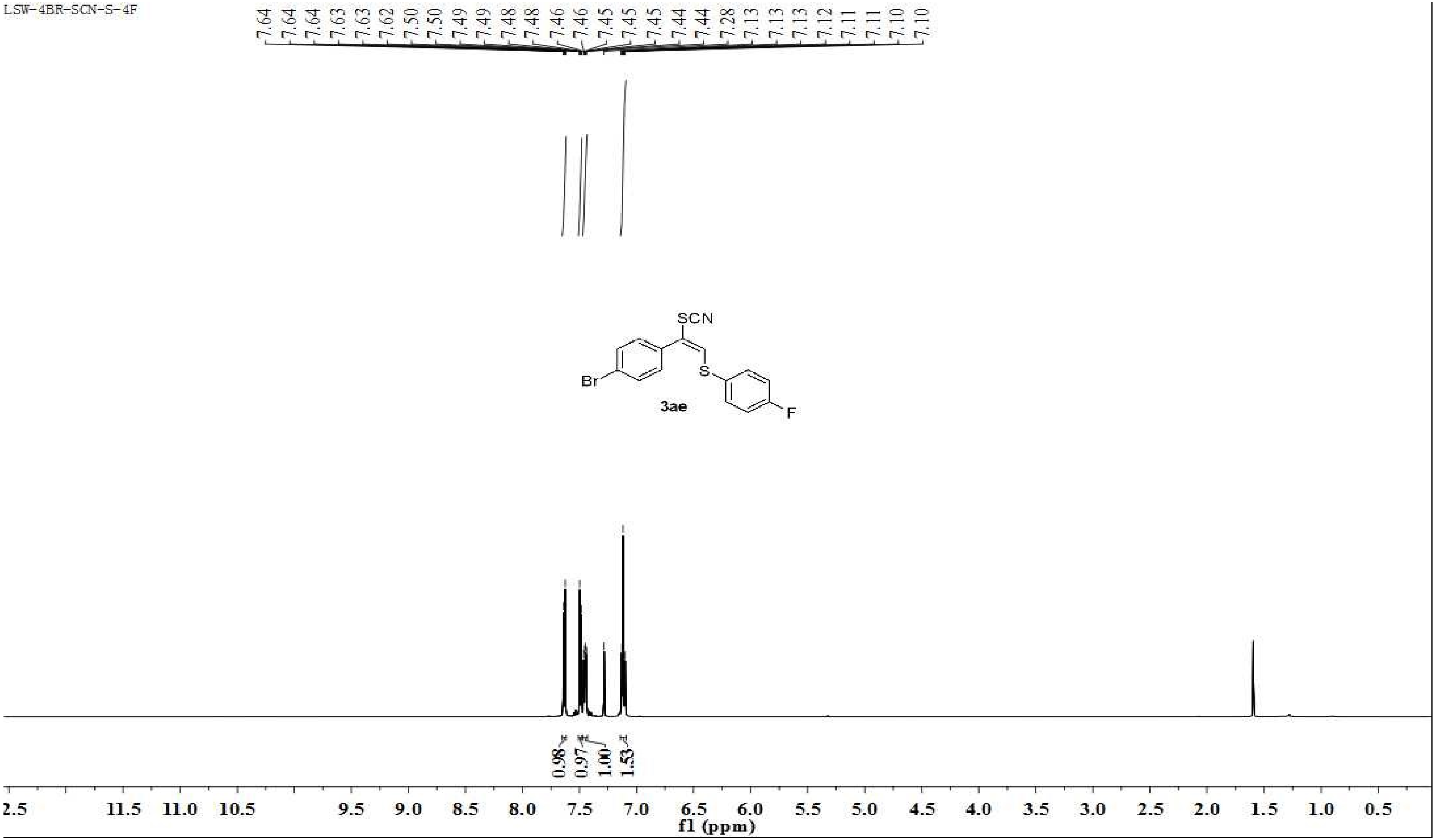

63. ^{13}C NMR of **3ae** (125 MHz, $CDCl_3$)

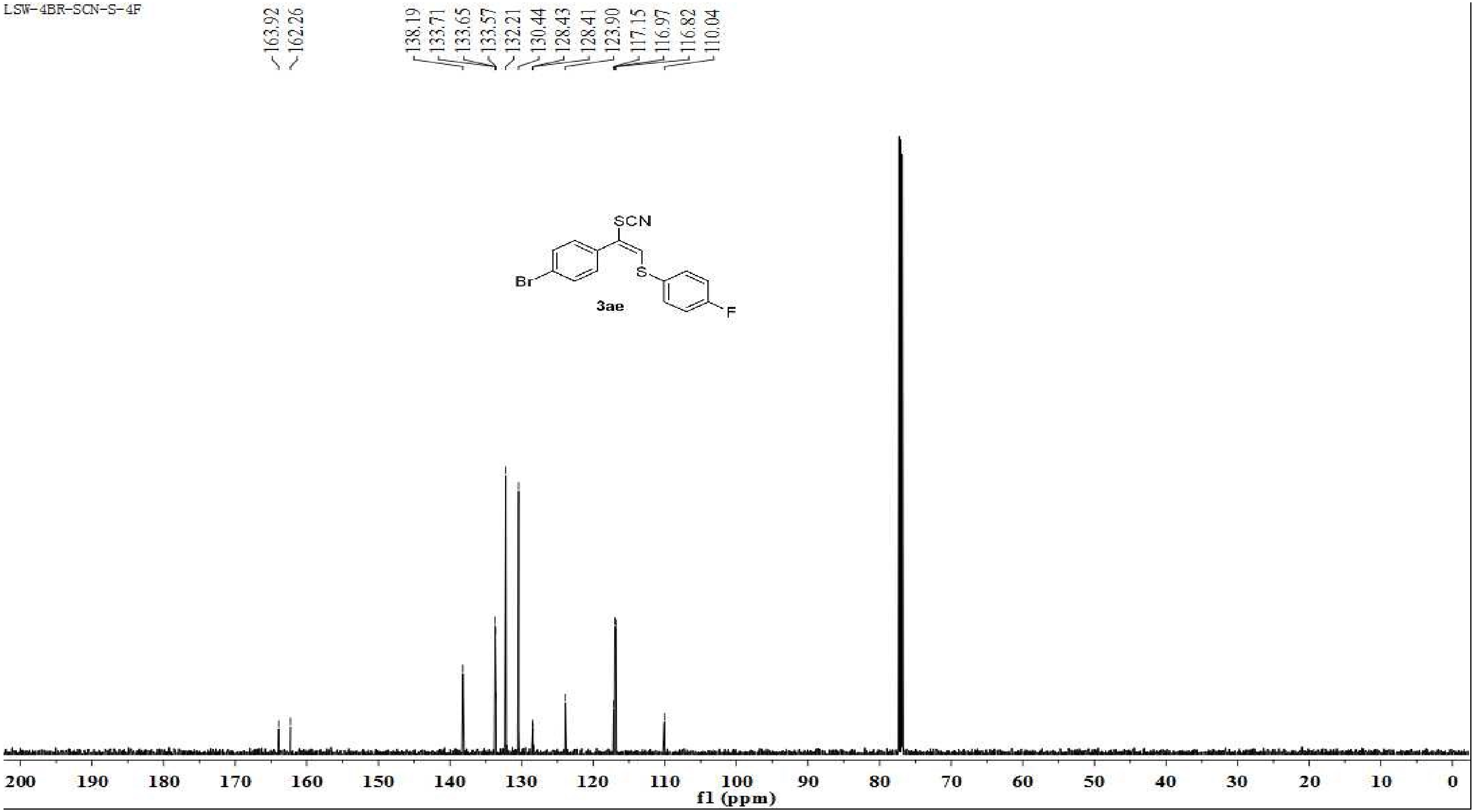

.64. ^{1}H NMR of **3af** (600 MHz, $CDCl_3$)

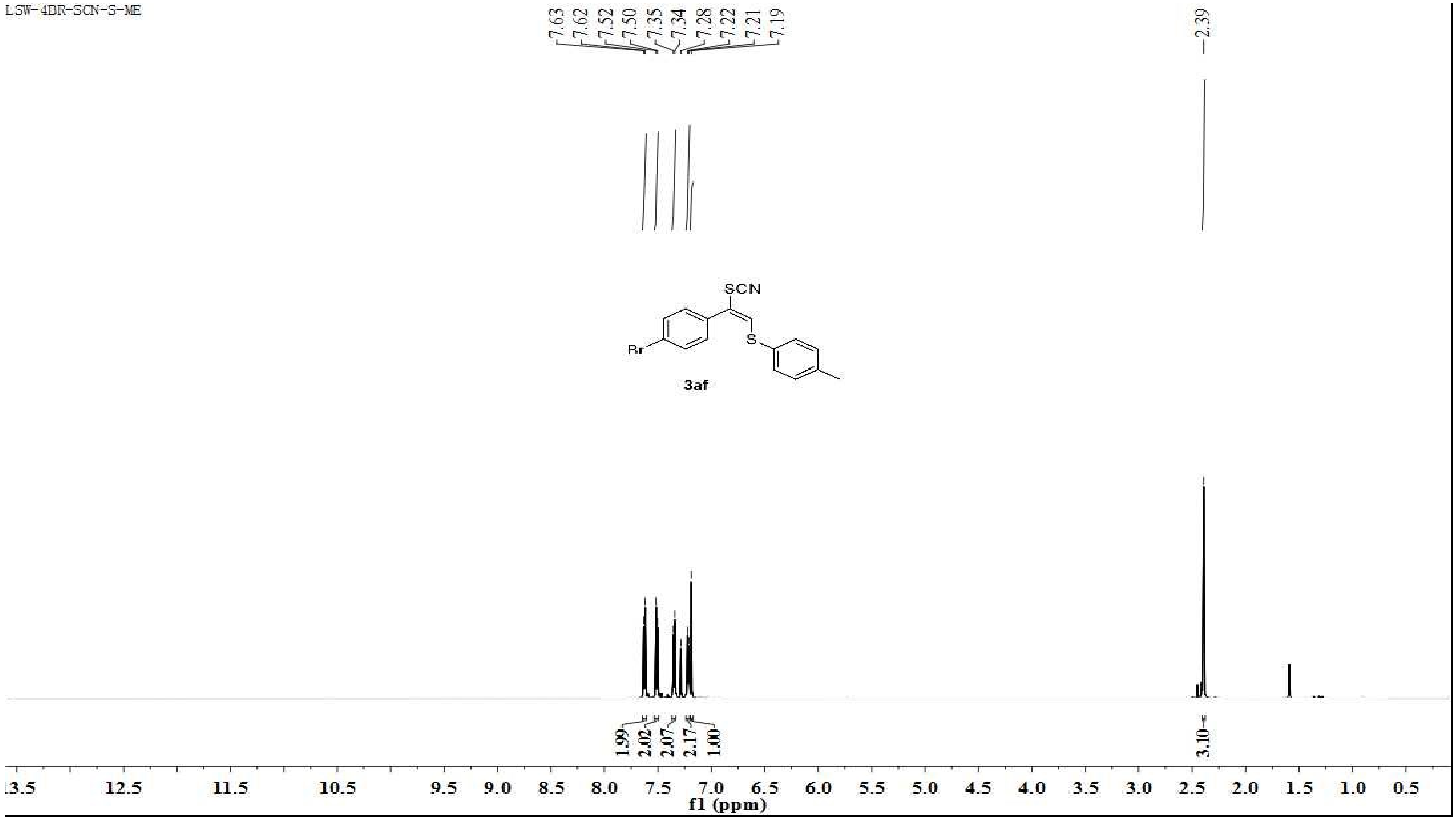

.65.^{13}C NMR of **3af** (125 MHz, $CDCl_3$)

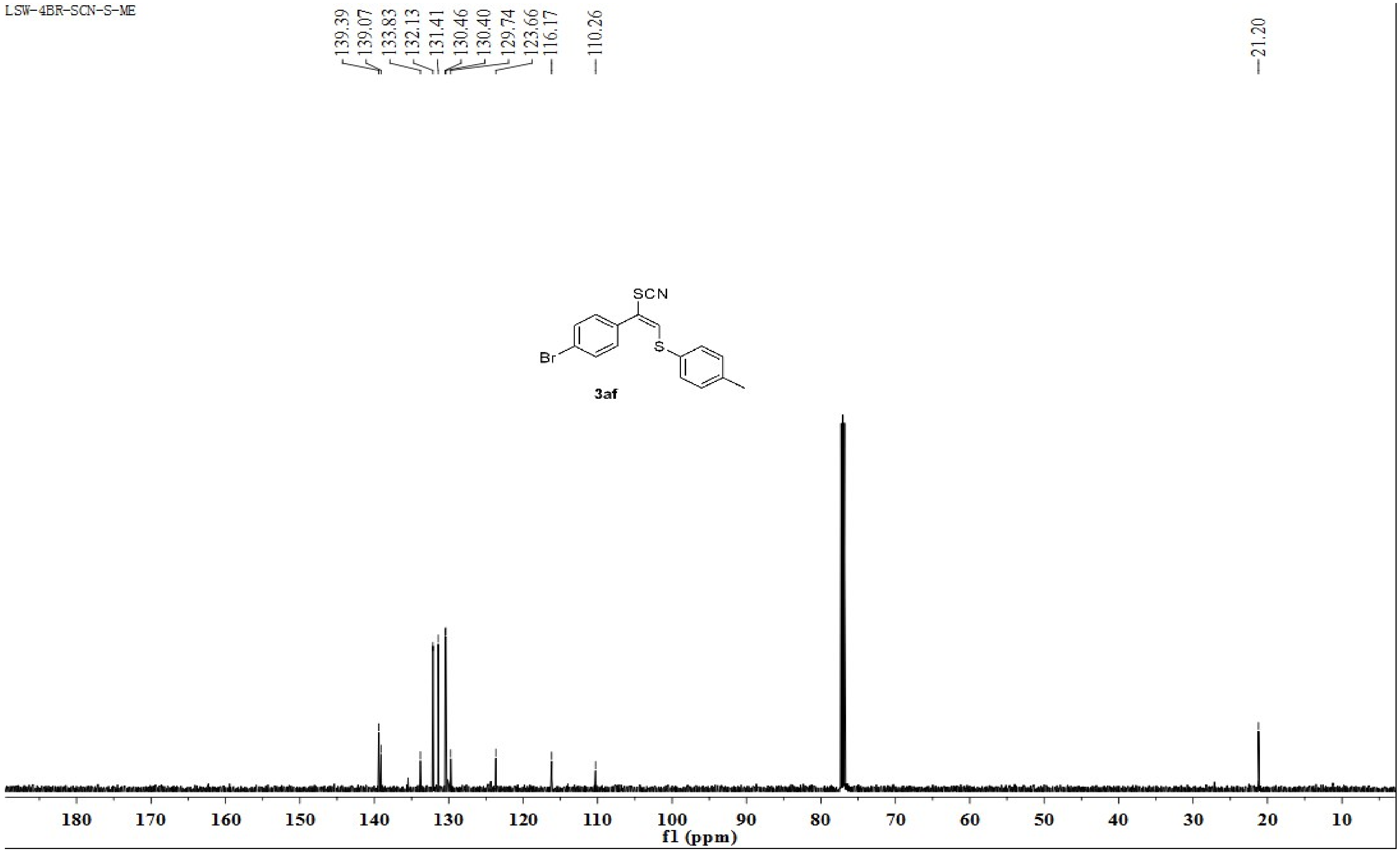

66. ^{1}H NMR of **3ag** (600 MHz, $CDCl_3$)

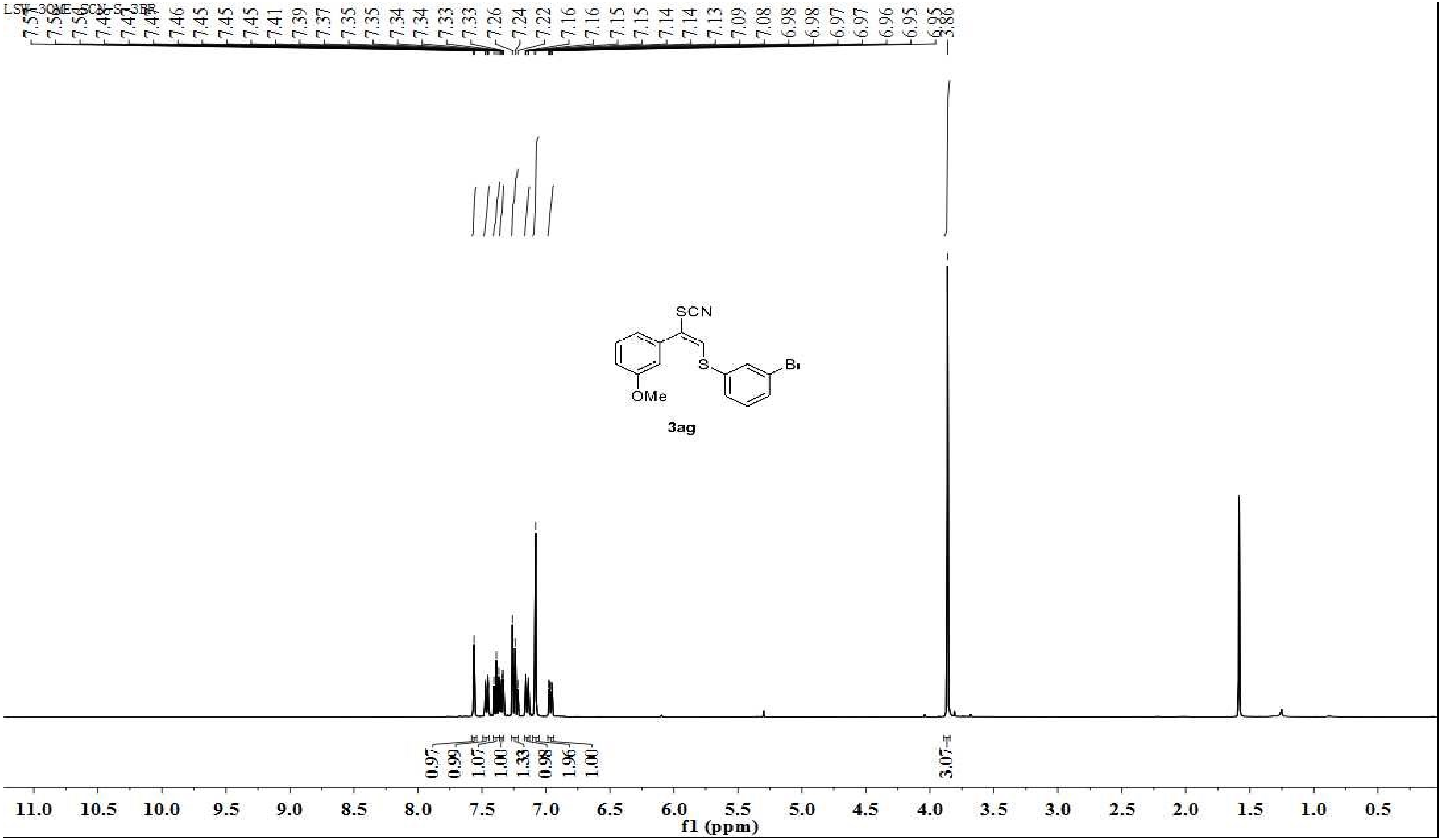

67. ^{13}C NMR of **3ag** (125 MHz, $CDCl_3$)

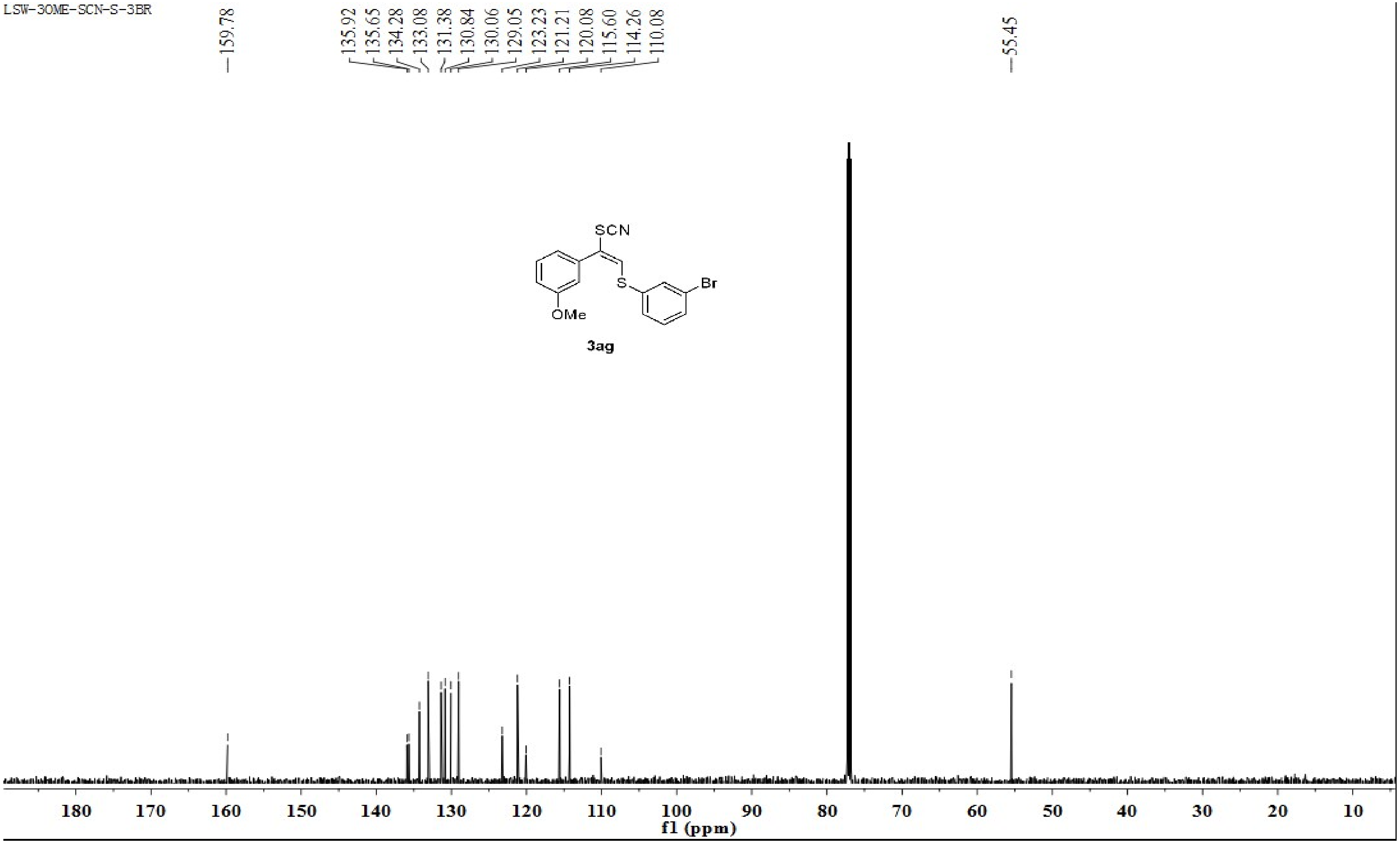

68. ^{1}H NMR of **3ah** (600 MHz, $CDCl_3$)

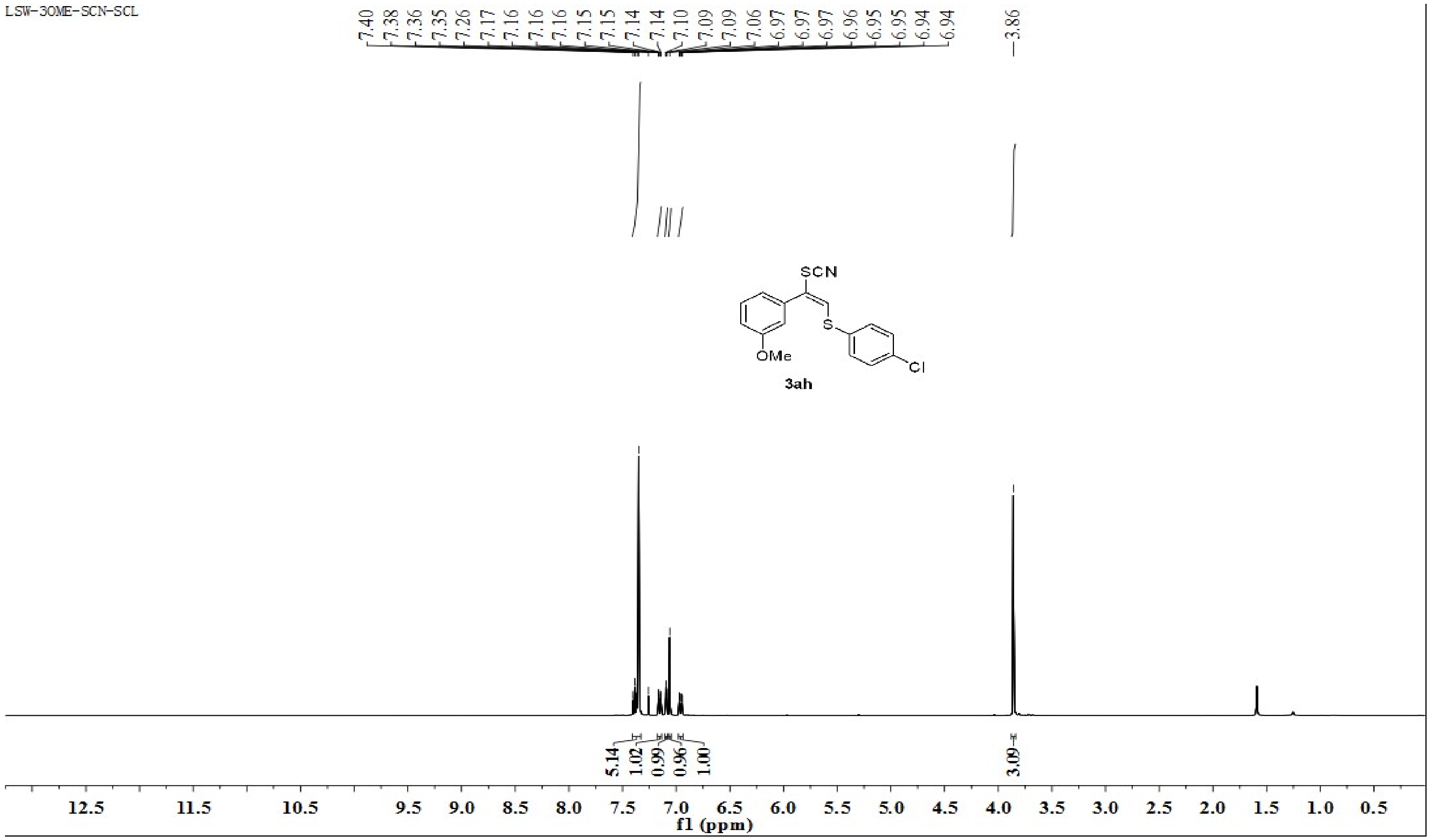

69. ^{13}C NMR of **3ah** (125 MHz, $CDCl_3$)

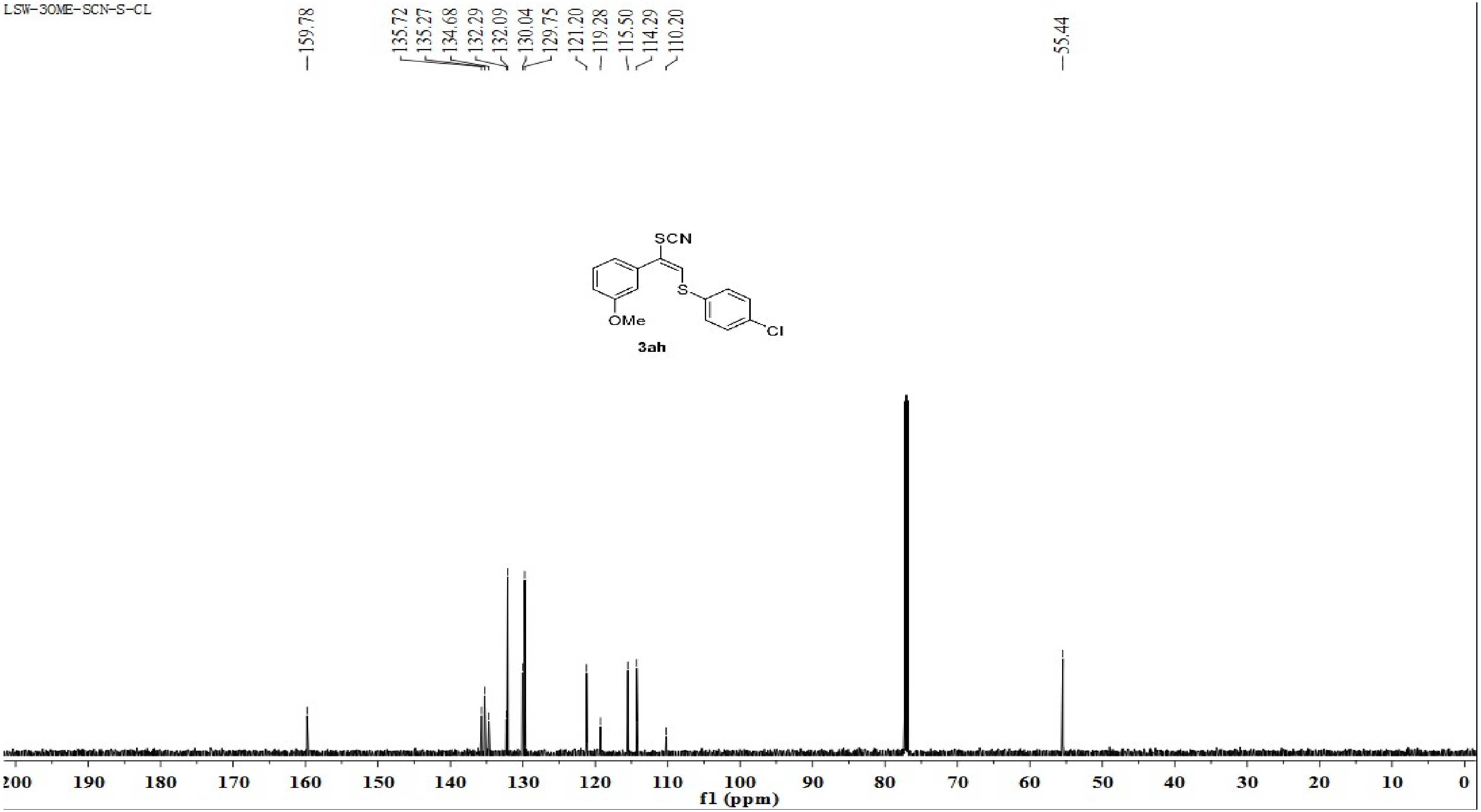

70. ^{1}H NMR of **3ai** (600 MHz, $CDCl_3$)

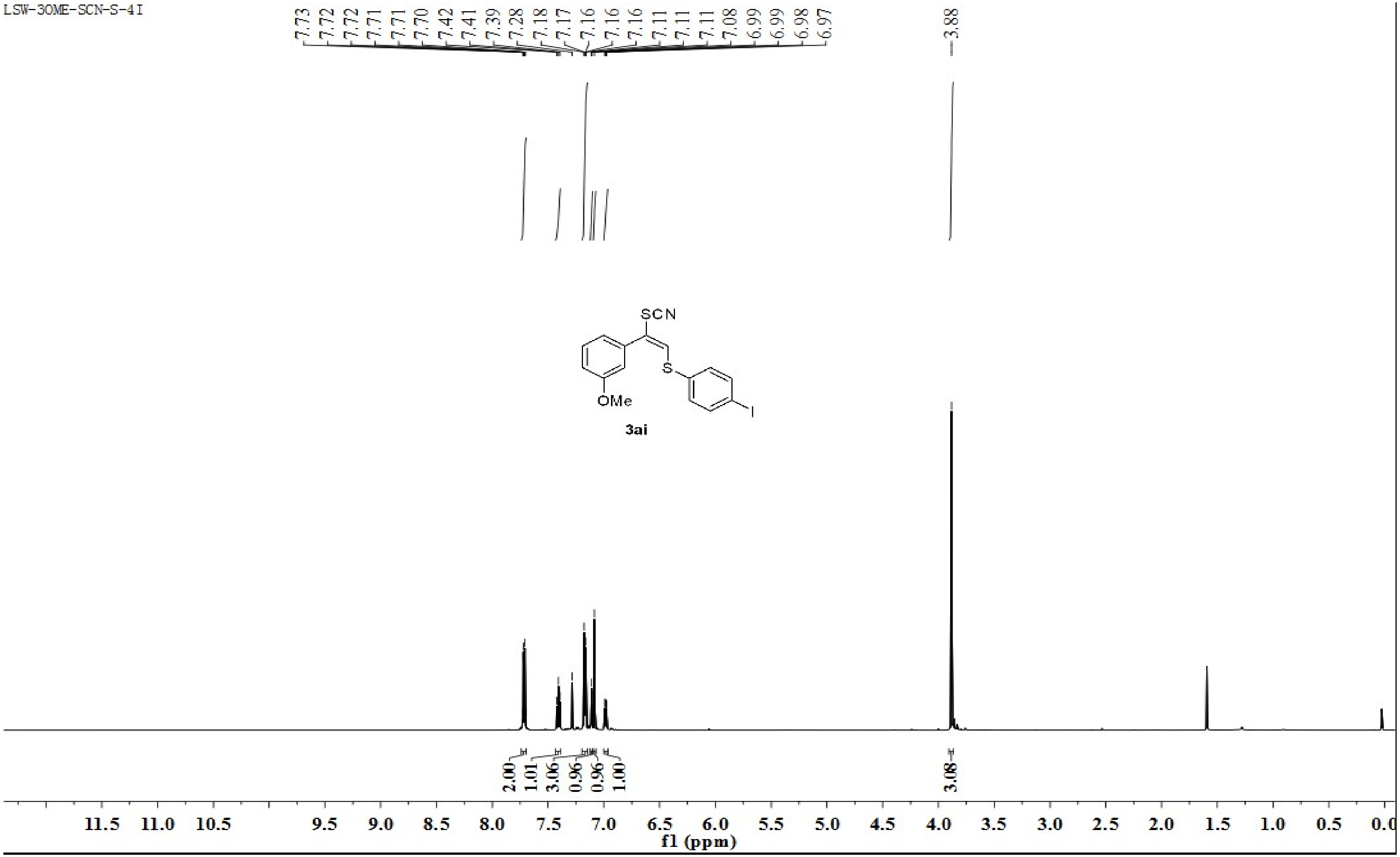

71. ^{13}C NMR of **3ai** (125 MHz, $CDCl_3$)

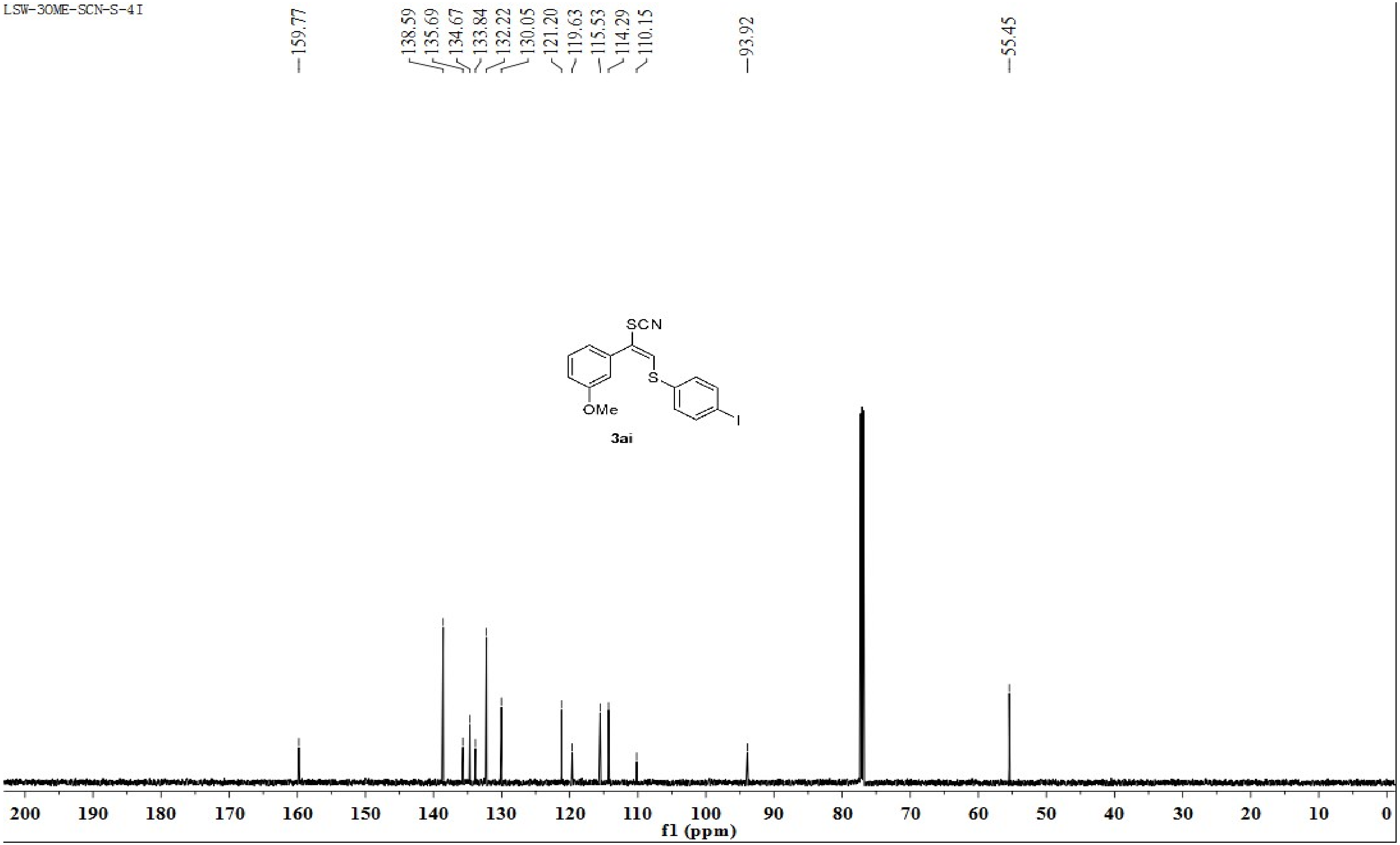

72. ^{1}H NMR of **3aj** (600 MHz, $CDCl_3$)

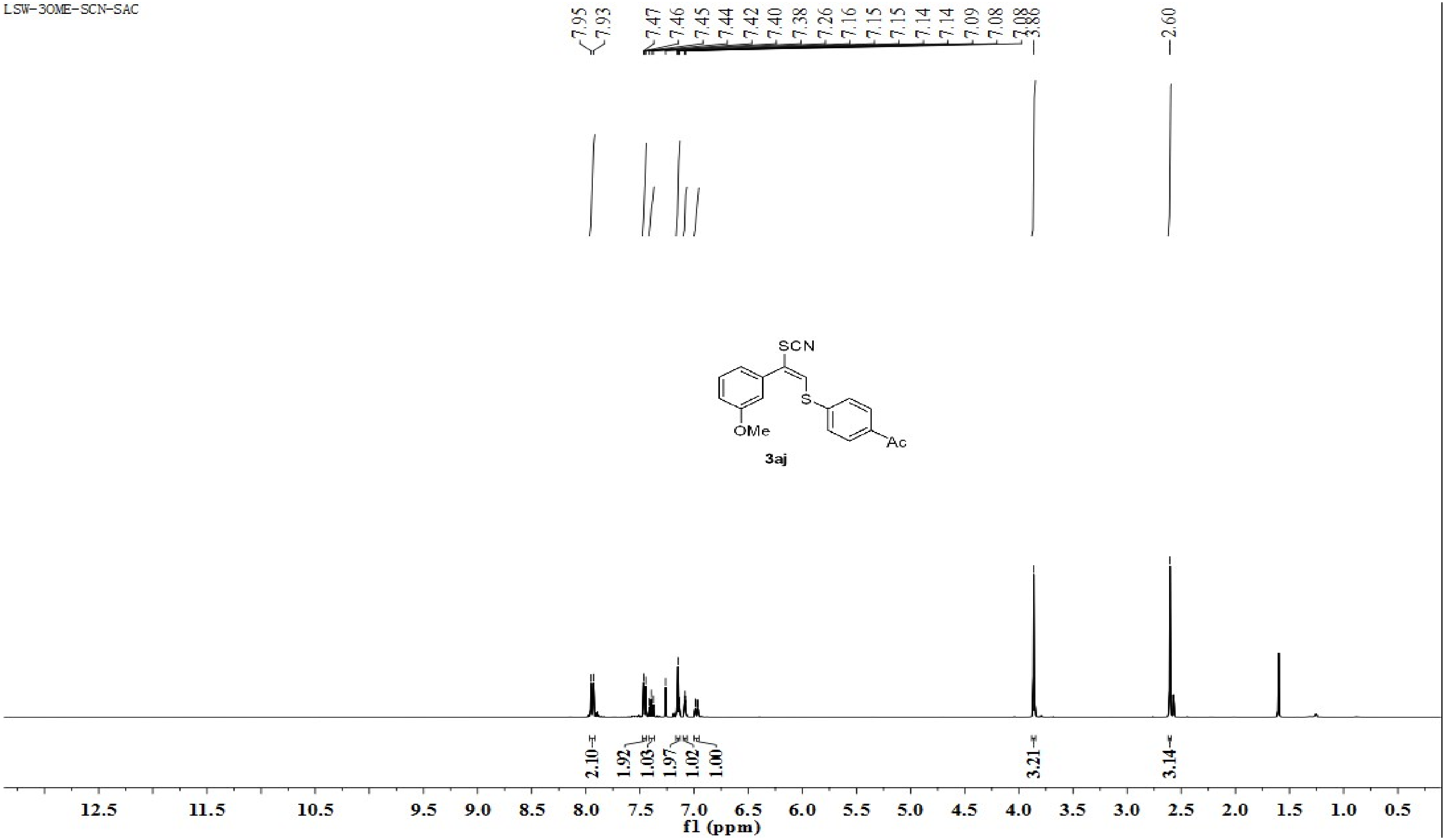

3aj

73. ^{13}C NMR of **3aj** (125 MHz, $CDCl_3$)

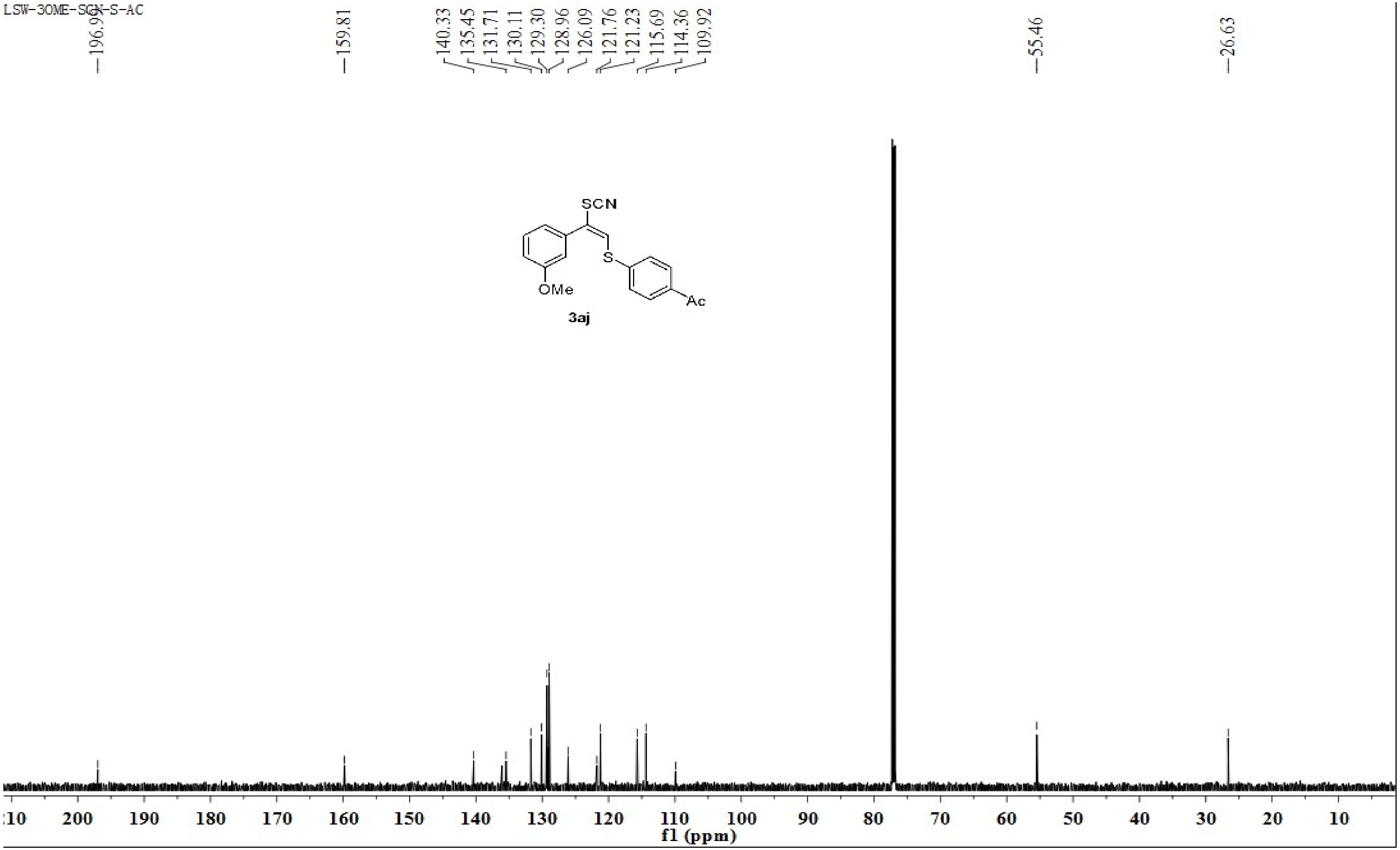

74. ^{1}H NMR of **3ak** (600 MHz, $CDCl_3$)

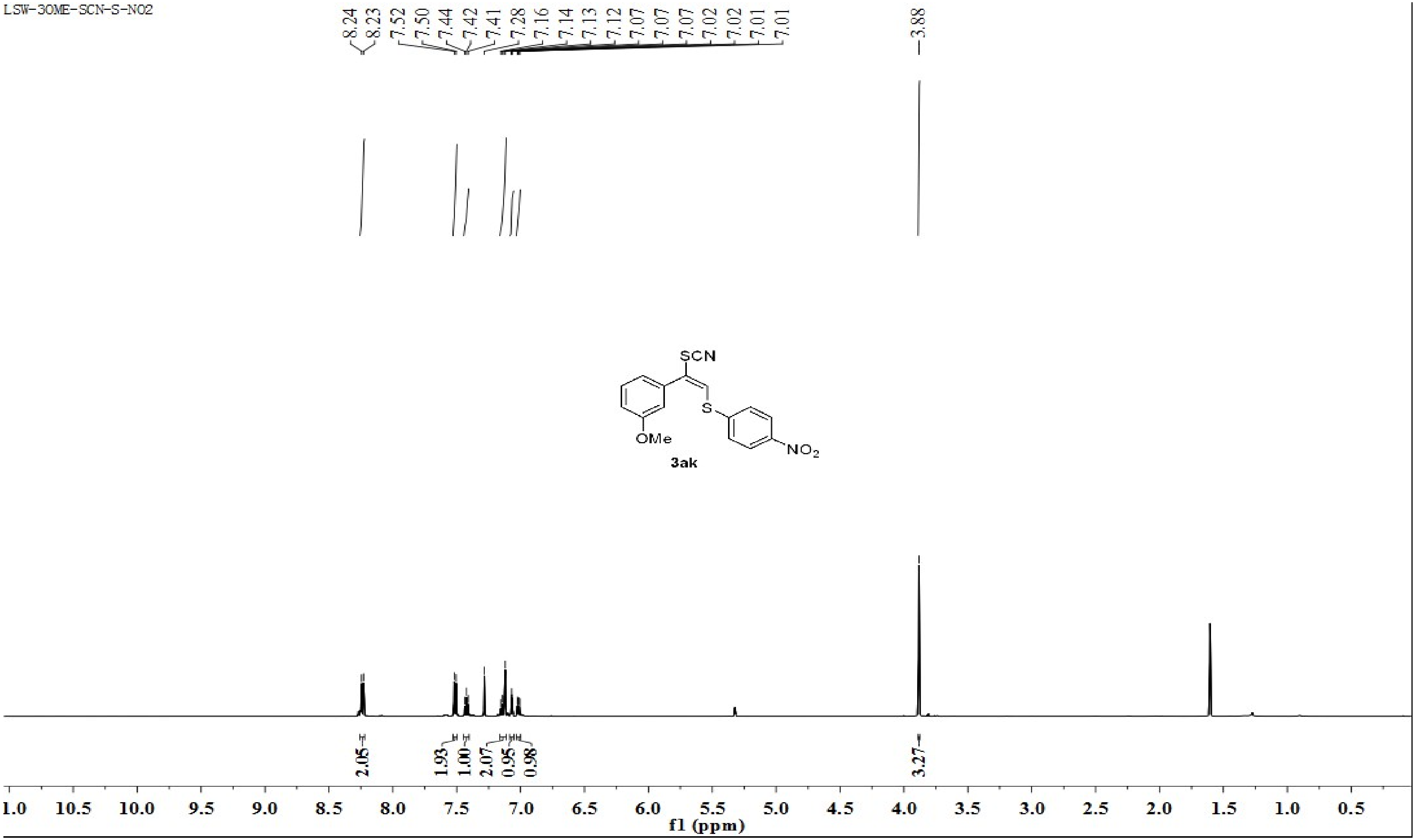

75. ^{13}C NMR of **3ak** (125 MHz, $CDCl_3$)

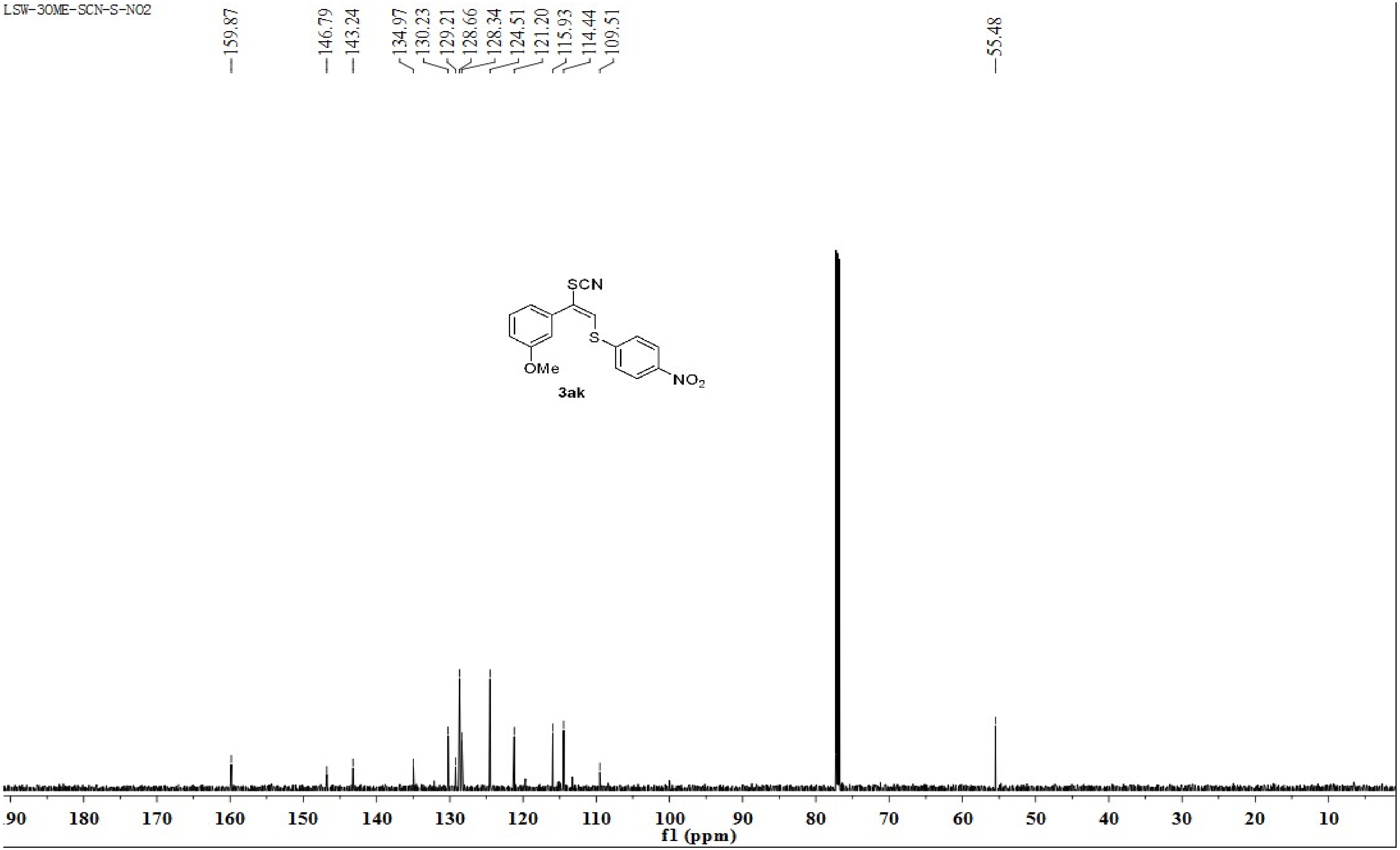

76. ^{1}H NMR of **3al** (600 MHz, $CDCl_3$)

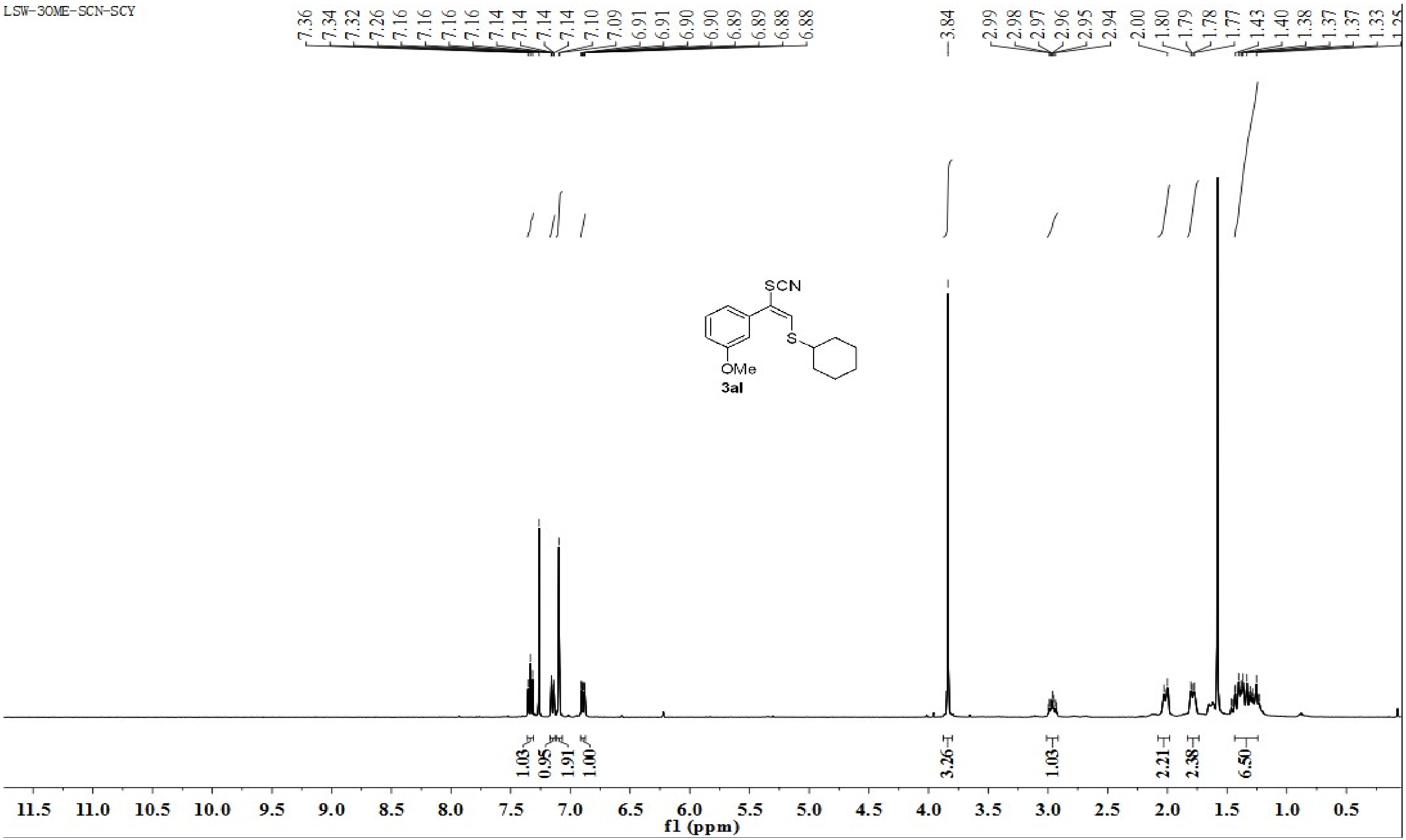

77. ^{13}C NMR of **3al** (125 MHz, $CDCl_3$)

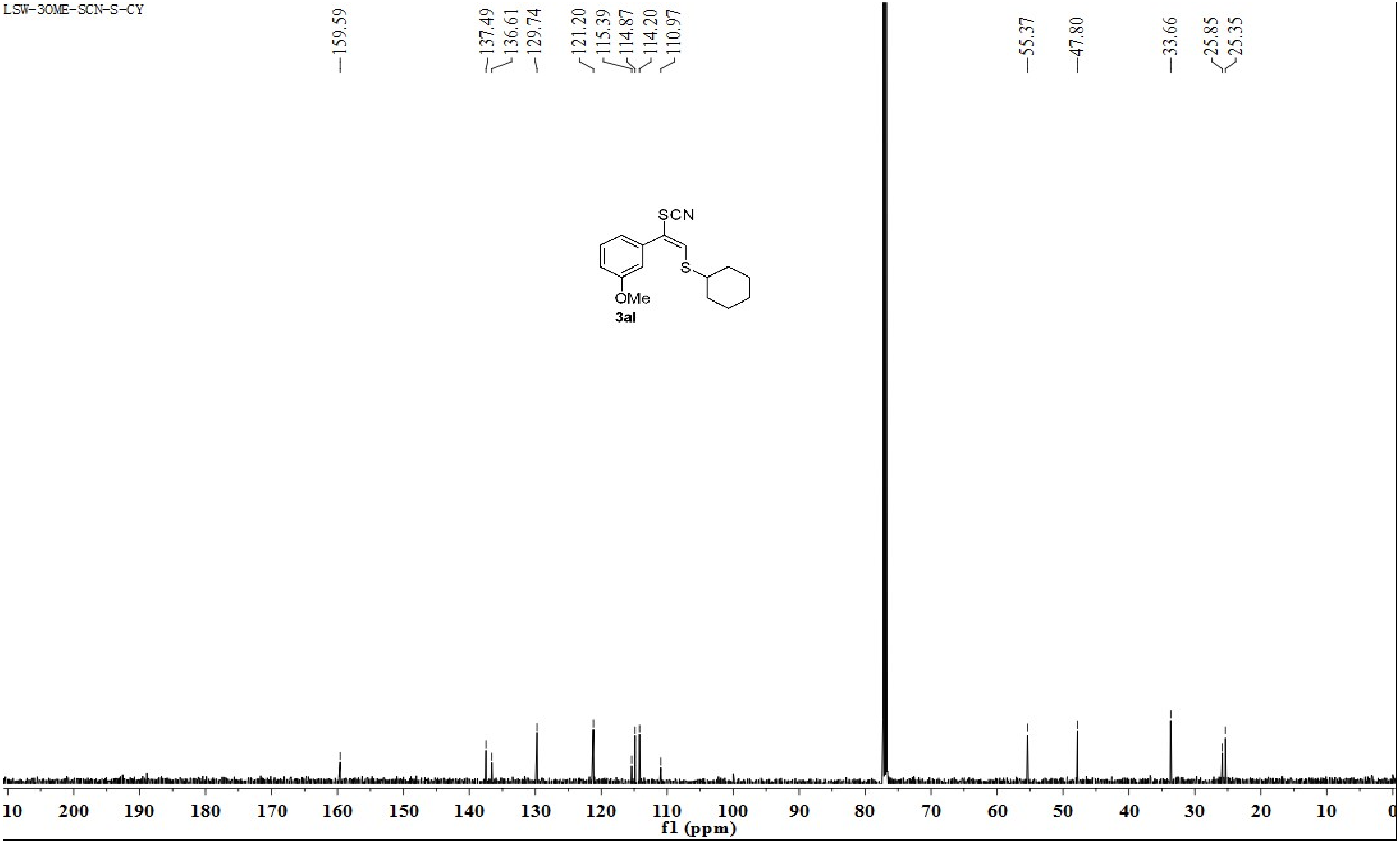

78. ^{1}H NMR of **3am** (600 MHz, $CDCl_3$)

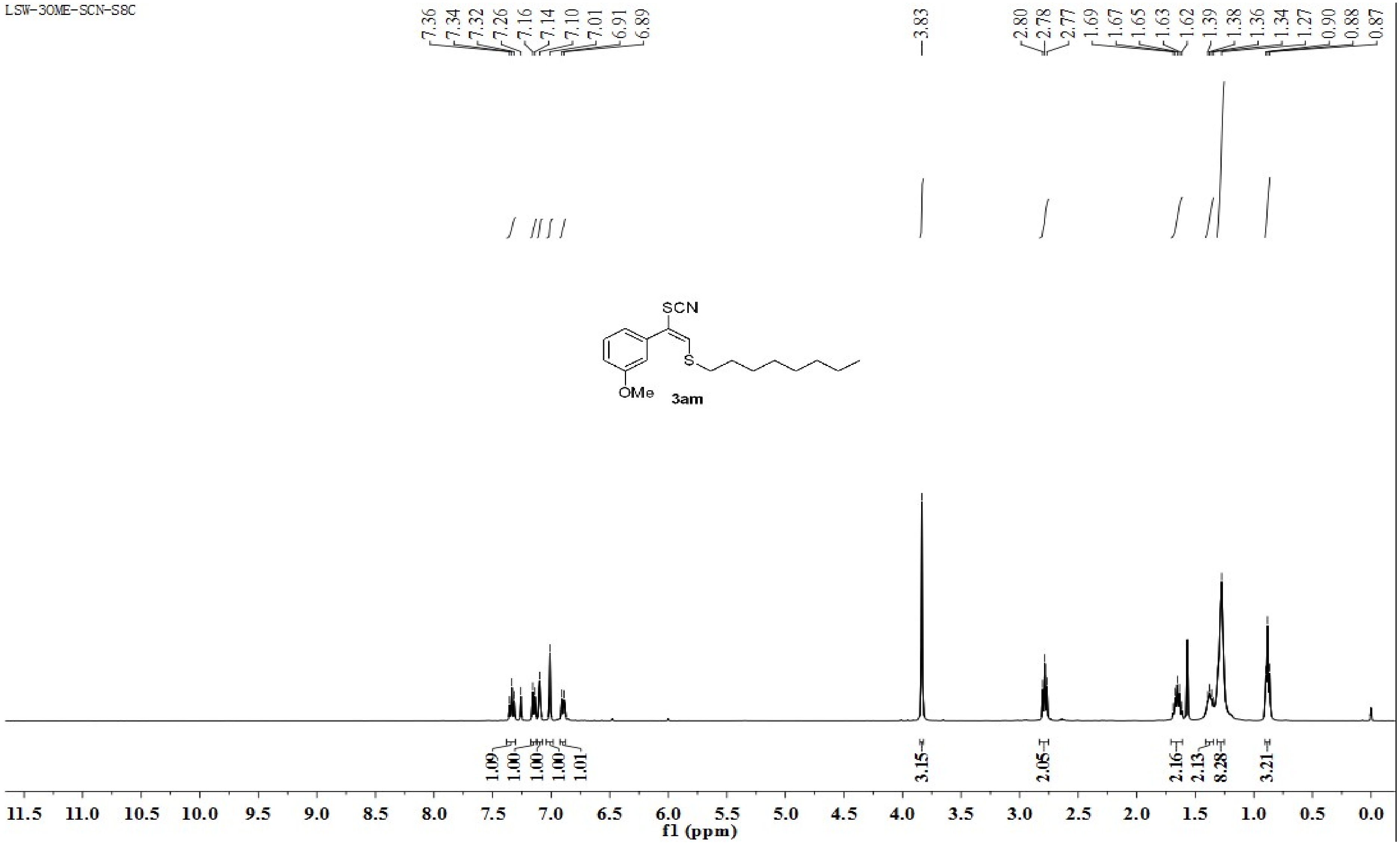

79. ^{13}C NMR of **3am** (600 MHz, $CDCl_3$)

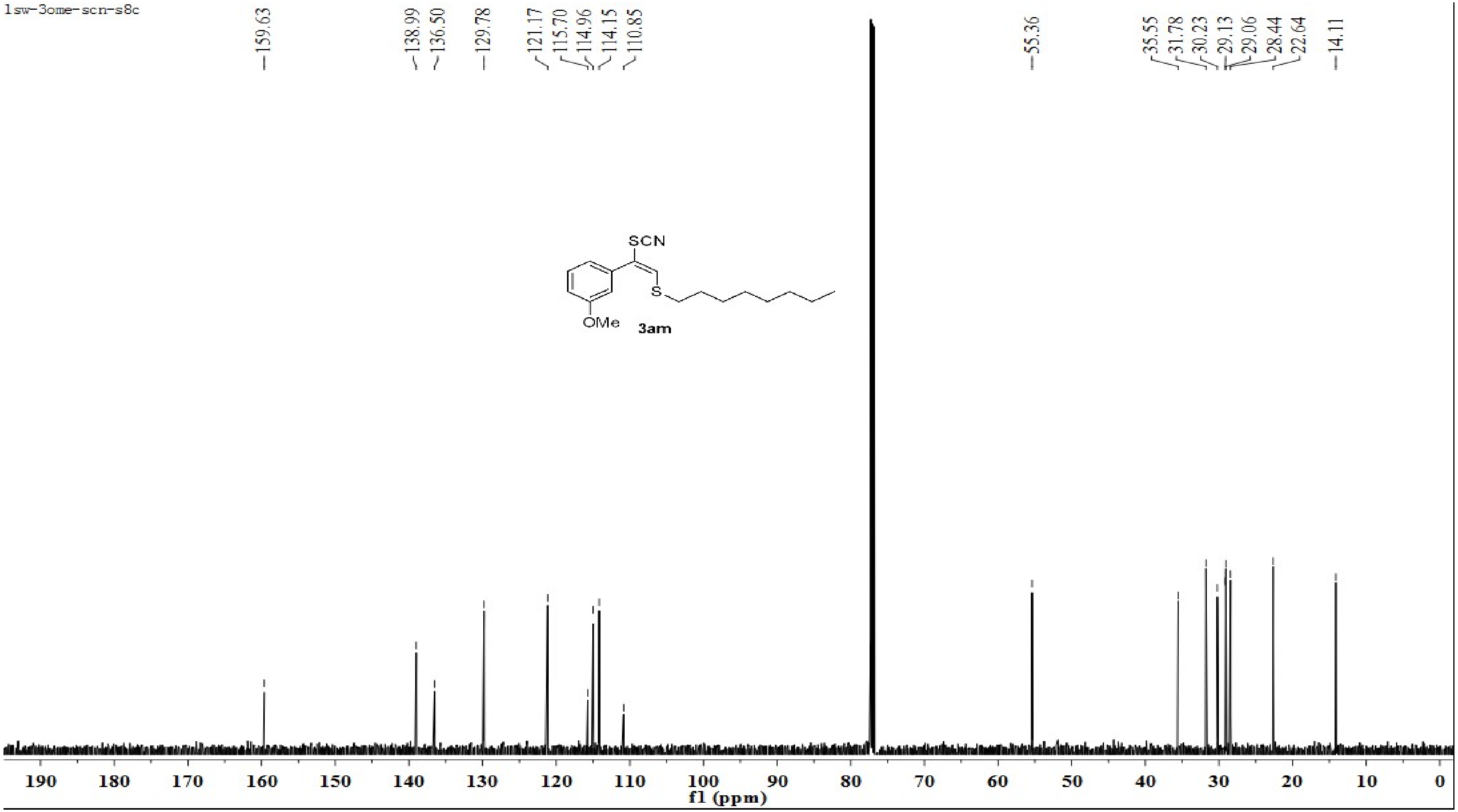

80.^{1}H NMR of **3an** (600 MHz, $CDCl_3$)

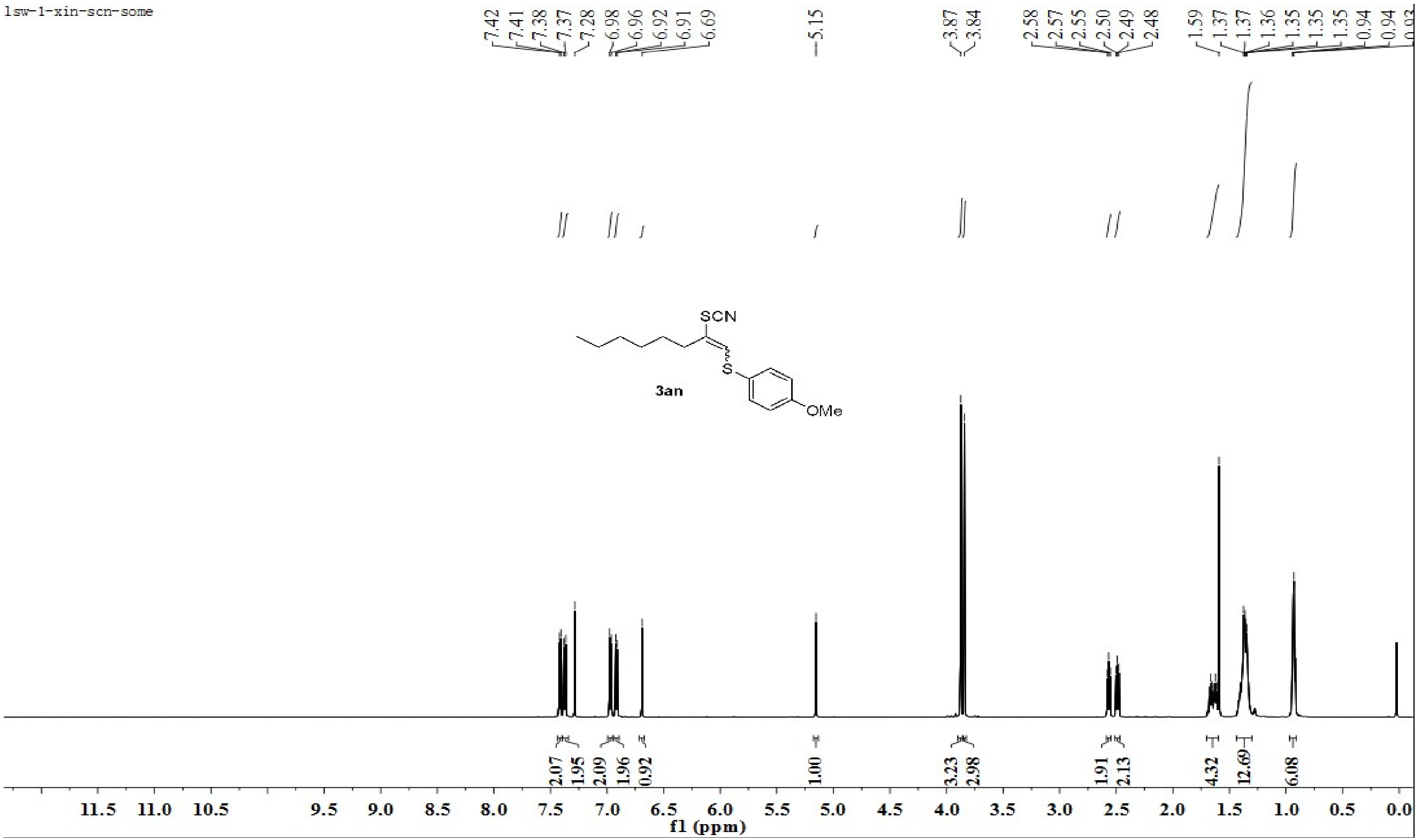

81. ^{13}C NMR of **3an** (125 MHz, $CDCl_3$)

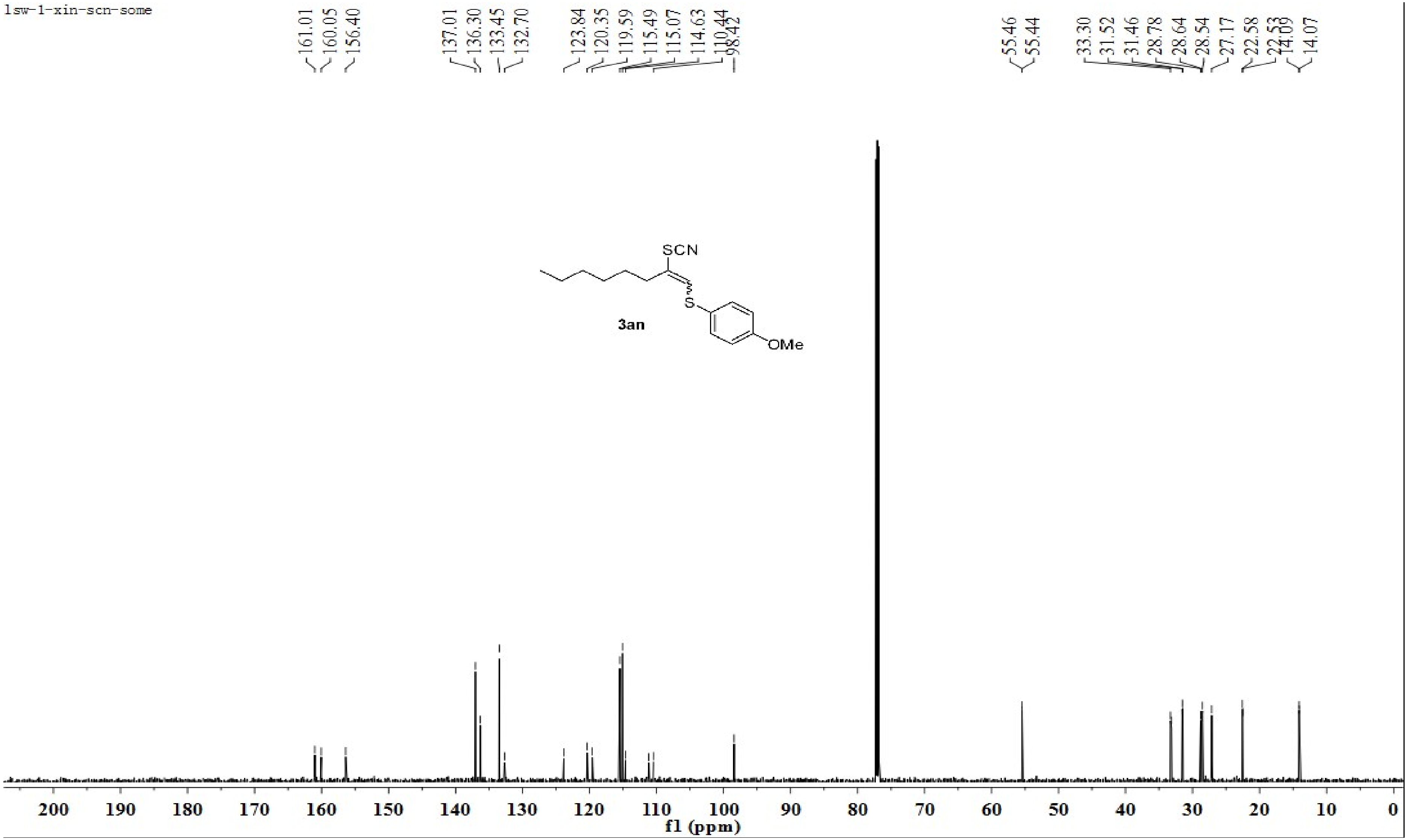

82. ^{1}H NMR of **3ao** (600 MHz, $CDCl_3$)

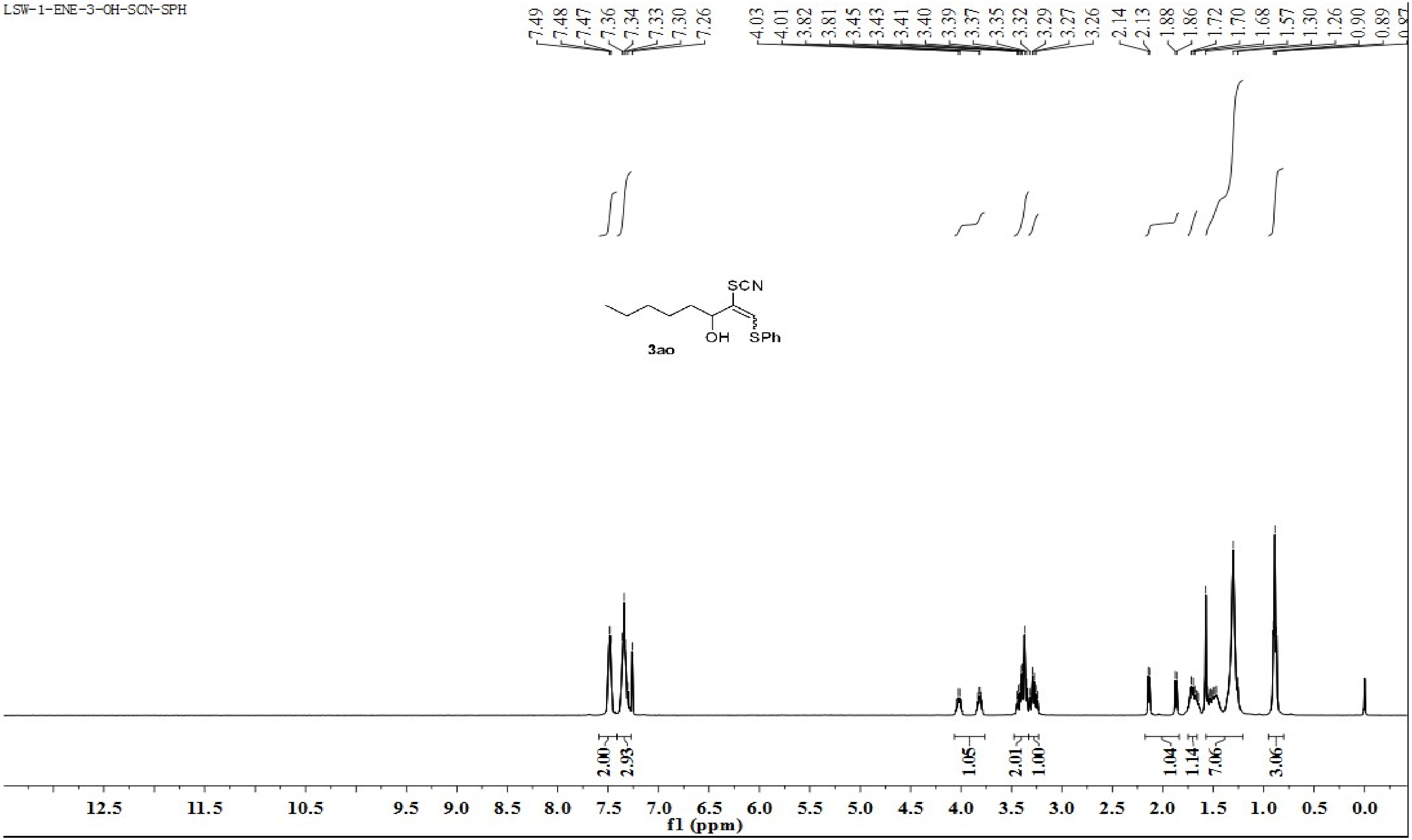

83. ^{13}C NMR of **3ao** (125 MHz, $CDCl_3$)

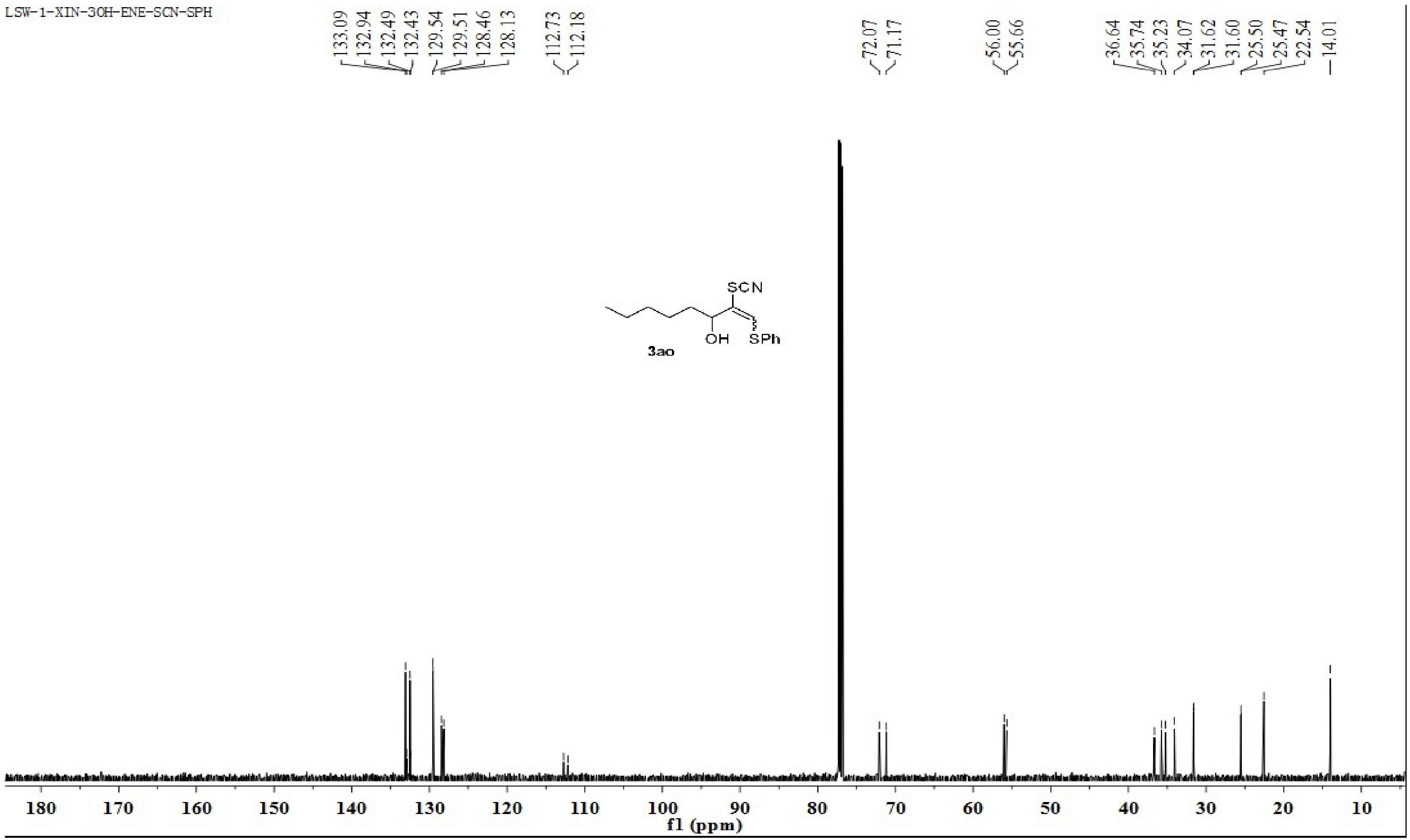

84. ^{1}H NMR of **3ap** (600 MHz, $CDCl_3$)

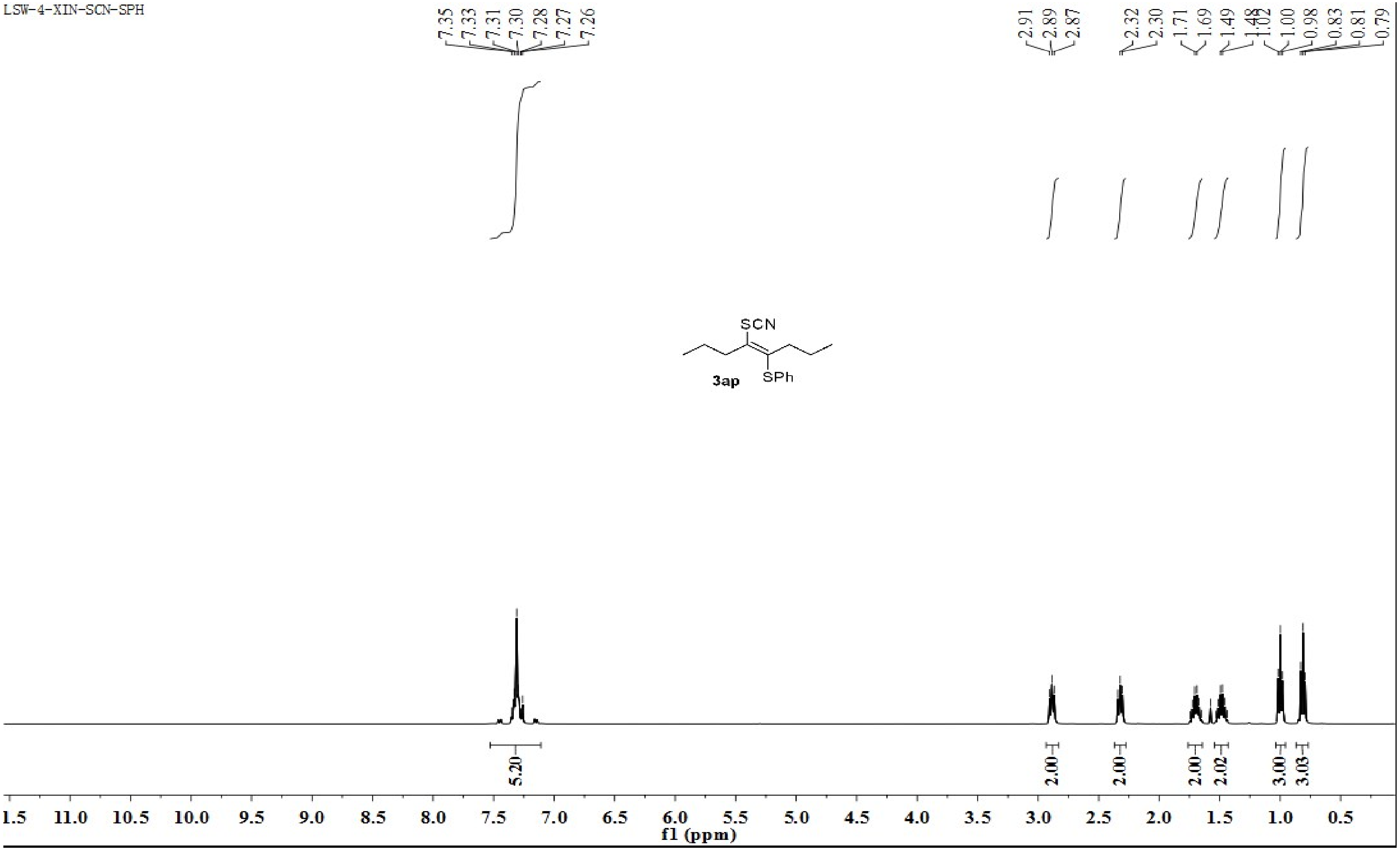

85. ^{13}C NMR of **3ap** (125 MHz, $CDCl_3$)

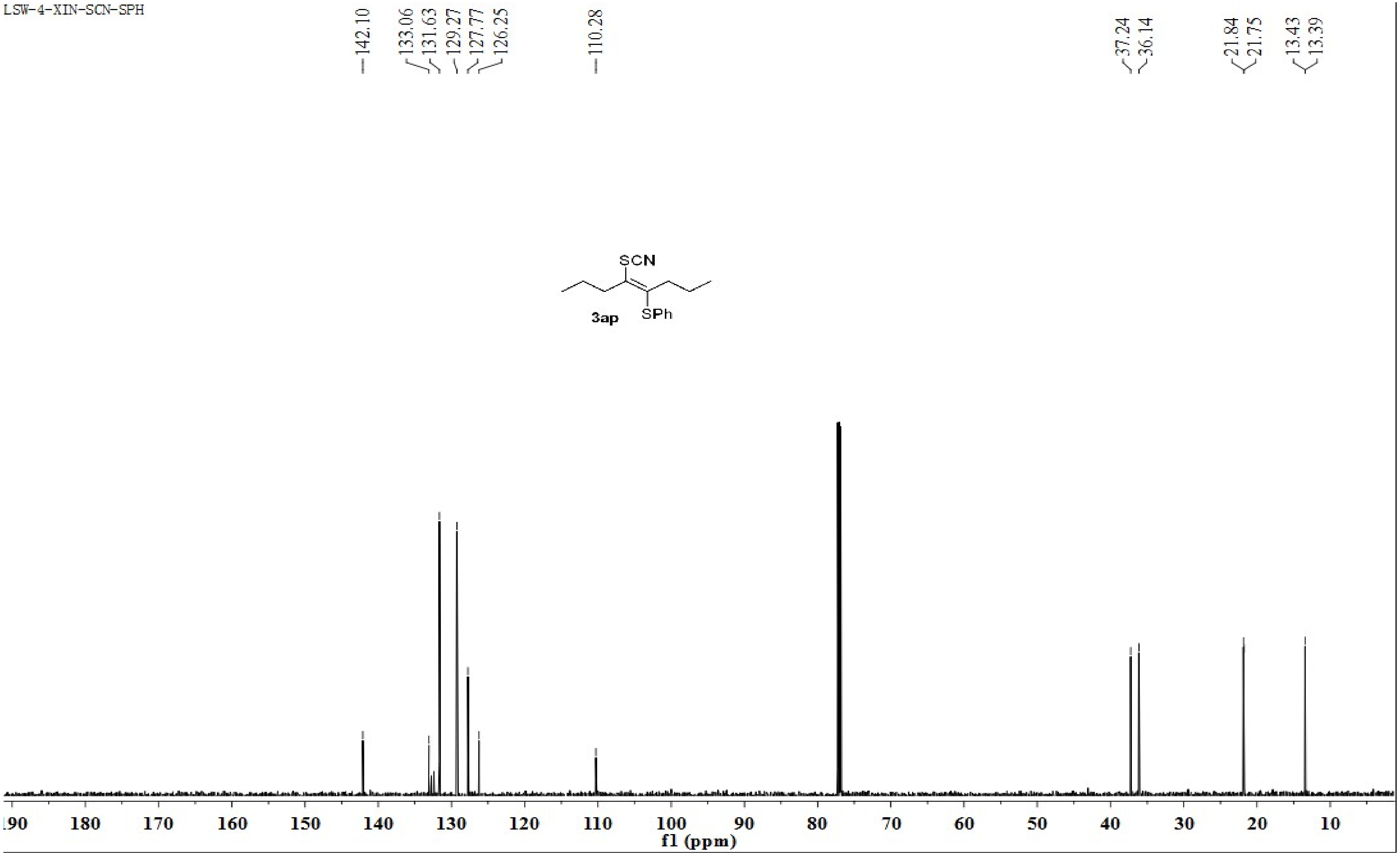

86. ^{1}H NMR of **3aq** (600 MHz, $CDCl_3$)

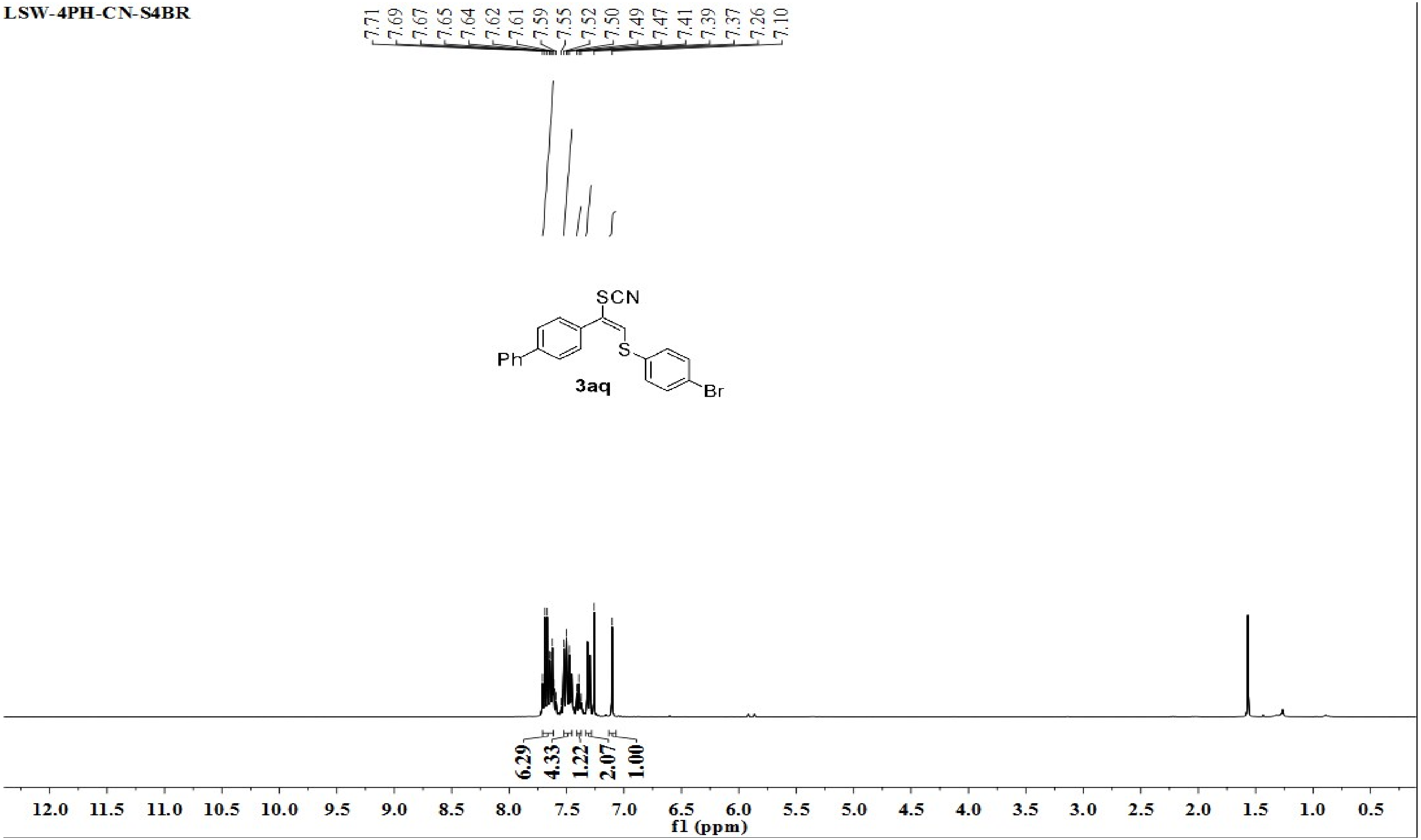

87. ^{3}C NMR of **3aq** (125 MHz, $CDCl_3$)

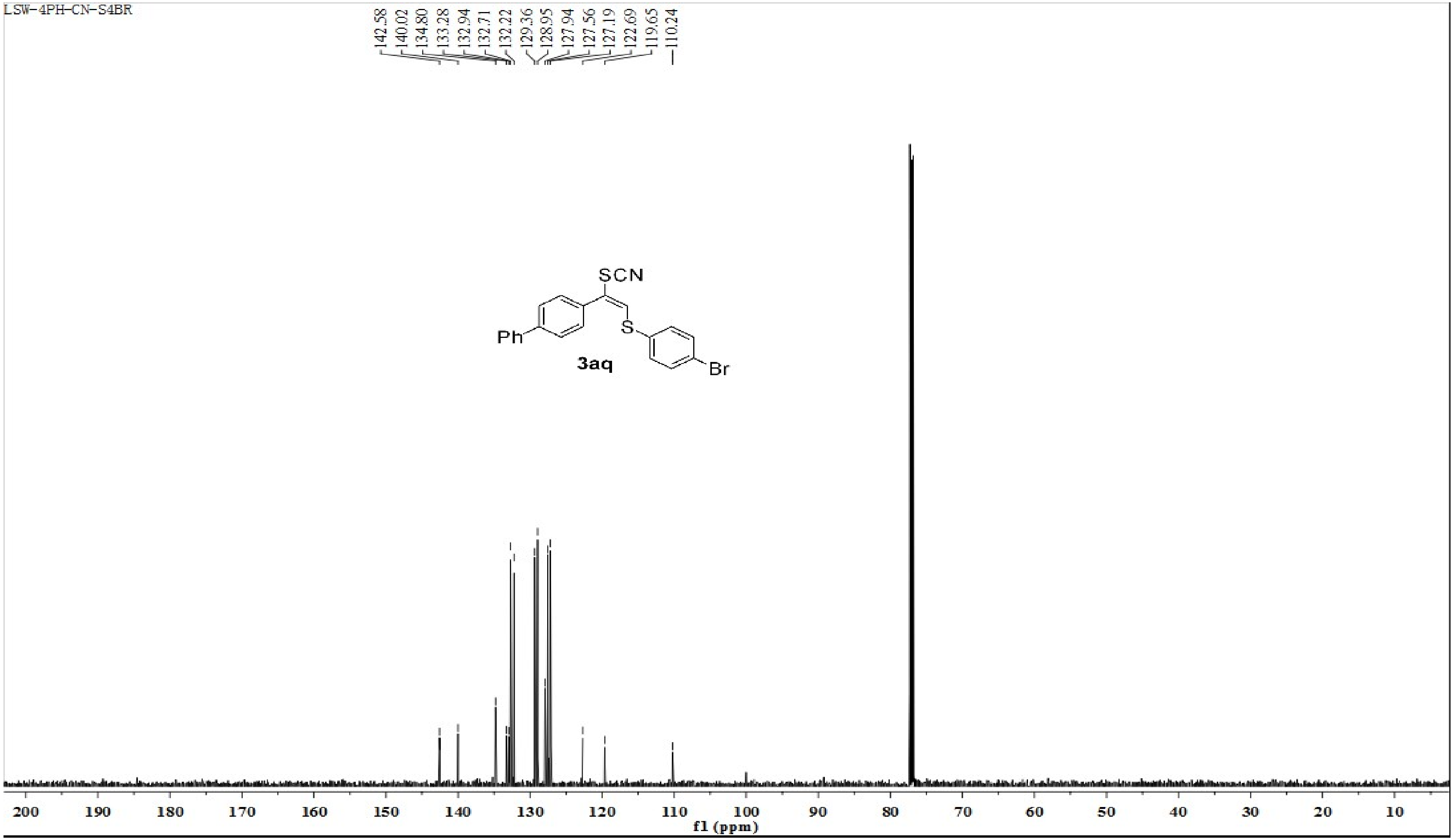

88. ^{1}H NMR of **5a** (600 MHz, $CDCl_3$)

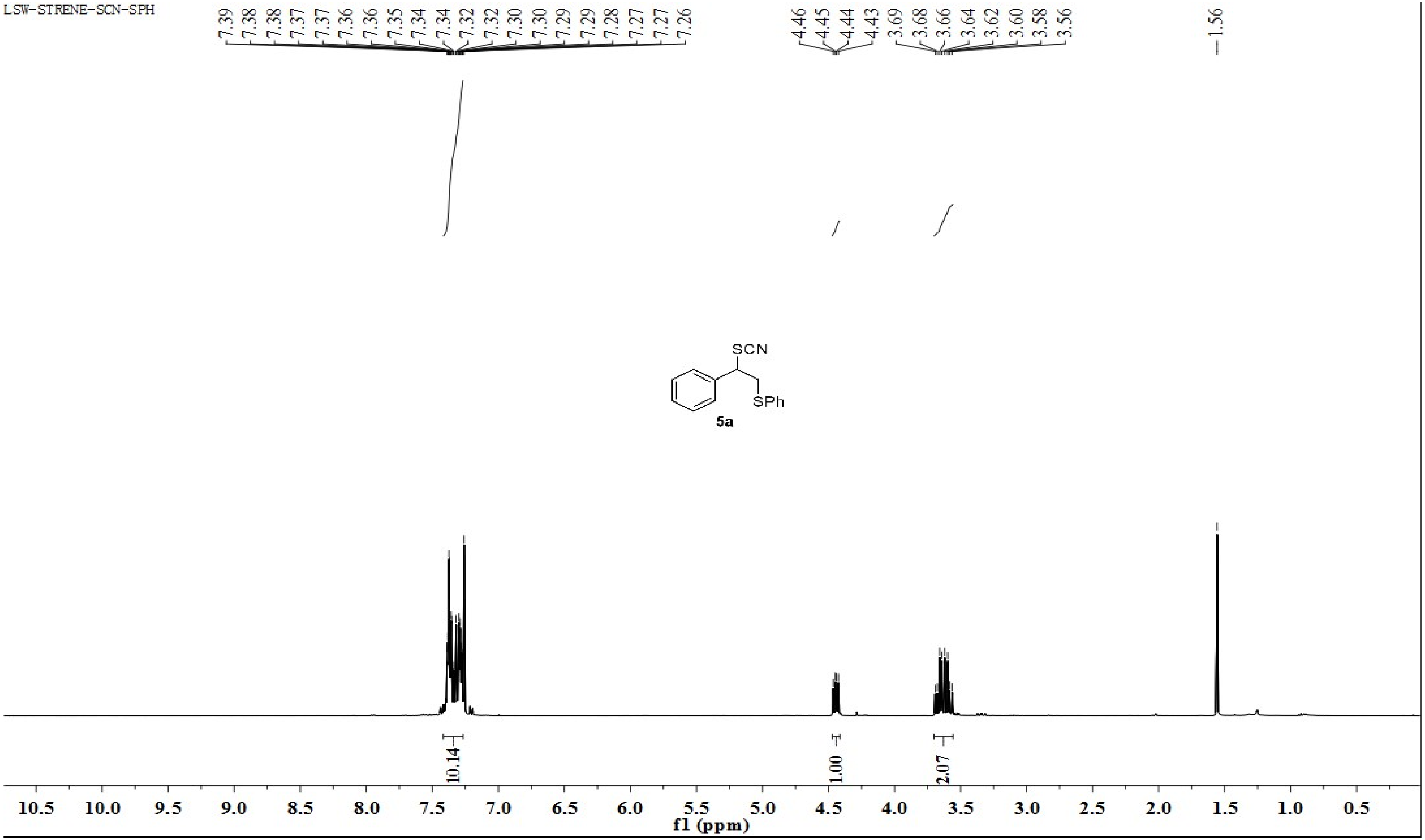

89. ^{13}C NMR of **5a** (125 MHz, $CDCl_3$)

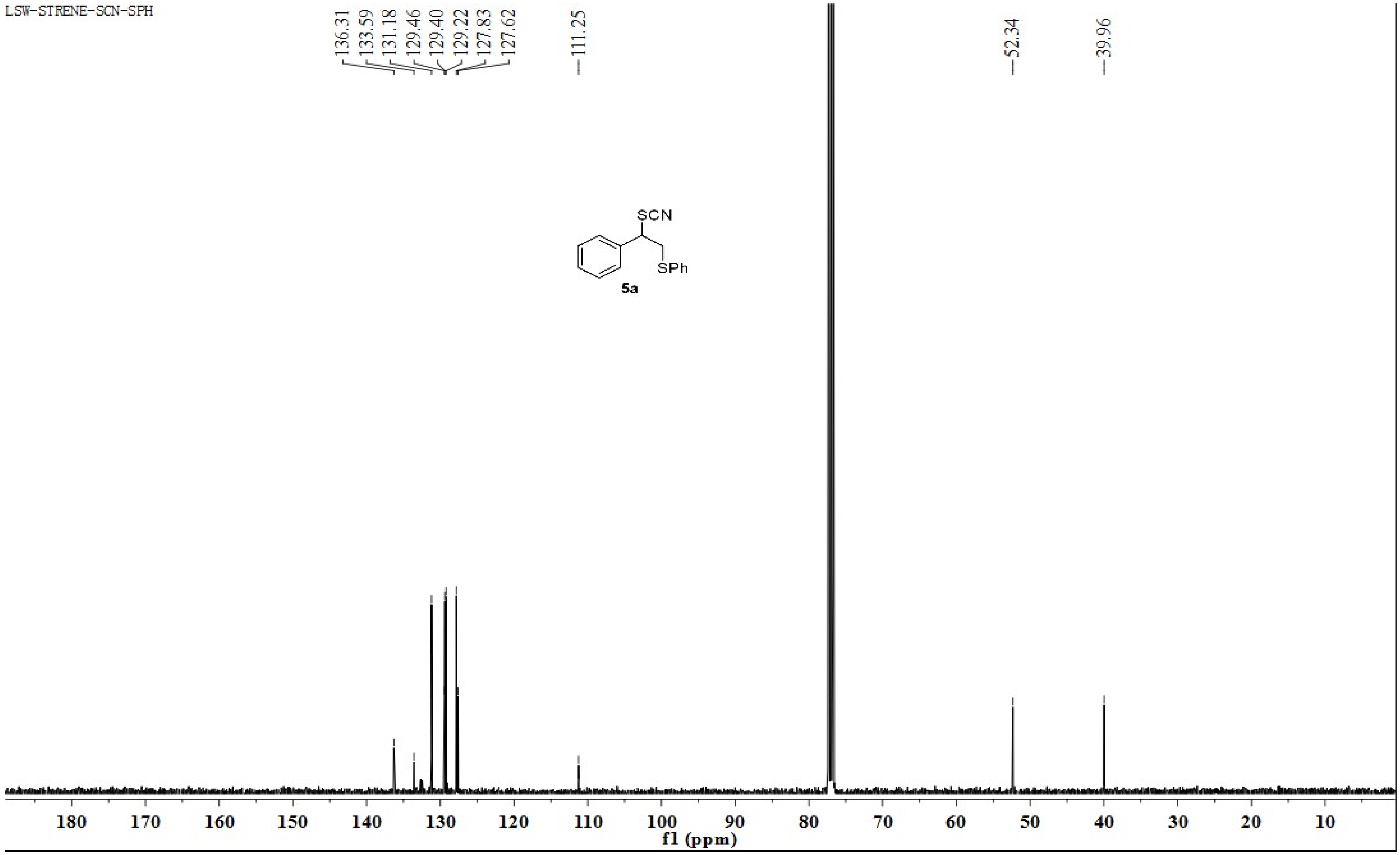

90. ^{1}H NMR of **5b** (600 MHz, $CDCl_3$)

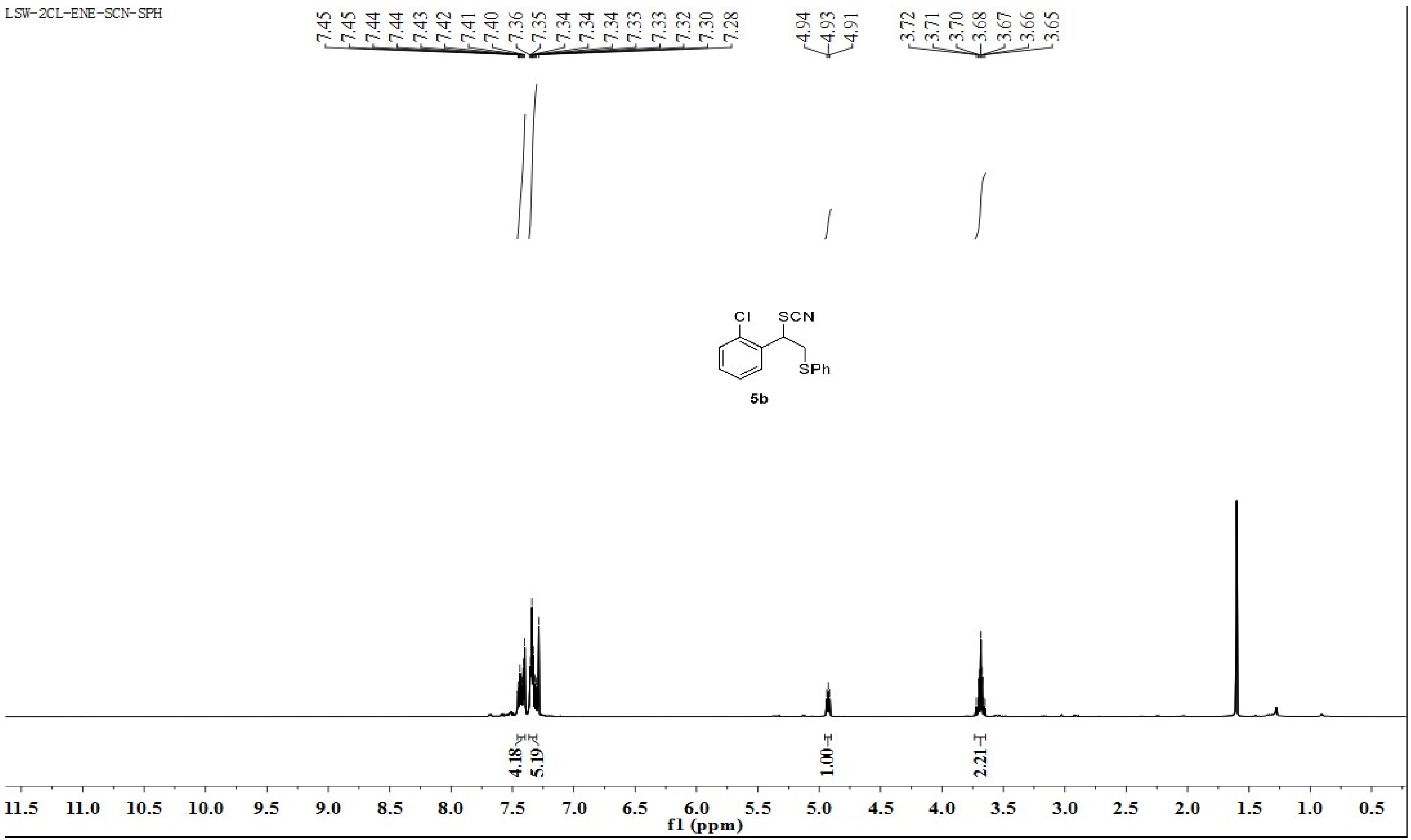

91. ^{13}C NMR of **5b** (125 MHz, $CDCl_3$)

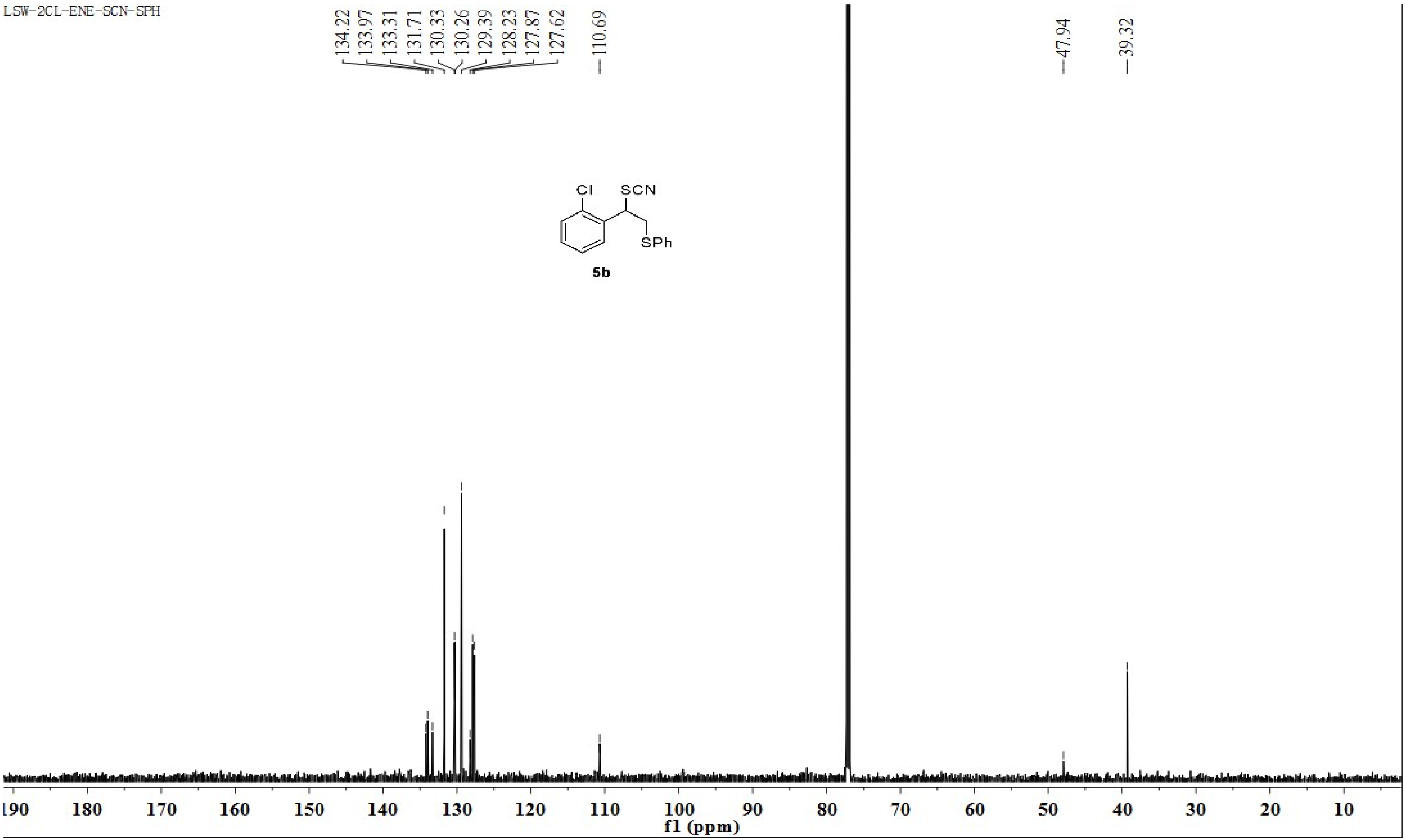

92. ^{1}H NMR of **5c** (600 MHz, $CDCl_3$)

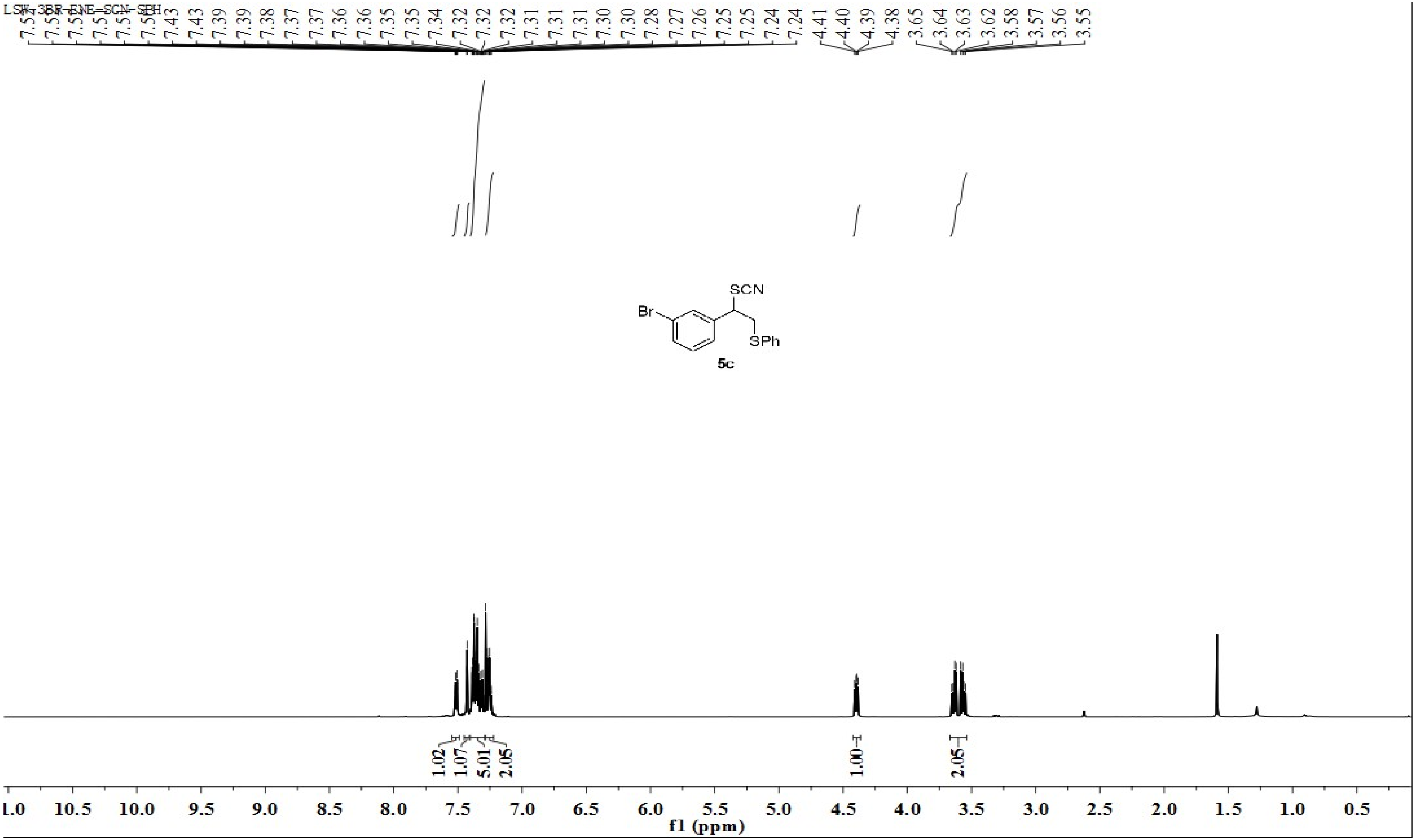

93. ^{13}C NMR of **5c** (125 MHz, $CDCl_3$)

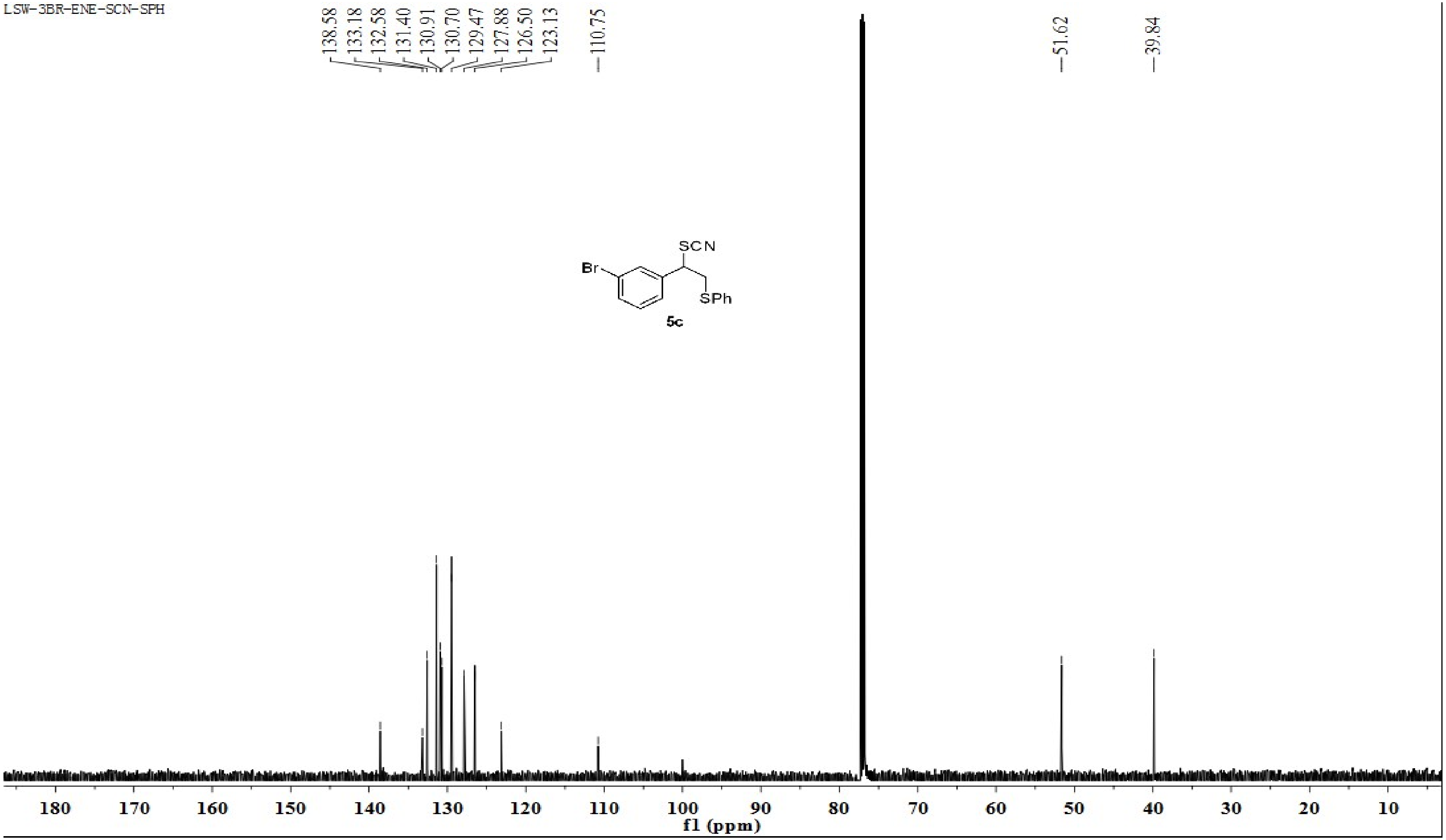

94. ^{1}H NMR of **5d** (600 MHz, $CDCl_3$)

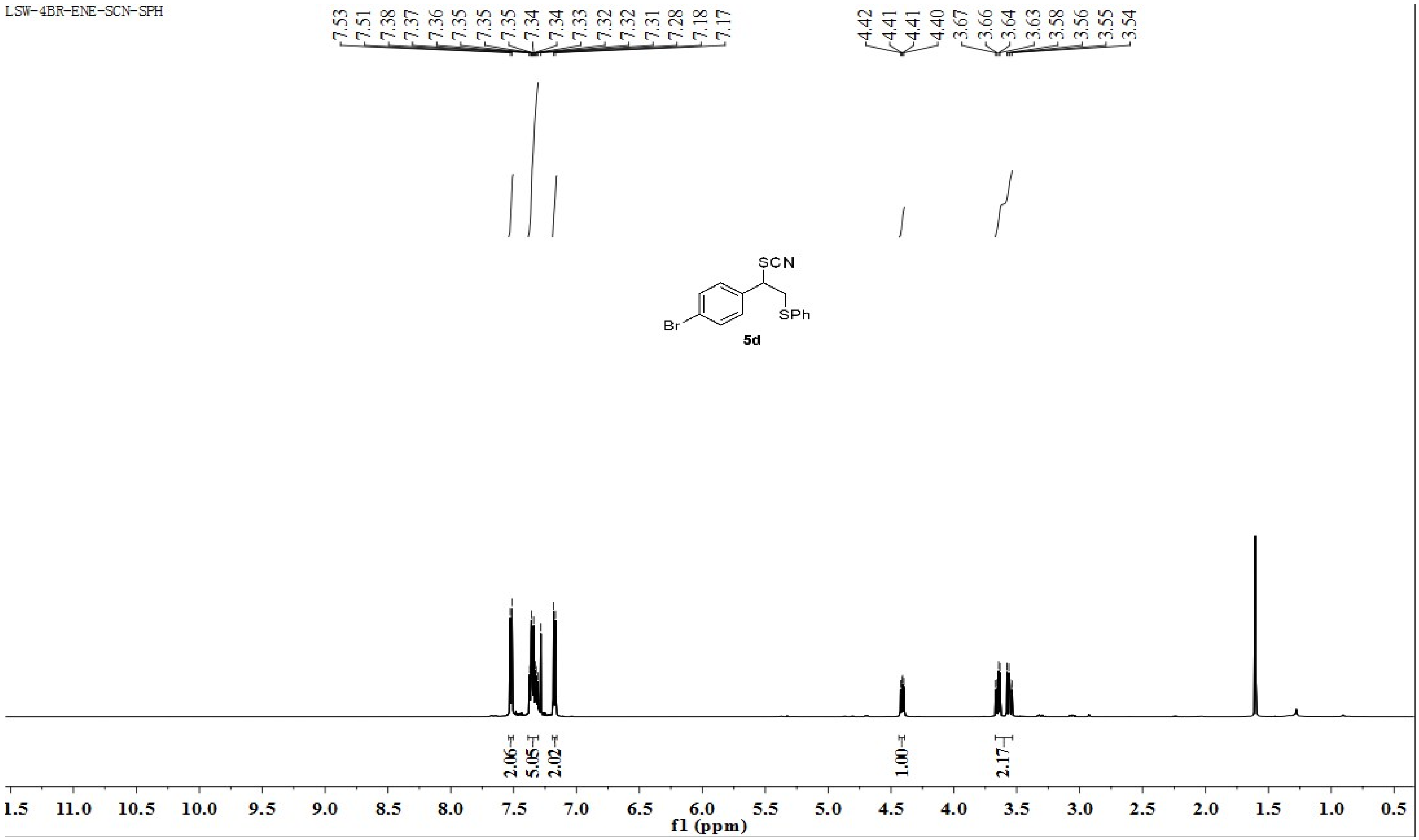

95. ^{13}C NMR of **5d** (125 MHz, $CDCl_3$)

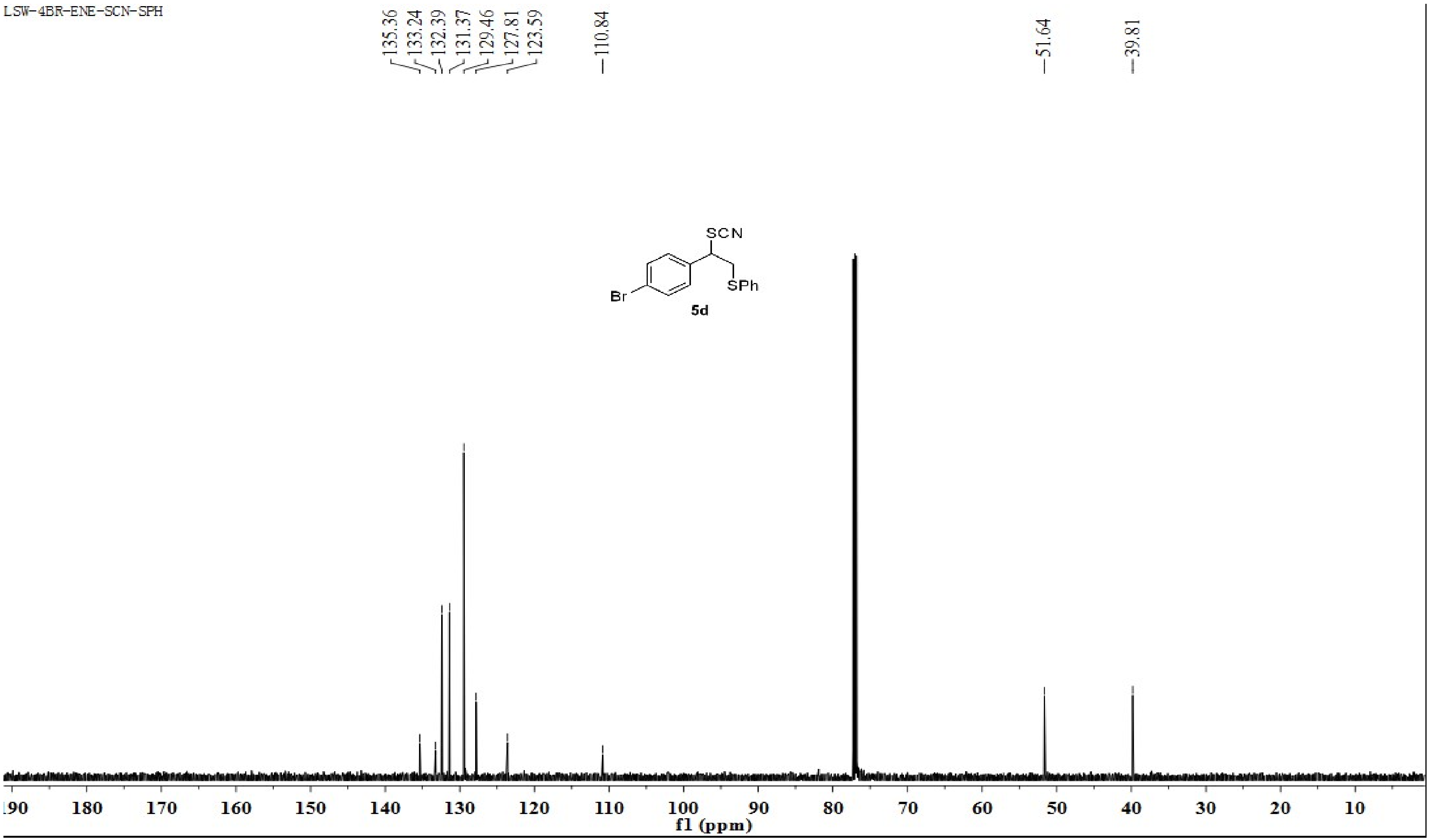

96. ^{1}H NMR of **5e** (600 MHz, $CDCl_3$)

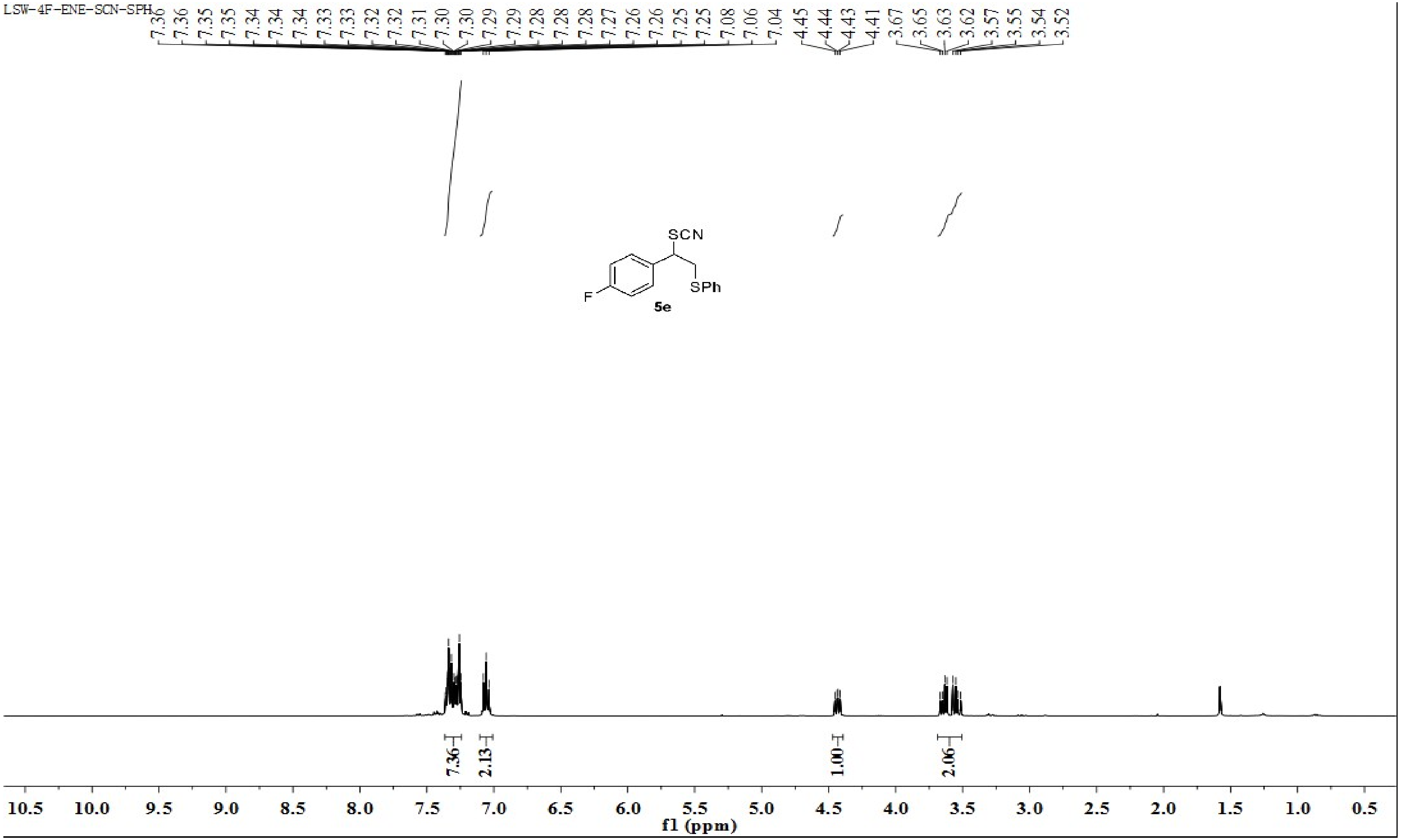

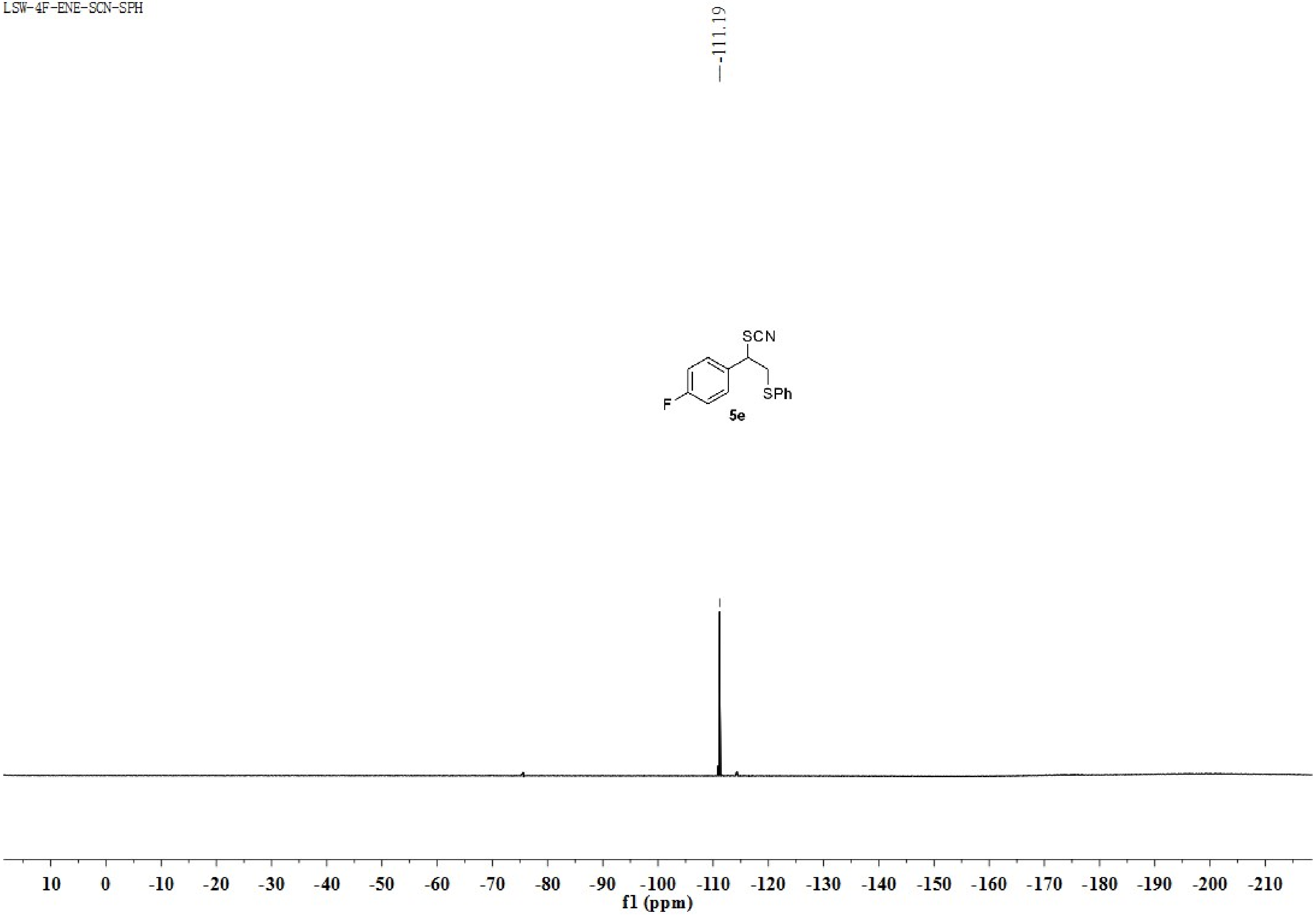
LSW-4F-ENE-SCN-SPH
-111.19
SCN
SPh
F
5e
10 0 -10 -20 -30 -40 -50 -60 -70 -80 -90 -100 -110 -120 -130 -140 -150 -160 -170 -180 -190 -200 -210
f1 (ppm)

97. ^{13}C NMR of **5e** (125 MHz, $CDCl_3$)

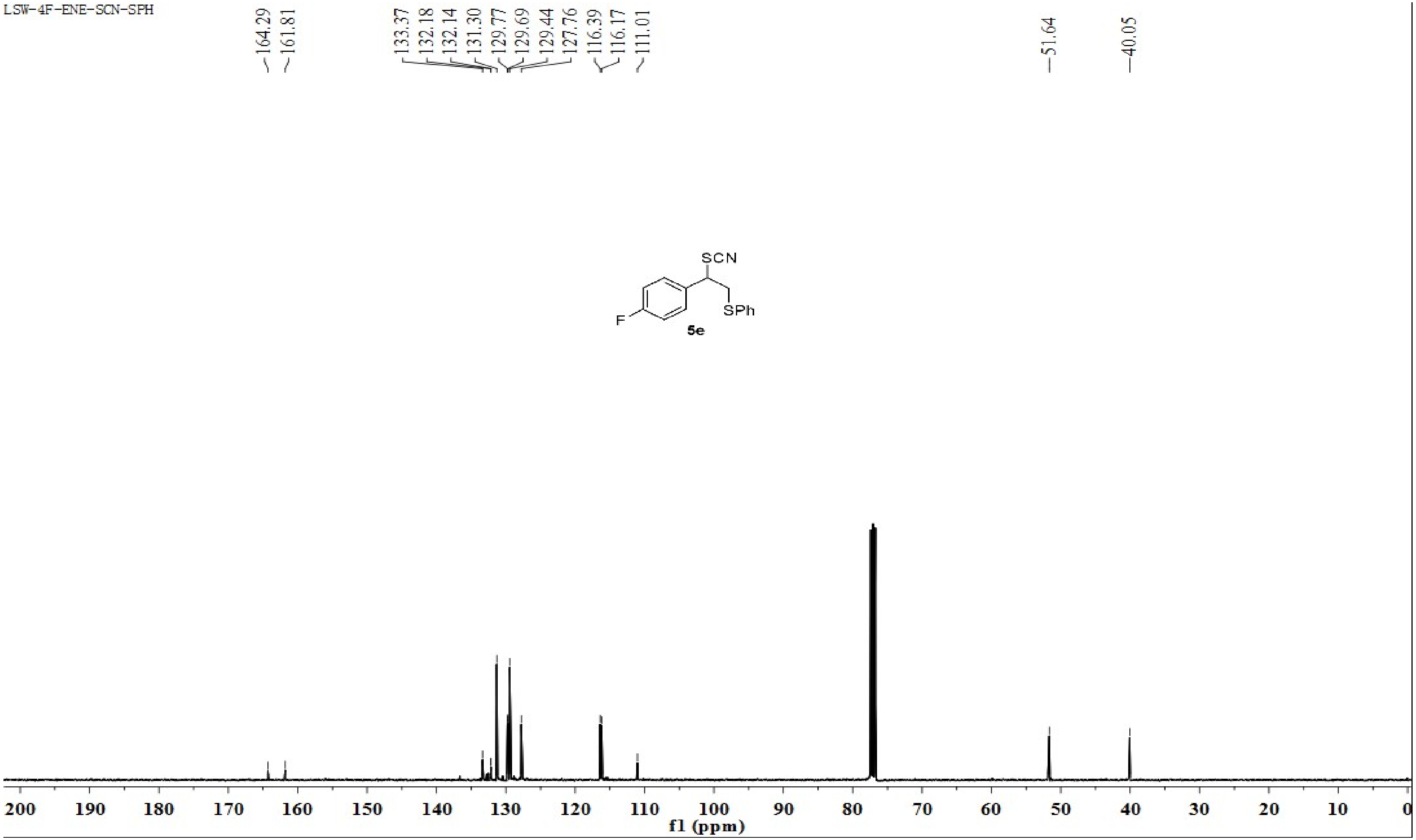

98. ^{1}H NMR of **5f** (600 MHz, $CDCl_3$)

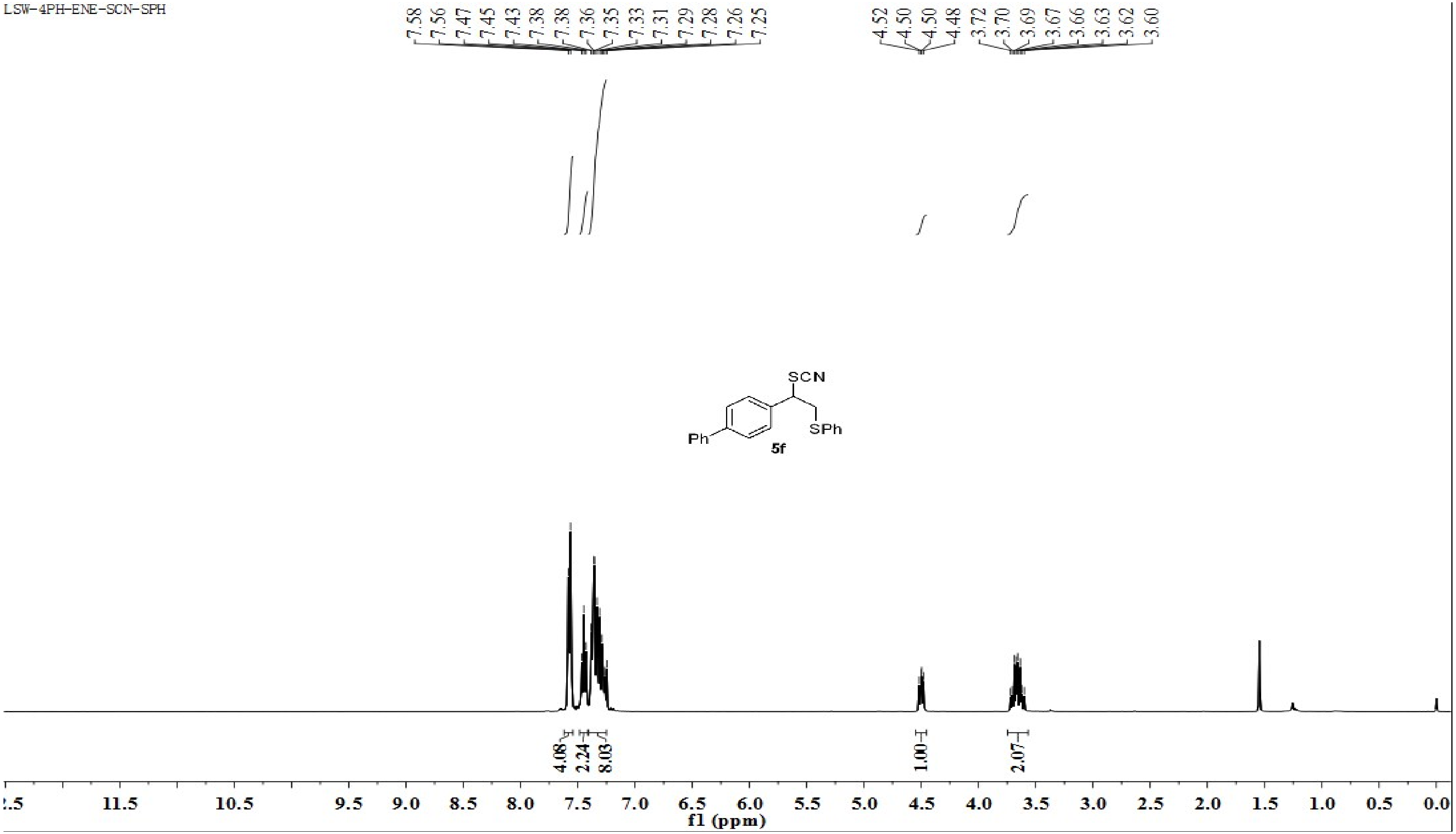

99. ^{13}C NMR of **5f** (125 MHz, $CDCl_3$)

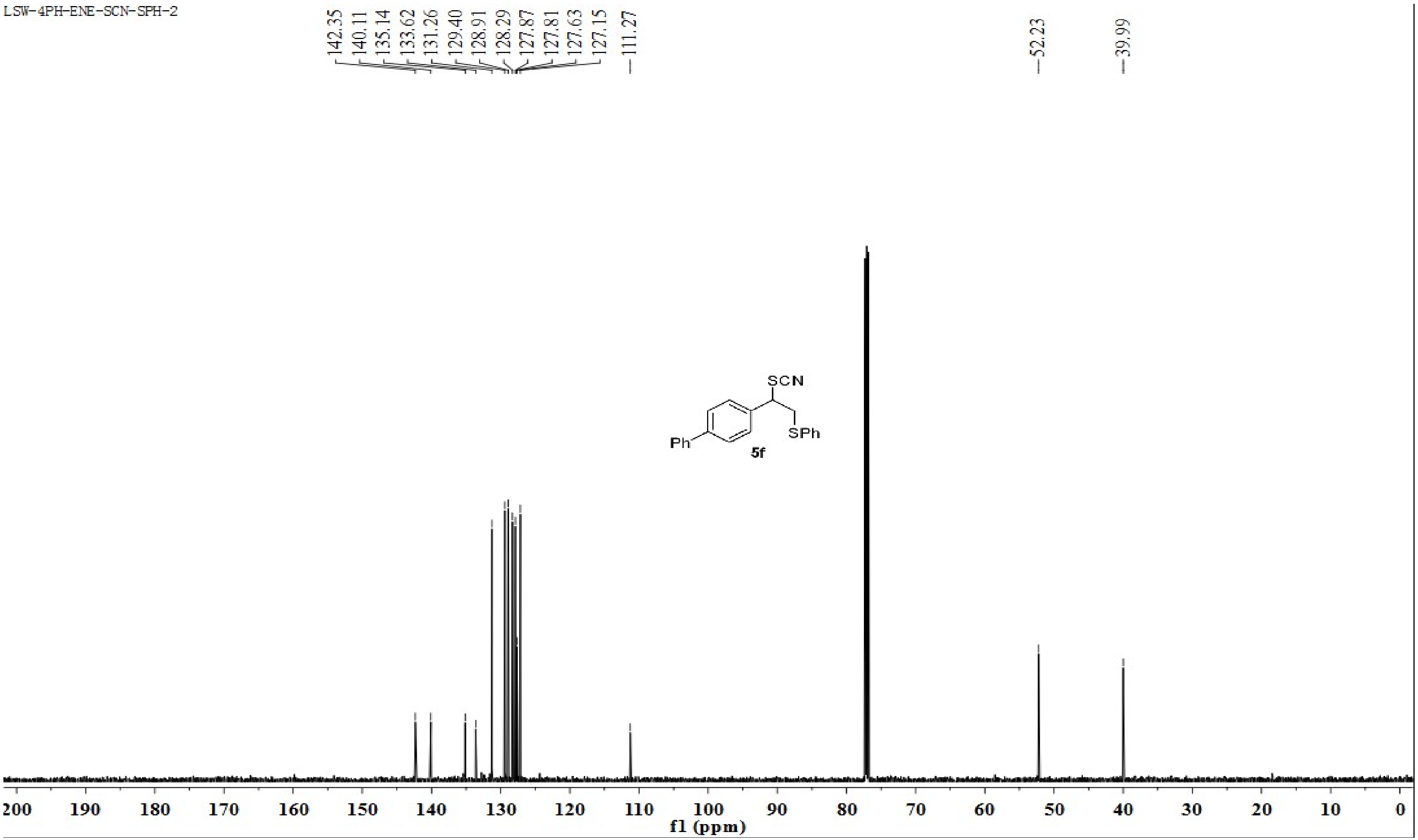

100. ^{1}H NMR of **5g** (600 MHz, $CDCl_3$)

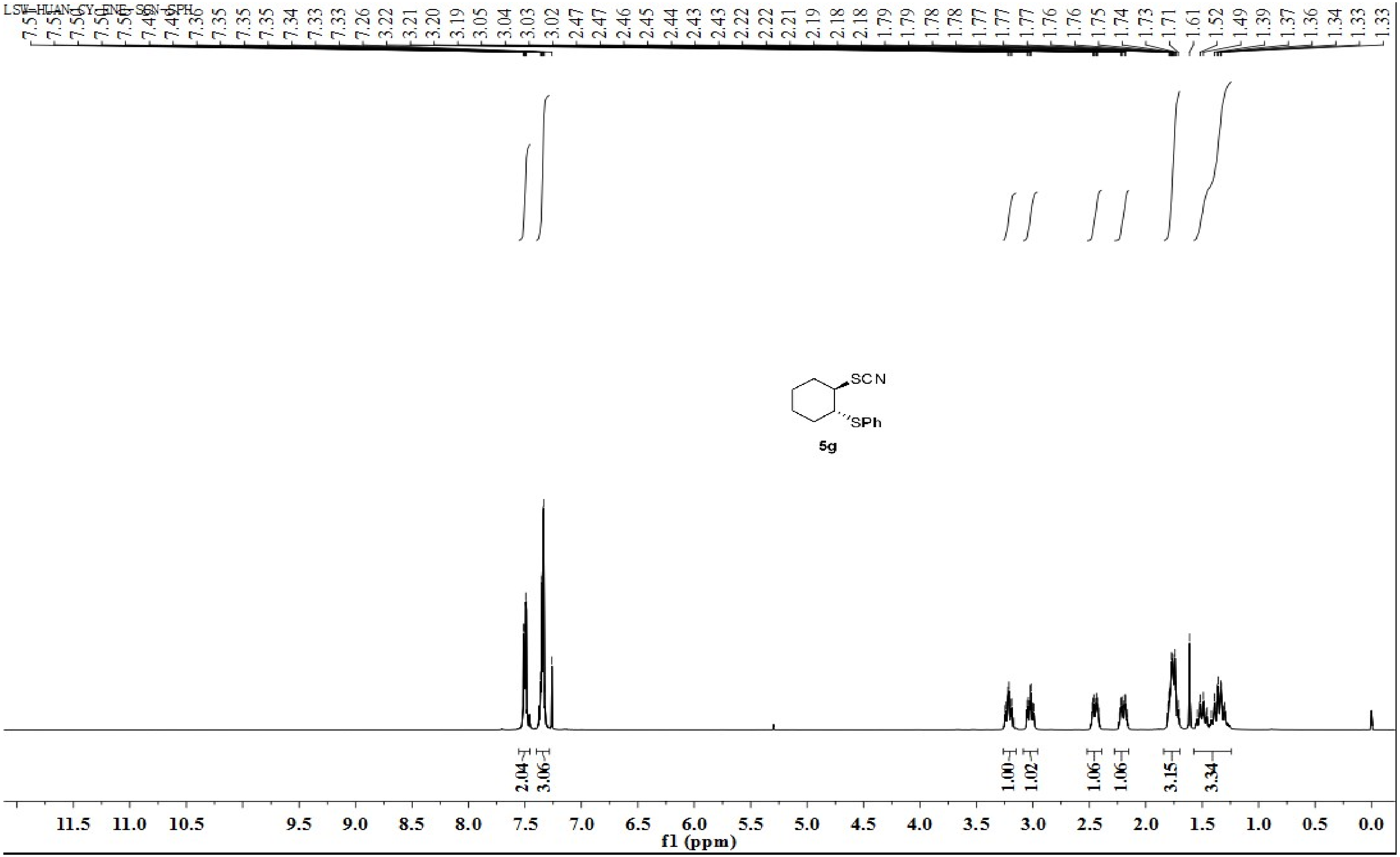

101. ^{13}C NMR of **5g** (125 MHz, $CDCl_3$)

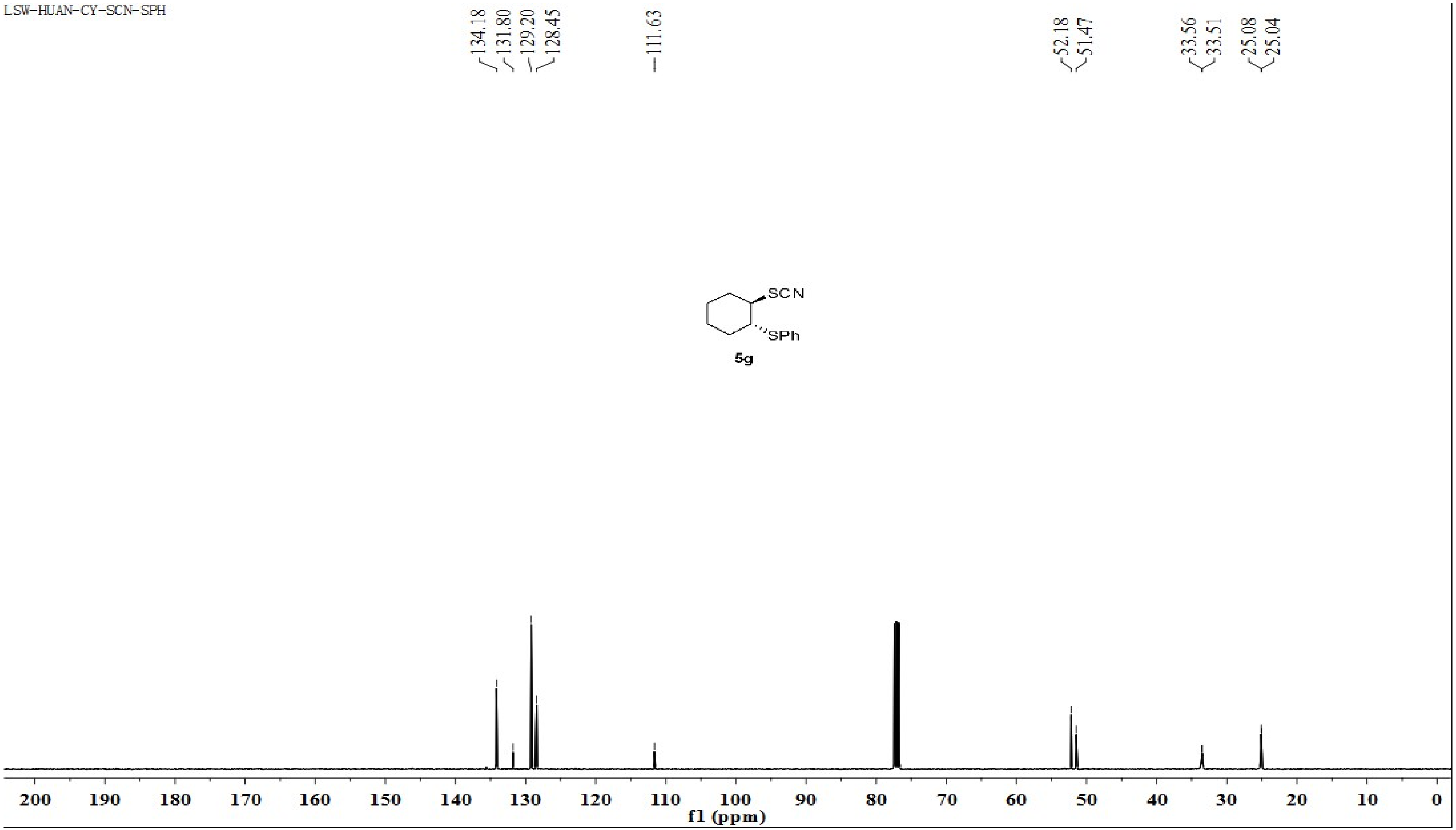

.102. ^{1}H NMR of **5h** (600 MHz, $CDCl_3$)

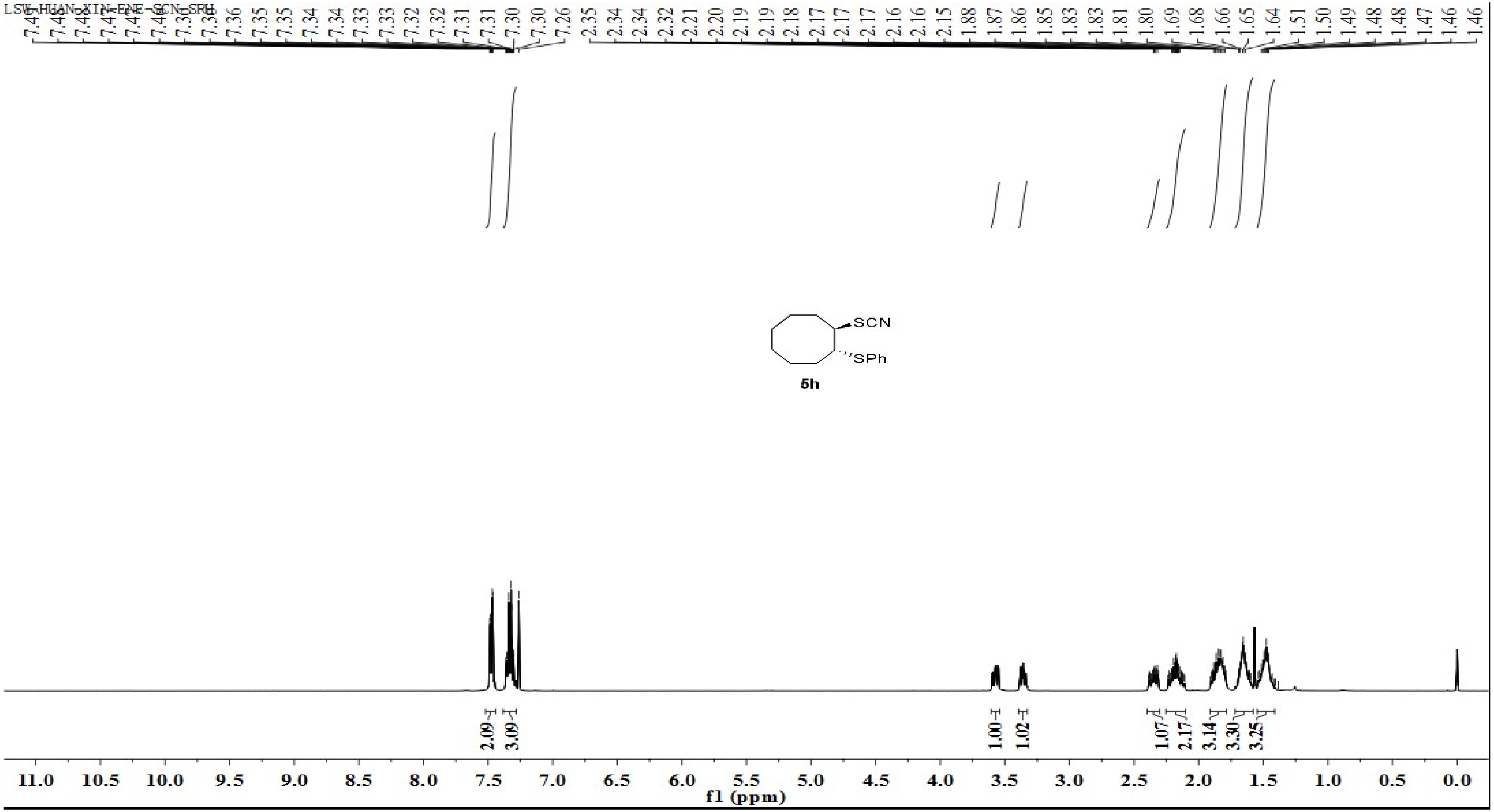

103. ^{13}C NMR of **5h** (125 MHz, $CDCl_3$)

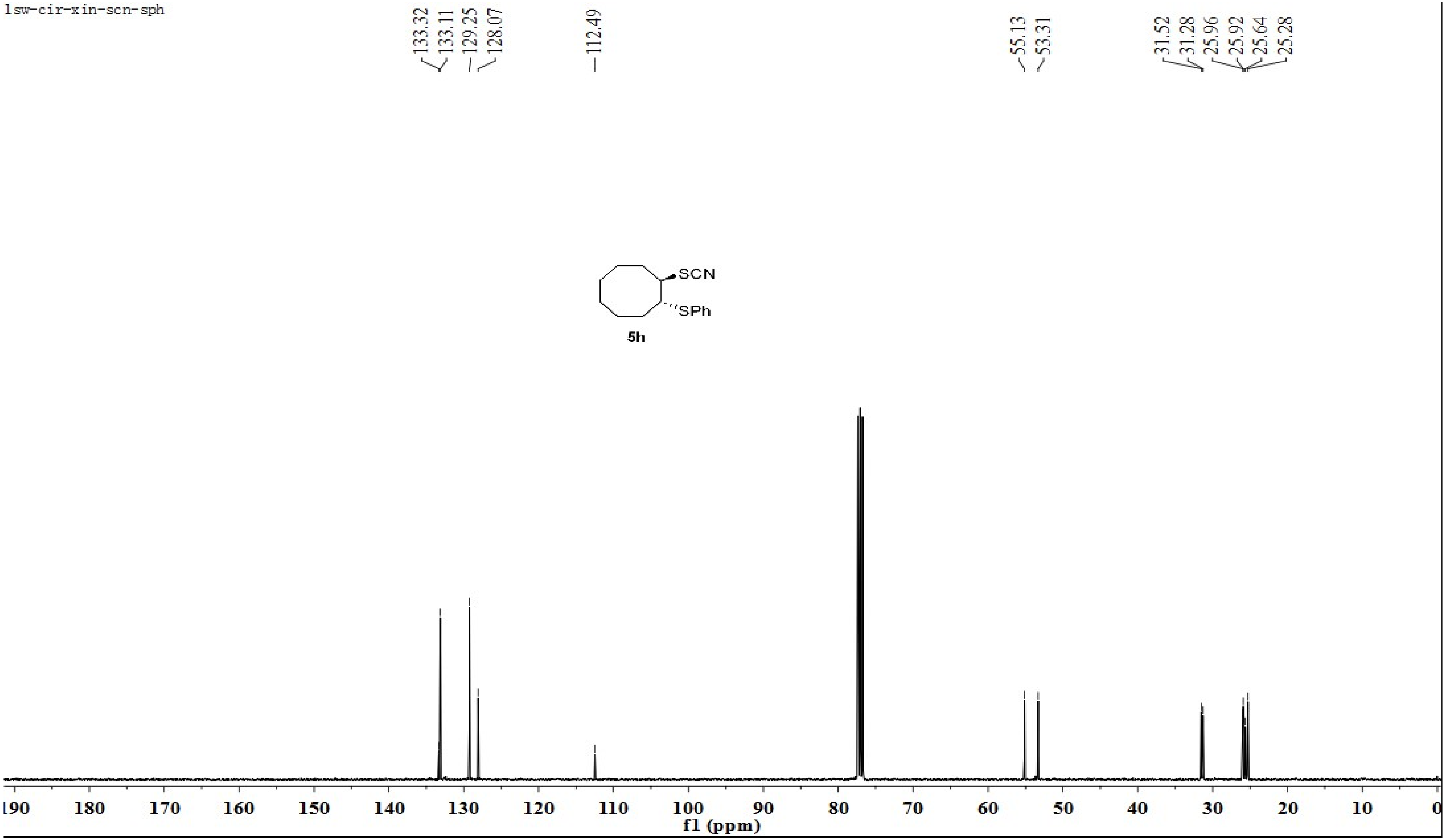

9. References

[1] C. J. Nalbandian, E. M. Miller, S. T. Toenjes, J. L. Gustafson, *Chemical Communications* **2017**, *53*, 1494-1497.
